# Electrochemical Deconstructive Functionalization of Cycloalkanols via Alkoxy Radicals Enabled by Proton-Coupled Electron Transfer

**DOI:** 10.1021/acs.orglett.2c01552

**Published:** 2022-05-23

**Authors:** Mishra Deepak Hareram, Albara A. M. A. El Gehani, James Harnedy, Alex C. Seastram, Andrew C. Jones, Matthew Burns, Thomas Wirth, Duncan L. Browne, Louis C. Morrill

**Affiliations:** † Cardiff Catalysis Institute, School of Chemistry, Cardiff University, Main Building, Park Place, Cardiff, CF10 3AT, United Kingdom; ‡ Chemical Development, Pharmaceutical Technology & Development, Operations, AstraZeneca, Macclesfield, SK10 2NA, United Kingdom; § School of Chemistry, Cardiff University, Main Building, Park Place, Cardiff, CF10 3AT, United Kingdom; ∥ Department of Pharmaceutical and Biological Chemistry, University College London, School of Pharmacy, London, W1CN 1AX, United Kingdom

## Abstract

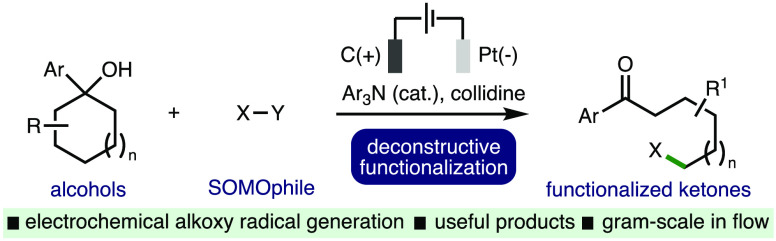

Herein, we report a new electrochemical method for alkoxy radical generation from alcohols using a proton-coupled electron transfer (PCET) approach, showcased via the deconstructive functionalization of cycloalkanols. The electrochemical method is applicable across a diverse array of substituted cycloalkanols, accessing a broad range of synthetically useful distally functionalized ketones. The orthogonal derivatization of the products has been demonstrated through chemoselective transformations, and the electrochemical process has been performed on a gram scale in continuous single-pass flow.

Alkoxy radicals are an important class of oxygen-centered radicals, which consist of an alkyl group bound to an electrophilic oxygen radical center.^1^ They are particularly high energy species due to the lack of stabilization provided by mesomeric effects and spin density delocalization found in other *O*-centered radicals, such as aryloxy radicals. Despite this, alkoxy radicals exhibit well-defined yet diverse reactivity, including β-scission processes, hydrogen atom transfers (HATs), and addition to π-systems (Scheme 1A). As such, these privileged intermediates have been successfully employed across a plethora of powerful transformations spanning selective C–H functionalization, C–C bond activation, and heterocycle synthesis to access valuable products, including within complex molecule synthesis.^2^ Photoredox catalysis has enabled alkoxy radical generation employing various radical precursors including peroxides,^3^*N*-alkoxyphthalimides,^4^*N*-alkoxypyridiniums,^5^*N*-alkoxybenzimidazoles,^6^*N*-alkoxytriazoliums,^7^ and oxyimino acids.^8^ Nevertheless, the use of *O*-functionalized precursors incurs additional synthetic effort for installation, particularly within more complex molecules, and decreases step/atom economy.^9^ Recent advances in transition metal catalysis and photoredox catalysis have enabled alkoxy radical intermediates to be accessed directly from unprotected aliphatic alcohols (Scheme 1B).^1^ However, many of these approaches require the use of stoichiometric oxidants (e.g., K_2_S_2_O_8_ or hypervalent iodine reagents) and/or precious metal (photo)catalysts. As such, the development of alternative, more sustainable, approaches for the generation of alkoxy radicals from commodity aliphatic alcohols is an important and timely pursuit.

**Scheme 1:**
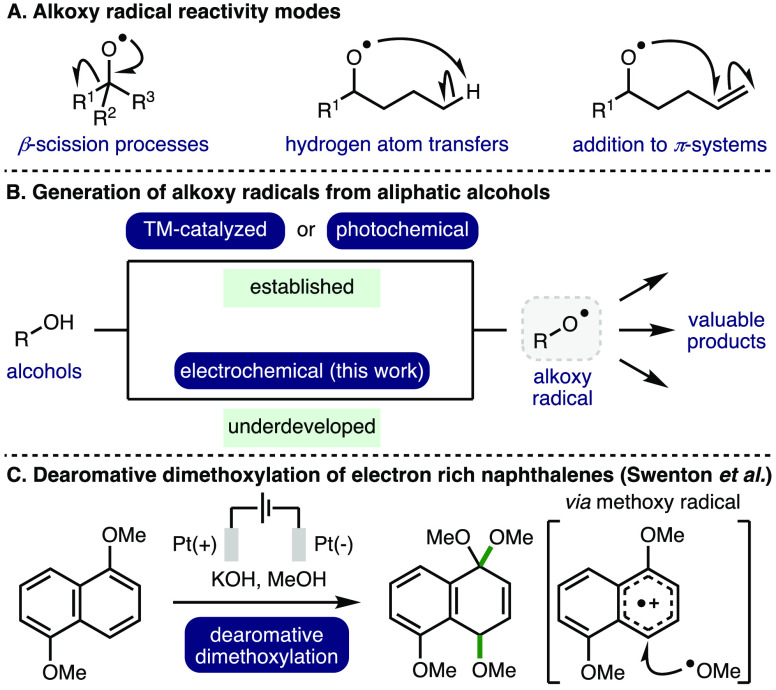
Context

The development of electrochemical methods^10^ for the generation of alkoxy radicals has received surprisingly little attention to date and remains significantly underdeveloped. In 1981, Swenton and co-workers reported the dearomative dimethoxylation of electron rich naphthalenes (Scheme 1C).^11^ The same approach was subsequently employed for the dimethoxylation of 4-methoxyanilines^12^ and dimethoxybenzenes.^13^ The authors proposed that the mechanism for these transformations involves the formation of methoxy radicals via direct anodic oxidation of methoxide anions and evidence to support this was provided through radical trapping experiments and electron spin resonance (ESR).^13a^ It was found that the broad potential window of boron-doped diamond or platinum anodes was essential for generating a sufficient concentration of methoxy radicals due to the high oxidation potential required for direct anodic oxidation of methoxide anions,^14,15^ which inherently limits the broader application of this approach in synthesis.^16,17^

Cognizant of the opportunities to make advances in this underdeveloped domain, our research team reported the manganese-catalyzed electrochemical deconstructive chlorination of tertiary cyclopropanols and cyclobutanols.^18^ However, the method could not be more broadly applied toward larger and more widely commercially available ring sizes or alternative functionalizations.^19^ Proton-coupled electron transfer (PCET),^20^ which involves the concerted movement of a proton and an electron in a single elementary step, can be used to overcome the energetic limitations of classical HAT reagents for the formation of alkoxy radicals. For example, spectroscopic studies by Baciocchi and co-workers described the β-scission of 1-arylalkanol radical cations,^21^ and this strategy has been applied toward the development of various photochemical transformations.^22^ To develop a more general electrochemical method to access and utilize alkoxy radicals, we envisaged an electrochemically driven PCET approach,^23^ and herein we report the successful realization of this strategy. During the latter stages of this investigation, Onomura and co-workers reported an electrophotochemical deconstructive bromination of cycloalkanols, which involves the electrochemical generation of hypobromite intermediates via anodic bromide oxidation followed by visible light-promoted homolysis of the weak O–Br bond to generate an alkoxy radical.^24^

To commence our studies, the deconstructive bromination of 1-(4-methoxyphenyl)cyclohexan-1-ol **1** to form ε-bromo ketone **2** was selected for reaction optimization (Table 1).^25^ The proposed electrochemical generation of alkoxy radicals by PCET was contingent upon efficient electrochemical oxidation of the 4-methyoxyphenyl ring present within **1** (*E*_p/2_ = 1.03 V vs Fc/Fc^+^). Employing BrCCl_3_ as the brominating agent, collidine as the Brønsted base, and LiClO_4_ as the supporting electrolyte in MeCN/TFE (12:1, [**1**] = 0.05 M) using galvanostatic conditions (*i* = 10 mA, *j*_anode_ = 7.1 mA/cm^2^, *Q* = 4.5 F/mol), a graphite anode and a platinum foil cathode in an undivided cell at 25 °C under N_2_, gave **2** in an encouraging 32% NMR yield (entry 1), with 68% recovered **1**. To improve conversion, we investigated the impact of employing a range of triarylamine^26^ and triarylimidazole^27^ redox mediators,^25^ and it was found that the addition of N(4-NO_2_C_6_H_4_)_2_Ph (5 mol %) (*E*_ox_ = 0.91 V vs Fc/Fc^+^) gave quantitative conversion to **2** with a 94% isolated yield (entry 2).^28^ The increase in conversion using a triarylamine mediator may be attributed to more efficient electron transfer and productive intermolecular reactivity through an electrochemical–chemical (EC′) mechanism.^29^ The use of a redox mediator also lowers the required applied potential below the oxidation potential of the substrate, and these milder electrochemical conditions can minimize side reactions, while improving functional group tolerance. No product formation was observed in the absence of electricity or in the absence of collidine (entries 3 and 4). Lowering the loading of collidine, BrCCl_3_, or redox mediator all reduced the NMR yield of **2** (entries 5–7), as did variation of cathode material, supporting electrolyte, and solvent (entries 8–11). Reducing the current and charge passed both had a detrimental impact upon product formation (entries 12 and 13).

**Table 1:**
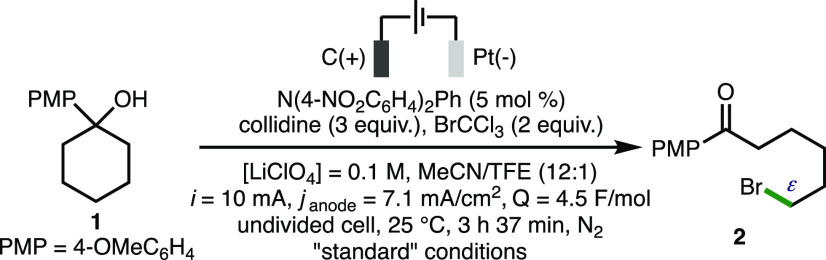
Optimization of the Electrochemical Process^a^

entry	variation from “standard” conditions	yield^b^ (%)
1	no N(4-NO_2_C_6_H_4_)_2_Ph	32
**2**	**none**	**>98 (94)**
3	no electricity	<2
4	no collidine	<2
5	collidine (2 equiv)	81
6	BrCCl_3_ (1.1 equiv)	49
7	N(4-NO_2_C_6_H_4_)_2_Ph (1 mol %)	50
8	Ni plate cathode	63
9	[TBAPF_6_] = 0.1 M as supporting electrolyte	80
10	MeCN/HFIP (12:1) as solvent	49
11	CH_2_Cl_2_/TFE (12:1) as solvent	74
12	*i* = 7.5 mA, *j*_anode_ = 5.3 mA/cm^2^	74
13^c^	*Q* = 2.5 F/mol	56

a Reactions performed with 0.3 mmol of **1** using the ElectraSyn 2.0 batch electrochemical reactor.

b As determined by ^1^H NMR analysis of the crude reaction mixture with 1,3,5-trimethoxybenzene as the internal standard. Isolated yield given in parentheses.

c 121 min reaction time.

With optimized reaction conditions in hand, the full scope of the electrochemical process was explored (Scheme 2). Initially, it was found that the deconstructive bromination of 3-, 4-, 5-, 6-, 7-, 8-, and 12-membered cycloalkanols proceeded efficiently to access the corresponding distally brominated ketone products **2**–**8** in high yields, overcoming a key limitation of our previous electrochemical approach, which was restricted to cyclopropanols and cyclobutanols.^18^ The 4-methoxyphenyl ring can be replaced with a variety of alternative electron-rich and/or extended aromatic systems to give brominated ketone products **9**–**16** in good isolated yields. No conversion to product **13** was observed employing the previously optimized reaction conditions (Table 1, entry 2), which may be attributed to the higher oxidation potential of the cycloalkanol substrate (*E*_p/2_ = 1.28 V vs Fc/Fc^+^). However, it was found that employing N(4-Cl,2-NO_2_C_6_H_3_)_3_ (10 mol %) (*E*_ox_ = 1.50 V vs Fc/Fc^+^) as the redox mediator enabled access to product **13**, which was isolated in 48% yield. Employing 1-phenylcyclohexan-1-ol as substrate (*E*_p/2_ = 1.60 V vs Fc/Fc^+^), resulted in quantitative recovery of starting material under the same reaction conditions. A substituted adamantane scaffold was converted to bicyclo[3.3.1] product **17** as a single diastereoisomer in 84% isolated yield. An unsymmetrical substituted *trans*-decalin substrate underwent regioselective deconstructive bromination to access 1,2-disubstituted cyclohexane product **18** as a 1:1 mixture of diastereoisomers in 70% isolated yield. Due to the reversibility of β-scission processes involving alkoxy radicals,^1^ the regioselectivity employing unsymmetrical cycloalkanols proceeds via selective cleavage of the more substituted β-C–C bond that produces the more stabilized *C*-centered radical.^30^ Indeed, where relevant, only a single product regioisomer was observed for all substrates. A range of substituted cyclohexanols, piperidines, and an azepane were found to be compatible with the electrochemical method, providing access to the corresponding distally brominated ketones **19**–**26** in high yields. The high regioselectivity observed in the formation of products **20** and **26** was attributed to subtle inductive effects resulting in differences in the relative stability of radical intermediates formed upon β-C–C homolysis. Employing an acyclic substrate resulted in the formation of ketone **27** in 85% isolated yield. Deconstructive chlorination and iodination were demonstrated by employing CCl_4_ and *N*-iodosuccinimide as electrophilic polarized SOMOphiles to access products **28** and **29**, respectively. Finally, in the absence of BrCCl_3_ and performing the reaction under air without degassing the reaction mixture prior to electrolysis, keto-aldehyde **30** was isolated in 70% yield.

**Scheme 2:**
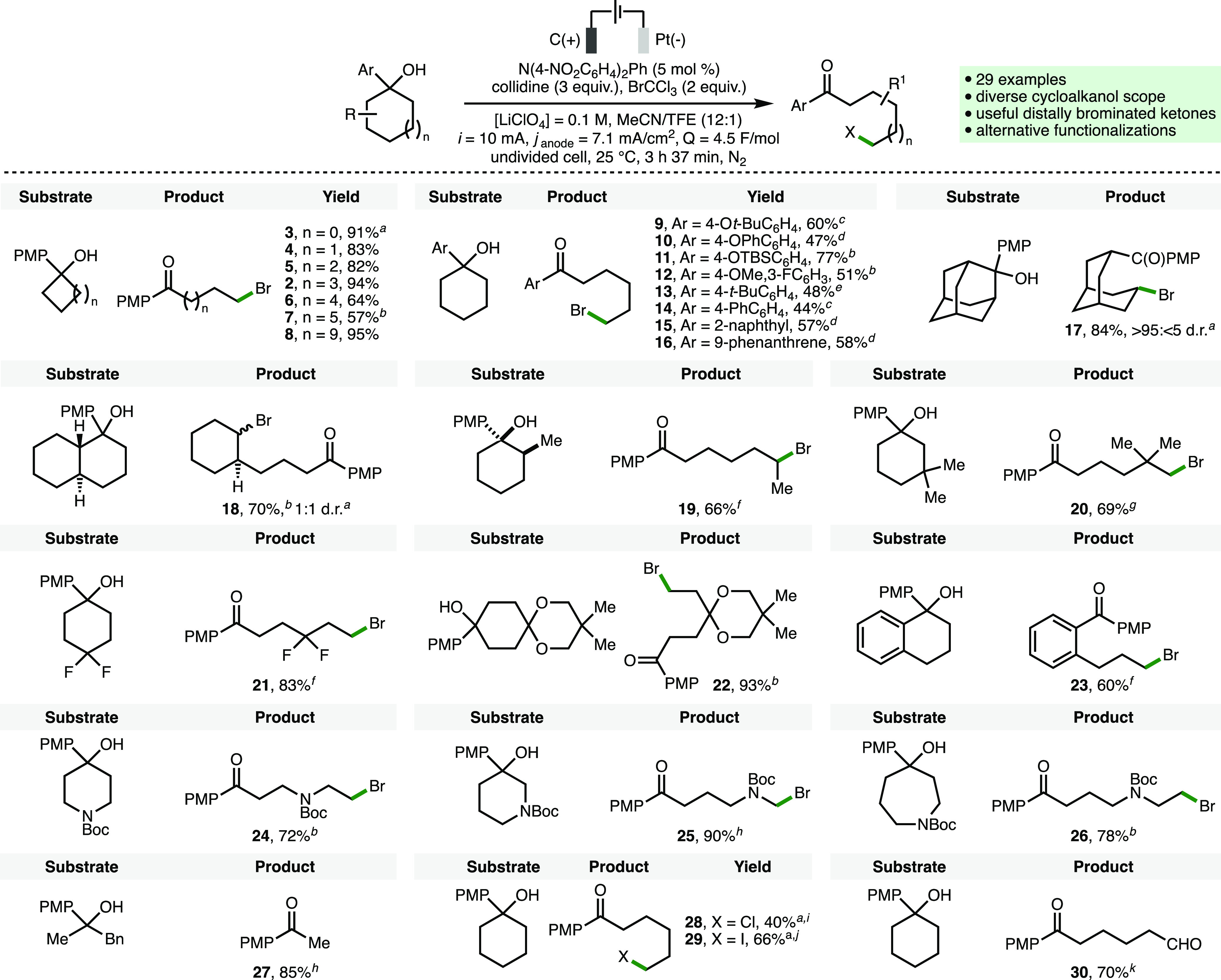
Substrate Scope Reactions performed with 0.3 mmol of cycloalkanol using the ElectraSyn 2.0 batch electrochemical reactor with isolated yields after chromatographic purification quoted unless stated otherwise. As determined by ^1^H NMR analysis of the crude reaction mixture with 1,3,5-trimethoxybenzene as the internal standard. *Q* = 6 F/mol. N(4-NO_2_C_6_H_4_)_2_Ph (10 mol %), *Q* = 9 F/mol. *Q* = 12.4 F/mol. N(4-Cl,2-NO_2_C_6_H_3_)_3_ (10 mol %), *i* = 20 mA, *Q* = 9 F/mol. *i* = 12.5 mA, *Q* = 9 F/mol. N(4-Cl,2-NO_2_C_6_H_3_)_3_ (5 mol %). *Q* = 9 F/mol. CCl_4_ (2 equiv). *N*-Iodosuccinimide (5 equiv). Without N_2_ atmosphere and in the absence of BrCCl_3_.

To demonstrate product utility, the orthogonal functionalization of ε-bromo ketone **2** was investigated (Scheme 3A). It was found that the C(sp^3^)–Br functionality could serve as a coupling partner in a nickel-catalyzed ball-milling enabled cross-electrophile coupling to access arylated ketone **31** in 78% isolated yield.^31^ Furthermore, the 4-methoxyphenyl ketone functionality within **2** was converted to the corresponding ester via Baeyer–Villiger oxidation to give **32** in 84% isolated yield. By employing a HPLC pump in combination with commercially available Ammonite8 flow electroreactor (volume = 1 mL, *i* = 840 mA),^32^ 10 mmol of 1-(4-methoxyphenyl)cyclohexan-1-ol **1** was converted to ε-bromo ketone **2** in 83% isolated yield (2.30 g) in a continuous single-pass. In comparison to batch, the flow process exhibits higher productivity (9.4 mmol/h vs 0.08 mmol/h), requires less charge for full consumption of **1** (*Q* = 2.9 F/mol vs *Q* = 4.5 F/mol), and employs an increased current density (*j*_anode_ = 42 mA/cm^2^ vs *j*_anode_ = 7.1 mA/cm^2^).

**Scheme 3:**
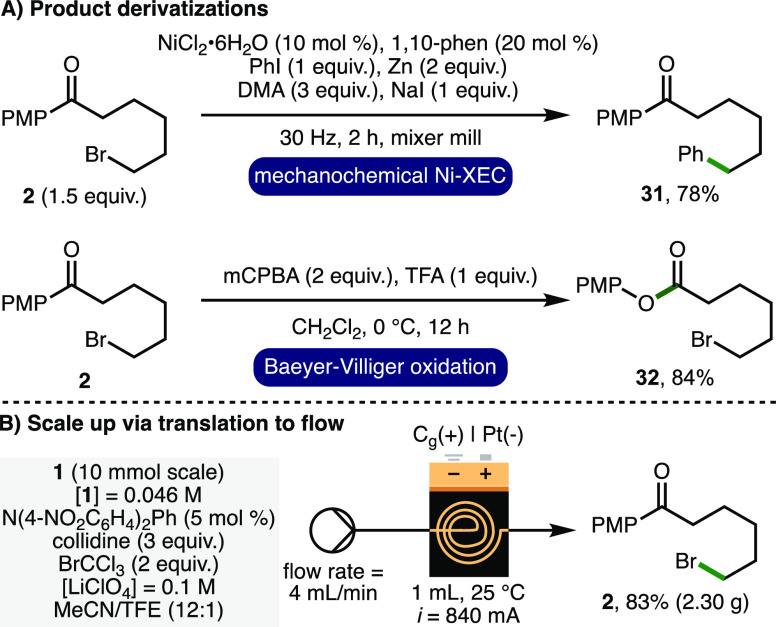
Product Utility and Reaction Scale Up in Flow

Using cyclic voltammetry, it was found that N(4-NO_2_C_6_H_4_)_2_Ph undergoes irreversible oxidation at 0.91 V vs Fc/Fc^+^,^25^ which is attributed to the generation of the corresponding triarylamine radical cation. An increase in the oxidation current was observed upon addition of 1-(4-methoxyphenyl)cyclohexan-1-ol **1**, which suggested that the triarylamine radical cation is consumed by **1** to reform N(4-NO_2_C_6_H_4_)_2_Ph and provided support for the proposed role of the triarylamine acting as a redox mediator as part of an electrochemical-chemical (EC′) mechanism.^29^ When methyl ether cyclohexane **33** was subjected to the standard electrochemical reaction conditions, no ketone **2** was observed, with 88% starting material recovered (Scheme 4A), which indicated that C–C bond cleavage does not proceed in the absence of a hydroxyl functional group. Deshielding of the hydroxyl proton shift within **1** upon the addition of collidine in CD_3_CN was observed, which indicated the presence of a hydrogen bond adduct between **1** and the base^25^ and provided support for a subsequent concerted multisite PCET.^33^ Analysis of the reaction mixture produced using optimized conditions (Table 1, entry 2) by ^1^H NMR in CD_3_CN revealed that the majority of the trichloromethyl radical is converted to CHCl_3_ (66% NMR yield),^25^ which may occur via hydrogen atom transfer or via cathodic reduction followed by protonation of the trichloromethyl anion. As such, a plausible reaction mechanism initiates with anodic oxidation of N(4-NO_2_C_6_H_4_)_2_Ph (*E*_ox_ = 0.91 V vs Fc/Fc^+^) to form a triarylamine radical cation (Scheme 4B), which subsequently oxidizes the electron-rich aromatic ring within **1** (*E*_p/2_ = 1.03 V vs Fc/Fc^+^). A concerted PCET involving alcohol deprotonation by collidine and oxidation by the internal aryl radical cation forms the alkoxy radical. Subsequent β-scission generates a distal alkyl radical, which is trapped with BrCCl_3_ to form a new C–Br bond. The counter cathodic reaction is hydrogen gas production via proton reduction and/or chloroform production via reduction of trichloromethyl radicals and subsequent protonation.

**Scheme 4:**
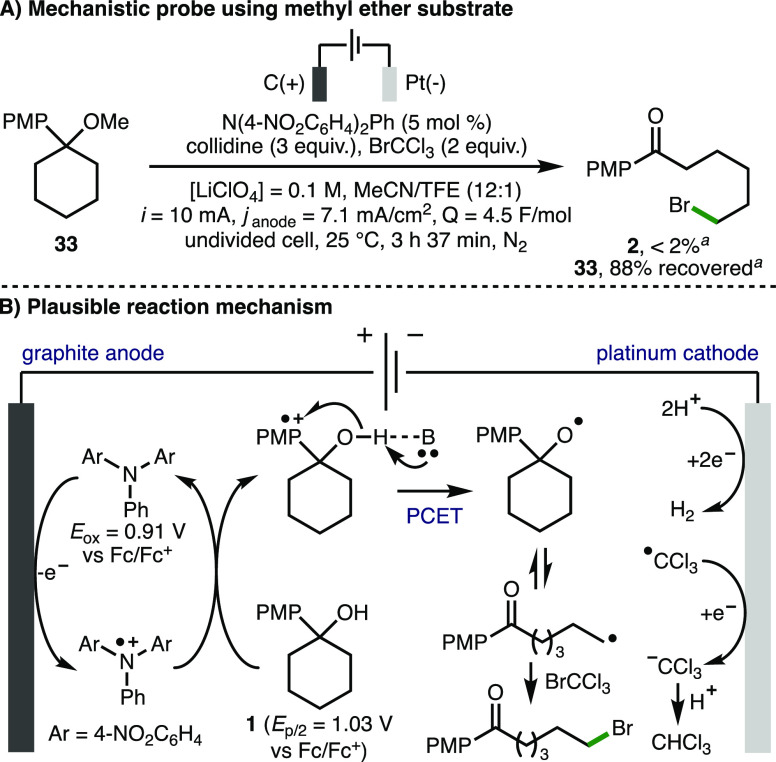
Mechanistic Studies

## Supporting Information Available

Optimization data, experimental procedures, characterization of new compounds, and spectral data (PDF)

## Notes

Data Access: The data that support the findings of this study are openly available in the Cardiff University data catalogue at http://doi.org/10.17035/d.2022.0197737325.
