# Supplementary material for: Electrochemical Deconstructive Functionalization of Cycloalkanols via Alkoxy Radicals Enabled by Proton-Coupled Electron Transfer
Source: Org Lett. 2022 May 23;24(21):3890–5. doi: 10.1021/acs.orglett.2c01552 (PMC9171832; doi:10.1021/acs.orglett.2c01552)
Supplement: Supplementary file 1 — ol2c01552_si_001.pdf [file ol2c01552_si_001.pdf]

SUPPORTING INFORMATION

Electrochemical Deconstructive Functionalization of Cycloalkanols *via* Alkoxy Radicals Enabled by Proton-Coupled Electron Transfer

Mishra Deepak Hareram,† Albara A. M. A. El Gehani,† James Harnedy,† Alex C. Seastram,† Andrew C. Jones,† Matthew Burns,‡ Thomas Wirth,§ Duncan L. Browne,¢ and Louis C. Morrill*,†

†Cardiff Catalysis Institute, School of Chemistry, Cardiff University, Main Building, Park Place, Cardiff, CF10 3AT, U.K.
‡Chemical Development, Pharmaceutical Technology & Development, Operations, AstraZeneca, Macclesfield, SK10 2NA, U.K.
§School of Chemistry, Cardiff University, Main Building, Park Place, Cardiff, CF10 3AT, U.K.
¢Department of Pharmaceutical and Biological Chemistry, University College London, School of Pharmacy, London, W1CN 1AX, U.K

*E-mail: MorrillLC@cardiff.ac.uk

Table of Contents

1. General Information 1

2. Experimental and Characterization Data 3

2.1. General Procedures – Substrate Synthesis 3

2.1.1. General Procedure A – Generation and Addition of Grignard Reagents to Cycloalkanones 3

2.1.2. General Procedure B – Generation and Addition of Organolithium Reagents to Cycloalkanones 3

2.2. Characterization of Substrates 4

2.3. Synthesis of Redox Mediators 29

3. Cyclic Voltammetry Data 32

4. Optimization Studies 42

4.1. General Procedure – Electrochemical Deconstructive Functionalization 44

4.2. Characterization of Products 45

5. A. Product Derivatizations 106

B. Experimental for Flow Electrochemical Scale Up 111

6. Mechanistic Studies 116

7. References 125

1. General Information

Unless otherwise stated, all non-electrochemical reactions were conducted in flame-dried glassware under an atmosphere of dry nitrogen or argon, sealed with septum seals and were stirred with Teflon-coated magnetic stirrer bars. Unless stated otherwise, all electrochemical reactions were performed using oven-dried 10 mL ElectraSyn vials under an atmosphere of dry nitrogen, sealed with an ElectraSyn Teflon cap fitted with a graphite anode and platinum cathode, and were stirred with Teflon-coated magnetic stirrer bars. Dry tetrahydrofuran (THF), diethyl ether (Et_2O) and acetonitrile (MeCN) were obtained after passing these previously degassed solvents through activated alumina columns (Mbraun, SPS-800). Tetra-*n*-butylammonium hexafluorophosphate ($TBAPF_6$) was recrystallised from ethanol or water, respectively, and dried in the oven before use. All other solvents and commercial reagents were used as supplied without further purification unless stated otherwise.

"Petrol" refers to the fraction boiling in the range of 40-60 °C unless otherwise stated.

All electrochemical reactions were conducted using an ElectraSyn 2.0 apparatus, purchased from IKA. Graphite, Platinum (Pt) and Nickel (Ni) electrodes were purchased from IKA and are of uniform dimensions. Graphite electrodes were used as supplied from IKA or were cut from a sheet of carbon foil (2 mm thickness) purchased from Goodfellow. The electrodes were cut to the dimension of 8 mm × 52 mm using a Startrite Bandsaw (model 18-T-5), with a Starrett, Durate SFB High Carbon Steel Blade, 2870mm x 10mm x 0.65mm, 3mm pitch, regular tooth. Graphite electrodes could be used several times by renewing the top surface of the graphite. This was achieved by scraping away the top layer with a razor blade, sonicating in MeCN for 5 minutes, followed by oven-drying for 30 mins. Platinum electrodes were used as supplied from IKA or cut from a sheet of platinum foil (0.05 mm thickness) purchased from Goodfellow. The electrodes were cut to the required fitting with a pair of scissors. Platinum electrodes were washed with water and acetone and burned over Bunsen burner before every reaction.

Cyclic voltammetry (CV) experiments were conducted using an Autolab PGSTAT204, controlled using Nova 2.1 software. The working electrode was a GC disc (3 mm dia., BASi part number MF-2012), the counter electrode was a Pt-wire (BASi part number

MW-4130) and a $Ag/AgNO_3$ reference electrode was used (BASi part number – MF-2052).

Room temperature (rt) refers to 20-25 °C. Ice/water and CO_2(s)/acetone baths were used to obtain temperatures of 0 °C and -78 °C respectively. All reactions involving heating were conducted using DrySyn blocks and a contact thermometer. *In vacuo* refers to reduced pressure through the use of a rotary evaporator.

Analytical thin layer chromatography was carried out using aluminium plates coated with silica (Kieselgel 60 F_{254} silica) and visualisation was achieved using ultraviolet light (254 nm), followed by staining with a 1% aqueous $KMnO_4$ solution, or a 10% w/v solution of phosphomolybdic acid in ethanol. 2,4-DNP was also used as a TLC stain. Flash column chromatography was performed using Kieselgel 60 silica in the solvent system stated using head-pressure by means of a compressed air line.

Melting points were recorded on an a Gallenkamp melting point apparatus and are reported corrected by linear calibration to benzophenone (47 - 49 °C) and benzoic acid (121 - 123 °C).

Infrared spectra were recorded on a Shimadzu IRAffinity-1 Fourier Transform ATR spectrometer as thin films using a Pike MIRacle ATR accessory. The most intense peaks and structurally important peaks are quoted. Absorption maxima (ν_{max}) are recorded in wavenumbers (cm^{-1}).

^{1}H, ^{13}C and ^{19}F NMR spectra were obtained on a Bruker Avance 300 (300 MHz ^{1}H, 75 MHz ^{13}C), Bruker Avance 400 (400 MHz ^{1}H, 101 MHz ^{13}C, 376 MHz ^{19}F) or a Bruker Avance 500 (500 MHz ^{1}H, 126 MHz ^{13}C, 471 MHz ^{19}F) spectrometer at rt in the solvent stated. Chemical shifts are reported in parts per million (ppm) relative to the residual solvent signal. All coupling constants, *J*, are quoted in Hz. Multiplicites are reported with the following symbols: br = broad, s = singlet, d = doublet, t = triplet, q = quartet, m = multiplet and combinations of these were used to denote higher order multiplicities. App = apparent.

High resolution mass spectrometry (HRMS, *m/z*) data was acquired at Cardiff University on a Micromass LCT Spectrometer.

2. Experimental and Characterization Data

2.1. General Procedures – Substrate Synthesis

2.1.1. General Procedure A – Generation and Addition of Grignard Reagents to Cycloalkanones

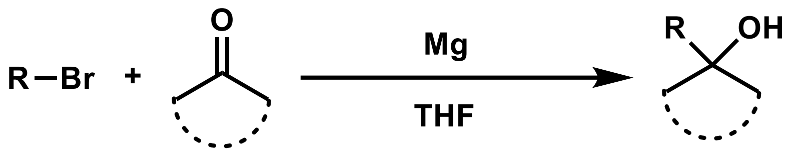

To a three-necked RBF fitted with a condenser, a glass stopper, and a septum seal was added magnesium turnings (1.65 equiv.) and the system was flame-dried under a high vacuum. After cooling under a N_2 atmosphere, a crystal of iodine was added followed by THF (1 M with respect to RBr). RBr (1.5 equiv.) was added dropwise, and the mixture was stirred at rt for 5 min, or until the exotherm had finished. The reaction mixture was then heated at reflux for 1-3 h. Upon cooling to 0 °C (ice bath), THF was added (0.2 M with respect to cycloalkanone), followed by drop-wise addition of the cycloalkanone (1.0 equiv.). The resulting mixture was left to warm to rt and stirred overnight. A saturated solution of NH_4Cl (aq.) was added and the mixture was diluted with EtOAc. The layers were separated, and the aqueous layer was further extracted with EtOAc (× 2). The combined organics were dried ($MgSO_4$), filtered, and concentrated under reduced pressure. The resultant crude material was purified by flash column chromatography to afford the desired compound.

2.1.2. General Procedure B – Generation and Addition of Organolithium Reagents to Cycloalkanones

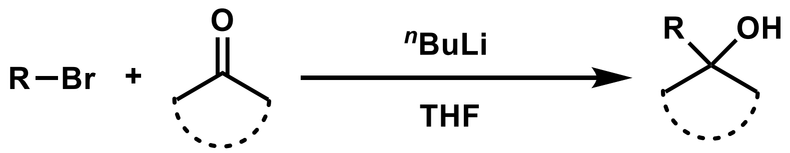

To a flame-dried RBF fitted with a septum seal was added a solution of alkyl or aryl halide (1.3 equiv.) in THF (0.3 M). The mixture was cooled to -78 °C (dry ice - acetone),

and a solution of *n*-butyllithium (solution in hexanes, 1.3 equiv.) was added dropwise. The solution was left to stir for 1-3 h before adding a solution of the cycloalkanone (1.0 equiv.) in THF (1.0 M) dropwise. The reaction mixture was left to stir for 1-3 h, whilst warming to rt. Water was added dropwise, followed by extraction with EtOAc (× 3). The combined organics were dried ($MgSO_4$), filtered, and concentrated under reduced pressure. The resultant crude material was purified by flash column chromatography to afford the desired compound.

2.2. Characterization of Substrates

1-(4-methoxyphenyl)cyclohexan-1-ol (1)

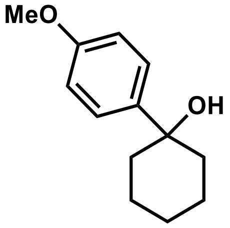

Prepared according to General Procedure B using 1-bromo-4-methoxybenzene (3.75 g, 20.0 mmol, 1.0 equiv.), *n*-butyllithium (12.0 mL, 2.5 M in hexanes, 30.0 mmol, 1.5 equiv.) and cyclohexanone (2.30 mL, 22.0 mmol, 1.1 equiv.). The crude residue was purified by flash column chromatography (5→10% EtOAc/Petrol, silica gel) to afford **1** (3.45 g, 54%) as a white solid.

R_f = 0.15 (10% EtOAc/Petrol); **M.p.** = 37-39 °C (lit.[1] 38-40 °C); **^{1}H NMR (500 MHz, $CDCl_3$)** δ = 7.43 (d, *J* = 8.8 Hz, 2H), 6.88 (d, *J* = 8.9 Hz, 2H), 3.81 (s, 3H), 1.86 – 1.70 (m, 7H), 1.64 – 1.62 (m, 2H), 1.54 (s, 1H) 1.31 – 1.25 (m, 1H); **^{13}C NMR (126 MHz, $CDCl_3$)** δ = 158.4, 141.7, 125.9, 113.6, 72.8, 55.4, 39.1, 25.6, 22.4

These data are consistent with those previously reported in the literature.[1]

1-(4-methoxyphenyl)cyclopropan-1-ol (S3)

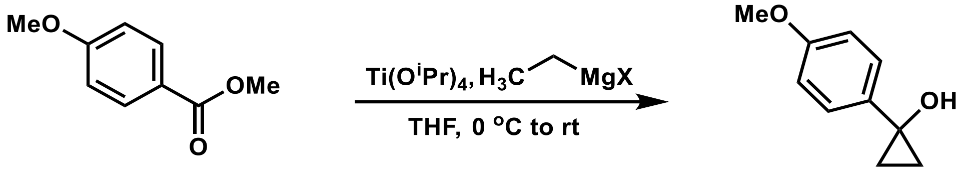

To a flame-dried RBF fitted with a septum seal was added methyl 4-methoxybenzoate (0.83 g, 5.0 mmol, 1.0 equiv.), Ti(O^{i}Pr)$_4$ (2.13 mL, 7.0 mmol, 1.4 equiv.), and THF (0.16 M with respect to the ester). The mixture was cooled to 0 °C (ice bath) and a solution of ethylmagnesium bromide (4.65 mL, 2.5 M, 14.0 mmol, 2.8 equiv.) was added dropwise. After complete addition, the resulting mixture was left to warm to rt and stirred overnight. After completion water was added and the resulting precipitate was filtered under vacuum through celite and washed with EtOAc. The layers of the filtrate were separated, and the aqueous layer was further extracted with EtOAc (× 2). The combined organics were dried ($MgSO_4$), filtered, and concentrated under reduced pressure. The resultant crude material was purified by flash column chromatography (5→10% EtOAc/Petrol, silica gel) to afford **S3** (560 mg, 68%) as a colourless oil.

R_f = 0.17 (10% EtOAc/Petrol); **^{1}H NMR (500 MHz, $CDCl_3$)** δ = 7.29 – 7.24 (m, 2H), 6.88 – 6.83 (m, 2H), 3.81 (s, 3H), 2.43 (s, 1H), 1.21 – 1.16 (m, 2H), 1.0 – 0.94 (m, 2H); **^{13}C NMR (126 MHz, $CDCl_3$)** δ = 158.4, 136.4, 125.9, 113.6, 56.6, 55.4, 16.6

These data are consistent with those previously reported in the literature.[2]

1-(4-methoxyphenyl)cyclobutan-1-ol (S4)

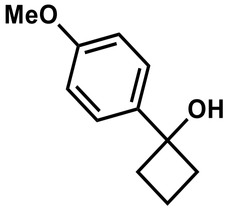

Prepared according to General Procedure B using 1-bromo-4-methoxybenzene (0.62 mL, 5.00 mmol, 1.0 equiv.), *n*-butyllithium (3.16 mL, 2.5 M in hexanes, 6.00 mmol, 1.5 equiv.) and cyclobutanone (0.56 mL, 5.50 mmol, 1.1 equiv.). The crude residue was

purified by flash column chromatography (5→10% EtOAc/Petrol, silica gel) to afford **S4** (0.79 g, 95%) as a colourless liquid.

R$_f$ = 0.12 (10% EtOAc/Petrol); **^{1}H NMR (500 MHz, CDCl$_3$)** δ = 7.43 (d, *J* = 8.9 Hz, 2H), 6.91 (d, *J* = 8.9 Hz, 2H), 3.82 (s, 3H), 2.64 – 2.44 (m, 2H), 2.39 – 2.31 (m, 2H), 2.02 – 1.92 (m, 2H), 1.69 – 1.56 (m, 1H); **^{13}C NMR (126 MHz, CDCl$_3$)** δ = 158.9, 138.6, 126.5, 113.9, 76.8, 55.4, 37.0, 13.0.

These data are consistent with those previously reported in the literature.[2]

1-(4-methoxyphenyl)cyclopentan-1-ol (*S*5)

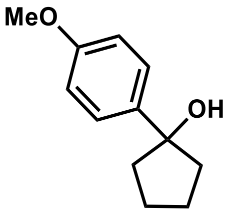

Prepared according to General Procedure B using 1-bromo-4-methoxybenzene (0.62 mL, 5.0 mmol, 1.0 equiv.), *n*-butyllithium (3.16 mL, 2.5 M in hexanes, 6.0 mmol, 1.5 equiv.) and cyclopentanone (0.66 mL, 5.5 mmol, 1.1 equiv.). The crude residue was purified by flash column chromatography (5→10% EtOAc/Petrol, silica gel) to afford **S5** (0.77g, 77%) as a colourless liquid.

R$_f$ = 0.16 (10% EtOAc/Petrol); **^{1}H NMR (300 MHz, CDCl$_3$)** δ = 7.42 (d, *J* = 8.8 Hz, 2H), 6.88 (d, *J* = 8.8 Hz, 2H), 3.81 (s, 3H), 2.12 – 1.86 (m, 6H), 1.89 – 1.75 (m, 2H), 1.51 (s, 1H).; **^{13}C NMR (75 MHz, CDCl$_3$)** δ = 158.6, 139.3, 126.5, 113.7, 83.3, 55.4, 41.7, 23.8.

These data are consistent with those previously reported in the literature.[3]

1-(4-methoxyphenyl)cycloheptan-1-ol (S6)

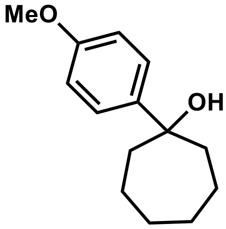

Prepared according to General Procedure B using 1-bromo-4-methoxybenzene (0.62 mL, 5.0 mmol, 1.0 equiv.), *n*-butyllithium (3.16 mL, 2.5 M in hexanes, 6.0 mmol, 1.5 equiv.) and cycloheptanone (0.7 mL, 5.5 mmol, 1.1 equiv.). The crude residue was purified by flash column chromatography (5→10% EtOAc/Petrol, silica gel) to afford **S6** (0.98 g, 70%) as a colourless liquid.

R_f = 0.19 (10% EtOAc/Petrol); **^{1}H NMR (300 MHz, $CDCl_3$)** δ = 7.42 (d, *J* = 8.9 Hz, 2H), 6.87 (d, *J* = 8.9 Hz, 2H), 3.80 (s, 3H), 2.16 – 1.66 (m, 8H), 1.55 – 1.50 (m, 4H), 0.99 – 0.82 (m, 1H); **^{13}C NMR (75 MHz, $CDCl_3$)** δ = 158.3, 142.9, 125.9, 113.5, 55.4, 43.4, 29.3, 24.0, 22.6.

These data are consistent with those previously reported in the literature.[3]

1-(4-methoxyphenyl)cyclooctan-1-ol (S7)

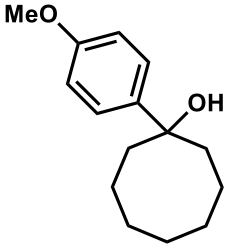

Prepared according to General Procedure B using 1-bromo-4-methoxybenzene (0.62 mL, 5.00 mmol, 1.0 equiv.), *n*-butyllithium (3.16 mL, 2.5 M in hexanes, 6.0 mmol, 1.5 equiv.) and cyclooctanone (0.70 mL, 5.50 mmol, 1.1 equiv.). The crude residue was purified by flash column chromatography (5→10% EtOAc/Petrol, silica gel) to afford **S7** (1.20 g, 88%) as a colourless liquid.

R_f = 0.21 (10% EtOAc/Petrol); **^{1}H NMR (300 MHz, $CDCl_3$)** δ = 7.44 (d, *J* = 8.9 Hz, 2H), 6.87 (d, *J* = 8.8 Hz, 2H), 3.81 (s, 3H), 2.03 – 1.96 (m, 5H), 1.73 – 1.70 (m, 5H), 1.52 – 1.50

(m, 4H), 1.05 – 0.83 (m, 1H); **^{13}C NMR (75 MHz, CDCl$_3$)** δ = 158.4, 141.3, 126.4, 113.5, 76.5, 55.4, 37.8, 28.5, 24.6, 22.2.

These data are consistent with those previously reported in the literature.[3]

1-(4-methoxyphenyl)cyclododecan-1-ol (S8)

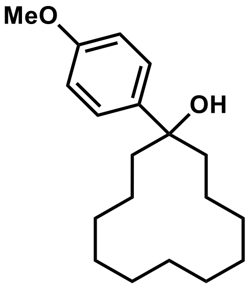

Prepared according to General Procedure B using 1-bromo-4-methoxybenzene (0.62 mL, 5.0 mmol, 1.0 equiv.), *n*-butyllithium (3.16 mL, 2.5 M in hexanes, 6.0 mmol, 1.5 equiv.) and cyclododecanone (1.30 g, 5.5 mmol, 1.1 equiv.). The crude residue was purified by flash column chromatography (5→10% EtOAc/Petrol, silica gel) to afford **S8** (0.70 g, 45%) as a colourless liquid.

R$_f$ = 0.21 (10% EtOAc/Petrol); **^{1}H NMR (500 MHz, CDCl$_3$)** δ = 7.40 (d, *J* = 8.9 Hz, 2H), 6.86 (d, *J* = 8.8 Hz, 2H), 3.80 (s, 3H), 1.85 (t, *J* = 7.5 Hz, 4H), 1.55 (s, 1H) 1.46 – 1.16 (m, 18H); **^{13}C NMR (126 MHz, CDCl$_3$)** δ = 158.4, 140.7, 126.5, 113.3, 76.3, 55.4, 35.6, 26.5, 26.2, 22.6, 22.3, 20.1.

These data are consistent with those previously reported in the literature.[3]

1-(4-(tert-butoxy)phenyl)cyclohexan-1-ol (S9)

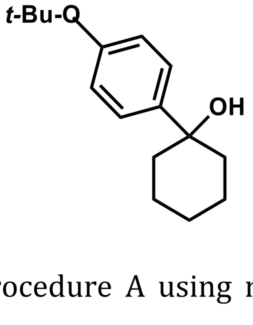

Prepared according to General Procedure A using magnesium turnings (118 mg, 4.8 mmol, 1.1 equiv.), 1-bromo-4-(tert-butoxy)benzene (1.0 g, 4.4 mmol, 1 equiv.)), and

cyclohexanone (0.46 mL, 4.4 mmol, 1 equiv.). The crude residue was purified by flash column chromatography (5→10% EtOAc/Petrol, silica gel) to afford **S9** (533 mg, 74%) as a white solid.

R_f = 0.20 (10% EtOAc/Petrol); **M.p.** = 88-90 °C; **^{1}H NMR (500 MHz, $CDCl_3$)** δ = 7.41 – 7.35 (m, 2H), 6.96 – 6.93 (m, 2H), 1.88 – 1.68 (m, 8H), 1.63 (m, 2H) , 1.55 (s, 1H), 1.54, 1.34 (s, 9H); **^{13}C NMR (126 MHz, $CDCl_3$)** δ = 154.2, 144.3, 125.3, 123.8, 78.4, 73.0, 39.0, 29.0, 25.7, 22.4.

These data are consistent with those previously reported in the literature.[4]

1-(4-phenoxyphenyl)cyclohexan-1-ol (S10)

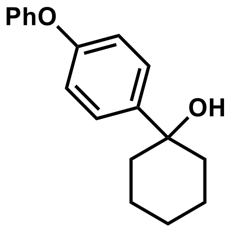

Prepared according to General Procedure A using 1-bromo-4-phenoxybenzene (1.62 g, 6.50 mmol, 1 equiv.) *n*-butyllithium (2.60 mL, 2.5 M in hexanes, 6.50 mmol, 1.3 equiv.), and cyclohexanone (491 mg, 518 μL, 5.00 mmol, equiv.). The crude residue was purified by flash column chromatography (6→10% EtOAc/Hexanes, silica gel) to afford **S10** (768 mg, 57%) as a colourless oil.

R_f = 0.10 (10% EtOAc/Petrol); **^{1}H NMR (500 MHz, $CDCl_3$)** δ = 7.52 – 7.43 (m, 2H), 7.38 – 7.30 (m, 2H), 7.10 (m, 1H), 7.05 – 6.93 (m, 4H), 1.93 – 1.51 (m, 10H), 1.37 – 1.23 (m, 1H); **^{13}C NMR (126 MHz, $CDCl_3$)** δ = 157.4, 156.0, 144.5, 129.9, 126.2, 123.3, 119.0, 118.6, 73.0, 39.1, 25.6, 22.4.

These data are consistent with those previously reported in the literature.[5]

1-(4-((tert-butyldimethylsilyl)oxy)phenyl)cyclohexan-1-ol (S11)

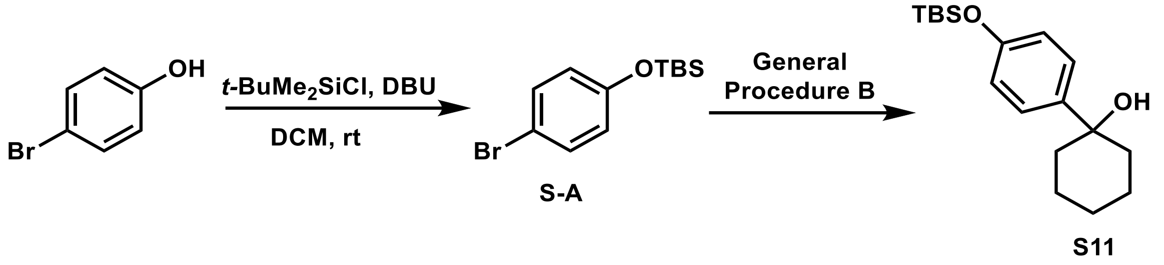

Prepared using 4-bromophenol (1.0 g, 5.78 mmol, 1 equiv.) and *tert*-butyldimethylsilyl chloride (1.74 g, 11.6 mmol, 2.0 equiv.) dissolved in DCM (30 mL). To this solution was added 1,8-diazabicyclo[5.4.0]undec-7-ene (DBU) (2.27 mL, 15 mmol, 2.5 equiv.) dropwise and the reaction mixture was stirred for 2 h at room temperature. After completion by TLC, the reaction was quenched by aq. $NaHCO_3$ and extracted with DCM (25 mL x2), dried ($MgSO_4$) and concentrated under reduced pressure to afford **S-A** (1.68 g, quant.) as a colourless oil that was used without further purification.

R_f = 0.91 (10% EtOAc/Petrol); **^{1}H NMR (400 MHz, $CDCl_3$)** δ = 7.35 – 7.26 (m, 2H), 6.80 – 6.67 (m, 2H), 0.97 (s, 9H), 0.18 (s, 6H); **^{13}C NMR (101 MHz, $CDCl_3$)** δ = 155.0, 132.4, 122.1, 113.7, 25.8, 18.3, -4.32

These data are consistent with those previously reported in the literature.[6]

S11 was prepared according to General Procedure B using **S-A** (1.43 g, 5.0 mmol, 1.0 equiv.), *n*-butyllithium (2.4 mL, 2.5 M in hexanes, 6.0 mmol, 1.5 equiv.) and cyclohexanone (0.62 mL, 6 mmol, 1.1 equiv.). The crude residue was purified by flash column chromatography (5→10% EtOAc/Petrol, silica gel) to afford **S11** (0.96 g, 63%) as a colourless oil.

R_f = 0.62 (10% EtOAc/Petrol); **^{1}H NMR (500 MHz, $CDCl_3$)** δ = 7.38 – 7.33 (m, 2H), 6.83 – 6.75 (m, 2H), 1.86 – 1.68 (m, 7H), 1.64 – 1.56 (m, 2H), 1.52 (s, 1H), 1.32 – 1.23 (m, 1H), 0.98 (s, 9H), 0.19 (s, 6H); **^{13}C NMR (125 MHz, $CDCl_3$)** δ = 154.4, 142.2, 126.0, 120.0 72.9, 39.0, 25.8, 25.7, 22.4, 18.3, -4.3.

These data are consistent with those previously reported in the literature.[4]

1-(3-fluoro-4-methoxyphenyl)cyclohexan-1-ol (S12)

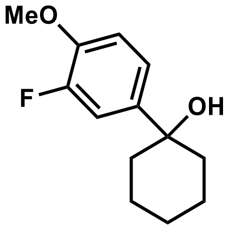

Prepared according to General Procedure B using 4-bromo-2-fluoro-1-methoxybenzene (0.65 mL, 5.0 mmol, 1.0 equiv.), *n*-butyllithium (2.4 mL, 2.5 M in hexanes, 6.0 mmol, 1.5 equiv.) and cyclohexanone (0.62 mL, 6 mmol, 1.1 equiv.). The crude residue was purified by flash column chromatography (5→10% EtOAc/Petrol, silica gel) to afford **S12** (0.70 g, 62%) as a colourless oil.

R_f = 0.21 (10% EtOAc/Petrol); **^{1}H NMR (500 MHz, $CDCl_3$)** δ = 7.23 – 7.17 (m, 1H), 7.15 (ddd, *J* = 8.5, 2.3, 1.2 Hz, 1H), 6.88 (app t, *J* = 8.7 Hz, 1H), 3.84 (s, 3H), 1.76 – 1.65 (m, 9H), 1.61 – 1.58 (m, 2H); **^{13}C NMR (126 MHz, $CDCl_3$)** δ = 152.6 (d, *J* = 244.7 Hz), 146.3 (d, *J* = 10.8 Hz), 143.0 (d, *J* = 4.7 Hz), 120.3 (d, *J* = 3.4 Hz), 113.2 (d, *J* = 6.4 Hz), 113.1 (d, *J* = 10.7 Hz), 72.7(d, *J* = 1.4 Hz), 56.5, 38.9, 25.5, 22.3; **^{19}F (471 MHz, $CDCl_3$)** δ = -135.37

These data are consistent with those previously reported in the literature.[4]

1-(4-(tert-butyl)phenyl)cyclohexan-1-ol (S13)

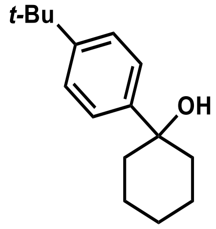

Prepared according to General Procedure B using 1-bromo-4-(tert-butyl)benzene (2.16 g, 10 mmol, 1.0 equiv.), *n*-butyllithium (4.4 mL, 2.5 M in hexanes, 6.0 mmol, 1.5 equiv.) and cyclohexanone (1.24 mL, 11 mmol, 1.1 equiv.). The crude residue was purified by flash column chromatography (5→10% EtOAc/Petrol, silica gel) to afford **S13** (1.4 g, 60%) as a white solid.

R_f = 0.20 (10% EtOAc/Petrol); **M.p.** = 65-67 °C (lit.[7] 66-67); **^{1}H NMR (500 MHz, $CDCl_3$)** δ = 7.50 – 7.31 (m, 4H), 1.90 – 1.60 (m, 10H), 1.55 (s, 1H), 1.33 (s, 9H); **^{13}C NMR (126 MHz, $CDCl_3$)** δ = 149.4, 146.5, 125.2, 124.4, 73.0, 39.0, 34.5, 31.5, 25.7, 22.3.

These data are consistent with those previously reported in the literature.[7]

1-([1,1'-biphenyl]-4-yl)cyclohexan-1-ol (S14)

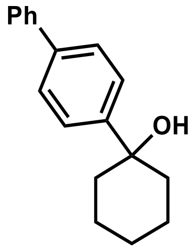

Prepared according to General Procedure B using 4-bromo-1,1'-biphenyl (1.52 g, 6.50 mmol, 1.3 equiv.) n-butyllithium (2.6 mL, 2.5 M in hexanes, 6.50 mmol, 1.3 equiv.), and cyclohexanone (518 µL, 5.00 mmol, 1 equiv.). The crude residue was purified by flash column chromatography (5→10% EtOAc/Hexanes, silica gel) to afford **S14** (907 mg, 75%) as an off white solid.

R_f = 0.19 (10% EtOAc/Petrol); **M.p. =** 74-76 °C; **FTIR** (ν_{max} cm^{-1}, thin film); 3520, 3381 (br), 2852, 1444, 1396, 1122, 1031, 981, 960, 846; **^{1}H NMR (500 MHz, $CDCl_3$)** δ = 7.62 – 7.56 (m, 6H), 7.46 – 7.40 (m, 2H), 7.36 – 7.31 (m, 1H), 1.94 – 1.71 (m, 7H), 1.67 (m, 2H), 1.61 (s, 1H), 1.40 – 1.27 (m, 1H). **^{13}C NMR (126 MHz, $CDCl_3$)** δ = 148.6, 141.0, 139.7, 128.9, 127.3, 127.2, 127.1, 125.2, 73.2, 39.0, 25.7, 22.3; **HRMS** (EI$^+$/FTMS) m/z : [M] Calcd for [$C_{18}H_{20}O$] 252.1514; Found 252.1510.

These data are consistent with those previously reported in the literature.[4]

1-(naphthalen-2-yl)cyclohexan-1-ol (S15)

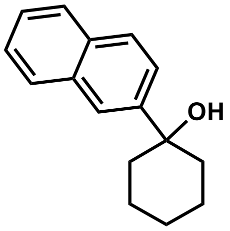

Prepared according to General Procedure B using 2-bromonaphthalene (1.35 g, 6.50 mmol, 1.3 equiv.) n-butyllithium (2.6 mL, 2.5 M in hexanes, 6.50 mmol, 1.3 equiv.), and cyclohexanone (518 µL, 5.00 mmol, 1 equiv.). The crude residue was purified by flash column chromatography (4→10% EtOAc/Hexanes, silica gel) to afford **S15** (450 mg, 40%) as a pink solid.

R_f = 0.20 (10% EtOAc/Petrol); **M.p.** = 63-65 °C; **FTIR** (ν_{max} cm^{-1}, thin film); 3402 (br), 2937, 2926, 2856, 1504, 1444, 1352, 1132, 1120, 966, 887, 850; **^{1}H NMR (500 MHz, $CDCl_3$)** δ = 7.98 – 7.95 (m, 1H), 7.88 – 7.79 (m, 3H), 7.64 (dd, J = 8.7, 1.9 Hz, 1H), 7.51 – 7.42 (m, 2H), 2.03 – 1.93 (m, 2H), 1.91 – 1.75 (m, 5H), 1.75 – 1.62 (m, 3H), 1.54 (s, 1H); **^{13}C NMR (126 MHz, $CDCl_3$)** δ = 146.9, 133.4, 132.5, 128.3, 128.0, 127.6, 126.13 125.8, 123.8, 123.0, 73.5, 38.9, 25.7, 22.4**; HRMS** (EI^+/FTMS) m/z : [M] Calcd for [$C_{16}H_{18}O$] 226.1358; Found 226.1351.

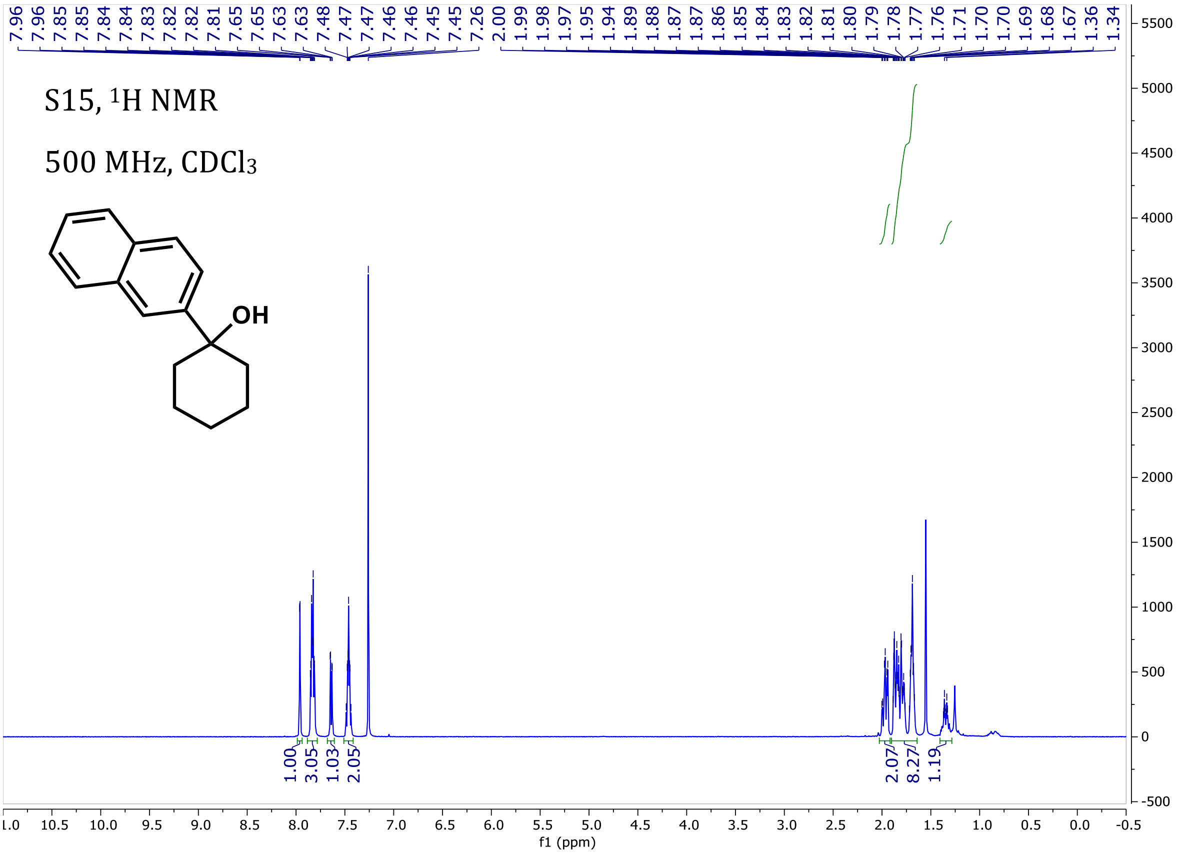

7.96 7.96 7.85 7.85 7.84 7.84 7.83 7.82 7.82 7.81 7.65 7.65 7.63 7.63 7.48 7.47 7.47 7.46 7.45 7.45 7.26 2.00 1.99 1.98 1.97 1.95 1.94 1.89 1.88 1.87 1.87 1.86 1.85 1.84 1.83 1.82 1.81 1.80 1.79 1.78 1.77 1.76 1.71 1.70 1.69 1.68 1.67 1.36 1.34
S15, 1H NMR
500 MHz, CDCl3
OH
1.00 3.05 1.03 2.05 2.07 8.27 1.19
f1 (ppm)

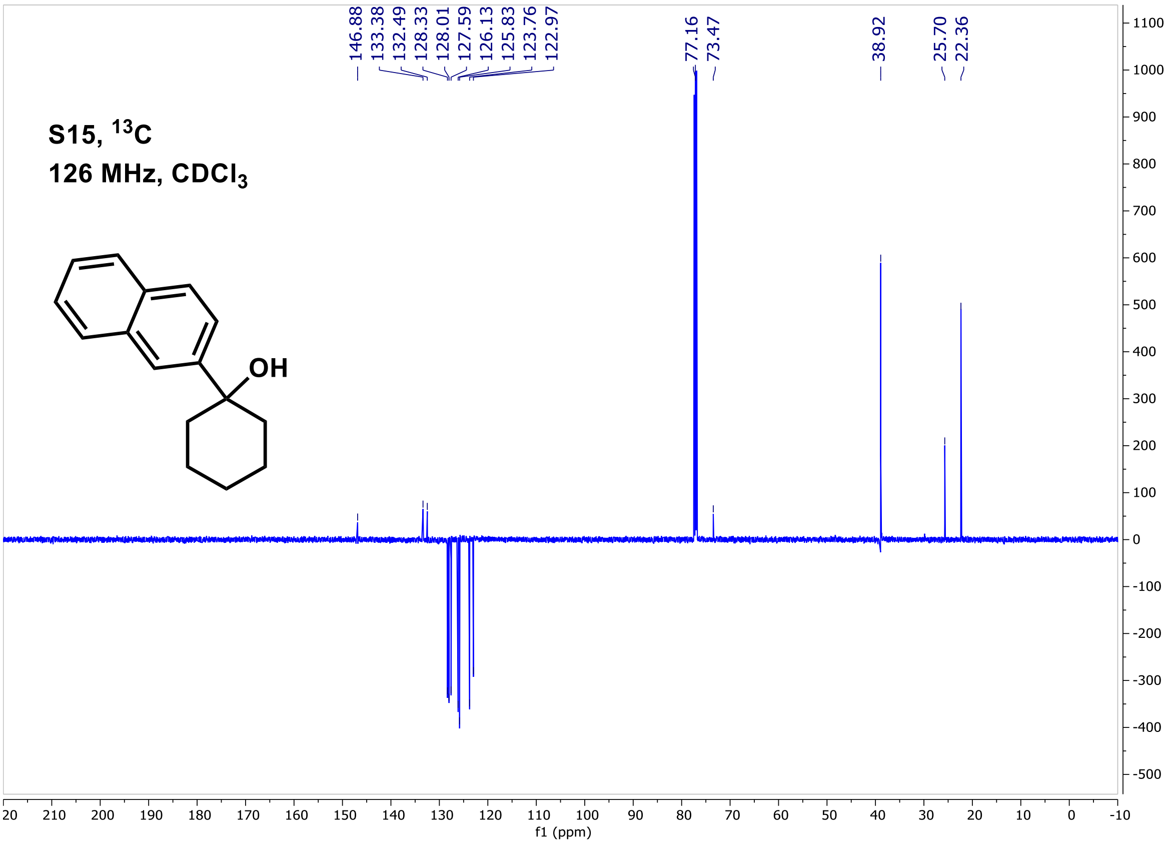

146.88 133.38 132.49 128.33 128.01 127.59 126.13 125.83 123.76 122.97 77.16 73.47 38.92 25.70 22.36
S15, 13C
126 MHz, CDCl3
OH
f1 (ppm)

1-(phenanthren-9-yl)cyclohexan-1-ol (S16)

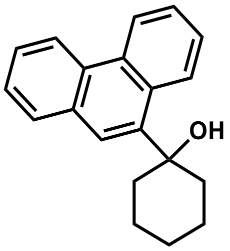

Prepared according to General Procedure B using 9-bromophenanthrene (1.67 g, 6.50 mmol, 1 equiv.) n-butyllithium (2.6 mL, 2.5 M in hexanes, 6.50 mmol, 1 equiv.), and cyclohexanone (518 µL, 5.00 mmol, 1 equiv.). The crude residue was purified by flash column chromatography (2→4% EtOAc/Hexanes, silica gel) to afford **S16** (410 mg, 30%) as an off-white solid.

R_f = 0.27 (5% EtOAc/Petrol); **M.p.**= 66-68 °C **^{1}H NMR (500 MHz, $CDCl_3$)** δ = 9.04 (dd, *J* = 8.4, 1.3 Hz, 1H), 8.76 (dd, *J* = 8.1, 1.6 Hz, 1H), 8.69 – 8.62 (m, 1H), 7.89 – 7.82 (m, 2H), 7.67 – 7.55 (m, 4H), 2.38 (d, J = 12.4 Hz, 2H), 2.10 (td, *J* = 13.1, 3.9 Hz, 2H), 1.97 (m, 2H), 1.87 – 1.79 (m, 2H), 1.79 – 1.71 (m, 2H), 1.38 (m, 1H); **^{13}C NMR (126 MHz, $CDCl_3$)** δ = 142.0, 131.9, 131.3, 130.4, 130.3, 129.1, 128.5, 126.9, 126.8, 126.0, 125.9, 124.1, 123.4, 122.5, 74.7, 38.5, 26.0, 22.4.

These data are consistent with those previously reported in the literature.[4]

(1r,3r,5r,7r)-2-(4-methoxyphenyl)adamantan-2-ol (S17)

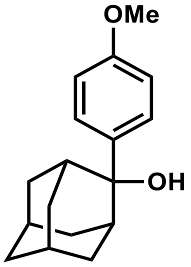

Prepared according to General Procedure B using 1-bromo-4-methoxybenzene (0.62 mL, 5.0 mmol, 1.0 equiv.), *n*-butyllithium (3.16 mL, 2.5 M in hexanes, 6.0 mmol, 1.5 equiv.) and adamantanone (1.08 g, 7.18 mmol, 1.2 equiv.). The crude residue was purified by flash column chromatography (5→10% EtOAc/Petrol, silica gel) to afford **S17** (1.08 g, 78%) as a white solid.

R$_f$ = 0.16 (10% EtOAc/Petrol); **M.p.** = 81-83 °C; **^{1}H NMR (500 MHz, CDCl$_3$)** δ = 7.47 (d, *J* = 8.9 Hz, 2H), 6.90 (d, *J* = 8.9 Hz, 2H), 3.81 (s, 3H), 2.54 (s, 2H), 2.43 – 2.36 (m, 2H), 1.90 (s, 1H), 1.77 – 1.66 (m, 9H), 1.45 (s, 1H); **^{13}C NMR (75 MHz, CDCl$_3$)** δ = 158.6, 137.7, 126.6, 114.1, 75.5, 55.4, 37.8, 35.9, 35.1, 33.1, 27.6, 27.0.

These data are consistent with those previously reported in the literature.[3]

(4aR,8aS)-1-(4-methoxyphenyl)decahydronaphthalen-1-ol (S18)

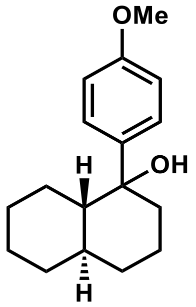

Prepared according to General Procedure A using magnesium turnings (267 mg, 11 mmol, 1.6 equiv.), 1-bromo-4-methoxybenzene (1.85 g, 1.2 mL, 9.9 mmol, 1.5 equiv.), and (4aR,8aS)-octahydronaphthalen-1(2H)-one (1.00 g, 6.6 mmol, 1 equiv.). The crude residue was purified by flash column chromatography (4→8% EtOAc/Petrol, silica gel) to afford **S18** (910 g, 53%) as a white solid.

R$_f$ = 0.24 (10% EtOAc/Petrol); **M.p.:** 58 – 60 °C; **FTIR** (ν_{max} cm^{-1}, thin film); 3523, 3462, 3003, 2954, 2927, 1606, 1581, 1477, 1460, 1386, 1369, 1301, 1278, 1149; **^{1}H NMR (500 MHz, CDCl$_3$)** δ = 7.34 (d, *J* = 8.6 Hz, 2H), 6.91 – 6.83 (m, 2H), 3.80 (s, 3H), 1.82 – 1.59 (m, 9H), 1.54 – 1.44 (m, 1H), 1.42 – 1.33 (m, 1H), 1.28 – 0.81 (m, 6H); **^{13}C NMR (126 MHz, CDCl$_3$)** δ = 158.0, 140.5, 125.9, 113.5, 75.7, 55.4, 50.9, 41.9, 37.8, 34.8, 34.1, 26.9, 26.6, 25.8, 22.0; **HRMS** (EC$^+$/Q-TOF) m/z : [M-H$_2$O+H] Calcd for [$C_{17}H_{23}O$] requires 243.1749; Found 243.1746.

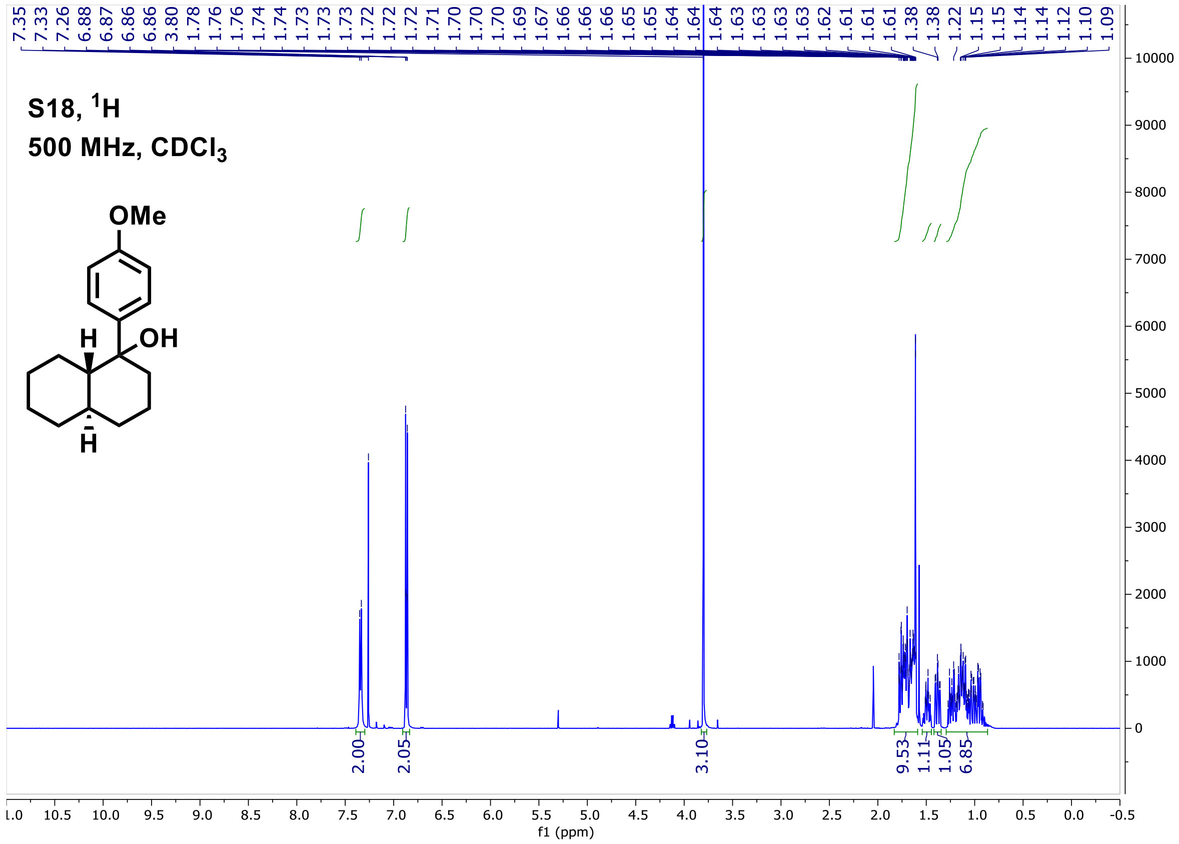
S18, 1H
500 MHz, CDCl3
OMe
H
OH
H
7.35
7.33
7.26
6.88
6.87
6.86
6.86
3.80
2.00
2.05
3.10
9.53
1.11
1.05
6.85
f1 (ppm)

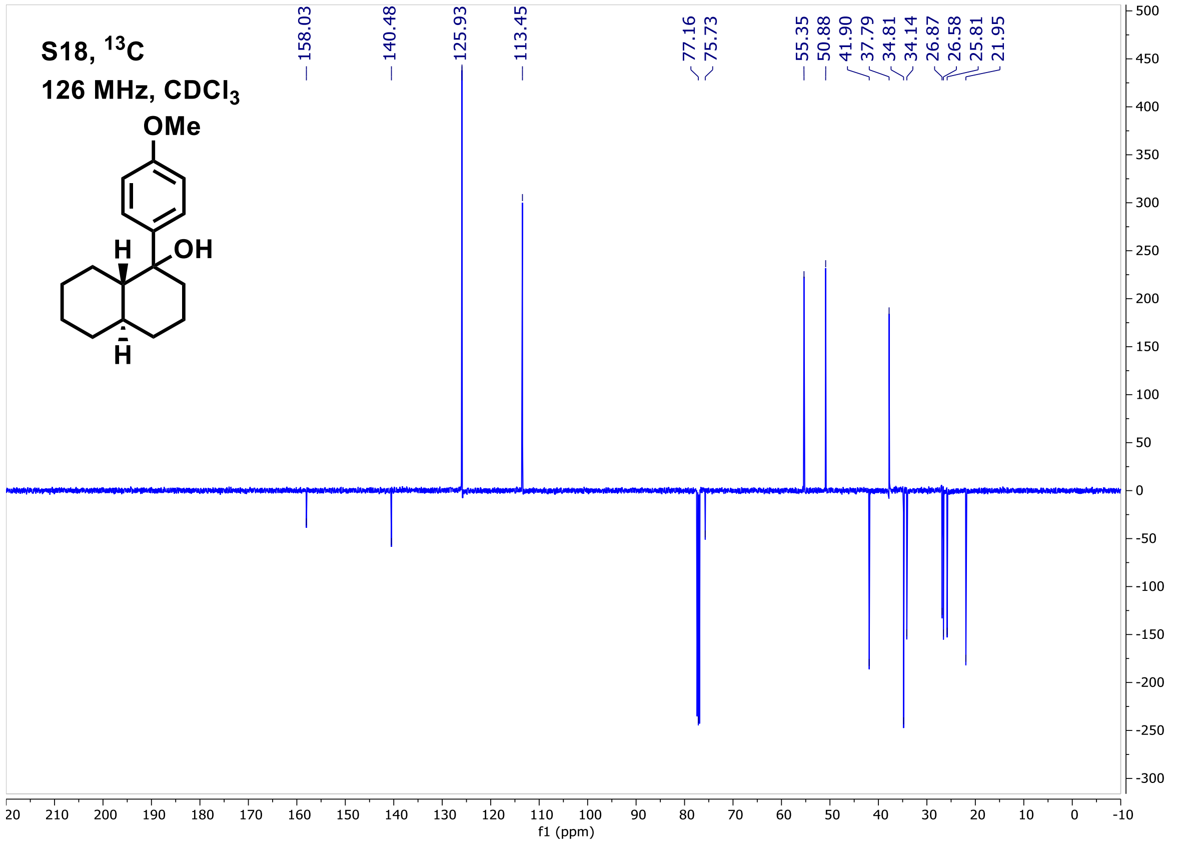
S18, 13C
126 MHz, CDCl3
OMe
H
OH
H
158.03
140.48
125.93
113.45
77.16
75.73
55.35
50.88
41.90
37.79
34.81
34.14
26.87
26.58
25.81
21.95
f1 (ppm)

1-(4-methoxyphenyl)-2-methylcyclohexan-1-ol (S19)

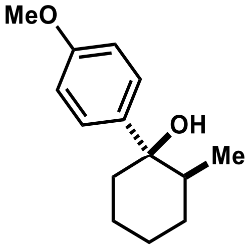

Prepared according to General Procedure A using magnesium turnings (602 mg, 24.8 mmol, 1.6 equiv.), 1-bromo-4-methoxybenzene (2.80 mL, 22.5 mmol, 1.5), and 2-methylcyclohexan-1-one (1.55 mL, 15 mmol, 1 equiv.). The crude residue was purified by flash column chromatography (5→10% EtOAc/Petrol, silica gel) to afford **S19** (2.15 g, 65%) as a colourless oil.

R_f = 0.23 (10% EtOAc/Petrol); **^{1}H NMR (500 MHz, $CDCl_3$)** δ = 7.38 – 7.33 (m, 2H), 6.91 – 6.85 (m, 2H), 3.81 (s, 3H), 1.94 – 1.84 (m, 1H), 1.78 (m, 1H), 1.75 – 1.59 (m, 6H), 1.51 – 1.32 (m, 2H), 0.62 (d, *J* = 6.8 Hz, 3H); **^{13}C NMR (126 MHz, $CDCl_3$)** δ = 158.1, 140.8, 125.9, 113.5, 75.6, 55.3, 41.5, 40.1, 30.6, 26.3, 22.2, 15.7.

These data are consistent with those previously reported in the literature.[4]

1-(4-methoxyphenyl)-3,3-dimethylcyclohexan-1-ol (S20)

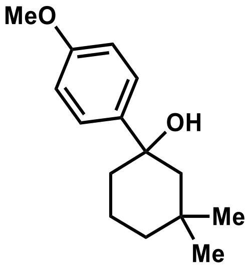

Prepared according to General Procedure A using magnesium turnings (401 mg, 17 mmol, 1.7 equiv.), 1-bromo-4-methoxybenzene (2.80 g, 1.88 mL, 15.0 mmol, 1.5 equiv.), and 3,3-dimethylcyclohexan-1-one (1.26 g, 1.39 mL, 10.0 mmol, 1.0 equiv.). The crude residue was purified by flash column chromatography (2→12% EtOAc/Petrol, silica gel) to afford **S20** (827 mg, 66%) as a white solid.

R$_f$ = 0.18 (10% EtOAc/Petrol); **M.p.** = 64-66 °C **;** ^{1}**H NMR (500 MHz, CDCl$_3$)** δ = 7.45 – 7.40 (m, 2H), 6.90 – 6.85 (m, 2H), 3.80 (s, 3H), 1.99 – 1.88 (m, 1H), 1.83 – 1.72 (m, 2H), 1.64 – 1.54 (m, 3H), 1.53 – 1.46 (m, 1H), 1.36 (s, 1H), 1.27 – 1.18 (m, 1H), 1.18 (s, 3H), 0.87 (s, 3H); ^{13}**C NMR (126 MHz, CDCl$_3$)** δ = 158.4, 142.5, 125.9, 113.6, 74.3, 55.4, 51.4, 39.0, 34.0, 31.4, 27.6, 19.1.

These data are consistent with those previously reported in the literature.[8]

4,4-difluoro-1-(4-methoxyphenyl)cyclohexan-1-ol (S21)

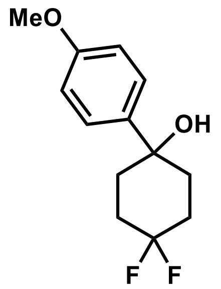

Prepared according to General Procedure A using magnesium turnings (200.5 mg, 8.25 mmol, 1.5 equiv.), 1-bromo-4-methoxybenzene (1.40 g, 0.94 mL, 7.5 mmol, 1.5 equiv.), and 4,4-difluorocyclohexan-1-one (671 mg, 5 mmol, 1 equiv.). The crude residue was purified by flash column chromatography (10→15% EtOAc/Petrol, silica gel) to afford **S21** (713 mg, 59%) as a colourless crystalline solid.

R$_f$ = 0.23 (15% EtOAc/Petrol); **M.p. =** 41-42 °C; ^{1}**H NMR (500 MHz, CDCl$_3$)** δ = 7.48 – 7.37 (m, 2H), 6.93 – 6.84 (m, 2H), 3.81 (s, 3H), 2.40 – 2.21 (m, 2H), 2.19 – 2.09 (m, 2H), 2.08 – 1.98 (m, 2H), 1.93 – 1.84 (m, 2H), 1.43 (s, 1H). ^{13}**C NMR (126 MHz, CDCl$_3$)** δ = 159.0, 139.7, 125.8, 124.0 (dd, *J* = 242.7, 238.5 Hz), 113.9, 71.6 (d, *J* = 1.6 Hz), 55.4, 35.5 (d, *J* = 9.6 Hz), 30.1 (app t, *J* = 25.2 Hz). ^{19}**F NMR (471 MHz, CDCl$_3$)** δ = -92.6 (d, *J* = 235.0 Hz), -104.20 (dt, *J* = 235.4 Hz, 34.3 Hz).

These data are consistent with those previously reported in the literature.[4]

9-(4-methoxyphenyl)-3,3-dimethyl-1,5-dioxaspiro[5.5]undecan-9-ol (*S*22)

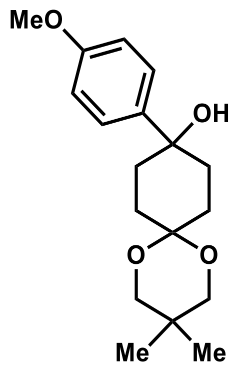

Prepared according to General Procedure B using 1-bromo-4-methoxybenzene (0.62 mL, 5.0 mmol, 1.0 equiv.), *n*-butyllithium (3.16 mL, 2.5 M in hexanes, 6.0 mmol, 1.5 equiv.) and 3,3-dimethyl-1,5-dioxaspiro[5.5]undecan-9-one (1.18 g, 5.5 mmol, 1.1 equiv.). The crude residue was purified by flash column chromatography (5→10% EtOAc/Petrol, silica gel) to afford **S22** (1.05 g, 70%) as a colourless oil.

R_f = 0.39 (15% EtOAc/Petrol); **FTIR** (ν_{max} cm^{-1}, thin film); 3448 (bs), 2951, 2868, 1698, 1512, 1470, 1389, 1365, 1249, 1178, 1037, 985, 830; **^{1}H NMR (500 MHz, $CDCl_3$)** δ = 7.43 (d, *J* = 9.0 Hz, 2H), 6.87 (d, *J* = 8.8 Hz, 2H), 3.80 (s, 3H), 3.58 (s, 2H), 3.51 (s, 2H), 2.22 – 2.13 (m, 2H), 2.06 (m, 2H), 1.90 (Hz, 2H), 1.76 – 1.68 (m, 2H), 1.47 (s, 1H), 0.99 (s, 6H); **^{13}C NMR (126 MHz, $CDCl_3$)** δ = 158.6, 140.8, 125.9, 113.6, 97.3, 72.6, 70.3, 70.2, 55.4, 35.5, 30.4, 28.3, 22.9; **HRMS** (ES$^+$/Q-TOF) m/z : [M-H_2O+H] Calcd for [$C_{18}H_{25}O_3$] requires 289.1804; Found 289.1815.

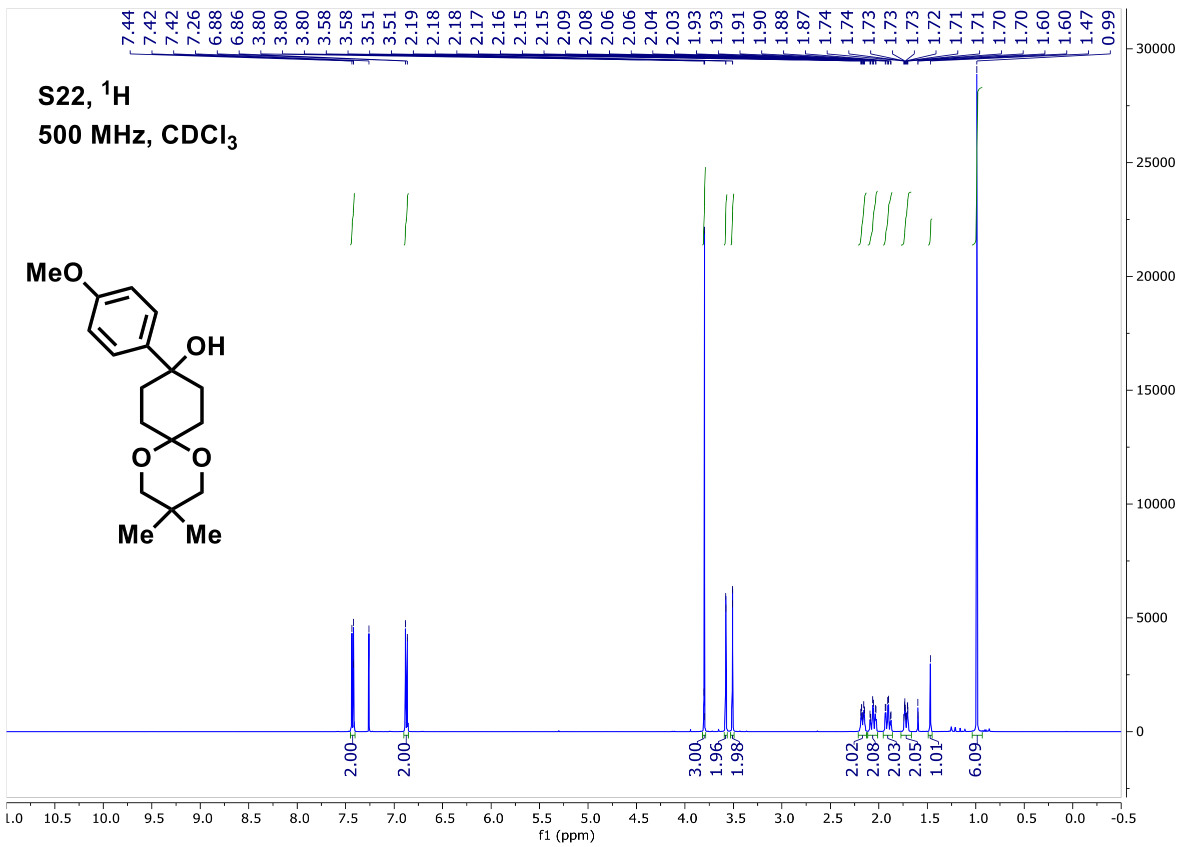

S22, ¹H
500 MHz, CDCl₃
MeO
OH
O
O
Me
Me
7.44 7.42 7.42 7.26 6.88 6.86 3.80 3.80 3.80 3.58 3.51 3.51 2.19 2.18 2.18 2.17 2.16 2.15 2.15 2.09 2.08 2.06 2.06 2.04 2.03 1.93 1.93 1.91 1.90 1.88 1.87 1.74 1.74 1.73 1.73 1.73 1.72 1.71 1.71 1.70 1.70 1.60 1.60 1.47 0.99
2.00 2.00 3.00 1.96 1.98 2.02 2.08 2.03 2.05 1.01 6.09
f1 (ppm)

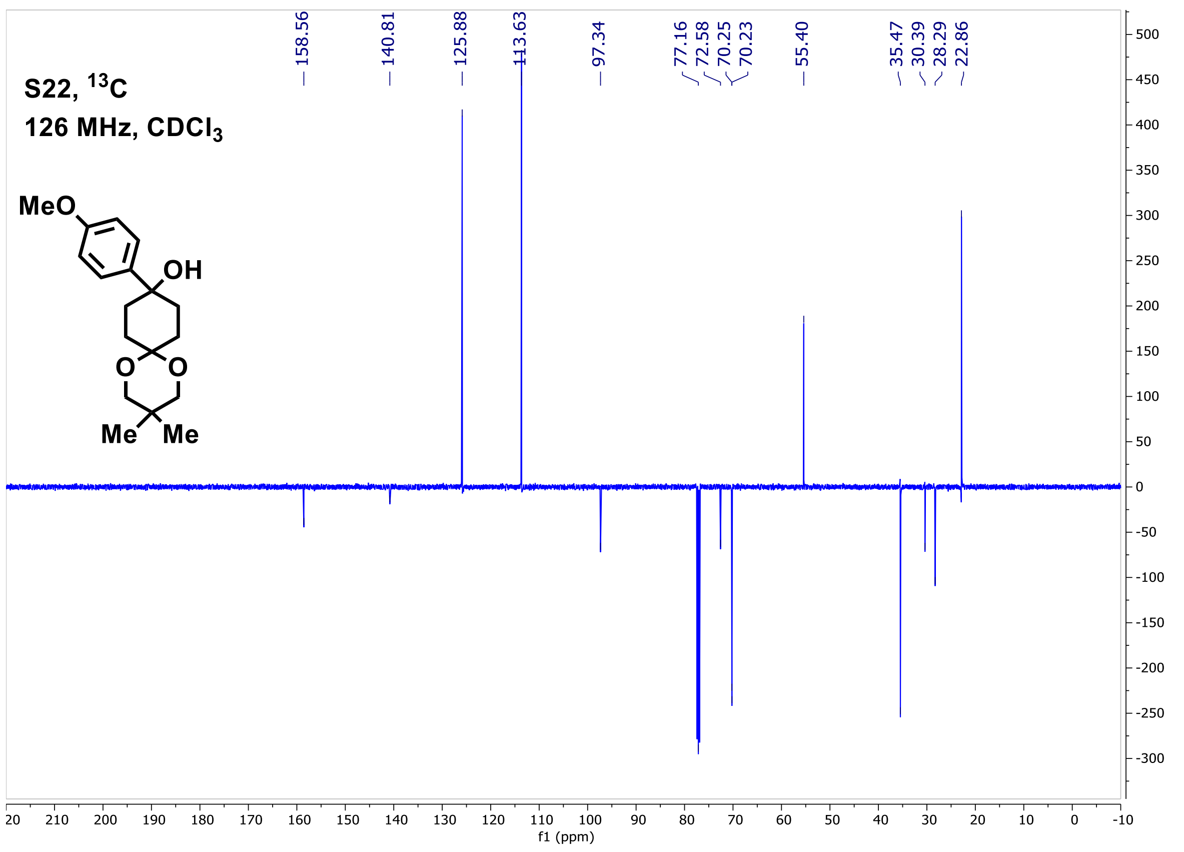

S22, ¹³C
126 MHz, CDCl₃
MeO
OH
O
O
Me
Me
158.56
140.81
125.88
113.63
97.34
77.16
72.58
70.25
70.23
55.40
35.47
30.39
28.29
22.86
f1 (ppm)

1-(4-methoxyphenyl)-1,2,3,4-tetrahydronaphthalen-1-ol (S23)

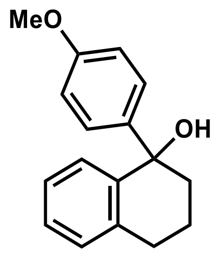

Prepared according to General Procedure A using magnesium turnings (401 mg, 17 mmol, 1.7 equiv.), 1-bromo-4-methoxybenzene (2.80 g, 1.88 mL, 15.0 mmol, 1.5 equiv.), and 3,4-dihydronaphthalen-1(2H)-one (1.46 g, 1.33 mL, 10.0 mmol, 1.0 equiv.). The crude residue was purified by flash column chromatography (8→10% EtOAc/Petrol, silica gel) to afford **S23** (1.85 g, 73%) as a colourless oil.

R_f = 0.16 (10% EtOAc/Petrol); **FTIR** (ν_{max} cm^{-1}, thin film); 3448 (br), 2933, 2835, 1608, 1487, 1440, 1508, 1440, 1300, 1244, 1031, 975; **^{1}H NMR (500 MHz, $CDCl_3$)** δ = 7.26 – 7.21 (m, 2H), 7.21 – 7.08 (m, 4H), 6.86 – 6.81 (m, 2H), 3.80 (s, 3H), 3.04 – 2.76 (m, 2H), 2.17 – 2.10 (m, 3H), 2.04 – 1.93 (m, 1H), 1.82 – 1.72 (m, 1H); **^{13}C NMR (126 MHz, $CDCl_3$)** δ = 158.4, 142.3, 141.2, 137.7, 129.0, 128.9, 127.7, 127.6, 126.6, 113.2, 75.3, 55.4, 41.6, 30.0, 19.8; **HRMS** (ES^+/Q-TOF) m/z : [M-H_2O+H] Calcd for [$C_{17}H_{17}O$] 237.1279; Found 237.1275.

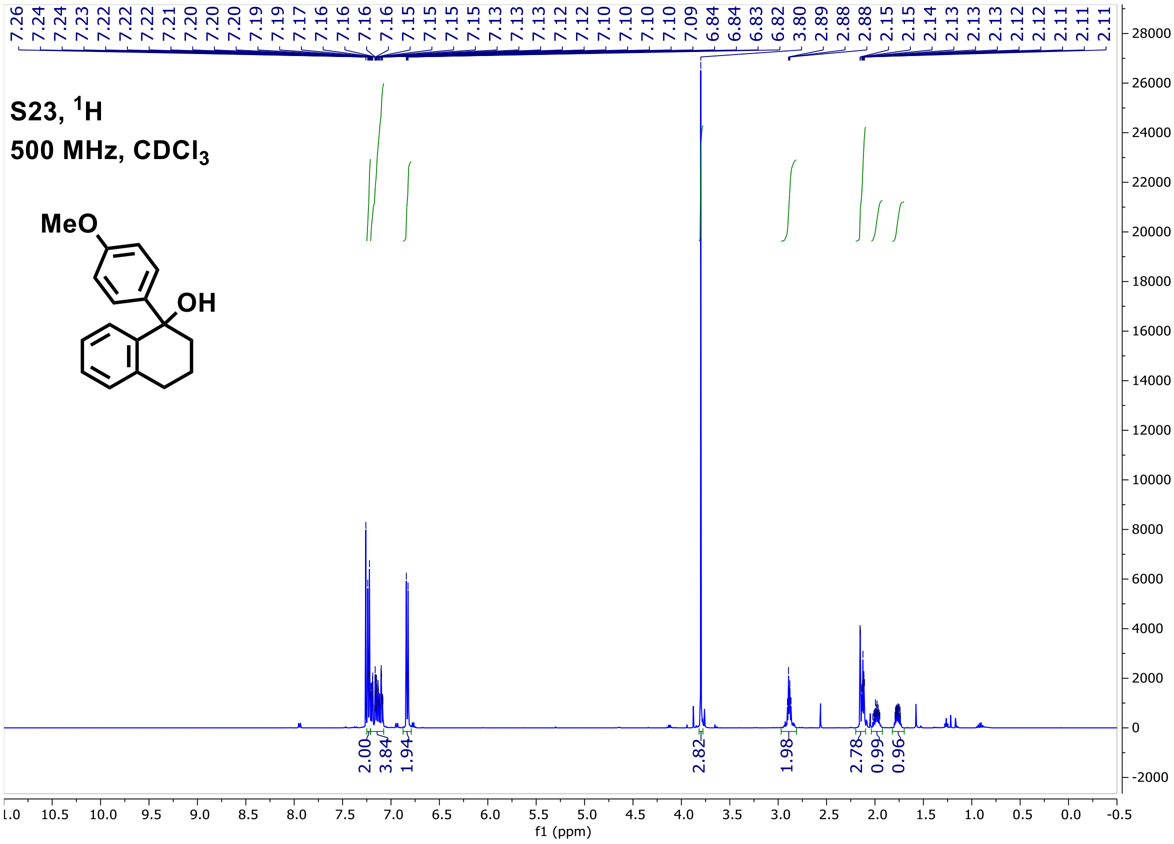
S23, 1H
500 MHz, CDCl3
MeO
OH
7.26
7.24
7.24
7.23
7.22
7.22
7.22
7.21
7.20
7.20
7.20
7.19
7.19
7.17
7.16
7.16
7.16
7.16
7.15
7.15
7.15
7.15
7.13
7.13
7.13
7.12
7.12
7.10
7.10
7.10
7.09
6.84
6.84
6.83
6.82
3.80
2.89
2.88
2.88
2.15
2.15
2.14
2.13
2.13
2.13
2.12
2.12
2.11
2.11
2.11
2.00
3.84
1.94
2.82
1.98
2.78
0.99
0.96
f1 (ppm)

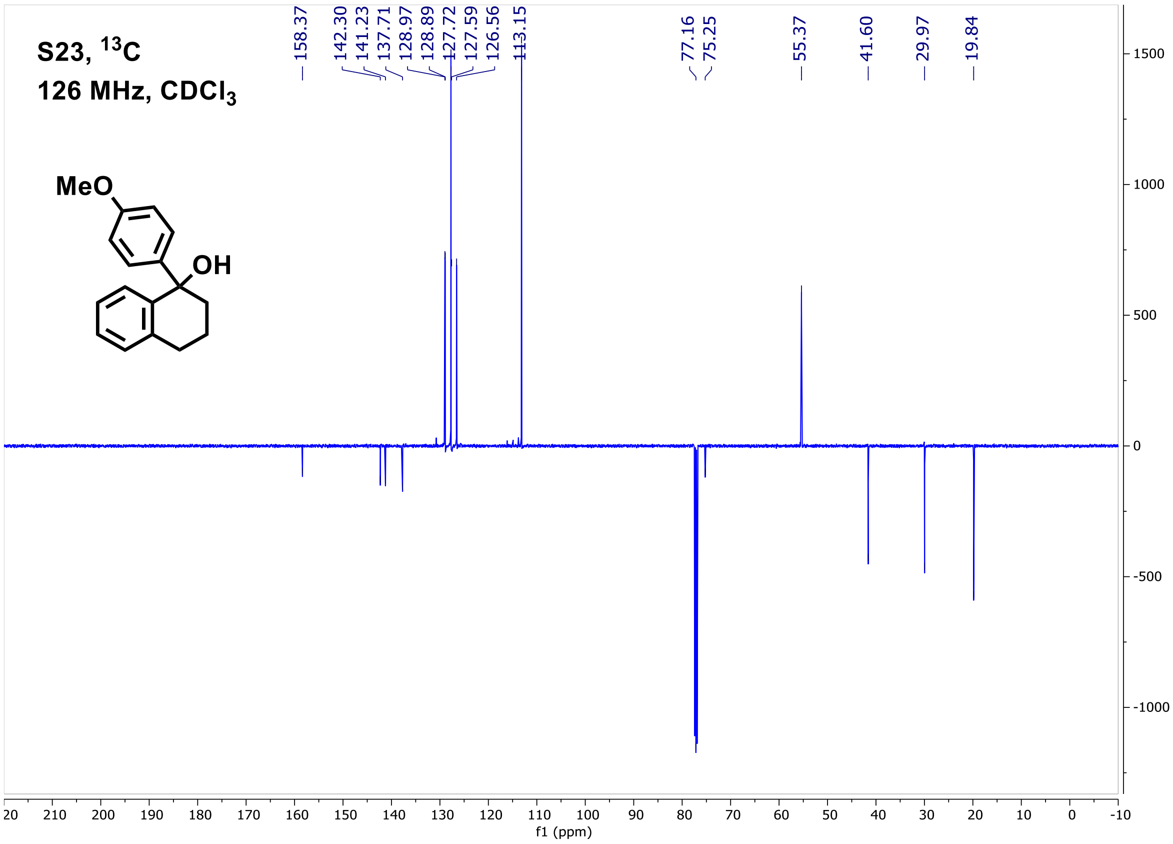
S23, 13C
126 MHz, CDCl3
MeO
OH
158.37
142.30
141.23
137.71
128.97
128.89
127.72
127.59
126.56
113.15
77.16
75.25
55.37
41.60
29.97
19.84
f1 (ppm)

tert-butyl 4-hydroxy-4-(4-methoxyphenyl)piperidine-1-carboxylate (S24)

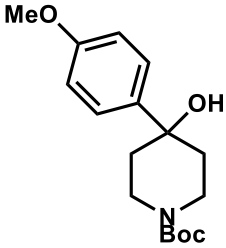

Prepared according to General Procedure A using magnesium turnings (201 mg, 8.30 mmol, 1.6 equiv.), 1-bromo-4-methoxybenzene (0.94 g, 0.63 mL, 5.0 mmol, 1.0 equiv.), and tert-butyl 4-oxopiperidine-1-carboxylate (1.20 g, 6.00 mmol, 1.2 equiv.). The crude residue was purified by flash column chromatography (20→25% EtOAc/Petrol, silica gel) to afford **24** (850 mg, 55%) white solid.

R_f = 0.23 (10% EtOAc/Petrol); **M.p.** = 80-82 °C; **FTIR** (ν_{max} cm^{-1}, thin film) 3350 (br), 2972, 2328, 1687, 1600, 1575, 1510, 1456, 1367, 1257, 1168, 1029, 833; **^{1}H NMR (500 MHz, $CDCl_3$)** δ = 7.39 (d, *J* = 8.2 Hz, 2H), 6.89 (d, *J* = 8.2 Hz, 2H), 4.03 – 3.94 (m, 2H), 3.81 (s, 3H), 3.24 (m, 2H), 2.01 – 1.91 (m, 2H), 1.78 – 1.67 (m, 2H), 1.55 (s, 1H), 1.48 (s, 9H); **^{13}C NMR (126 MHz, $CDCl_3$)** δ = 159.1, 155.0, 140.3, 125.8, 113.9, 79.6, 71.3, 55.5, 38.3, 28.6; **HRMS** (CI^+/FTMS) m/z : [M-H_2O+H] Calcd for [$C_{17}H_{24}O_3N$] requires 290.1756; Found 290.1750.

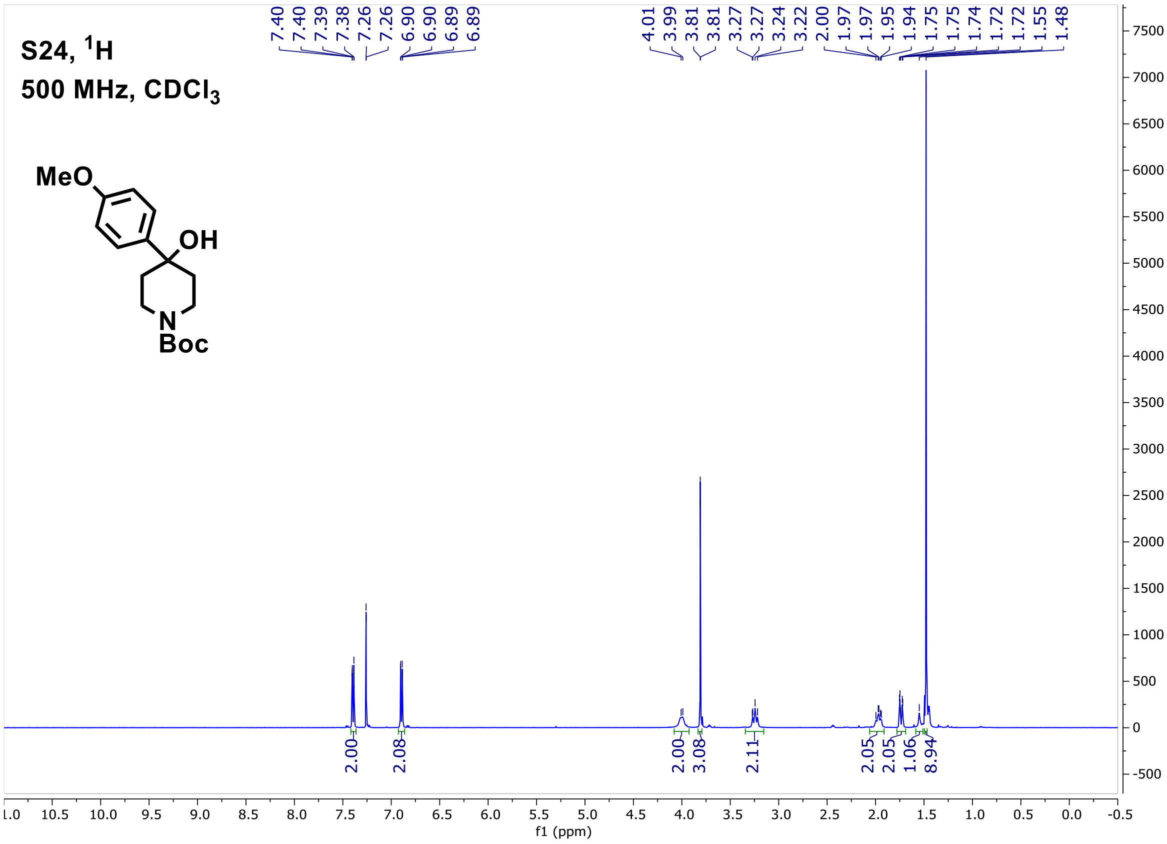
S24, 1H
500 MHz, CDCl3
MeO
OH
N
Boc
7.40 7.40 7.39 7.38 7.26 7.26 6.90 6.90 6.89 6.89
4.01 3.99 3.81 3.81 3.27 3.27 3.24 3.22 2.00 1.97 1.97 1.95 1.94 1.75 1.75 1.74 1.72 1.72 1.55 1.48
2.00 2.08 2.00 3.08 2.11 2.05 2.05 1.06 8.94
f1 (ppm)

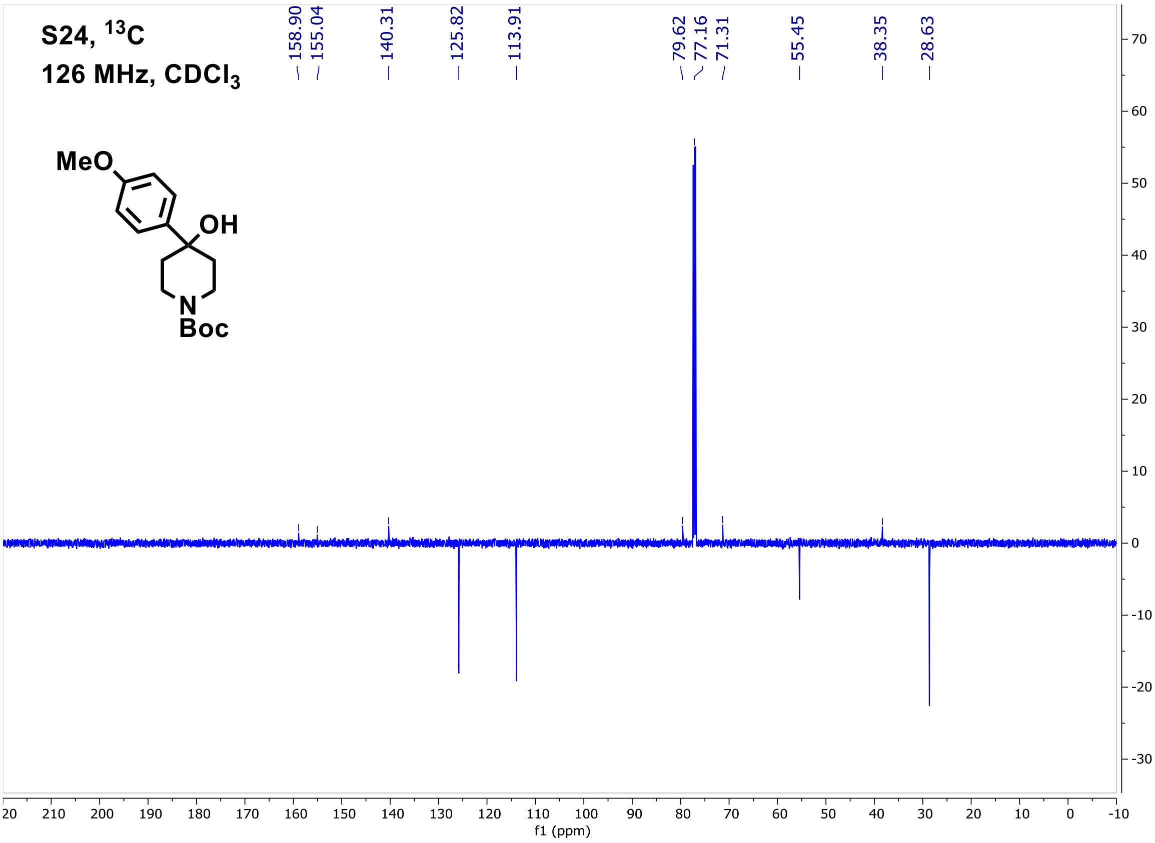
S24, 13C
126 MHz, CDCl3
MeO
OH
N
Boc
158.90 155.04 140.31 125.82 113.91 79.62 77.16 71.31 55.45 38.35 28.63
f1 (ppm)

tert-butyl 3-hydroxy-3-(4-methoxyphenyl)piperidine-1-carboxylate (S25)

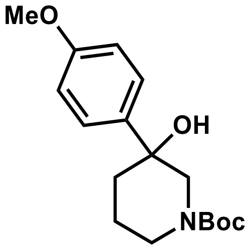

Prepared according to General Procedure A using magnesium turnings (401 mg, 16.5 mmol, 1.6 equiv.), 1-bromo-4-methoxybenzene (2.80 g, 1.88 mL, 15.0 mmol, 1.5 equiv.), and tert-butyl 3-oxopiperidine-1-carboxylate (671 mg, 10 mmol, 1.0 equiv.). The crude residue was purified by flash column chromatography (17→23% EtOAc/Petrol, silica gel) to afford **25** (1.63 g, 53%) as a colourless oil.

$\mathbf{R_f}$ = 0.11 (20% EtOAc/Petrol); **^{1}H NMR (500 MHz, $CDCl_3$)** δ = 7.50 – 7.38 (m, 2H), 6.96 – 6.79 (m, 2H), 4.15 – 3.84 (m, 2H), 3.81 (s, 3H), 3.16 (d, *J* = 11.8 Hz, 1H), 2.91 – 2.74 (m 1H), 2.05 – 1.81 (m, 3H), 1.70 (s, 1H), 1.65 – 1.54 (m, 1H), 1.47 (s, 9H); **^{13}C NMR (126 MHz, $CDCl_3$)** δ = 158.9, 137.8, 130.4, 126.2, 113.8, 80.1, 71.6, 55.4, 39.9, 37.0, 28.5, 28.5, 21.5.

These data are consistent with those previously reported in the literature.[8]

tert-butyl 4-hydroxy-4-(4-methoxyphenyl)azepane-1-carboxylate (*S*26)

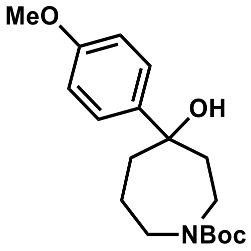

Prepared according to General Procedure A using magnesium turnings (201 mg, 8.30 mmol, 1.6 equiv.), 1-bromo-4-methoxybenzene (0.940 g, 0.63 mL, 5.00 mmol, 1.0 equiv.), and tert-butyl 4-oxoazepane-1-carboxylate (1.28 g, 6.00 mmol, 1.2 equiv.). The crude residue was purified by flash column chromatography (17→23% EtOAc/Petrol, silica gel) to afford **26** (424 mg, 26%) as a pale yellow solid.

R_f = 0.11 (20% EtOAc/Petrol); **M.p.** = 93-95 °C; **FTIR** (ν_{max} cm^{-1}, thin film); 3398 (br), 2933, 1660, 1510, 1477, 1222, 977; **^{1}H NMR (500 MHz, $CDCl_3$)** δ = 7.38 – 7.36 (m, 2H), 6.88 – 6.85 (m, 2H), 3.80 (s, 3H), 3.75 – 3.48 (m, 2H), 3.42 – 3.26 (m, 2H), 2.72 – 2.57 (m, 1H), 2.25 – 2.10 (m, 1H), 2.06 – 2.01 (m, 1H), 1.97 – 1.68 (m, 4H), 1.46 (s, 9H); **^{13}C NMR (126 MHz, $CDCl_3$)** δ = 158.5, 156.0, 142.2, 125.6, 113.7, 79.4, 74.7, 55.4, 47.0, 45.8, 44.2, 39.8, 28.7. 21.0; **HRMS** (CI^+/FTMS) m/z : [M] Calcd for [$C_{18}H_{27}O_4N$] requires 321.1940; Found 321.1936.

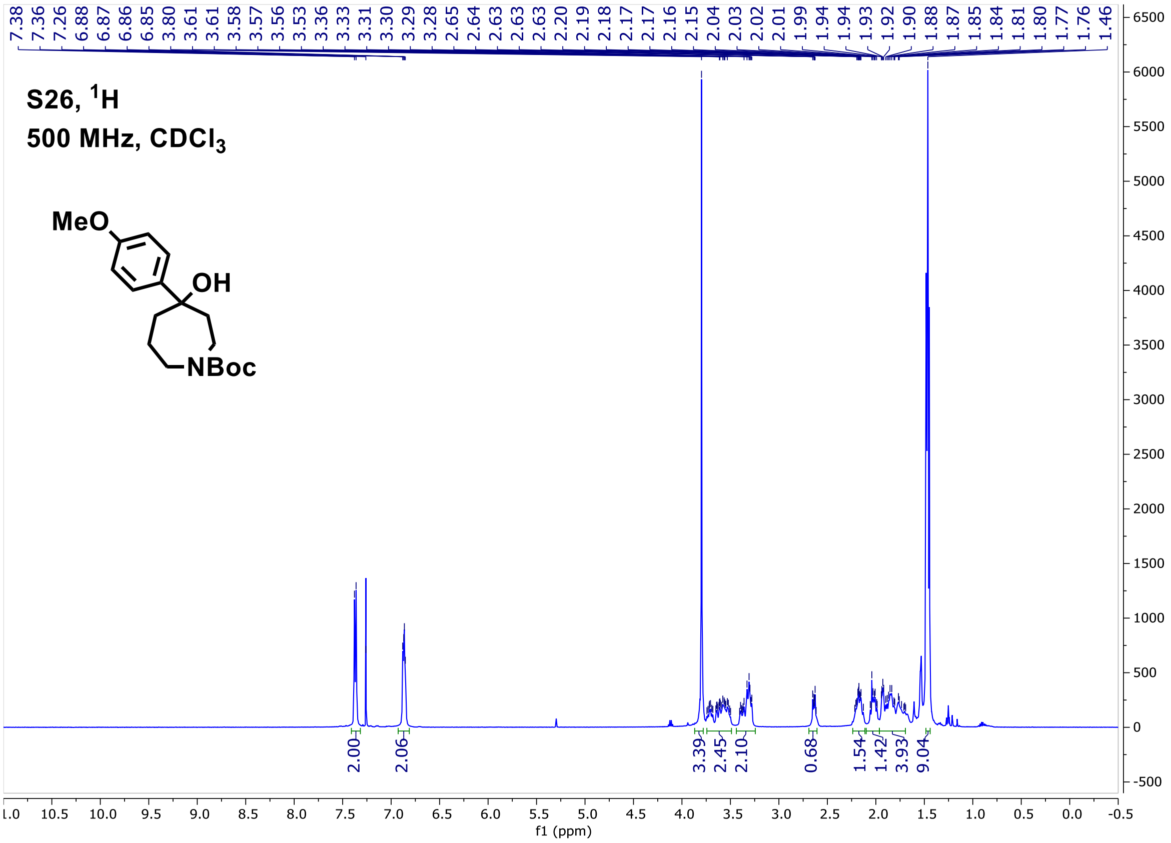
S26, 1H
500 MHz, CDCl3
MeO
OH
NBoc
7.38 7.36 7.26 6.88 6.87 6.86 6.85 3.80 3.61 3.61 3.58 3.57 3.56 3.53 3.36 3.33 3.31 3.30 3.29 3.28 2.65 2.64 2.63 2.63 2.63 2.20 2.19 2.18 2.17 2.17 2.16 2.15 2.04 2.03 2.02 2.01 1.99 1.94 1.94 1.93 1.92 1.90 1.88 1.87 1.85 1.84 1.81 1.80 1.77 1.76 1.46
2.00 2.06 3.39 2.45 2.10 0.68 1.54 1.42 3.93 9.04
f1 (ppm)

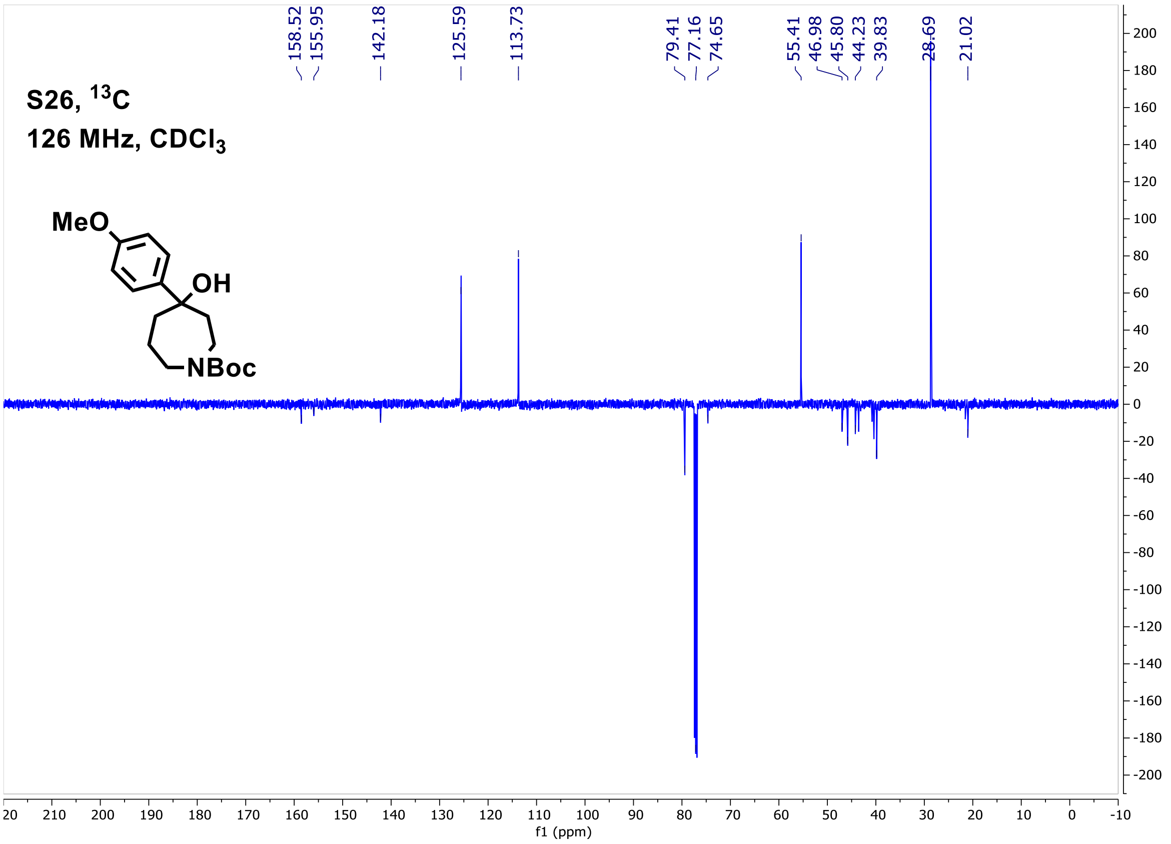
S26, 13C
126 MHz, CDCl3
MeO
OH
NBoc
158.52 155.95 142.18 125.59 113.73 79.41 77.16 74.65 55.41 46.98 45.80 44.23 39.83 28.69 21.02
f1 (ppm)

2-(4-methoxyphenyl)-1-phenylpropan-2-ol (*S*27)

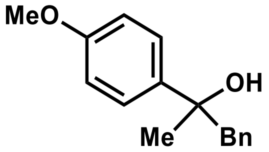

Prepared according to General Procedure A using magnesium turnings (602 mg, 24.8 mmol, 1.6 equiv.), 1-bromo-4-methoxybenzene (4.20 g, 2.8 mL, 22.5 mmol, 1.5 equiv.), and 1-phenylpropan-2-one (2.10 g, 2.1 mL, 15.0 mmol, 1.0 equiv.). The crude residue was purified by flash column chromatography (2→10% EtOAc/Petrol, silica gel) to afford **S27** (1.67 g, 46%) as a colourless oil.

R_f = 0.23 (10% EtOAc/Petrol); **^{1}H NMR (500 MHz, $CDCl_3$)** δ = 7.41 – 7.33 (m, 2H), 7.33 – 7.23 (m, 3H), 7.09 – 7.01 (m, 2H), 6.96 – 6.88 (m, 2H), 3.85 (s, 3H), 3.11-3.14 (m, 2H), 2.14 (s, 1H), 1.60 (s, 3H); **^{13}C NMR (126 MHz, $CDCl_3$)** δ = 158.4, 139.9, 137.0, 130.8, 128.8, 126.7, 126.3, 113.5, 74.3, 55.4, 50.8, 29.6.

These data are consistent with those previously reported in the literature.[8]

2.3. Synthesis of Redox Mediators

4-Nitro-*N*-(4-nitrophenyl)-*N*-phenylaniline ($N(4\text{-}NO_2C_6H_4)_2Ph$) (RM-2)

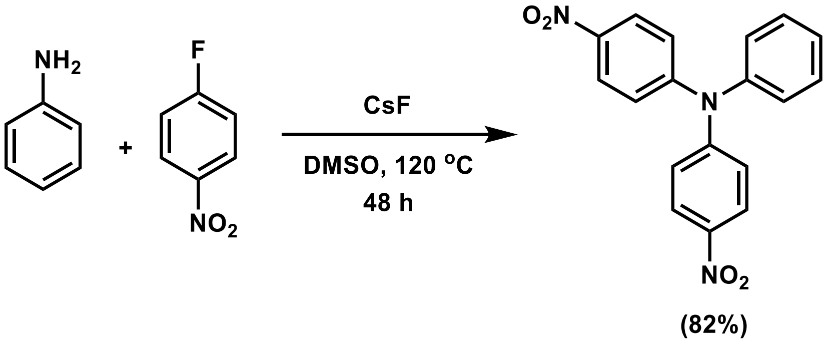

Prepared by using aniline (2.15 g, 23.1 mmol, 1.0 equiv.), 4-fluoronitrobenzene (7.24 g, 51.3 mmol, 2.2 equiv.) and cesium fluoride (7.79 g, 51.3 mmol. 2.2 equiv.) were dissolved in dried 100 ml DMSO, and the reactant mixture was stirred at 120 °C using a drysyn heating block with temperature probe hot stirring plate for 48 h. The reaction mixture was cooled to room temperature, poured into 500 mL of cold water and the

precipitated orange solid was then collected by filtration and washed with methanol to afford **RM-2** (6.73 g, 82%) as a yellow solid.

M.p. = 191-193 °C (lit.[9] 194-196 °C); **^{1}H NMR (500 MHz, $CDCl_3$)** δ = 8.23 – 8.06 (m, 4H), 7.53 – 7.38 (m, 2H), 7.36 – 7.30 (m, 1H), 7.22 – 7.11 (m, 6H); **^{13}C NMR (101 MHz, $CDCl_3$)** δ = 151.9, 145.0, 142.9, 130.6, 127.4, 127.2, 125.6, 122.4

These data are consistent with those previously reported in the literature.[10]

Tris(4-chloro-2-nitrophenyl)amine (RM-4)

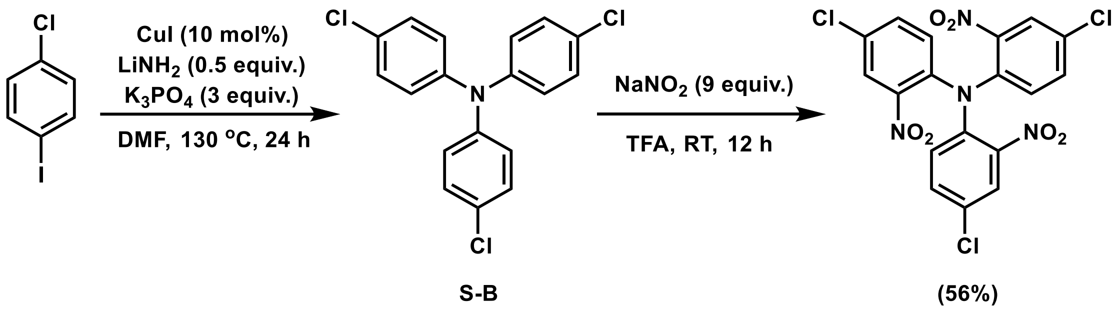

To an oven dried seal-tube, was added 4-chloro iodobenzene (1.17 g, 5.00 mmol), $LiNH_2$ (60 mg, 0.50 mmol), CuI (95 mg, 10 mol%), K_3PO_4 (3.18 g, 15.0 mmol, 3.0 equiv.) and the tube was backfilled with N_2 3 times before anhydrous DMF (10 mL) was added . The tube was sealed and heater to 130 °C for 24 h. After cooling to room temperature, water was added to the reaction mixture and extracted with DCM x 3. Combined organic layers were dried over $MgSO_4$ and distilled under reduced pressure. The obtained crude residue was purified by flash column chromatography (100% petrol, silica gel) to afford **S-B** (0.98 g, 41%) as a white solid and was used directly in the next step.

To a solution of **S-B** (349 mg, 1.00 mmol) in TFA (100 mL) was added sodium nitrite (625 mg, 9.00 mmol). The reaction turned first deep blue then green in colour. After stirring the reaction mixture for overnight, water was added, and the reaction mixture was them cooled to 0 °C using ice-bath. While stirring, NaOH pellets (50 g) were added slowly (*exothermic*) until the solution was alkaline. The product was extracted with DCM (10 mL x 3) and washed with cold water and dried under vacuum over night to afford **RM-4** (290 mg, 60%) as a yellow amorphous solid.

M.p. = 299-301 °C (lit.[11] 302-303 °C); **^{1}H NMR (500 MHz, DMSO-d$_6$)** δ = 8.15 (d, *J* = 2.5 Hz, 3H), 7.76 (dd, *J* = 8.8, 2.5 Hz, 3H), 7.29 (d, *J* = 8.8 Hz, 3H); **^{13}C NMR (126 MHz, DMSO-d$_6$)** δ = 143.8, 136.5, 134.5, 130.5, 129.7, 126.1.

These data are consistent with those previously reported in the literature.[10]

2-(4-bromophenyl)-1-methyl-4,5-diphenyl-1H-imidazole (RM-5)

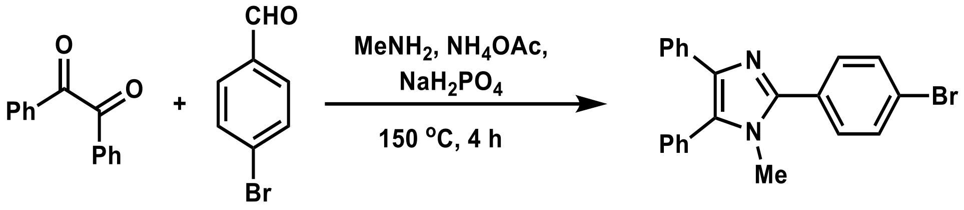

RM-5 (69%)

Prepared according to a previously reported procedure[12] using Benzil (1.05 g, 5.00 mmol), 4-Bromobenzaldehyde (0.91 g, 5.00 mmol), methyl amine (40% aq) (173 µL, 5.00 mmol), ammonium acetate (385 mg, 5.0 mmol) and sodium phosphate (180 mg, 1.50 mmol) taken in sealed tube and stirred at 150 °C using a drysyn heating block with temperature probe hot stirring plate for 4 h. Then the reaction mixture was cooled to room temperature. Acetone was added to dissolve the mixture and the undissolved residue was removed by filtration. After concentration of the filterate under reduced pressure, the resulting solid residue was recrystallized from acetone–water to obtain pure compound as a yellow solid (1.27 g, 69%).

M.p. = 200-202 °C (lit.[13] 198-200 °C); **^{1}H NMR (400 MHz, DMSO-d$_6$)** δ = 7.75 (m, 3H), 7.53 (d, *J* = 7.4 Hz, 3H), 7.48 – 7.36 (m, 5H), 7.22 (t, *J* = 7.4 Hz, 2H), 7.14 (t, *J* = 7.3 Hz, 1H) 3.48 (s, 3H).; **^{13}C NMR (101 MHz, DMSO-d$_6$)** δ = 146.0, 137.0, 135.0, 131.5, 131.0, 130.7, 130.5, 130.0, 129.9, 129.1, 128.1, 126.3, 126.2, 122.1, 33.1

These data are consistent with those previously reported in the literature.[13]

RM-1 is commercially available and used as supplied.

3. Cyclic Voltammetry Data

General Information: Cyclic Voltammetry (CV) experiments were conducted with in a 10 mL glass vial fitted with a glassy carbon working electrode (3 mm dia., BASi), a Ag/$AgNO_3$ reference electrode and a platinum wire counter electrode. The solution of interest was purged with N_2 for 5 minutes before data collection. After data collection, ferrocene (5 mM) was added, and an additional scan was run as an internal reference.

CV data for Substrates

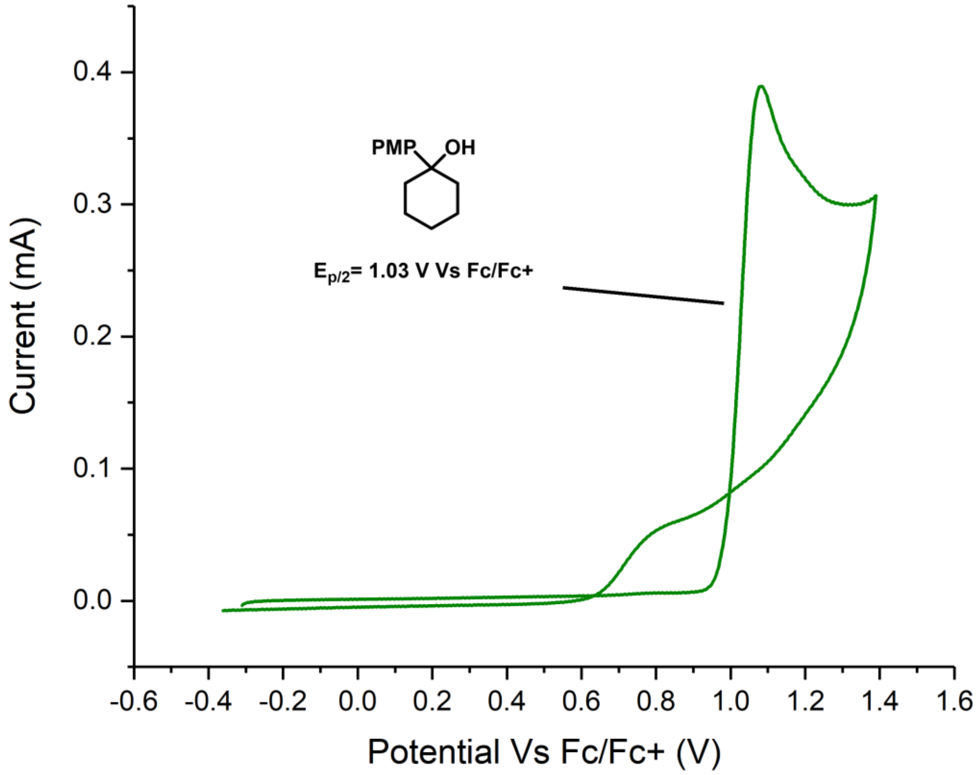

Figure S1 – Cyclic voltammogram of compound (4.0 mM) MeCN (4 mM), $LiClO_4$ (0.1 M).
***Scan rate**: 100 mV/s*

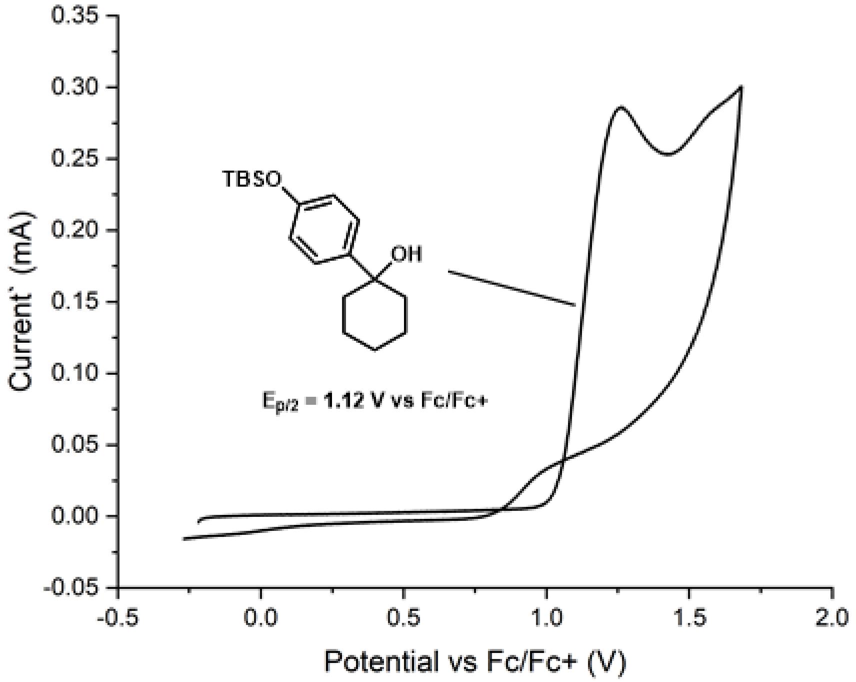

Figure S2 – Cyclic voltammogram of compound (4.0 mM) MeCN (4 mM), $LiClO_4$ (0.1 M).
***Scan rate**: 100 mV/s*

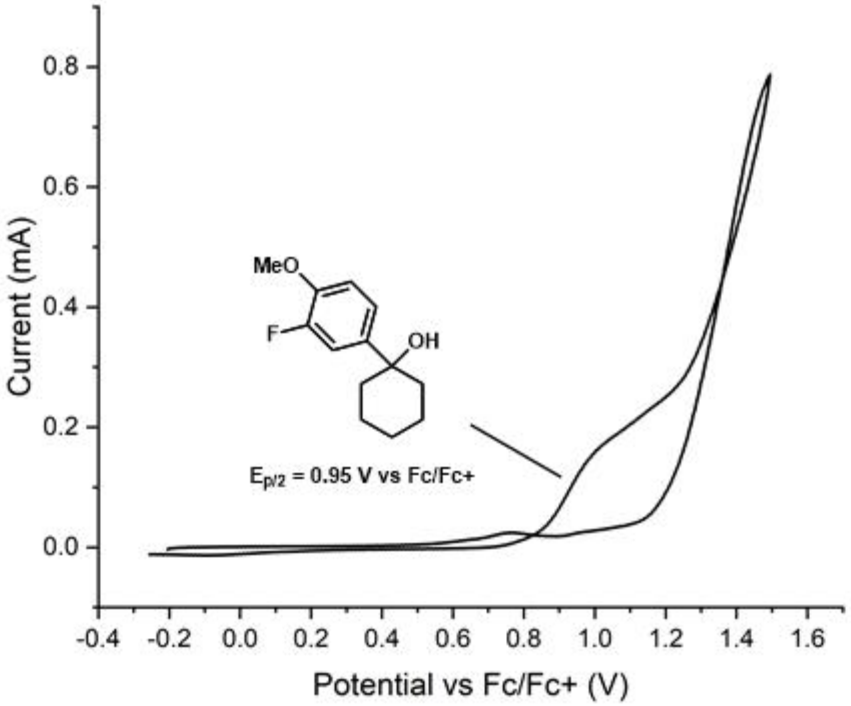

Figure S3 – Cyclic voltammogram of compound (4.0 mM) MeCN (4 mM), $LiClO_4$ (0.1 M).
***Scan rate**: 100 mV/s*

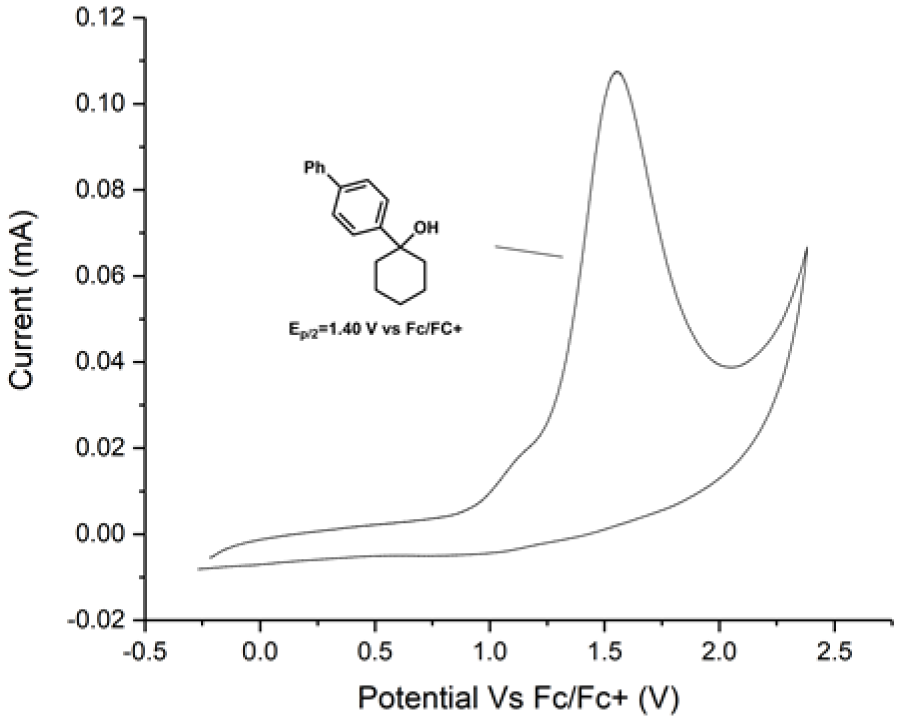

Figure S4 – Cyclic voltammogram of compound (5.0 mM) MeCN (4 mM), $LiClO_4$ (0.1 M).
***Scan rate**: 100 mV/s*

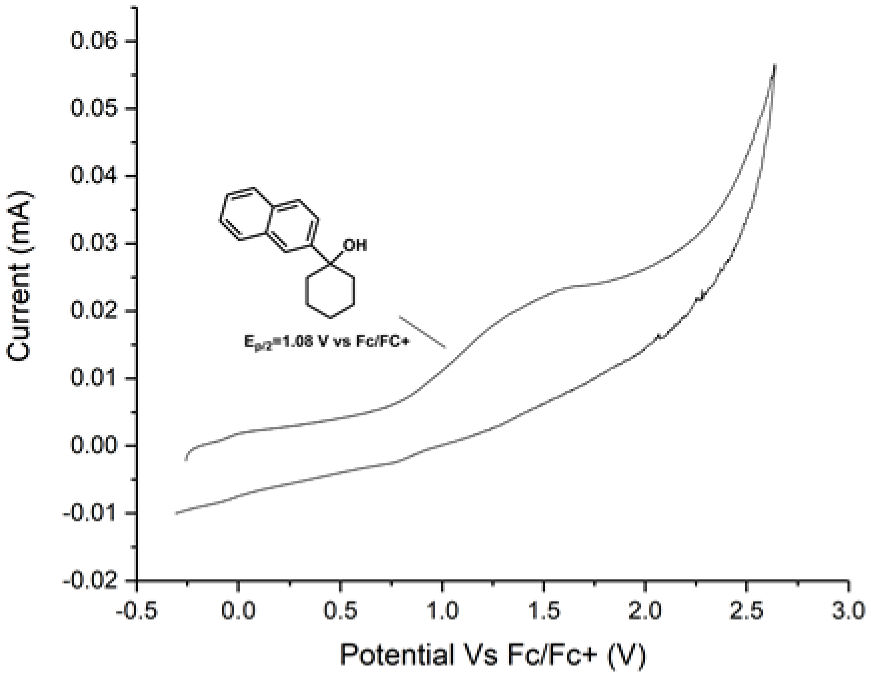

Figure S5 – Cyclic voltammogram of compound (5.0 mM) MeCN (4 mM), $LiClO_4$ (0.1 M).
***Scan rate**: 100 mV/s*

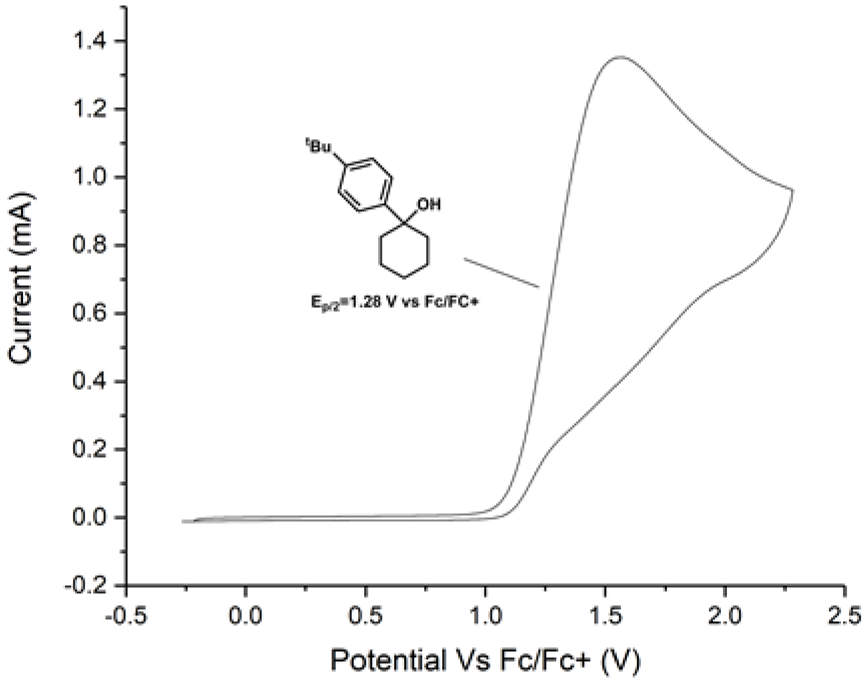

Figure S6 – Cyclic voltammogram of compound (5.0 mM) MeCN (4 mM), $LiClO_4$ (0.1 M).
***Scan rate**: 100 mV/s*

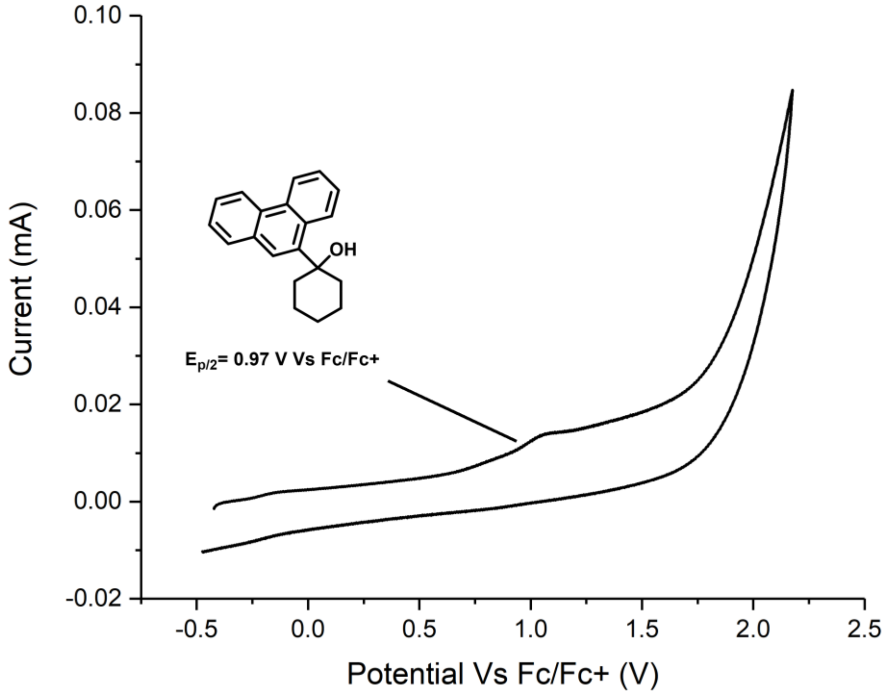

Figure S7 – Cyclic voltammogram of compound (5.0 mM) MeCN (4 mM), $LiClO_4$ (0.1 M).
***Scan rate**: 50 mV/s*

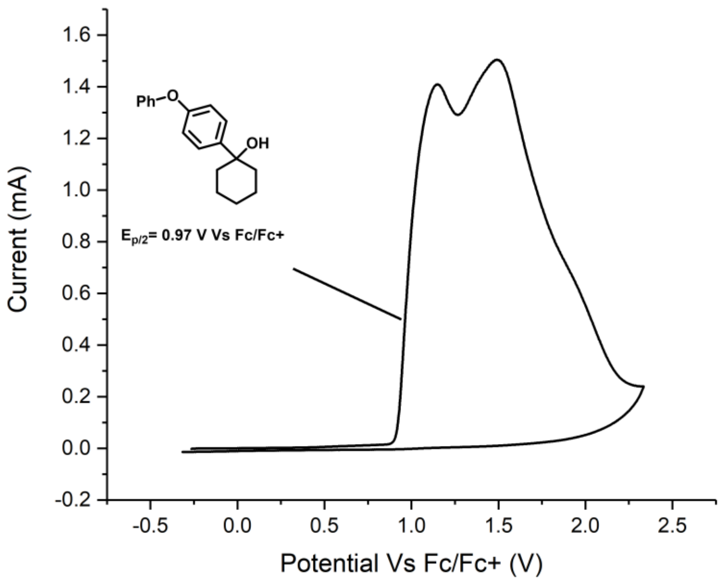

Figure S8 – Cyclic voltammogram of compound (5.0 mM) MeCN (4 mM), $LiClO_4$ (0.1 M).
***Scan rate**: 50 mV/s*

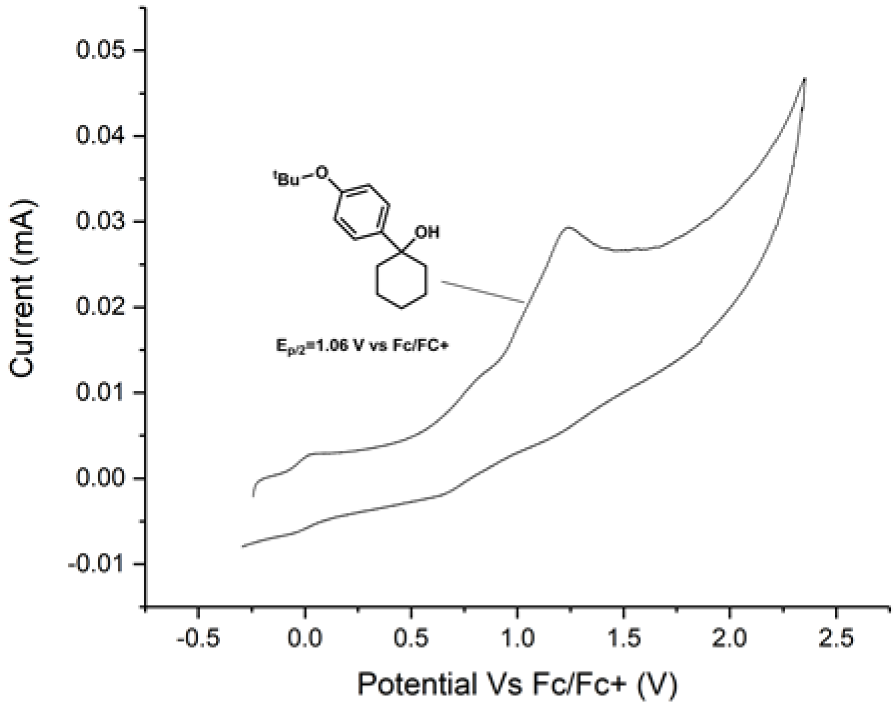

Figure S9 – Cyclic voltammogram of compound (5.0 mM) MeCN (4 mM), $LiClO_4$ (0.1 M).
***Scan rate**: 100 mV/s*

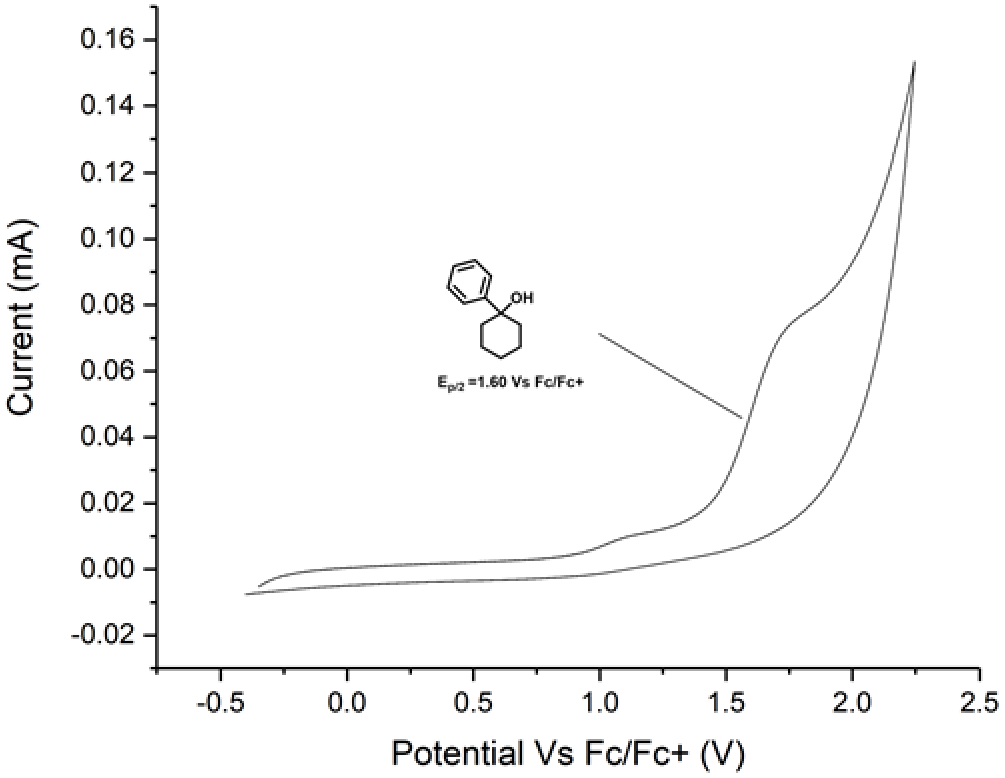

Figure S10 – Cyclic voltammogram of compound (4.0 mM) MeCN (4 mM), $LiClO_4$ (0.1 M).
***Scan rate**: 100 mV/s*

Oxidation Potential ($E_{p/2}$) of substrates – Summary

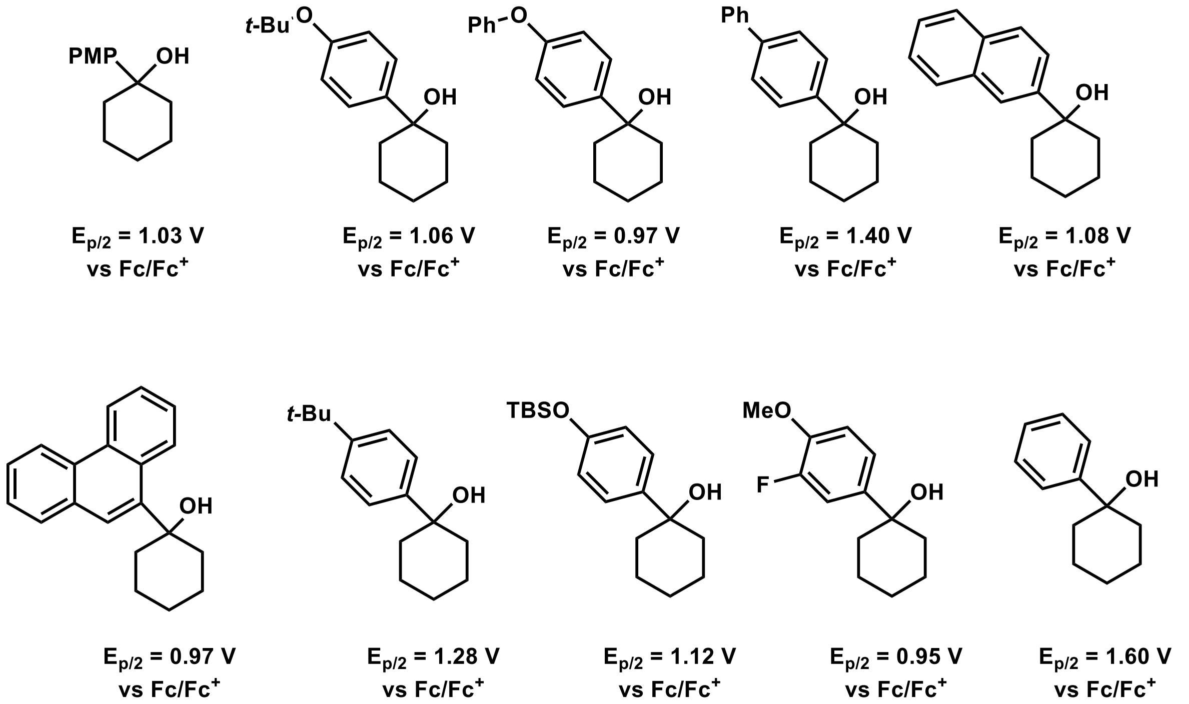

Figure S11 – Summary of the oxidation potential of substrates

CV of Redox Mediators

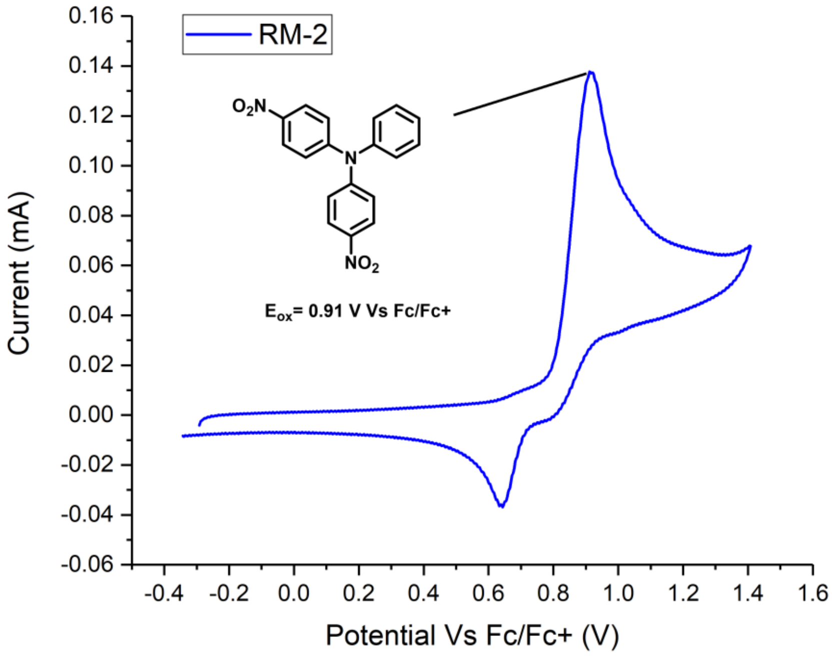

Figure S12 – Cyclic voltammogram of compound (4.0 mM) MeCN (4 mM), $LiClO_4$ (0.1 M).
***Scan rate**: 100 mV/s*

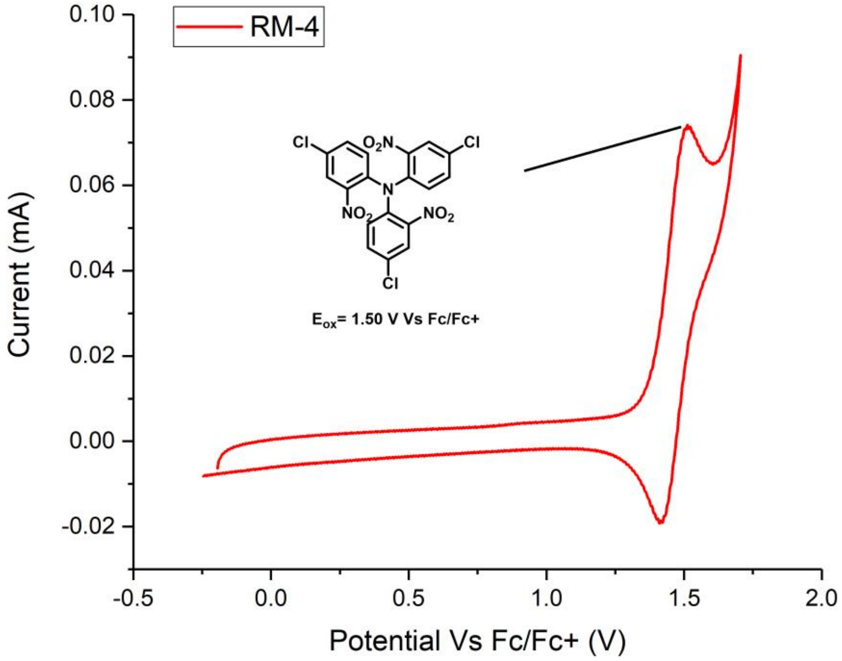

Figure S13 – Cyclic voltammogram of compound (4.0 mM) MeCN (4 mM), $LiClO_4$ (0.1 M).
***Scan rate**: 100 mV/s*

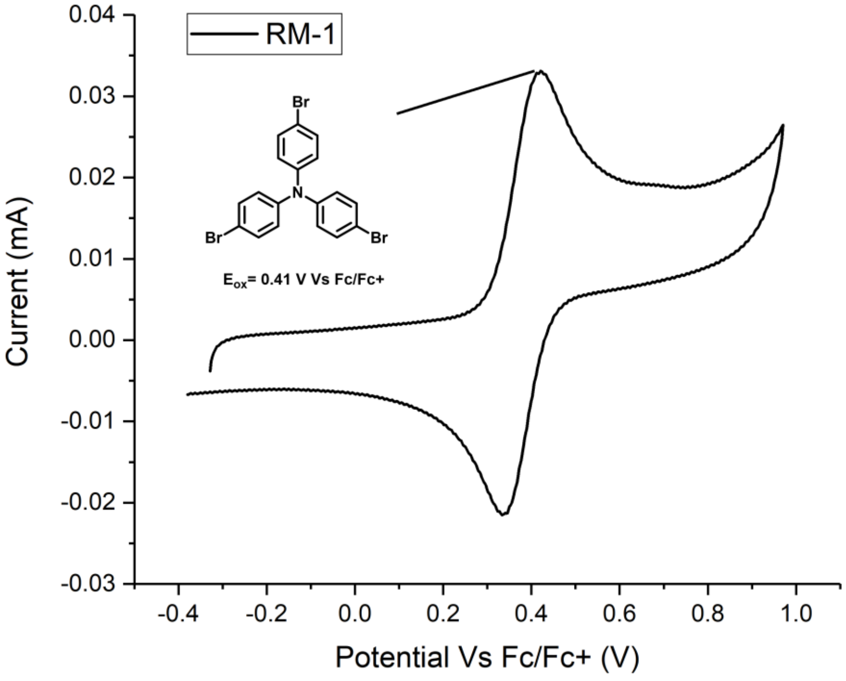

Figure S14 – Cyclic voltammogram of compound (4.0 mM) MeCN (4 mM), $LiClO_4$ (0.1 M).
***Scan rate**: 100 mV/s*

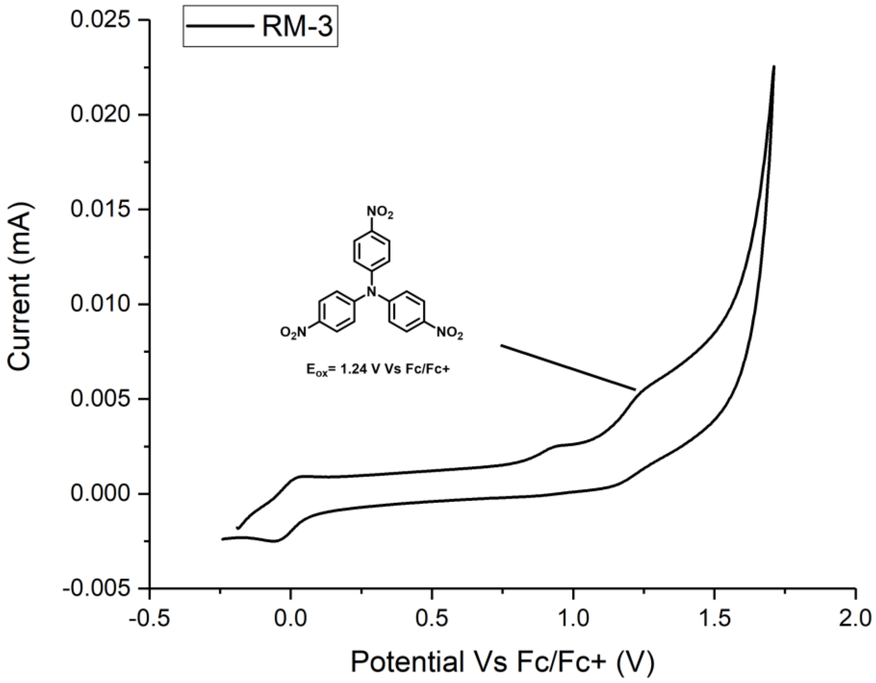

Figure S15 – Cyclic voltammogram of compound (4.0 mM) MeCN (4 mM), LiClO$_4$ (0.1 M).
***Scan rate**: 100 mV/s*

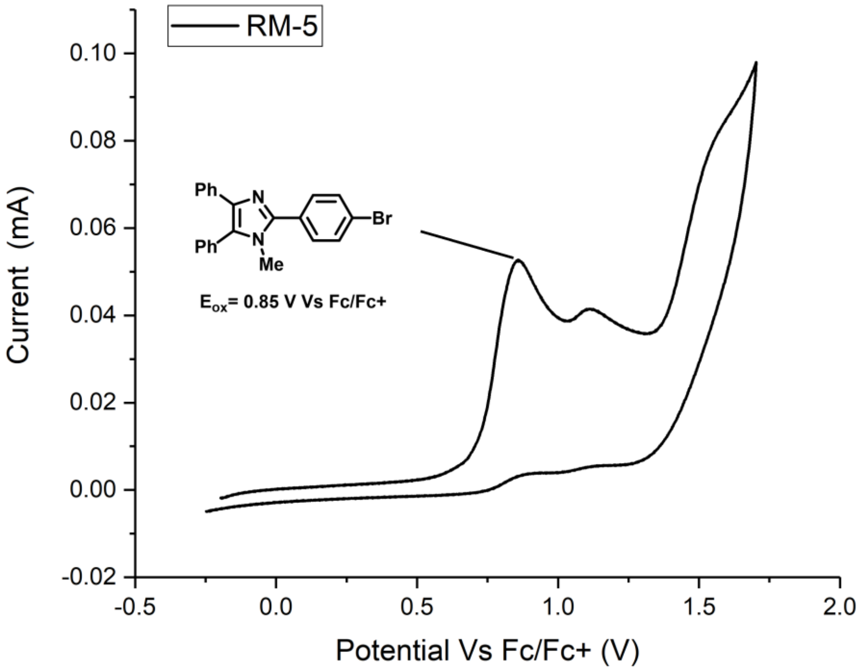

Figure S16 – Cyclic voltammogram of compound (4.0 mM) MeCN (4 mM), LiClO$_4$ (0.1 M).
***Scan rate**: 100 mV/*

4. Optimization Studies

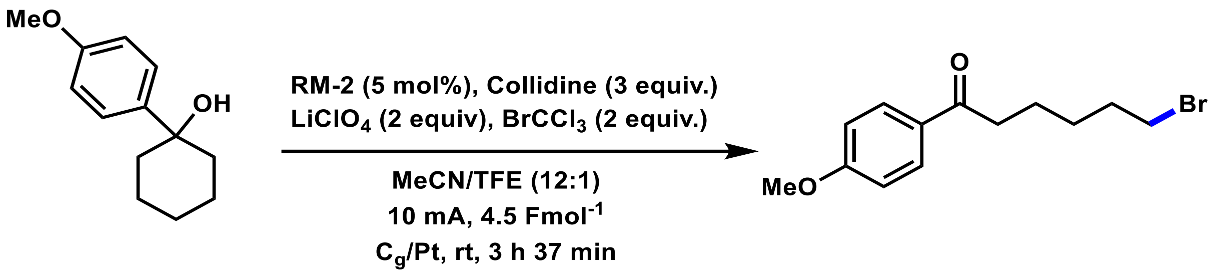

Optimization table

Table S1 – Optimization table

Entry	Variation	Yield %	SM %
**1**	none	98 (94)	<2
**2**	No electricity	<2	95
**3**	No Collidine	<2	20
**4**	1 equiv. of Collidine	65	30
**5**	2 equiv. of Collidine	81	16
**6**	K_2CO_3 instead of Collidine	3	34
**7**	7.5 mA instead of 10 mA	74	14
**8**	12.5 mA instead of 10 mA	98	<2
**9**	20 mA instead of 10mA	92	<2
**10**	2.5 F mol^{-1} instead of 4.5	56	26
**11**	No redox mediator	32	68
**12**	1 mol% redox mediator	50	23
**13**	Nickel plate as cathode	63	32
**14**	Graphite as Cathode	34	49
**15**	1.1 equiv of $BrCCl_3$	49	<2
**16**	HFIP as proton source	49	51
**17**	No proton Source	53	<2
**18**	$TBAPF_6$ as supporting electrolyte	80	20
**19**	DCE as solvent	51	40
**20**	DCM as solvent	74	<2

Yields calculated by ^1H NMR spectroscopy using 1,3,5-trimethoxybenzene as an internal standard. Isolated yield in parathesis.

Redox Mediator Screening

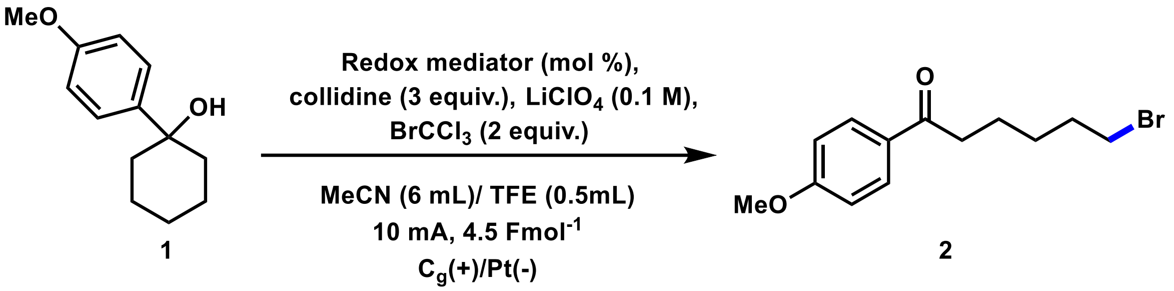

Table S2 – Redox mediator screening

Entry	Redox Mediator (mol %)	1 % (^{1}HNMR)	2 % (^{1}HNMR)
1	1 (5)	98	<2
2	**2 (5)**	**<2**	**98**
3	3 (5) insoluble	N/A	N/A
4	4 (5)	41	32
5	5 (5)	60	<2

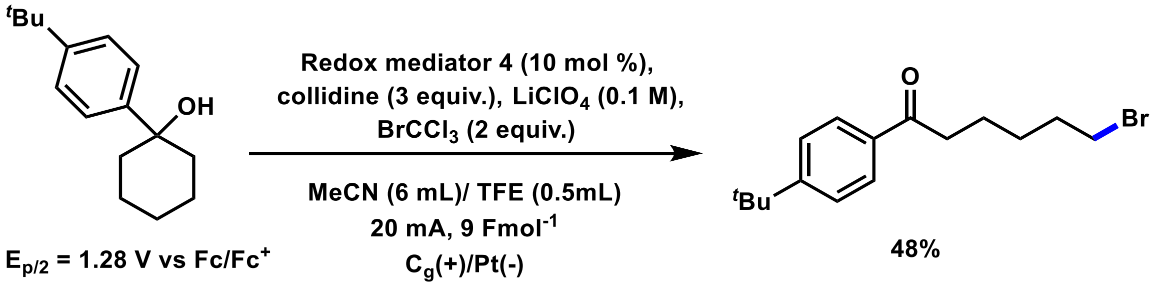

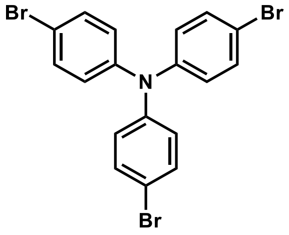

RM-1
E_{ox}= 0.41 V
vs Fc/Fc$^+$

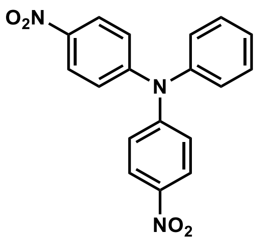

RM-2
E_{ox}= 0.91 V
vs Fc/Fc$^+$

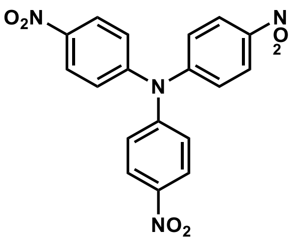

RM-3
E_{ox}= 1.24 V
vs Fc/Fc$^+$

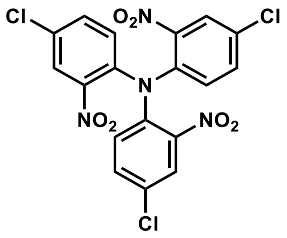

RM-4
E_{ox}= 1.50 V
vs Fc/Fc$^+$

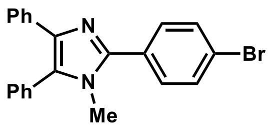

RM-5
E_{ox}= 0.85 V
vs Fc/Fc$^+$

4.1. General Procedure – Electrochemical Deconstructive Functionalization

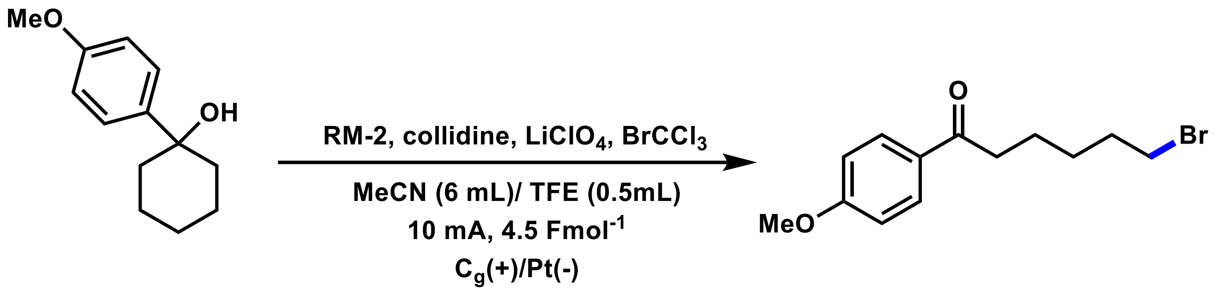

Experimental Procedure: To an oven dried 10 mL Electrasyn vial, was added cycloalkanol (0.30 mmol), redox-mediator (5 mol %), collidine dried over molecular sieves (3.0 equiv.), lithium perchlorate (2.0 equiv.) and the vial was sealed with an Electrasyn cap comprised of a graphite anode and platinum cathode. The vial was back filled with N_2 (three cycles) and anhydrous acetonitrile (6 mL) and trifluoroethanol dried over molecular sieves (0.5 mL) was added under N_2 atmosphere. The vial was then purged with N_2 for 10 minutes and bromotrichloromethane (2.0 equiv.) was added and the reaction mixture was then electrolysed using an Electrasyn 2.0 at a constant current of 10 mA until 4.5 Fmol^{-1} of charge (3.37 hours) was passed at ambient temperature. To the reaction mixture was then added 0.1 mmol of trimethoxybenzene as an internal standard to get the crude NMR yield.

The reaction was quenched with aq. NH_4Cl, washed with aq. $CuSO_4$ solution, and extracted with EtOAc (10 mL x 2). The organic layer was dried over $MgSO_4$, filtered and distilled under reduced pressure. The crude residue obtained was purified flash column chromatography (EtOAc/Petrol, silica gel) to afford the brominated ketones.

Additional Conditions:

B: 10 mA, 5 mol% RM-2, 6 F/mol

C: 10 mA, 10 mol% RM-2, 9F/mol

D: 10 mA, 5 mol% RM-2, 12.4 F/mol

E: 20 mA, 10 mol% RM-4, 9F/mol

F: 12.5 mA, 5 mol% RM-2, 9 F/mol

G: 10 mA, 5 mol% RM-4, 4.5 F/mol

H: 10 mA, 5mol% RM-2, 9 F/mol

4.2. Characterization of Products

6-bromo-1-(4-methoxyphenyl)hexan-1-one (2)

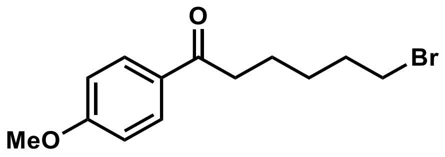

Prepared according to General Procedure (standard conditions) using **1** (62 mg). The crude was purified by flash column chromatography (5→10% EtOAc/Petrol, silica gel) to afford **2** (80.4 mg, 94%) as a low melting solid.

R_f = 0.54 (15% EtOAc/Petrol); **FTIR** (ν_{max} cm^{-1}, thin film) = 2939, 2841, 1675, 1600, 1572, 1509, 1456, 1383, 1345, 1254, 1173, 920, 837, 807; **^{1}H NMR (500 MHz, $CDCl_3$)** δ = 7.94 (d, *J* = 9.0 Hz, 2H), 6.93 (d, *J* = 8.9 Hz, 2H), 3.87 (s, 3H), 3.43 (t, *J* = 6.8 Hz, 2H), 2.97 – 2.89 (m, 2H), 2.01 – 1.87 (m, 2H), 1.80 – 1.70 (m, 2H), 1.59 – 1.49 (m, 2H); **^{13}C NMR (126 MHz, $CDCl_3$)** δ = 198.8, 163.6, 130.4, 130.2, 113.9, 55.6, 38.1, 33.8, 32.8, 28.1, 23.7; **HRMS** (ES^+/Q-TOF) m/z : [M+H] Calcd for [$C_{13}H_{18}O_2Br$] requires 285.0490; Found 285.0484.

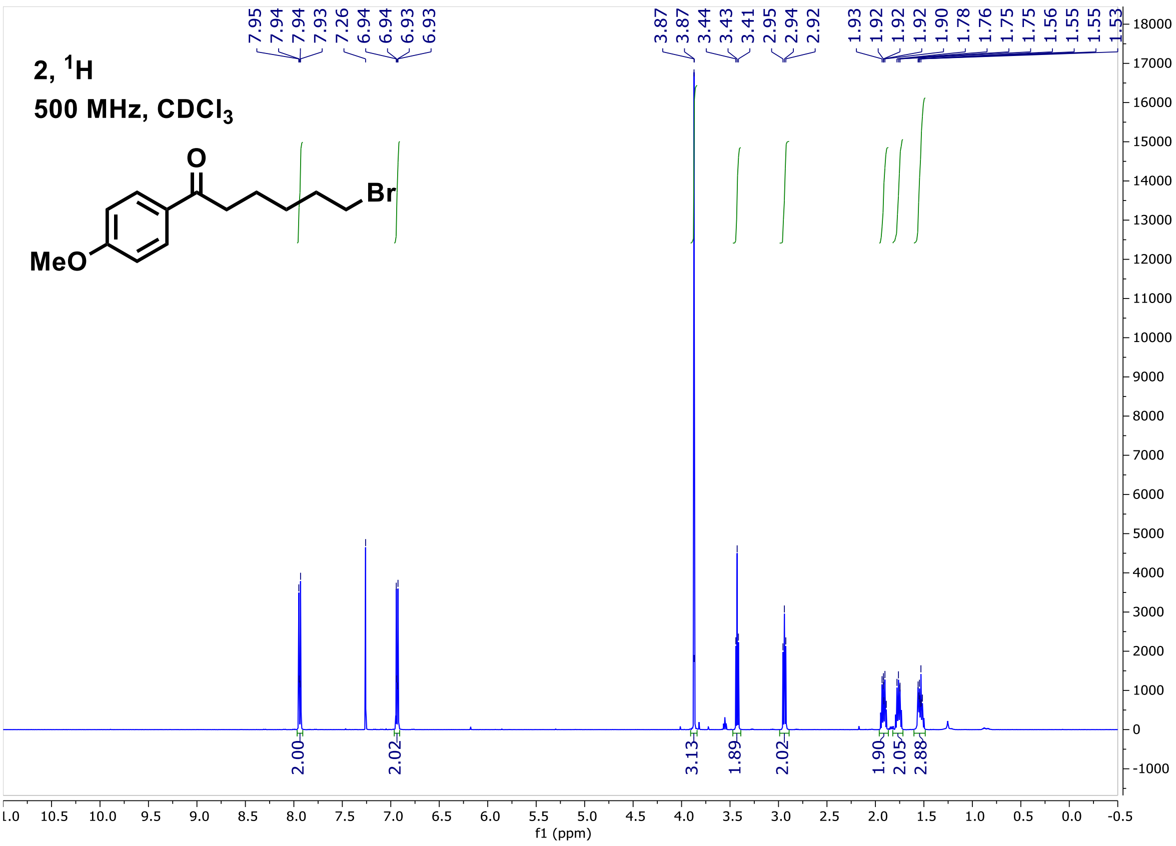
2, ¹H
500 MHz, CDCl3
MeO
O
Br
7.95 7.94 7.94 7.93 7.26 6.94 6.94 6.93 6.93
3.87 3.87 3.44 3.43 3.41 2.95 2.94 2.92
1.93 1.92 1.92 1.92 1.90 1.78 1.76 1.75 1.75 1.56 1.55 1.55 1.53
2.00 2.02 3.13 1.89 2.02 1.90 2.05 2.88
f1 (ppm)

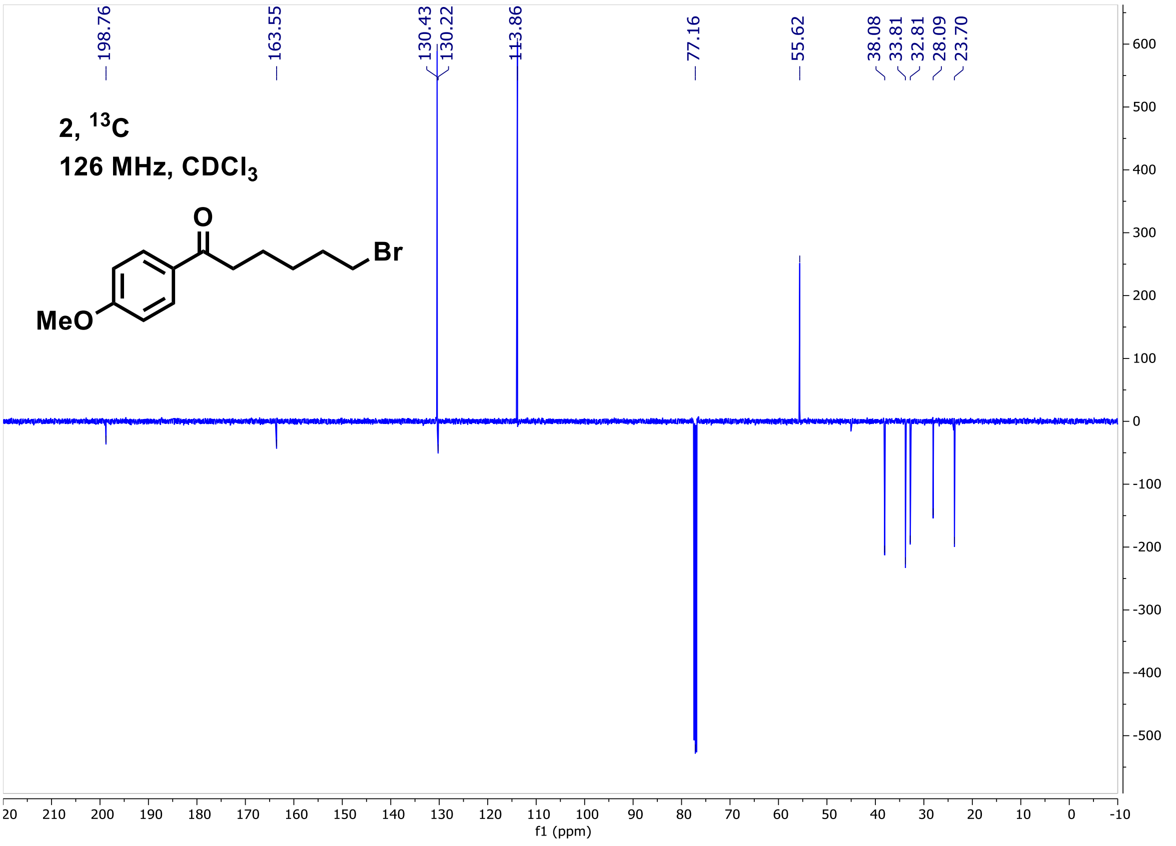
2, ¹³C
126 MHz, CDCl3
MeO
O
Br
198.76 163.55 130.43 130.22 113.86 77.16 55.62 38.08 33.81 32.81 28.09 23.70
f1 (ppm)

3-bromo-1-(4-methoxyphenyl)propan-1-one (3)

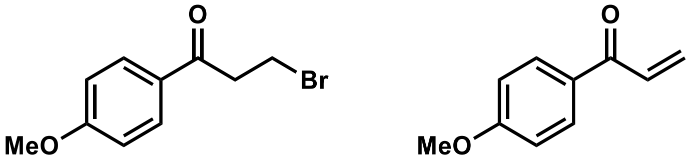

3*

Prepared according to General Procedure (standard conditions) using **S3** (49 mg). The yield 91% was determined by ^{1}HNMR spectroscopy of the crude reaction mixture . Upon column chromatography (2→8% EtOAc/Petrol, silica gel) elimination product (3*) was isolated in 72% as a colourless oil.

R_f = 0.67 (15% EtOAc/Petrol); **^{1}H NMR (300 MHz, $CDCl_3$)** δ = 7.97 (d, *J* = 8.9 Hz, 2H), 7.18 (dd, *J* = 17.1, 10.5 Hz, 1H), 6.96 (d, *J* = 8.9 Hz, 2H), 6.43 (dd, *J* = 17.1, 1.8 Hz, 1H), 5.88 (dd, *J* = 10.5, 1.8 Hz, 1H), 3.88 (s, 3H); **^{13}C NMR (126 MHz, $CDCl_3$)** δ = 189.4, 163.7, 132.3, 131.2, 130.2, 129.4, 114.0, 55.6.

These data are consistent with those previously reported in the literature.[14]

Crude NMR

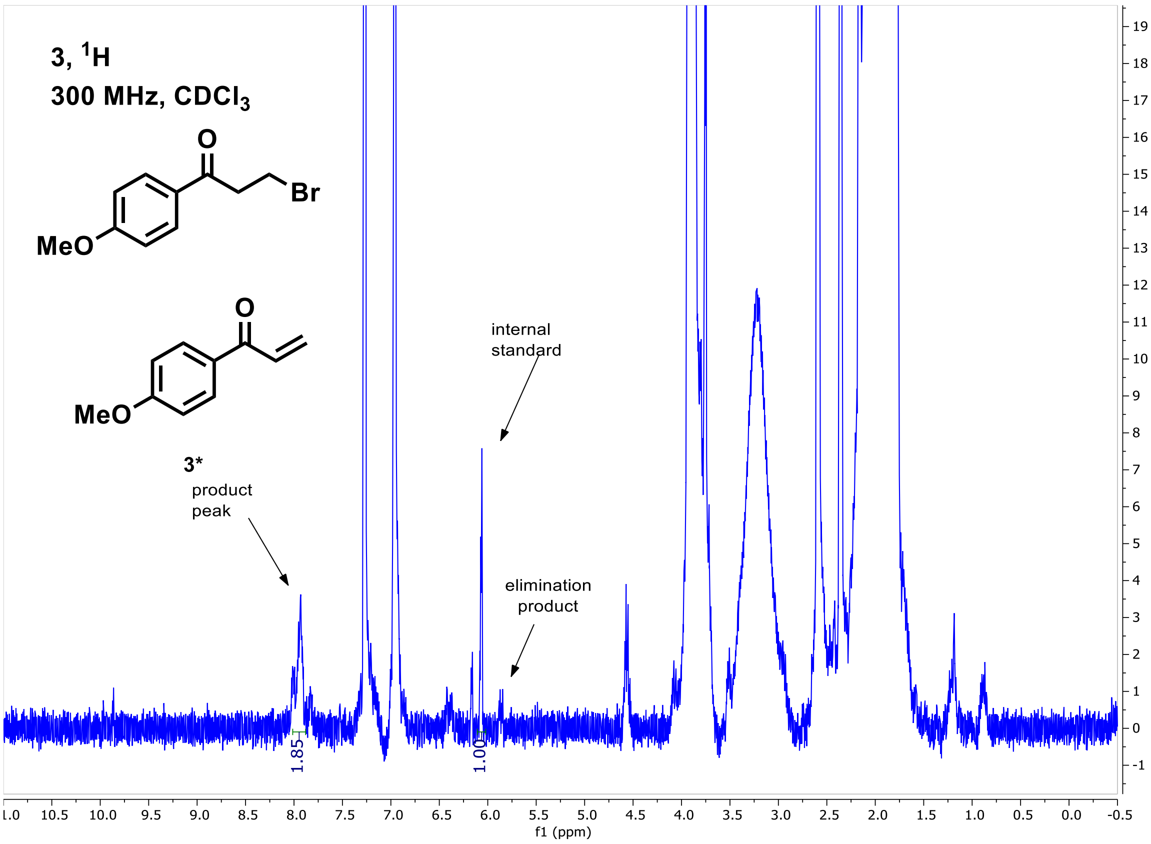
3, 1H
300 MHz, CDCl3
O
Br
MeO
O
MeO
3*
product
peak
internal
standard
elimination
product
1.85
1.00
f1 (ppm)

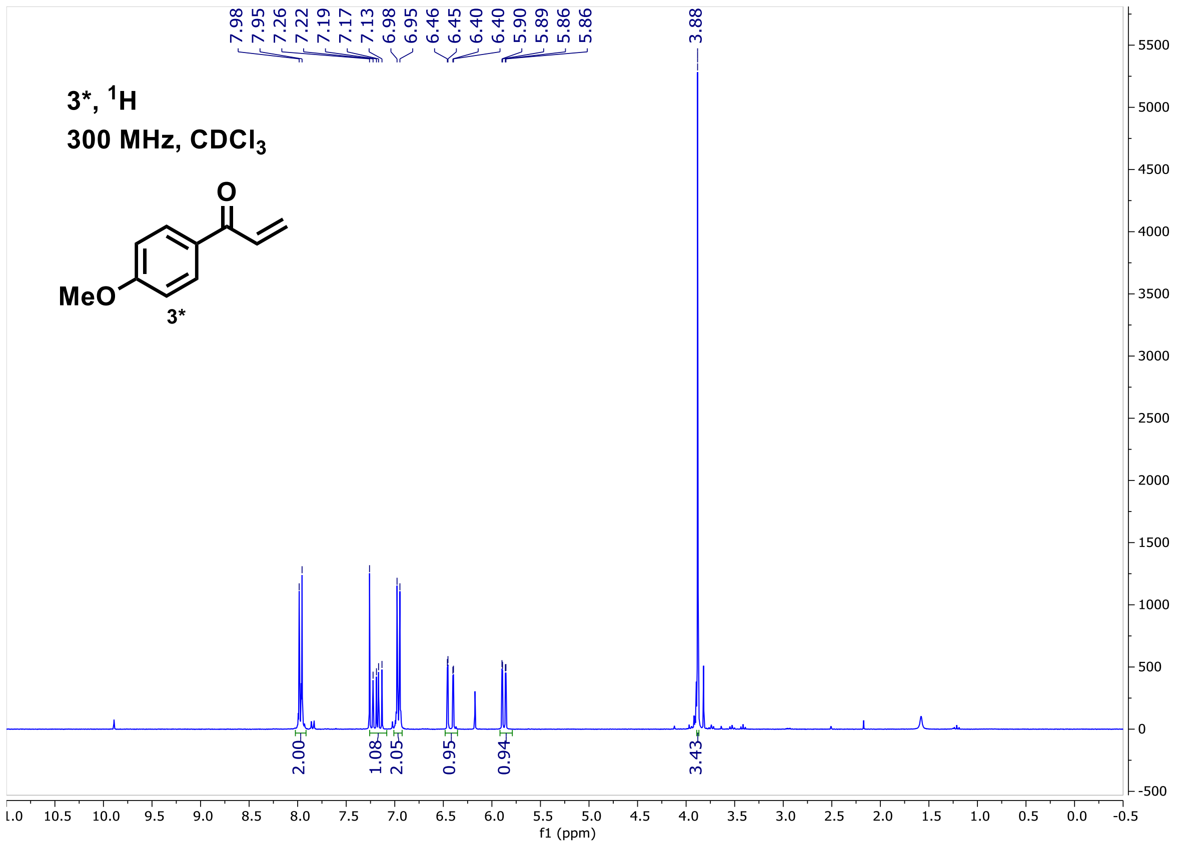

3*, 1H
300 MHz, CDCl3
MeO
3*
7.98
7.95
7.26
7.22
7.19
7.17
7.13
6.98
6.95
6.46
6.45
6.40
6.40
5.90
5.89
5.86
5.86
3.88
2.00
1.08
2.05
0.95
0.94
3.43
f1 (ppm)

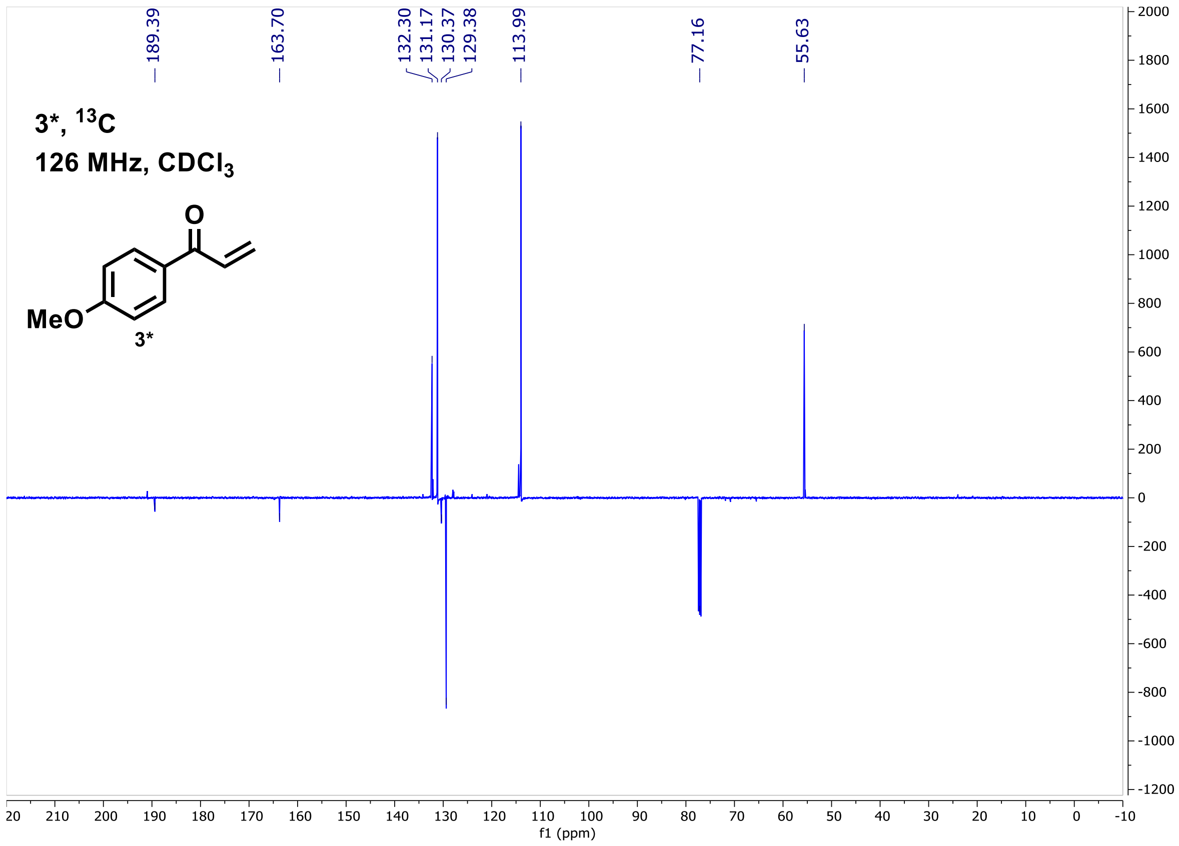

3*, 13C
126 MHz, CDCl3
MeO
3*
189.39
163.70
132.30
131.17
130.37
129.38
113.99
77.16
55.63
f1 (ppm)

4-bromo-1-(4-methoxyphenyl)butan-1-one (4)

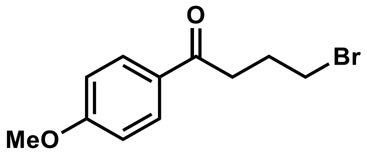

Prepared according to General Procedure (standard conditions) using **S4** (54 mg). The crude was purified by flash column chromatography (5→10% EtOAc/Petrol, silica gel) to afford **4** (65 mg, 83%) as a colourless oil.

R_f = 0.48 (15% EtOAc/Petrol); **^{1}H NMR (500 MHz, $CDCl_3$)** δ = 7.97 (d, *J* = 9.0 Hz, 2H), 6.94 (d, *J* = 9.0 Hz, 2H), 3.88 (s, 3H), 3.55 (t, *J* = 6.3 Hz, 2H), 3.13 (t, *J* = 7.0 Hz, 2H), 2.34 – 2.26 (m, 2H); **^{13}C NMR (126 MHz, $CDCl_3$)** δ = 197.5, 163.7, 130.4, 130.0, 114.0, 55.6, 36.3, 34.0, 27.1.

These data are consistent with those previously reported in the literature.[15]

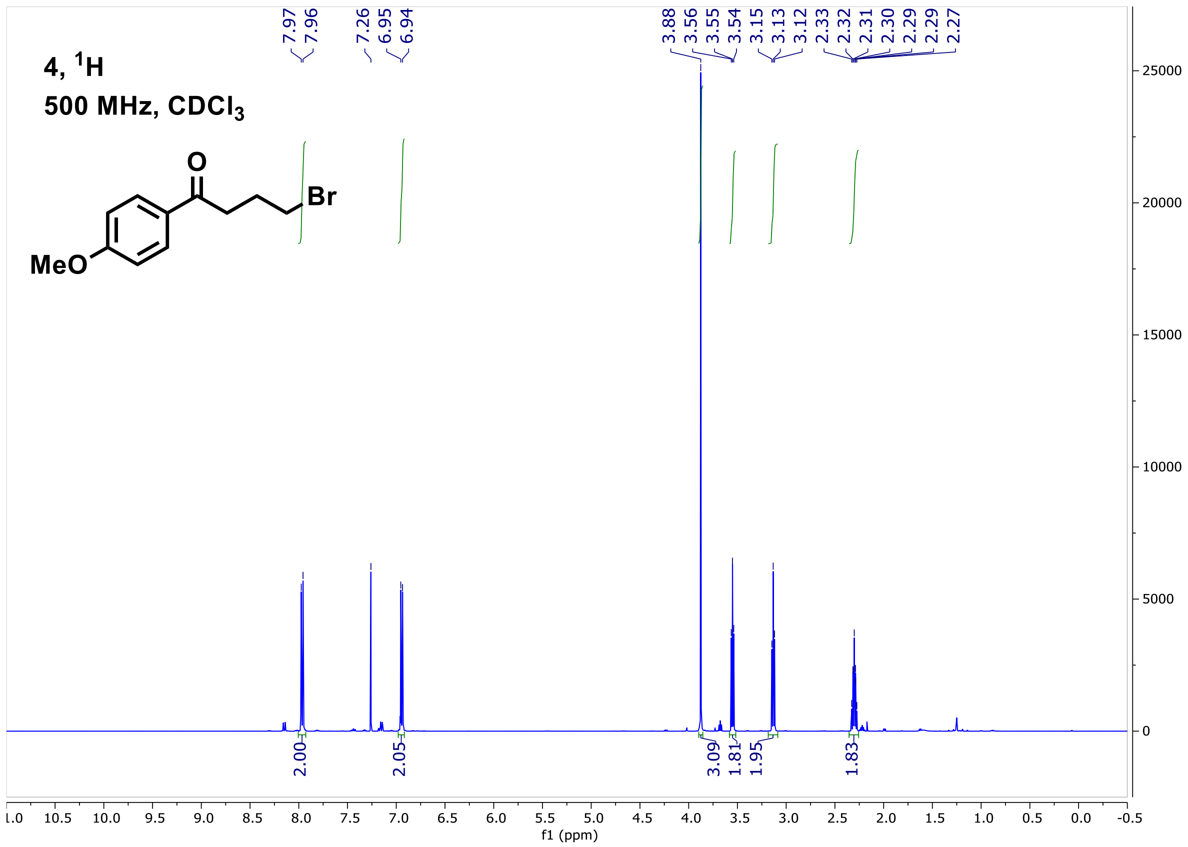
4, 1H
500 MHz, CDCl3
O
Br
MeO
7.97
7.96
7.26
6.95
6.94
3.88
3.56
3.55
3.54
3.15
3.13
3.12
2.33
2.32
2.31
2.30
2.29
2.29
2.27
2.00
2.05
3.09
1.81
1.95
1.83
f1 (ppm)

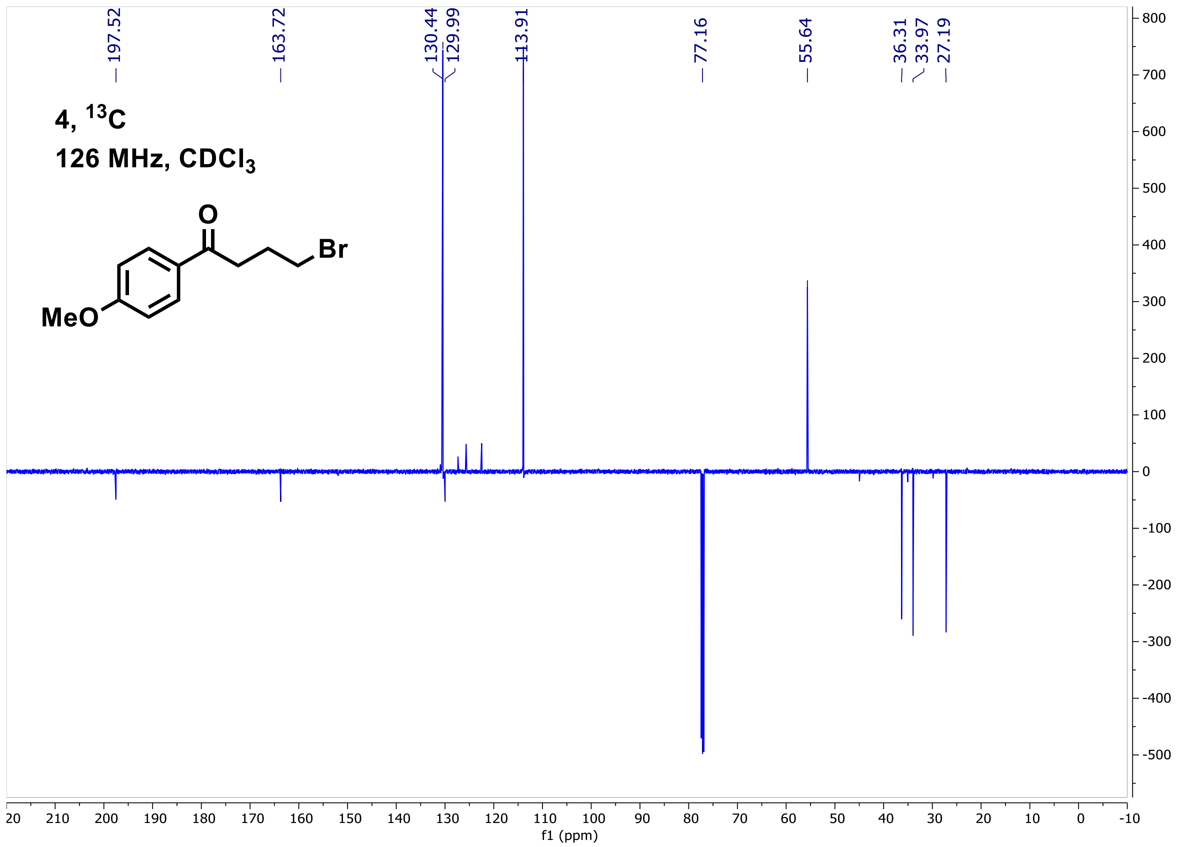
4, 13C
126 MHz, CDCl3
O
Br
MeO
197.52
163.72
130.44
129.99
113.91
77.16
55.64
36.31
33.97
27.19
f1 (ppm)

5-bromo-1-(4-methoxyphenyl)pentan-1-one (5)

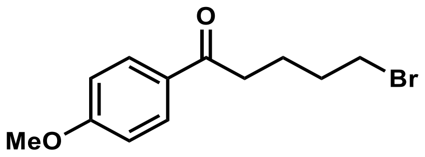

Prepared according to General Procedure (standard conditions) using **S5** (58 mg). The crude was purified by flash column chromatography (5→10% EtOAc/Petrol, silica gel) to afford **5** (68 mg, 82%) as a colourless oil.

R_f = 0.41 (15% EtOAc/Petrol); **^{1}H NMR (500 MHz, $CDCl_3$)** δ = 7.94 (d, *J* = 9.0 Hz, 2H), 6.94 (d, *J* = 8.9 Hz, 2H), 3.87 (s, 3H), 3.45 (t, *J* = 6.6 Hz, 2H), 2.96 (t, *J* = 7.0 Hz, 2H), 2.17 – 1.68 (m, 4H); **^{13}C NMR (126 MHz, $CDCl_3$)** δ = 198.3, 163.6, 130.4, 130.1, 113.9, 55.6, 37.2, 33.6, 32.4, 23.1

These data are consistent with those previously reported in the literature.[8]

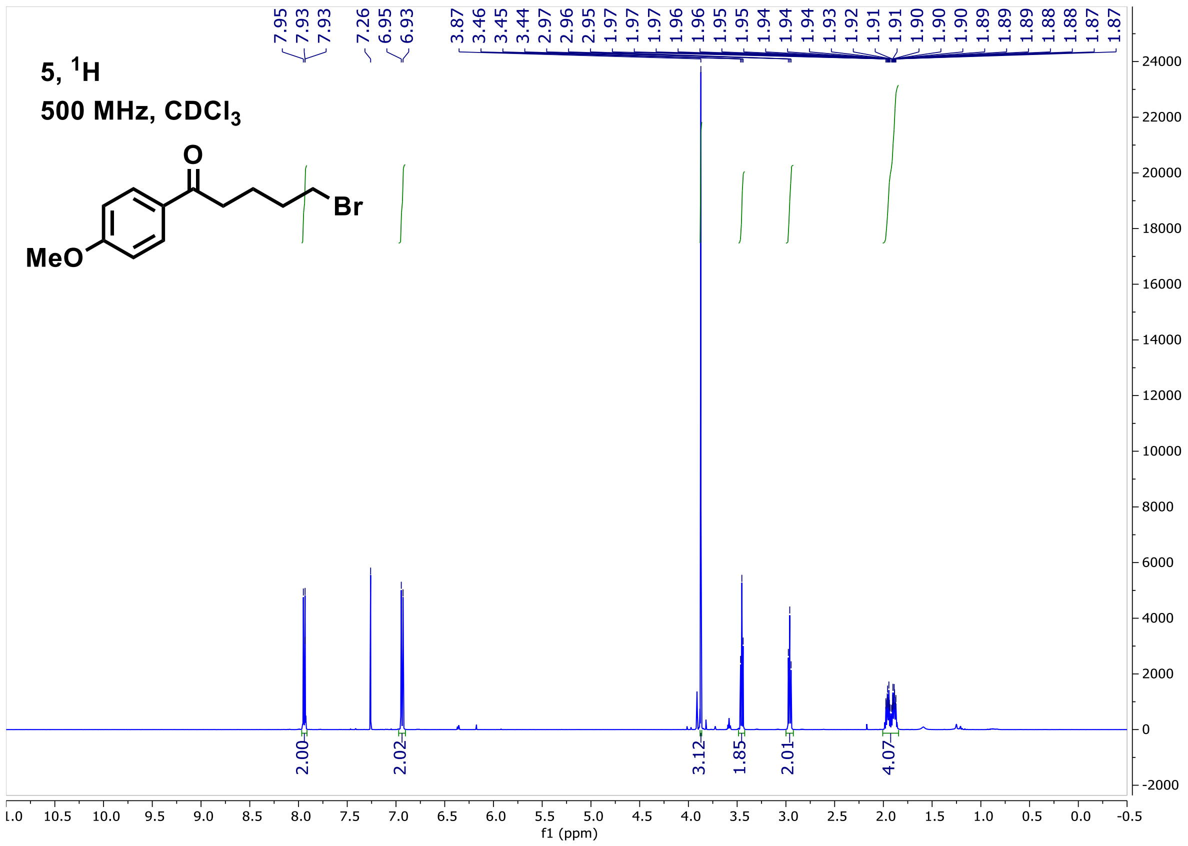
5, 1H
500 MHz, CDCl3
MeO
O
Br
f1 (ppm)

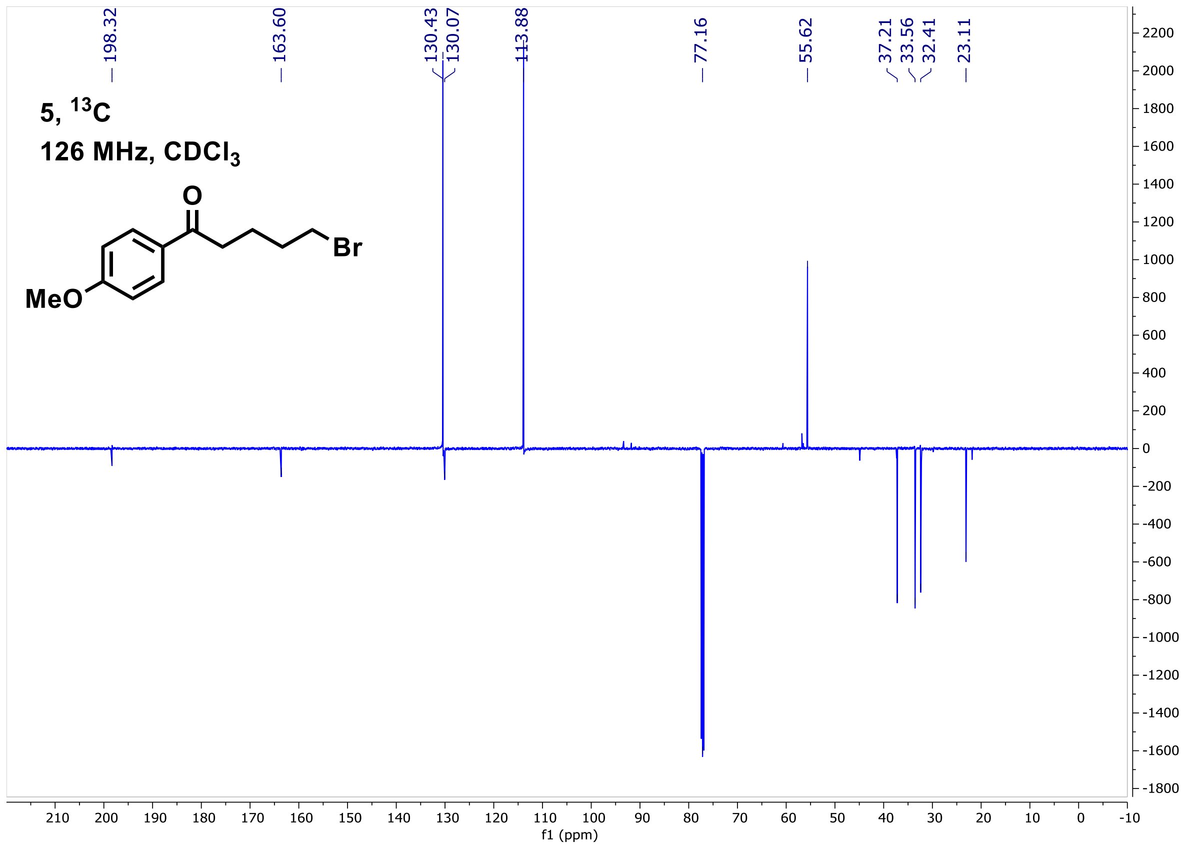
5, 13C
126 MHz, CDCl3
MeO
O
Br
f1 (ppm)

7-bromo-1-(4-methoxyphenyl)heptan-1-one (6)

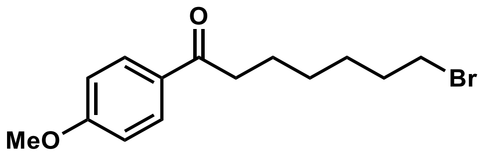

Prepared according to General Procedure (standard conditions) using **S6** (66 mg). The crude was purified by flash column chromatography (5→10% EtOAc/Petrol, silica gel) to afford **6** (57 mg, 64%) as a colourless oil.

R_f = 0.59 (15% EtOAc/Petrol); **FTIR** (ν_{max} cm^{-1}, thin film) = 2937, 2848, 1676, 1602, 1574, 1512, 1465, 1417, 1310, 1255, 1216, 1170, 1112, 1026, 830; **^1H NMR (500 MHz, $CDCl_3$)** δ = 7.94 (d, *J* = 9.0 Hz, 2H), 6.93 (d, *J* = 9.0 Hz, 2H), 3.87 (s, 3H), 3.41 (t, *J* = 6.8 Hz, 2H), 2.96 – 2.88 (m, 2H), 1.95 – 1.82 (m, 2H), 1.79 – 1.70 (m, 2H), 1.53 – 1.35 (m, 4H); **^{13}C NMR (126 MHz, $CDCl_3$)** δ = 199.1, 163.5, 130.5, 130.3, 113.8, 55.6, 38.2, 34.1, 32.7, 28.6, 28.2, 24.4; **HRMS** (ES^+/Q-TOF) m/z : [M+H] Calcd for [$C_{14}H_{20}O_2Br$] requires 299.0647; Found 299.0644.

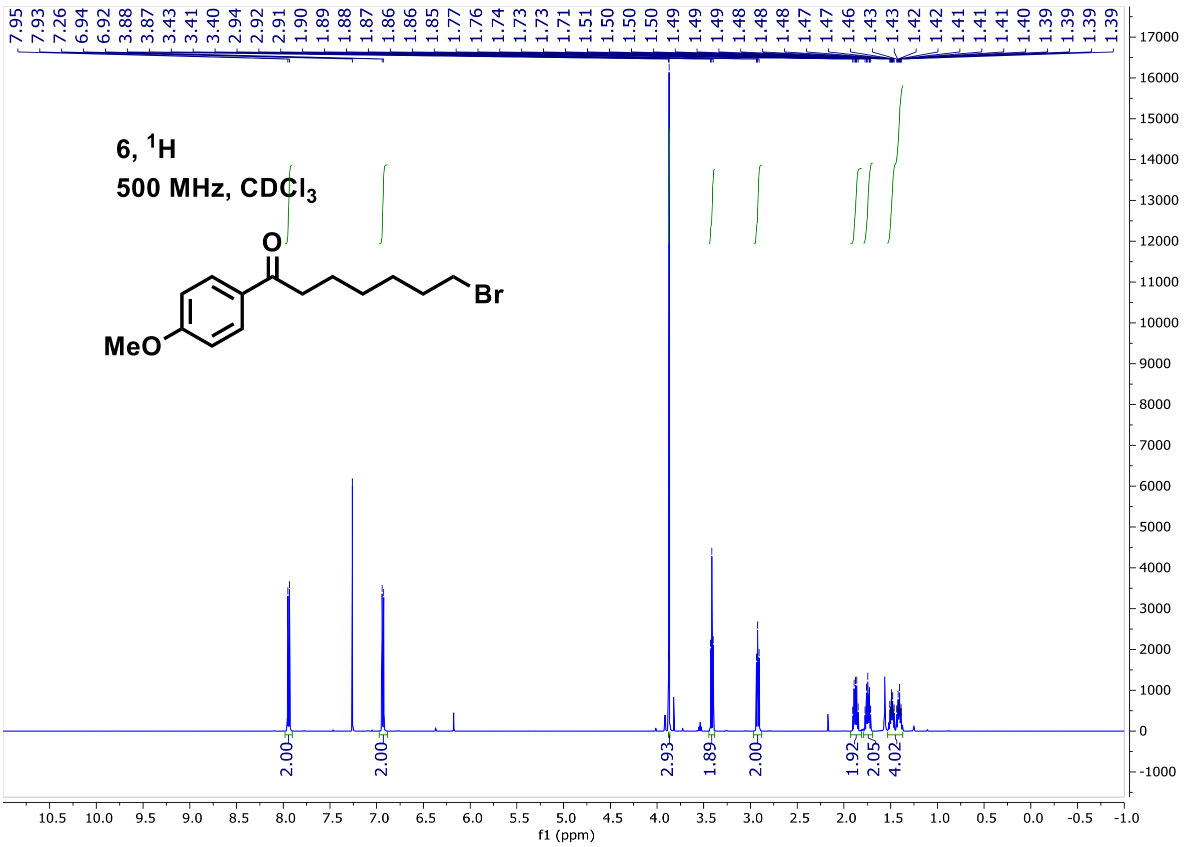
6, 1H
500 MHz, CDCl3
O
Br
MeO
7.95
7.93
7.26
6.94
6.92
3.88
3.87
3.43
3.41
3.40
2.94
2.92
2.91
1.90
1.89
1.88
1.87
1.86
1.86
1.85
1.77
1.76
1.74
1.73
1.73
1.71
1.51
1.50
1.50
1.50
1.49
1.49
1.49
1.48
1.48
1.48
1.47
1.47
1.46
1.43
1.43
1.42
1.42
1.41
1.41
1.41
1.40
1.39
1.39
1.39
2.00
2.00
2.93
1.89
2.00
1.92
2.05
4.02
f1 (ppm)

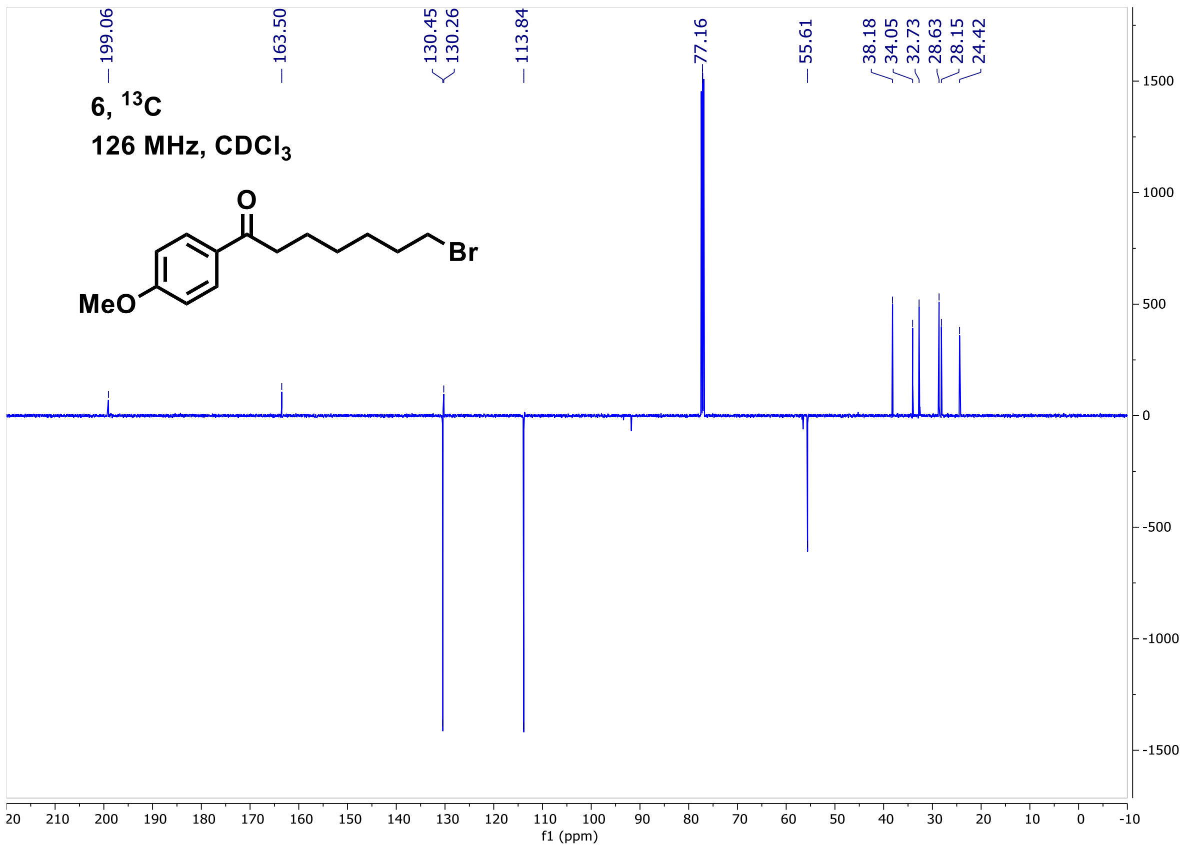
6, 13C
126 MHz, CDCl3
O
Br
MeO
199.06
163.50
130.45
130.26
113.84
77.16
55.61
38.18
34.05
32.73
28.63
28.15
24.42
f1 (ppm)

8-bromo-1-(4-methoxyphenyl)octan-1-one (7)

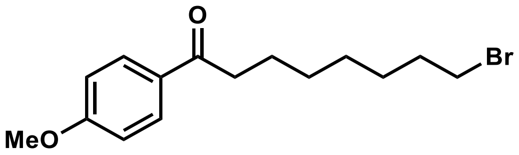

Prepared according to General Procedure (condition B) using **S7** (70 mg). The crude was purified by flash column chromatography (5→10 % EtOAc/Petrol, silica gel) to afford **7** (54 mg, 57%) as a colourless oil.

R_f = 0.55 (15% EtOAc/Petrol); **FTIR** (ν_{max} cm^{-1}, thin film) = 2935, 2848, 1676, 1595, 1510, 1459, 1421, 1310, 1257, 1231, 1168, 1129, 1030, 841, 805; **^1H NMR (500 MHz, $CDCl_3$)** δ = 7.94 (d, *J* = 8.9 Hz, 2H), 6.93 (d, *J* = 8.9 Hz, 2H), 3.87 (s, 3H), 3.40 (t, *J* = 6.8 Hz, 2H), 2.91 (t, *J* = 7.4 Hz, 2H), 1.94 – 1.82 (m, 2H), 1.77 – 1.69 (m, 2H), 1.50 – 1.28 (m, 6H); **^{13}C NMR (126 MHz, $CDCl_3$)** δ = 199.2, 163.5, 130.5, 130.3, 113.8, 55.6, 38.3, 34.1, 32.9, 29.3, 28.9, 28.2, 24.6; **HRMS** (ES^+/Q-TOF) m/z : [M+H] Calcd for [$C_{15}H_{22}O_2Br$] requires 313.0803; Found 313.0790.

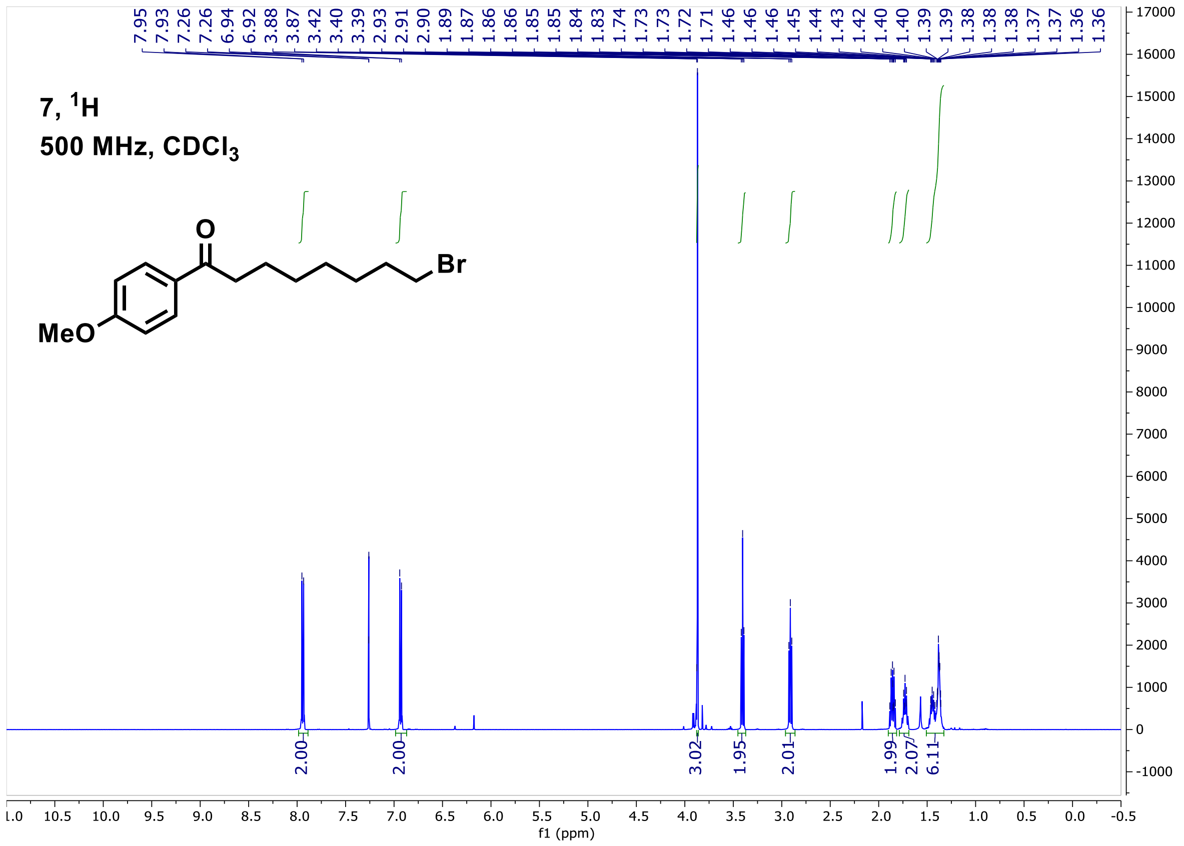
7, ¹H
500 MHz, CDCl3
MeO
O
Br
f1 (ppm)

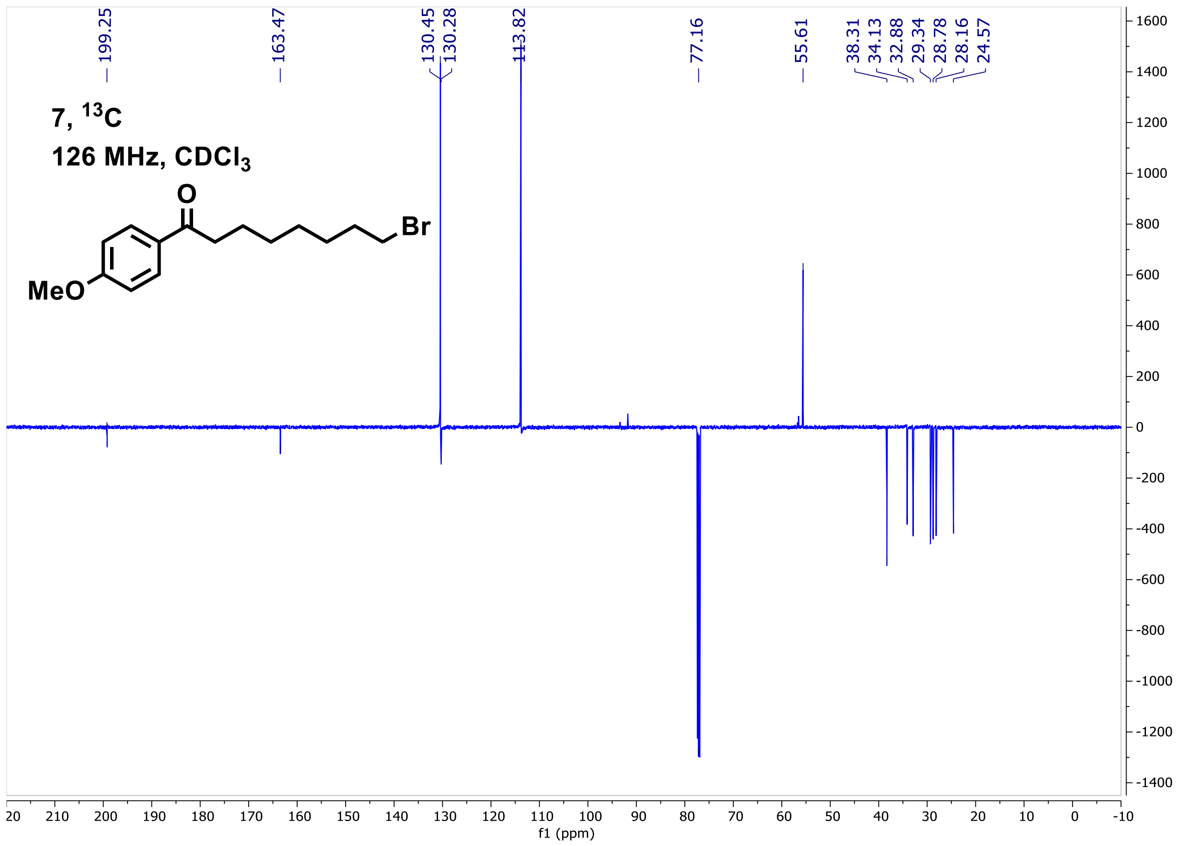
7, ¹³C
126 MHz, CDCl3
MeO
O
Br
f1 (ppm)

12-bromo-1-(4-methoxyphenyl)dodecan-1-one (8)

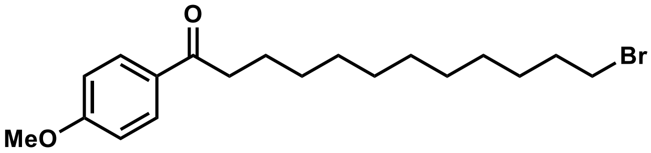

Prepared according to General Procedure (standard conditions) using **S8** (87 mg). The crude was purified by flash column chromatography (5→10% EtOAc/Petrol, silica gel) to afford **8** (105 mg, 95%) as a colourless oil.

R_f = 0.58 (15% EtOAc/Petrol); **FTIR** (ν_{max} cm^{-1}, thin film): 2915, 2850, 1680, 1606, 1510, 1472, 1263, 1168, 1114, 1031, 971, 832; **^1H NMR (500 MHz, $CDCl_3$)** δ = 7.94 (d, *J* = 8.9 Hz, 2H), 6.93 (d, *J* = 8.9 Hz, 2H), 3.87 (s, 3H), 3.41 (t, *J* = 6.9 Hz, 2H), 3.04 – 2.80 (m, 2H), 1.88 – 1.82 (m, 2H), 1.74 – 1.67 (m, 2H), 1.44 – 1.23 (m, 14H); **^{13}C NMR (126 MHz, $CDCl_3$)** δ = 199.4, 163.4, 130.5, 130.3, 113.8, 55.6, 38.5, 34.3, 33.0, 29.6 (5C), 28.9, 28.3, 24.8; **HRMS** (EC^+/Q-TOF) m/z : [M] Calcd for [$C_{19}H_{29}O_2Br$] requires 368.1351; Found 368.1341.

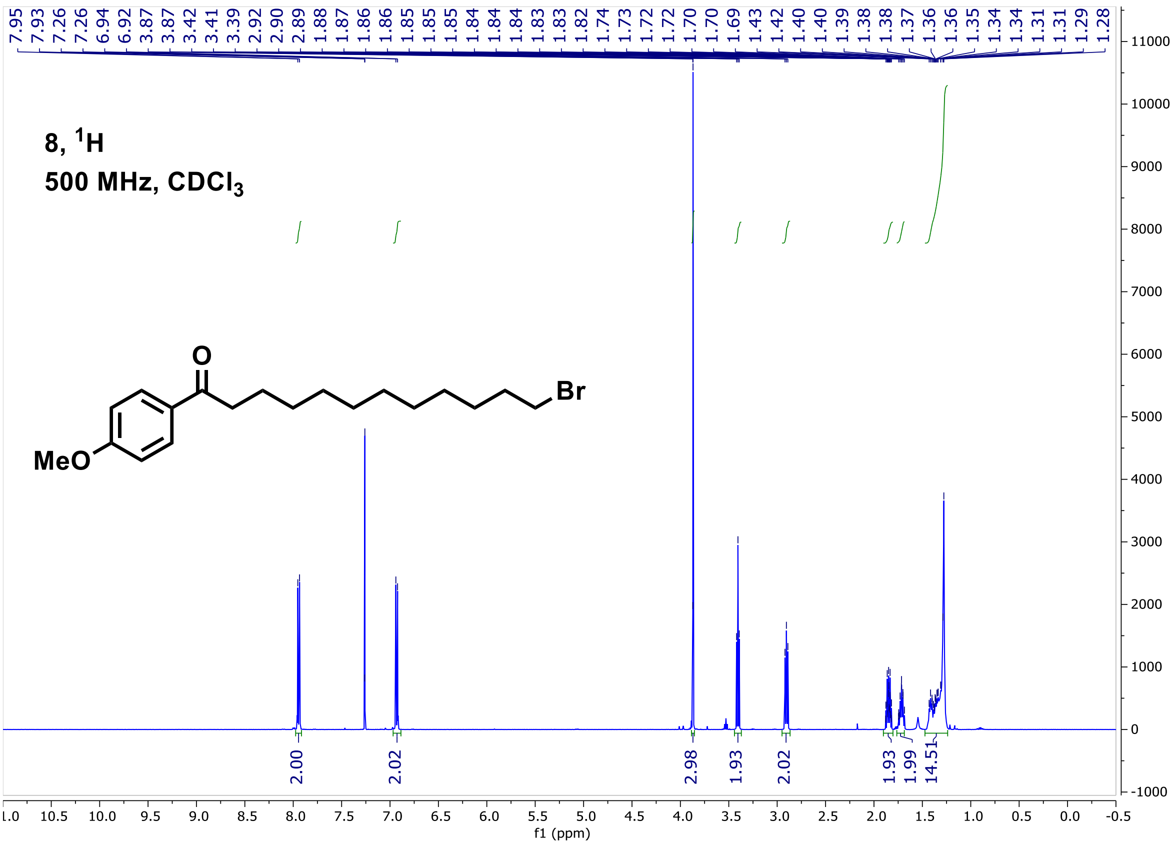
8, 1H
500 MHz, CDCl3
MeO
O
Br
f1 (ppm)

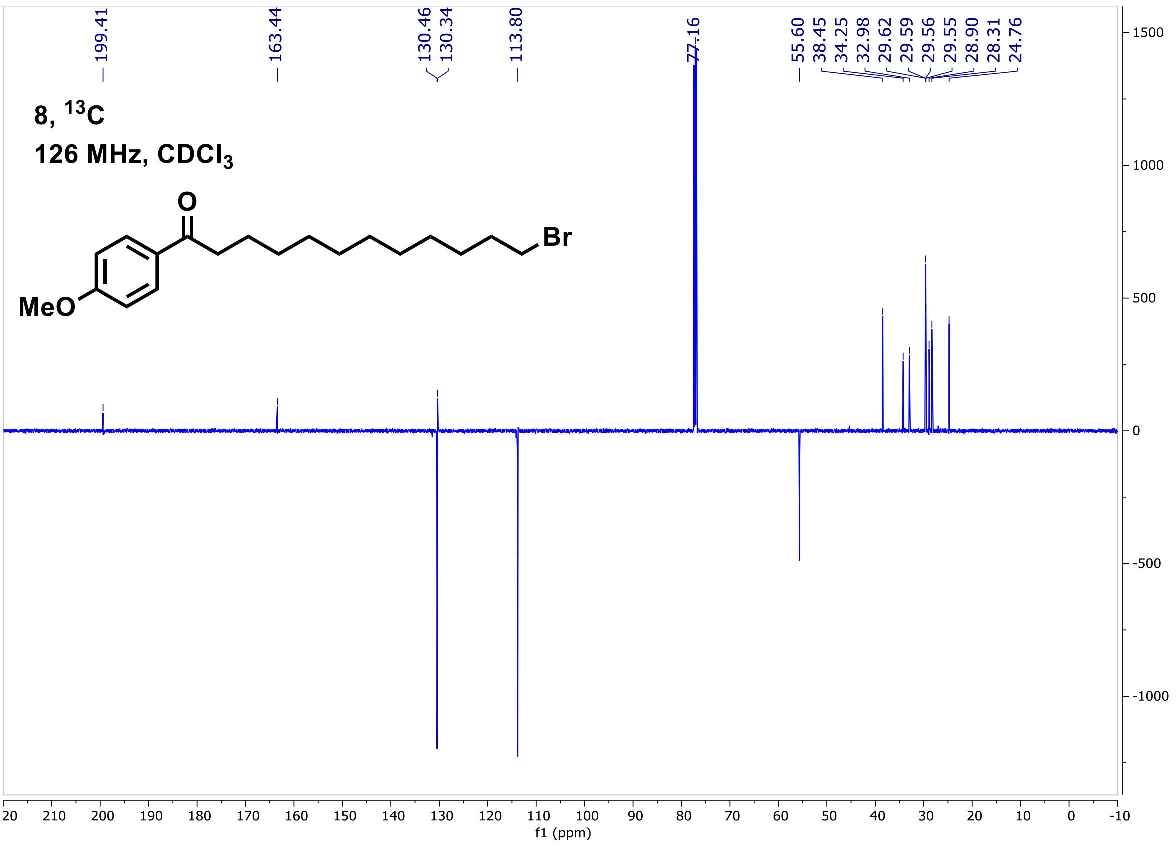
8, 13C
126 MHz, CDCl3
MeO
O
Br
f1 (ppm)

6-bromo-1-(4-(tert-butoxy)phenyl)hexan-1-one (9)

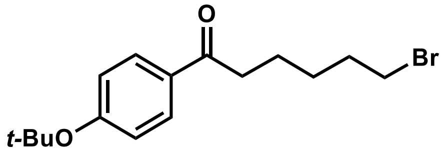

Prepared according to General Procedure (condition C) A using **S9** (74 mg, 0.3 mmol). The crude residue was purified by flash column chromatography (2→8% EtOAc/Petrol, silica gel) to afford **9** (59 mg, 60%) as a brown oil.

R$_f$ = 0.23 (10% EtOAc/Petrol); **FTIR** (ν_{max} cm^{-1}, thin film); 2976, 2935, 2866, 1676, 1595, 1571, 1504, 1456, 1244, 1205, 1153, 1124**; ^{1}H NMR (500 MHz, CDCl$_3$)** δ = 7.93 – 7.85 (m, 2H), 7.08 – 6.97 (m, 2H), 3.43 (t, *J* = 6.8 Hz, 2H), 2.99 – 2.90 (m, 2H), 1.97 – 1.87 (m, 2H), 1.81 – 1.70 (m, 2H), 1.57 – 1.45 (m, 2H), 1.41 (s, 9H); **^{13}C NMR (126 MHz, CDCl$_3$)** δ = 199.1, 160.4, 131.7, 129.6, 122.6, 79.8, 38.2, 33.8, 32.8, 29.1, 28.1 23.6; **HRMS** (ES$^+$/Q-TOF) m/z : [M] Calcd for [$C_{16}H_{24}O_2{}^{81}Br$] requires 329.0939; Found 329.0949.

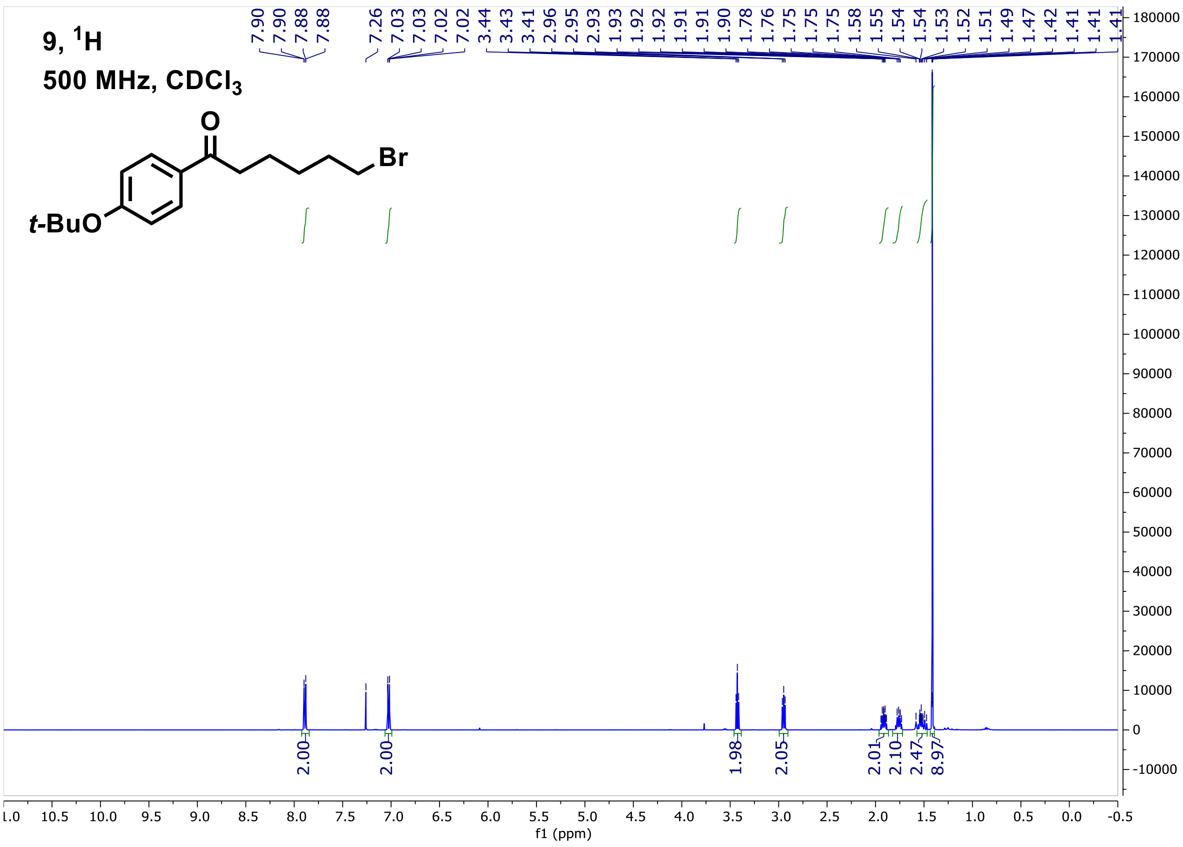

9, ^{1}H
500 MHz, CDCl$_3$
O
Br
t-BuO
7.90 7.90 7.88 7.88 7.26 7.03 7.03 7.02 7.02 3.44 3.43 3.41 2.96 2.95 2.93 1.93 1.92 1.92 1.91 1.91 1.90 1.78 1.76 1.75 1.75 1.75 1.58 1.55 1.54 1.54 1.53 1.52 1.51 1.49 1.47 1.42 1.41 1.41 1.41
2.00 2.00 1.98 2.05 2.01 2.10 2.47 8.97
f1 (ppm)

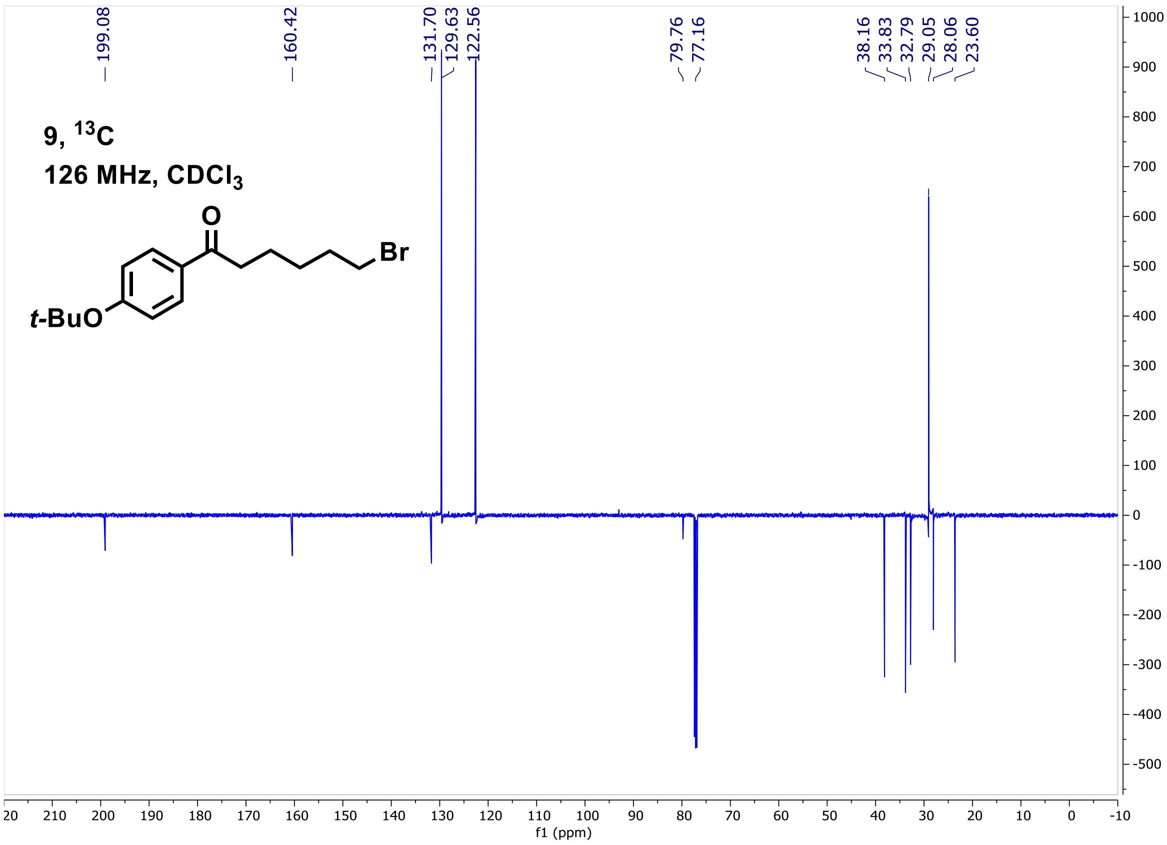

199.08 160.42 131.70 129.63 122.56 79.76 77.16 38.16 33.83 32.79 29.05 28.06 23.60
9, ^{13}C
126 MHz, CDCl$_3$
O
Br
t-BuO
f1 (ppm)

6-bromo-1-(4-phenoxyphenyl)hexan-1-one (10)

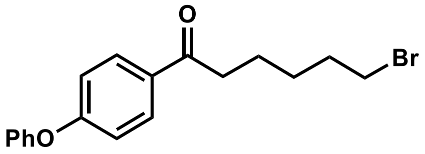

Prepared according to General Procedure (condition D) using **S10** (81 mg, 0.3 mmol). The crude residue was purified by flash column chromatography (2→5% EtOAc/Petrol, silica gel) to afford **10** (49 mg, 47%) as a brown oil.

R_f = 0.27 (10% EtOAc/Petrol); **FTIR** (ν_{max} cm^{-1}, thin film); 3231, 2932, 2835, 2312, 1909, 1676, 1597, 1369, 1305, 1263, 1184, 1130, 1026.; **^{1}H NMR (500 MHz, $CDCl_3$)** δ = 7.98 – 7.91 (m, 2H), 7.43 – 7.36 (m, 2H), 7.23 – 7.17 (m, 1H), 7.09 – 7.04 (m, 2H), 7.03 – 6.97 (m, 2H), 3.43 (t, *J* = 6.8 Hz, 2H), 2.95 (t, *J* = 7.3 Hz, 2H), 2.01 – 1.86 (m, 2H), 1.82 – 1.69 (m, 2H), 1.62 – 1.42 (m, 2H). **^{13}C NMR (126 MHz, $CDCl_3$)** δ = 198.7, 162.0, 155.7, 131.8, 130.4, 130.2, 124.7, 120.3, 117.5, 38.2, 33.8, 32.8, 28.0, 23.6; **HRMS** (ES^+/Q-TOF) m/z : [M+H] Calcd for [$C_{18}H_{20}O_2{}^{79}Br$] 347.0647; Found 347.0647.

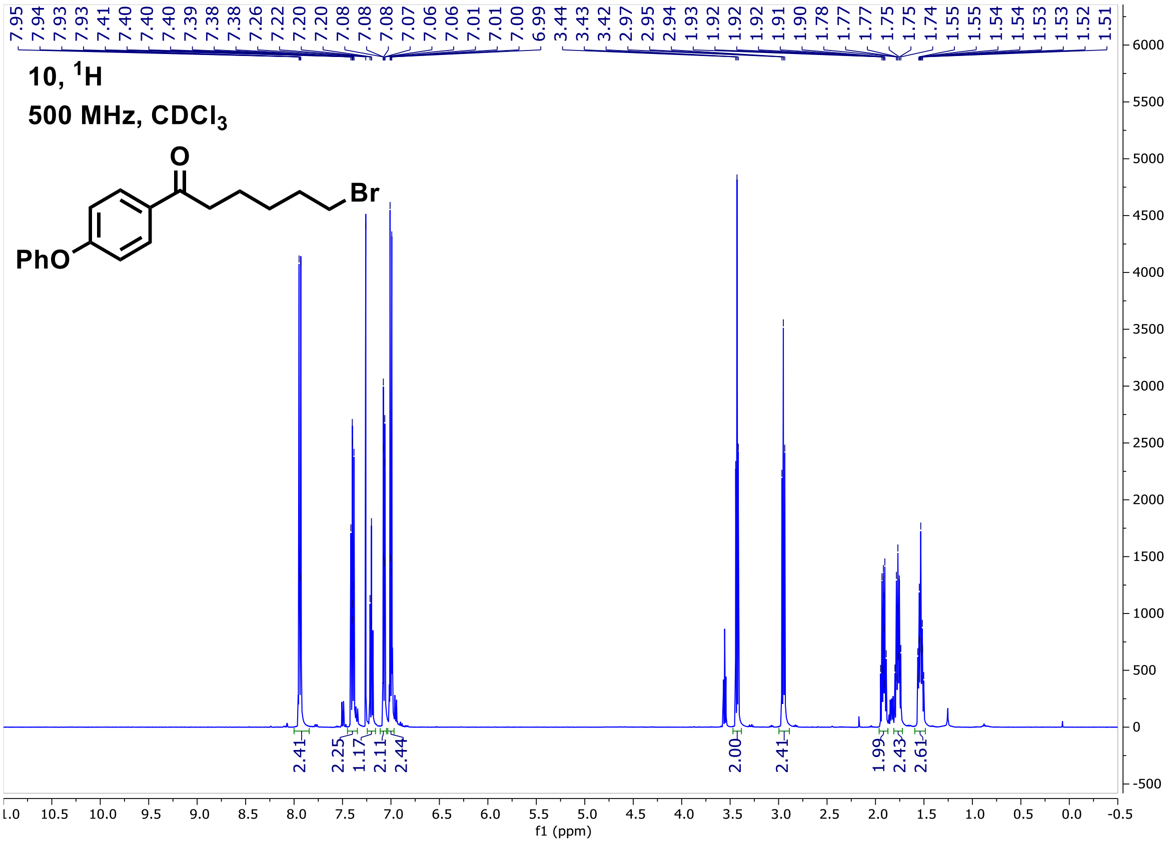
10, ¹H
500 MHz, CDCl₃
PhO
O
Br
f1 (ppm)

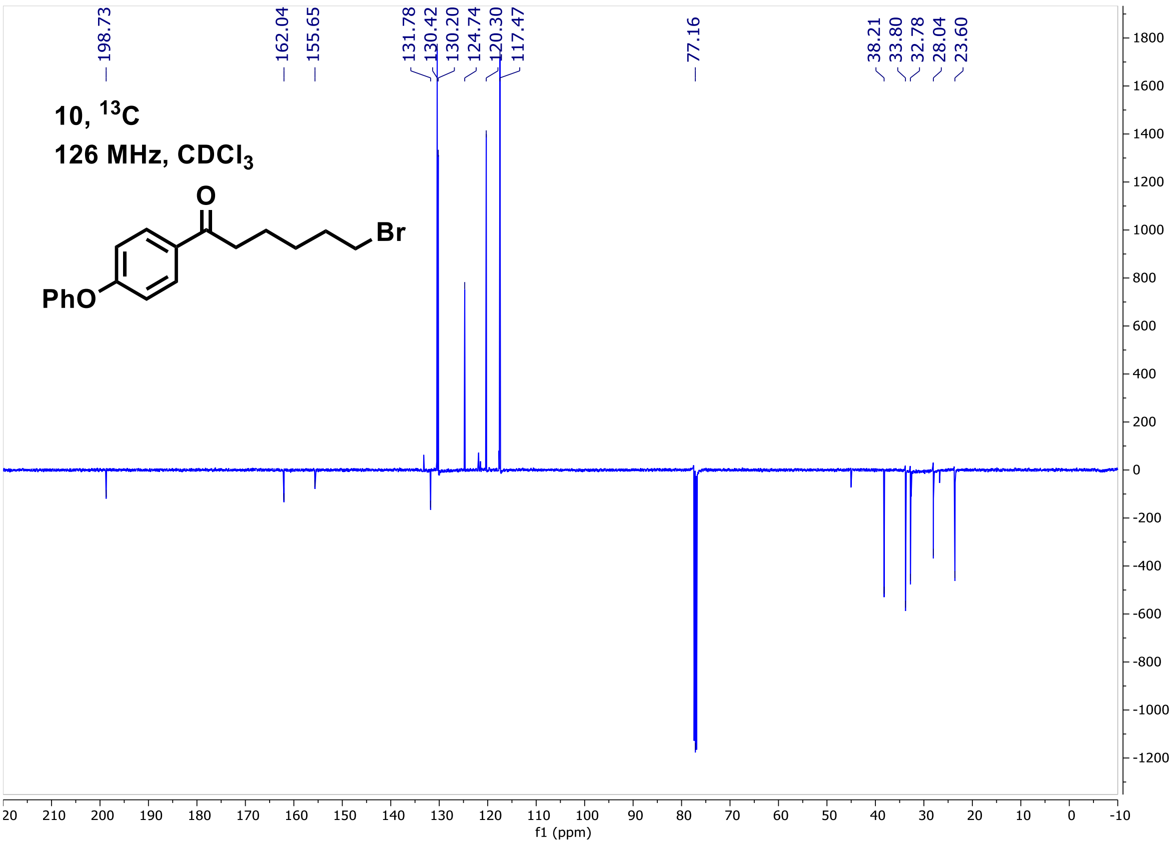
10, ¹³C
126 MHz, CDCl₃
PhO
O
Br
f1 (ppm)

6-bromo-1-(4-((tert-butyldimethylsilyl)oxy)phenyl)hexan-1-one (11)

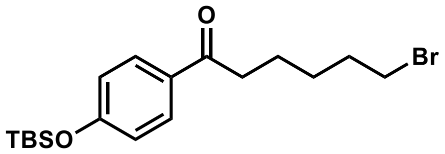

Prepared according to General Procedure (condition B) using **S11** (92 mg). The crude was purified by flash column chromatography (5→10 % EtOAc/Petrol, silica gel) to afford **11** (88 mg, 77%) as a colourless oil.

R_f = 0.61 (15% EtOAc/Petrol); **FTIR** (ν_{max} cm^{-1}, thin film) = 2935, 2862, 1676, 1595, 1510, 1463, 1417, 1363, 1255, 1203, 1148, 1069, 969, 835; **^{1}H NMR (500 MHz, $CDCl_3$)** δ = 7.92 – 7.85 (m, 2H), 6.92 – 6.82 (m, 2H), 3.43 (t, *J* = 6.8 Hz, 2H), 2.93 (t, *J* = 6.9 Hz, 2H), 2.00 – 1.86 (m, 2H), 1.81 – 1.71 (m, 2H), 1.58 – 1.47 (m, 2H), 0.99 (s, 9H), 0.23 (s, 6H); **^{13}C NMR (126 MHz, $CDCl_3$)** δ = 198.9, 160.3, 130.7, 130.3, 120.1, 38.1, 33.8, 32.8, 28.1, 25.7, 23.6, 18.4, -4.2; **HRMS =** (ES^+/Q-TOF) m/z : [M+H] Calcd for [$C_{18}H_{30}O_2SiBr$] 385.1198, found 385.1207.

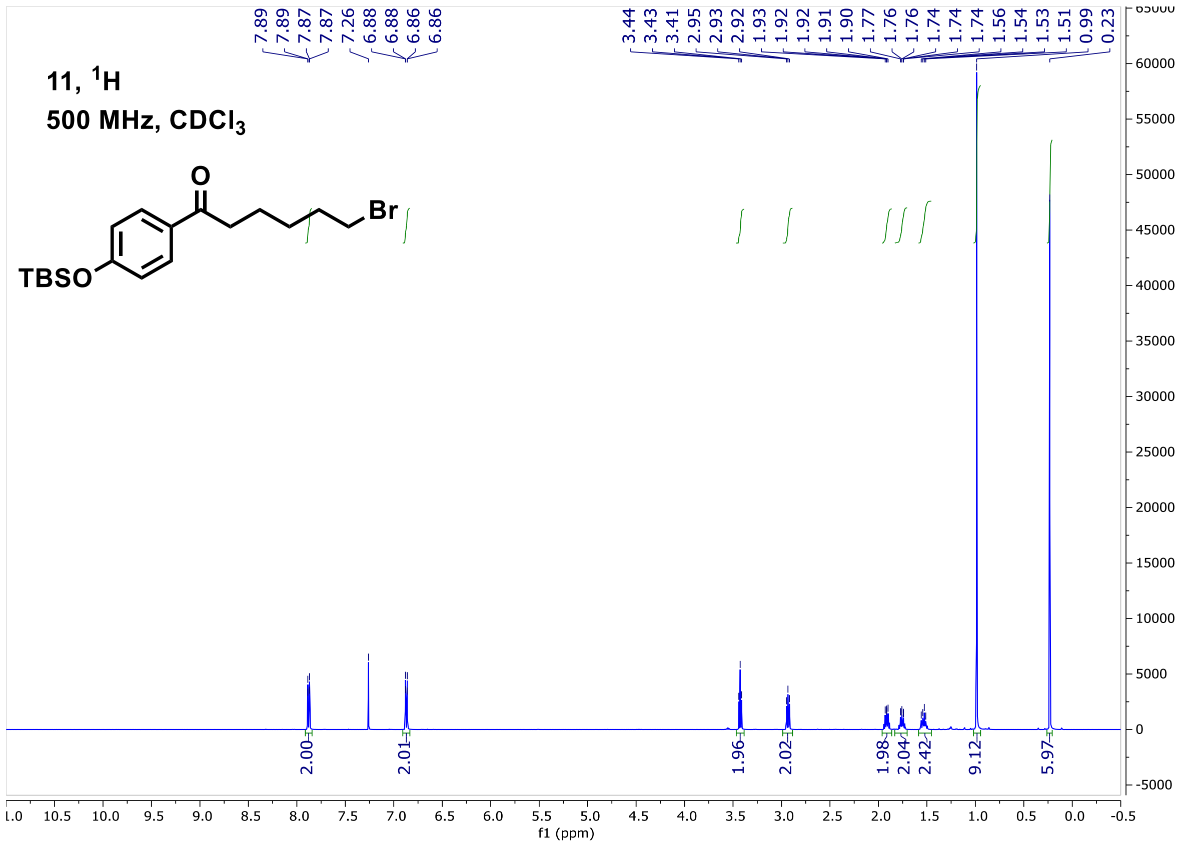

11, 1H
500 MHz, CDCl3
TBSO
O
Br
f1 (ppm)

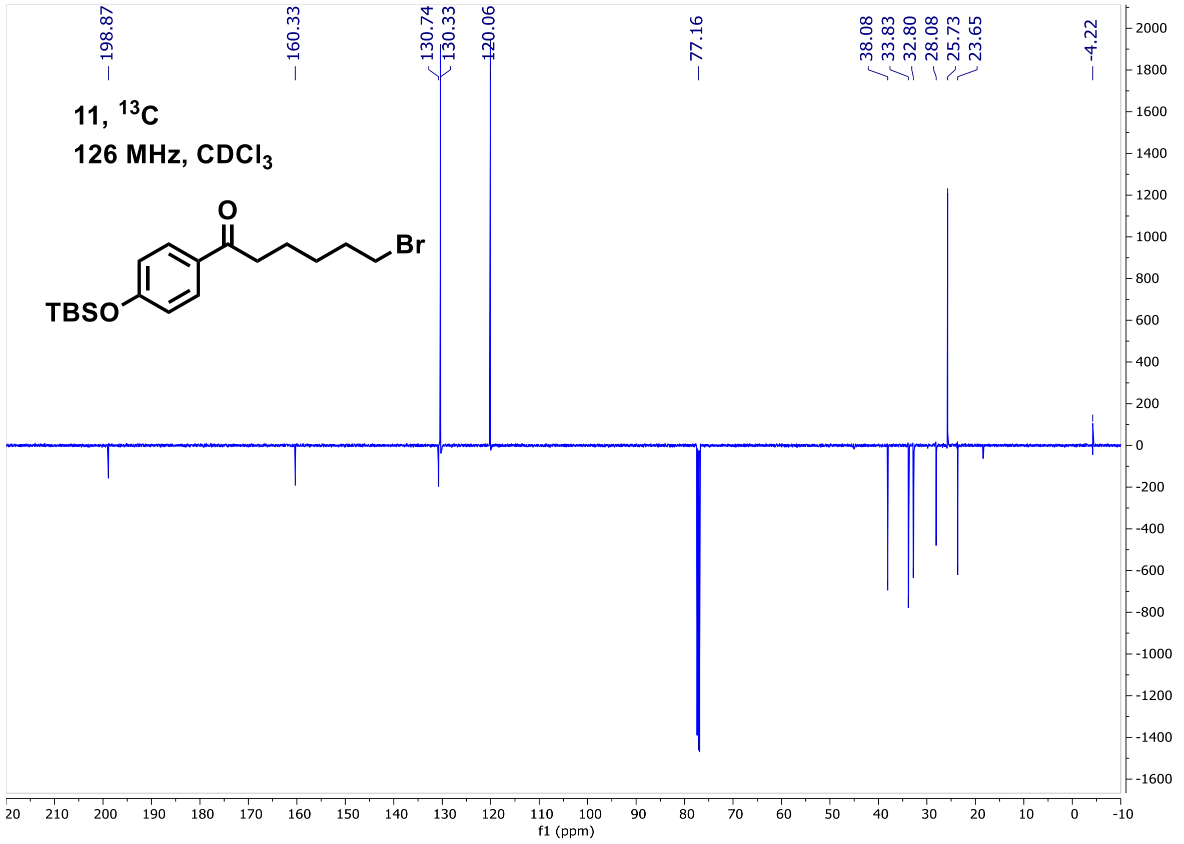

11, 13C
126 MHz, CDCl3
TBSO
O
Br
f1 (ppm)

6-bromo-1-(3-fluoro-4-methoxyphenyl)hexan-1-one (12)

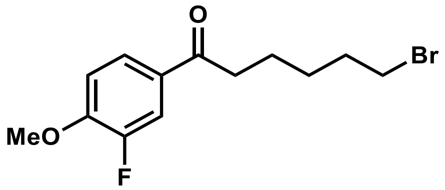

Prepared according to General Procedure (condition B) using **S12** (67 mg). The crude was purified by flash column chromatography (5→10% EtOAc/Petrol, silica gel) to afford **12** (46 mg, 51%) as a colourless oil.

R_f = 0.39 (15% EtOAc/Petrol); **FTIR** (ν_{max} cm^{-1}, thin film) = 2930, 2857, 1678, 1599, 1505, 1464, 1411, 1263, 1166, 911, 842, 781; **^{1}H NMR (500 MHz, $CDCl_3$)** δ = 7.74 (ddd, *J* = 8.5, 2.1, 1.1 Hz, 1H), 7.70 (dd, *J* = 11.9, 2.1 Hz, 1H), 7.00 (app t, *J* = 8.3 Hz, 1H), 3.96 (s, 3H), 3.43 (t, *J* = 6.7 Hz, 2H), 2.92 (t, *J* = 7.3 Hz, 2H), 2.00 – 1.85 (m, 2H), 1.76 (m, 2H), 1.54 – 1.49 (m, 2H); **^{13}C NMR (126 MHz, $CDCl_3$)** δ = 197.8, 152.2 (d, *J* = 248.2 Hz), 151.9 (d, 11.3 Hz), 130.4 (d, *J* = 4.6 Hz), 125.4 (d, *J* = 3.4 Hz), 115.9 (d, *J* = 18.9 Hz), 112.5 (d, *J* = 1.8 Hz), 56.4, 38.1, 33.8, 32.6, 28.0, 23.5; **^{19}F NMR (471 MHz, $CDCl_3$)** δ = -134.2; **HRMS** (ES^+/Q-TOF) m/z : [M+H] Calcd for [$C_{13}H_{17}O_2FBr$] 303.0396, found 303.0406.

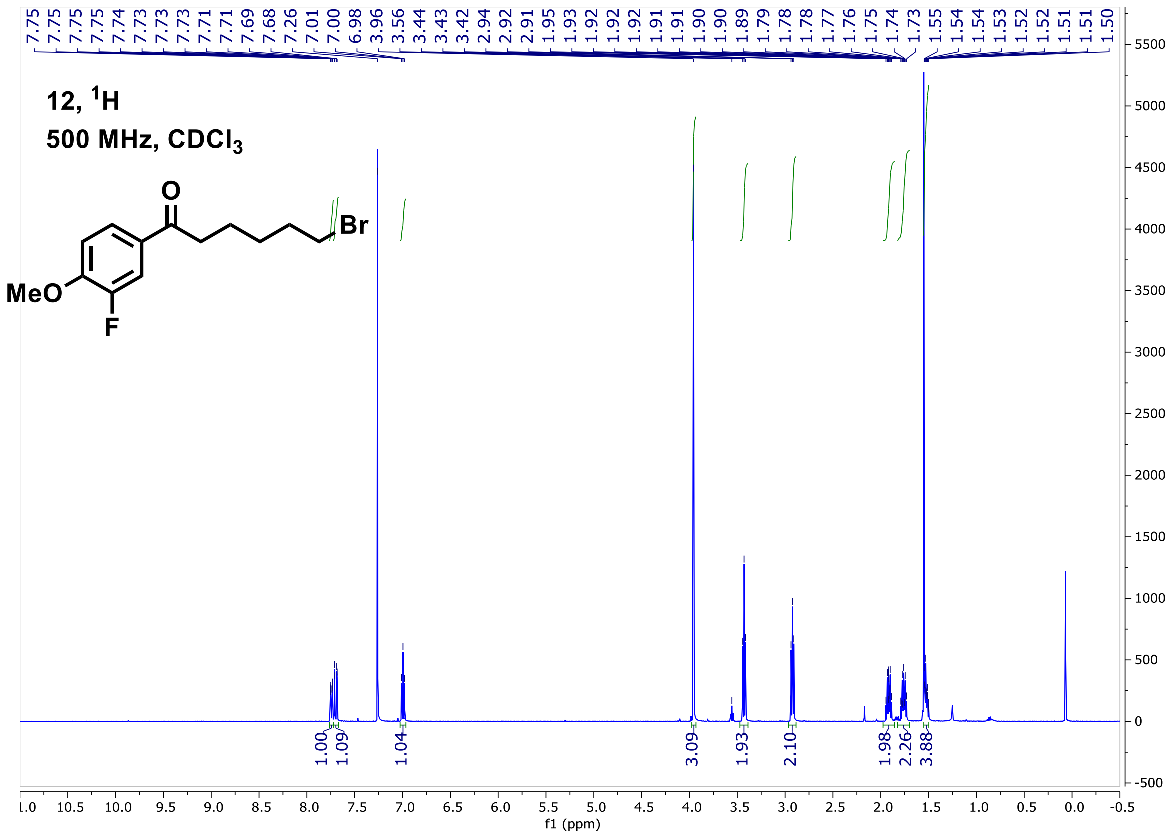
7.75 7.75 7.75 7.75 7.74 7.73 7.73 7.73 7.71 7.71 7.69 7.68 7.26 7.01 7.00 6.98 3.96 3.56 3.44 3.43 3.42 2.94 2.92 2.91 1.95 1.93 1.92 1.92 1.92 1.91 1.91 1.90 1.90 1.89 1.79 1.78 1.78 1.77 1.76 1.75 1.74 1.73 1.55 1.54 1.54 1.53 1.52 1.52 1.51 1.51 1.50
12, 1H
500 MHz, CDCl3
O
Br
MeO
F
1.00
1.09
1.04
3.09
1.93
2.10
1.98
2.26
3.88
f1 (ppm)

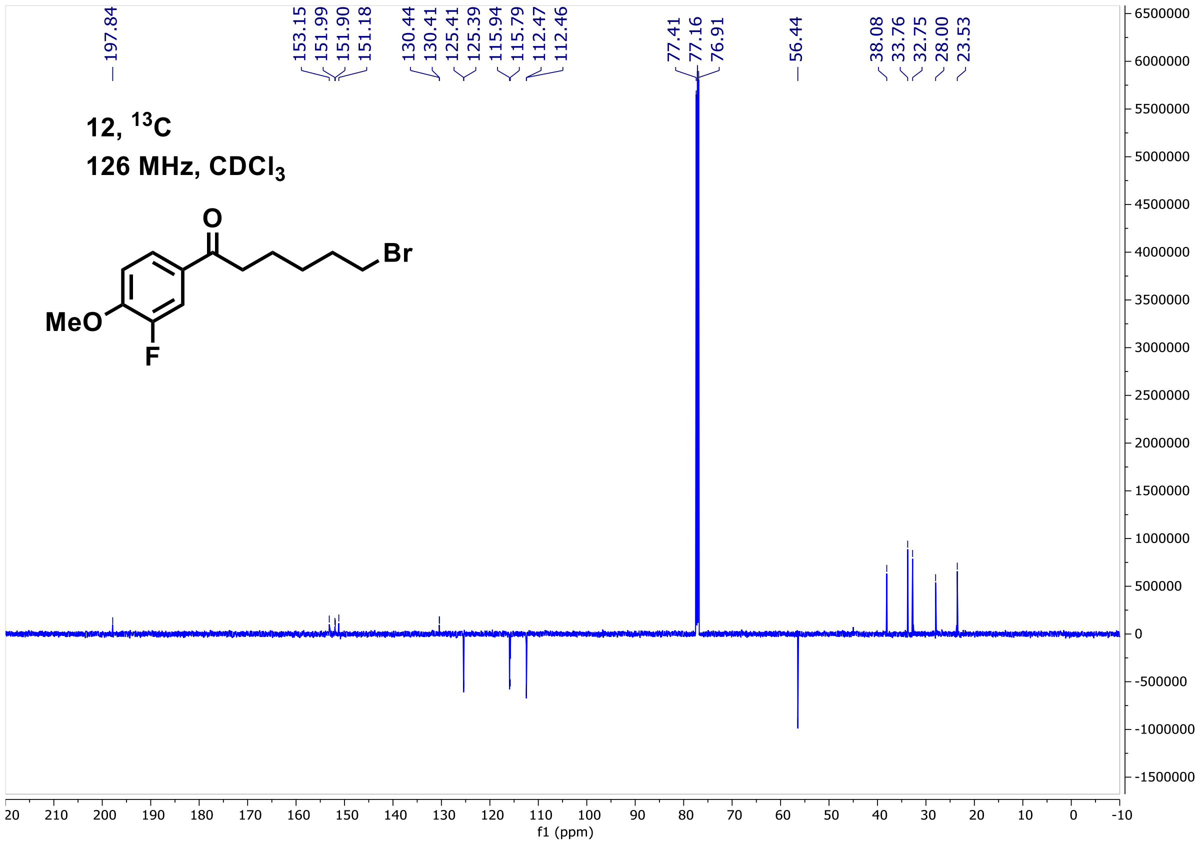
197.84
153.15 151.99 151.90 151.18
130.44 130.41 125.41 125.39 115.94 115.79 112.47 112.46
77.41 77.16 76.91
56.44
38.08 33.76 32.75 28.00 23.53
12, 13C
126 MHz, CDCl3
O
Br
MeO
F
f1 (ppm)

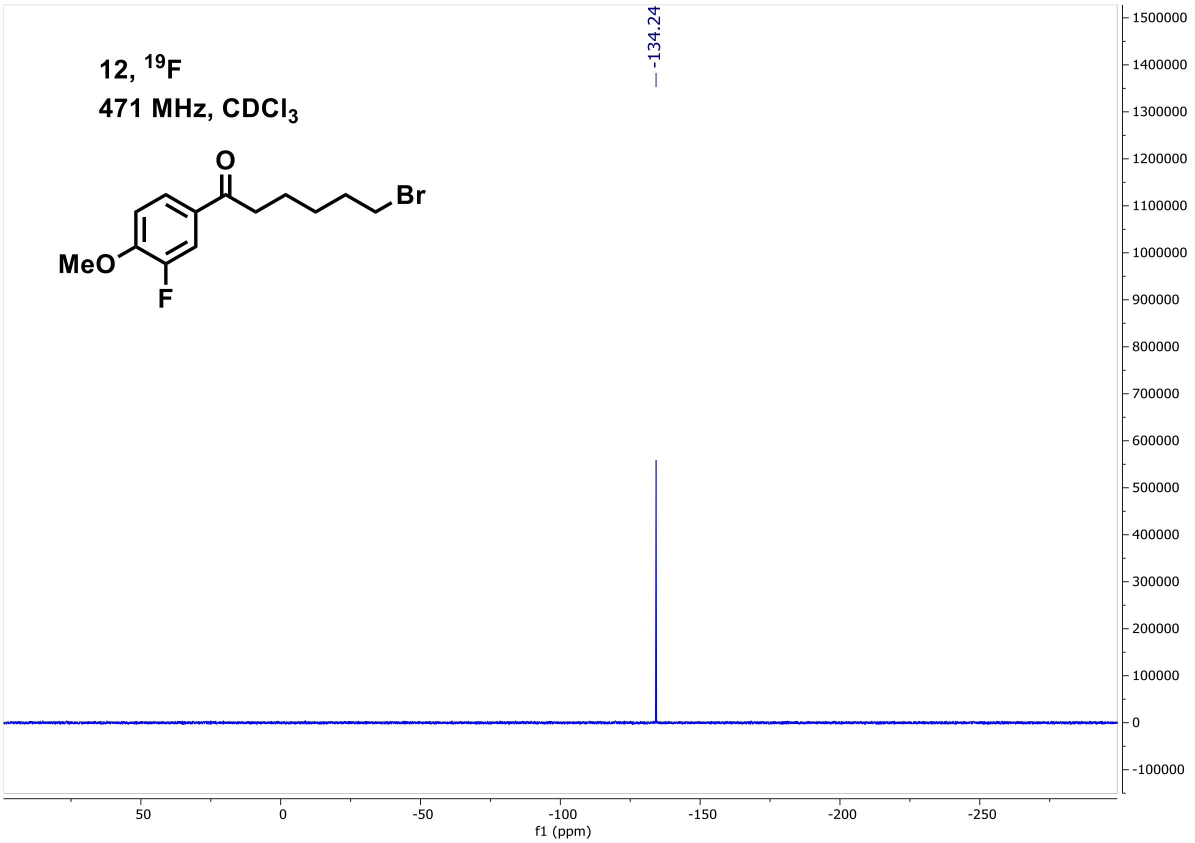
12, ^{19}F
471 MHz, $CDCl_3$
O
Br
MeO
F
-134.24
f1 (ppm)
50
0
-50
-100
-150
-200
-250
1500000
1400000
1300000
1200000
1100000
1000000
900000
800000
700000
600000
500000
400000
300000
200000
100000
0
-100000

6-bromo-1-(4-(tert-butyl)phenyl)hexan-1-one (13)

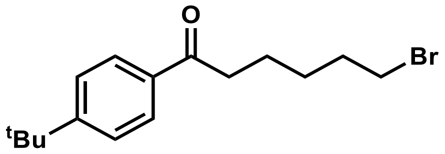

Prepared according to General Procedure (condition E) using **S13** (70 mg). The crude was purified by flash column chromatography (5→10 % EtOAc/Petrol, silica gel) to afford **13** (45 mg, 48%) as a colourless oil.

R_f = 0.31 (15% EtOAc/Petrol); **FTIR** (ν_{max} cm^{-1}, thin film) = 2965, 2868, 1682, 1604, 1465, 1403, 1361, 1265, 1203, 1193, 1148, 1107, 980, 828; **^{1}H NMR (500 MHz, $CDCl_3$)** δ = 7.97 – 7.85 (m, 2H), 7.57 – 7.41 (m, 2H), 3.44 (t, *J* = 6.8 Hz, 2H), 3.13 – 2.85 (m, 2H), 2.02 – 1.85 (m, 2H), 1.83 – 1.70 (m, 2H), 1.61 – 1.46 (m, 2H), 1.35 (s, 9H); **^{13}C NMR (126 MHz, $CDCl_3$)** δ = 200.0, 156.6, 134.5, 128.1, 125.7, 38.3, 35.2, 33.9, 32.8, 31.2, 28.0, 23.5; **HRMS** (ES^+/Q-TOF) m/z : [M+H] Calcd for [$C_{16}H_{24}BrO$] 311.1011; Found 311.1016.

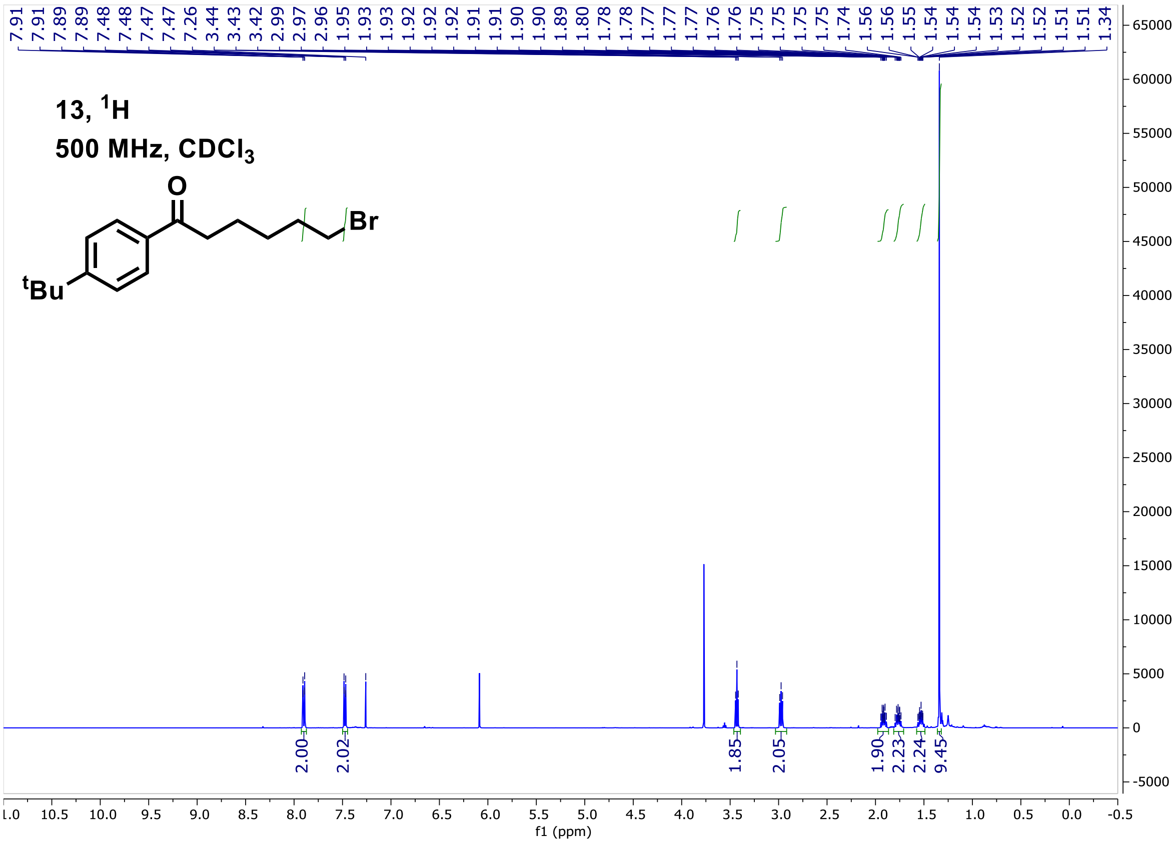
13, ^{1}H
500 MHz, CDCl$_3$
O
Br
^{t}Bu
7.91 7.91 7.89 7.89 7.48 7.48 7.47 7.47 7.26 3.44 3.43 3.42 2.99 2.97 2.96 1.95 1.93 1.93 1.92 1.92 1.92 1.91 1.91 1.90 1.90 1.89 1.80 1.78 1.78 1.77 1.77 1.77 1.76 1.76 1.75 1.75 1.75 1.75 1.74 1.56 1.56 1.55 1.54 1.54 1.54 1.53 1.52 1.52 1.51 1.51 1.34
2.00 2.02 1.85 2.05 1.90 2.23 2.24 9.45
f1 (ppm)

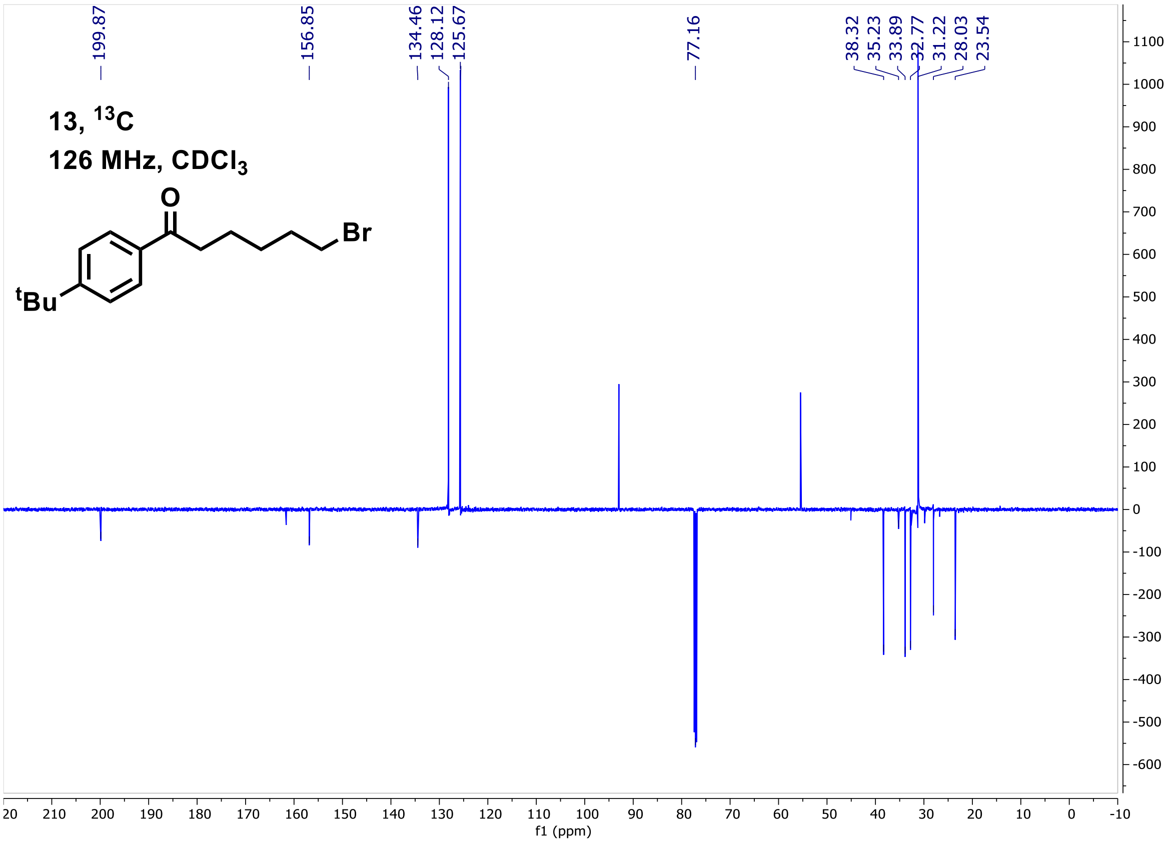
13, ^{13}C
126 MHz, CDCl$_3$
O
Br
^{t}Bu
199.87
156.85
134.46
128.12
125.67
77.16
38.32
35.23
33.89
32.77
31.22
28.03
23.54
f1 (ppm)

1-([1,1'-biphenyl]-4-yl)-6-bromohexan-1-one (14)

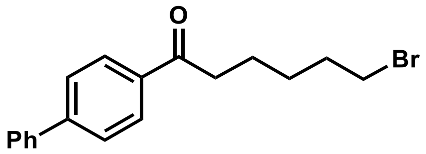

Prepared according to General Procedure (condition C) using **S14** (76 mg,). The crude residue was purified by flash column chromatography (2→5% EtOAc/Petrol, silica gel) to afford **14** (43.7 mg, 44%) as a brown oil.

R_f = 0.30 (15% EtOAc/Petrol); **FTIR** (ν_{max} cm^{-1}, thin film); 2976, 2935, 2866, 1676, 1595, 1571, 1504, 1456, 1244, 1205, 1153, 1124; **^{1}H NMR (500 MHz, $CDCl_3$)** δ = 8.09 – 7.99 (m, 2H), 7.74 – 7.67 (m, 2H), 7.62-7.64 (m, 2H), 7.51 – 7.45 (m, 2H), 7.44 – 7.38 (m, 1H), 3.45 (t, *J* = 6.8 Hz, 2H), 3.09 – 2.98 (m, 2H), 1.94 (dt, *J* = 14.5, 6.8 Hz, 2H), 1.85 – 1.76 (m, 2H), 1.63 – 1.50 (m, 2H); **^{13}C NMR (126 MHz, $CDCl_3$)** δ = 199.8, 145.8, 140.0, 135.7, 129.1, 128.8, 128.4, 127.4, 127.4, 38.5, 33.9, 32.8, 28.0, 23.5; **HRMS** ($ASAP^+$/Q-TOF) m/z : [M+H] Calcd for [$C_{18}H_{20}OBr$] 331.0698; Found 331.0689.

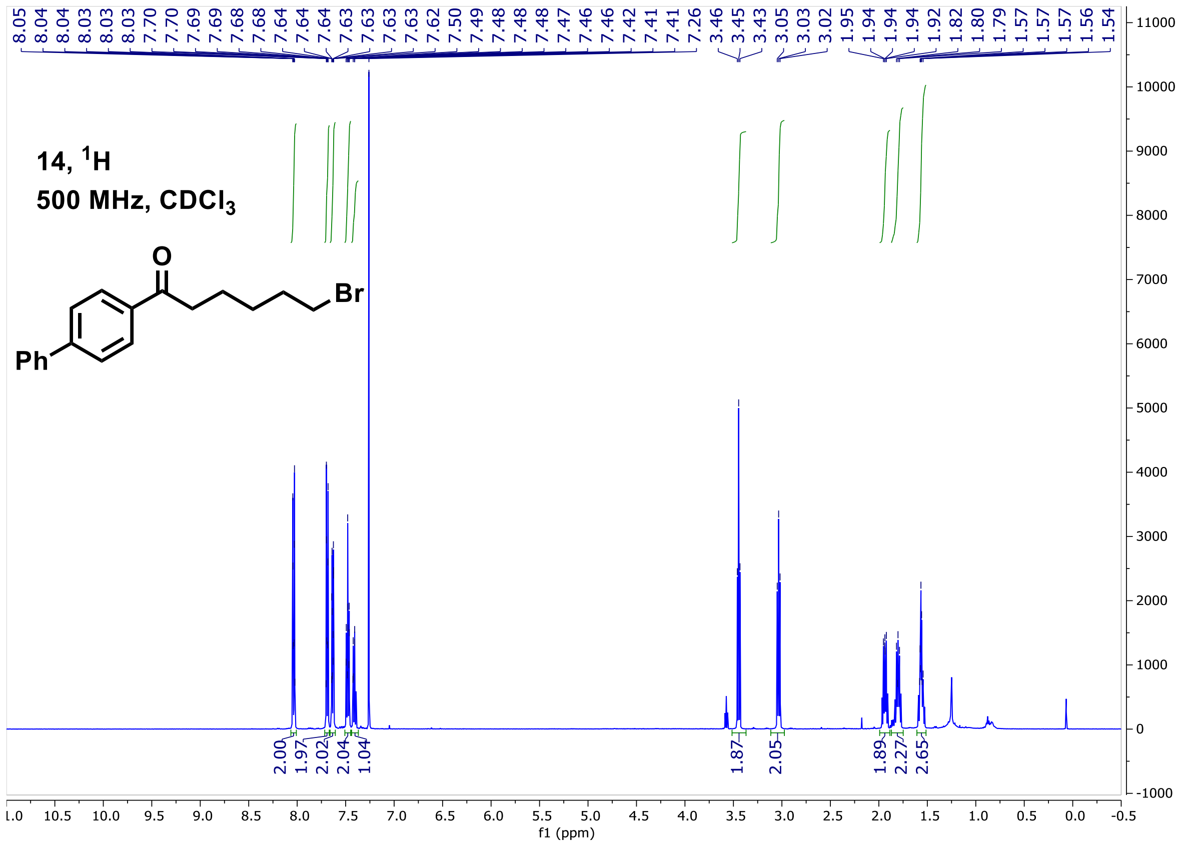
14, ^{1}H
500 MHz, $CDCl_3$
O
Br
Ph
f1 (ppm)

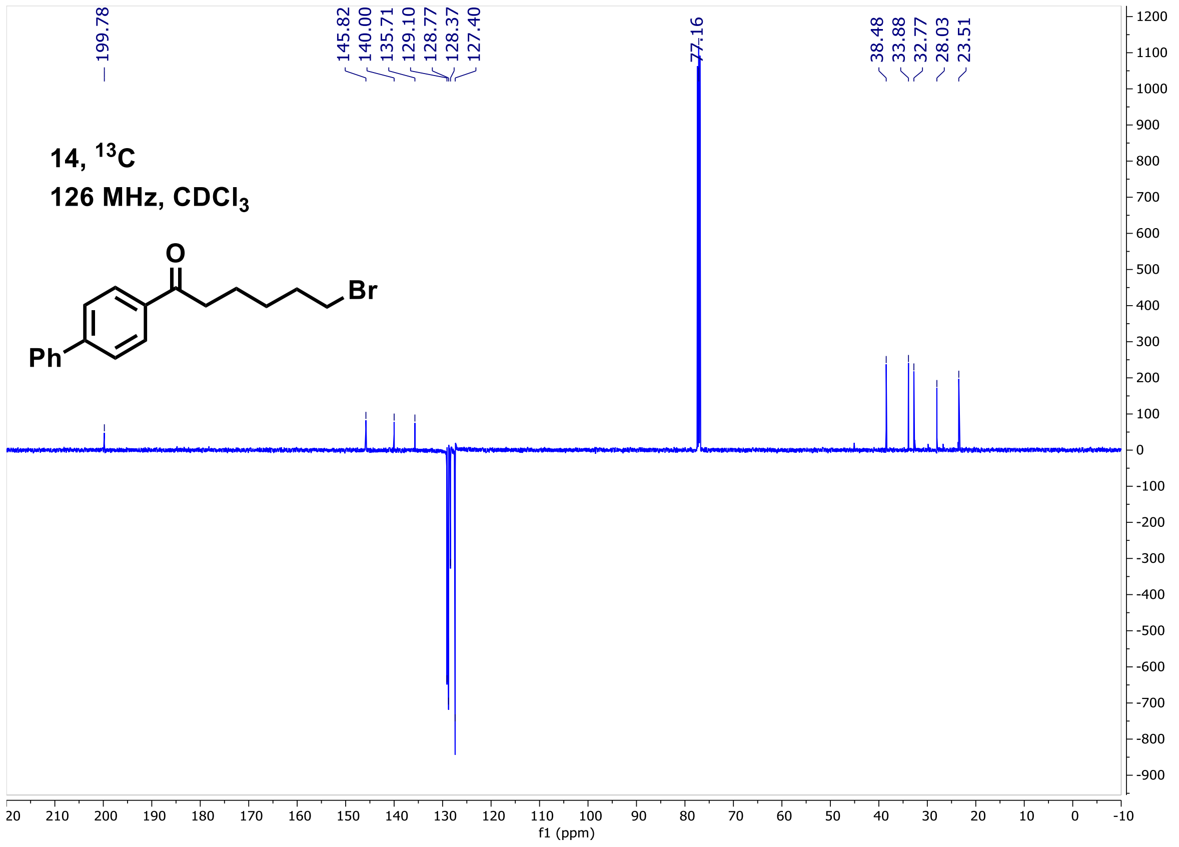
14, ^{13}C
126 MHz, $CDCl_3$
O
Br
Ph
f1 (ppm)

6-bromo-1-(naphthalen-2-yl)hexan-1-one (15)

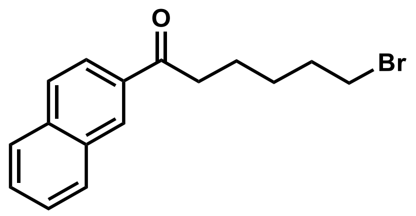

Prepared according to General Procedure (condition D) using **S15** (68 mg, 0.3 mmol). The crude residue was purified by flash column chromatography (3% EtOAc/Petrol, silica gel) to afford **15** (52 mg, 57%) as a brown oil.

R$_f$ = 0.30 (5% EtOAc/Petrol); **FTIR** (ν_{max} cm^{-1}, thin film); 3331 (br), 2933, 2839, 2312, 1909, 1676, 1597, 1369, 1305, 1263, 1184, 1130, 1026; **^{1}H NMR (500 MHz, CDCl$_3$)** δ = 8.46 (d, *J* = 10.9 Hz, 1H), 8.01– 8.04 (m, 1H), 7.97 (d, *J* = 8.0 Hz, 1H), 7.93 – 7.85 (m, 2H), 7.65 – 7.53 (m, 2H), 3.50 – 3.38 (m, 2H), 3.13 (dd, *J* = 13.3, 5.9 Hz, 2H), 2.01 – 1.90 (m, 2H), 1.90 – 1.76 (m, 2H), 1.57– 1.60 (m, 2H); **^{13}C NMR (126 MHz, CDCl$_3$)** δ = 200.1, 135.7, 134.4, 132.7, 129.8, 129.7, 128.6, 128.6, 127.9, 126.9, 124.0, 38.5, 33.8, 32.8, 28.1, 23.6.

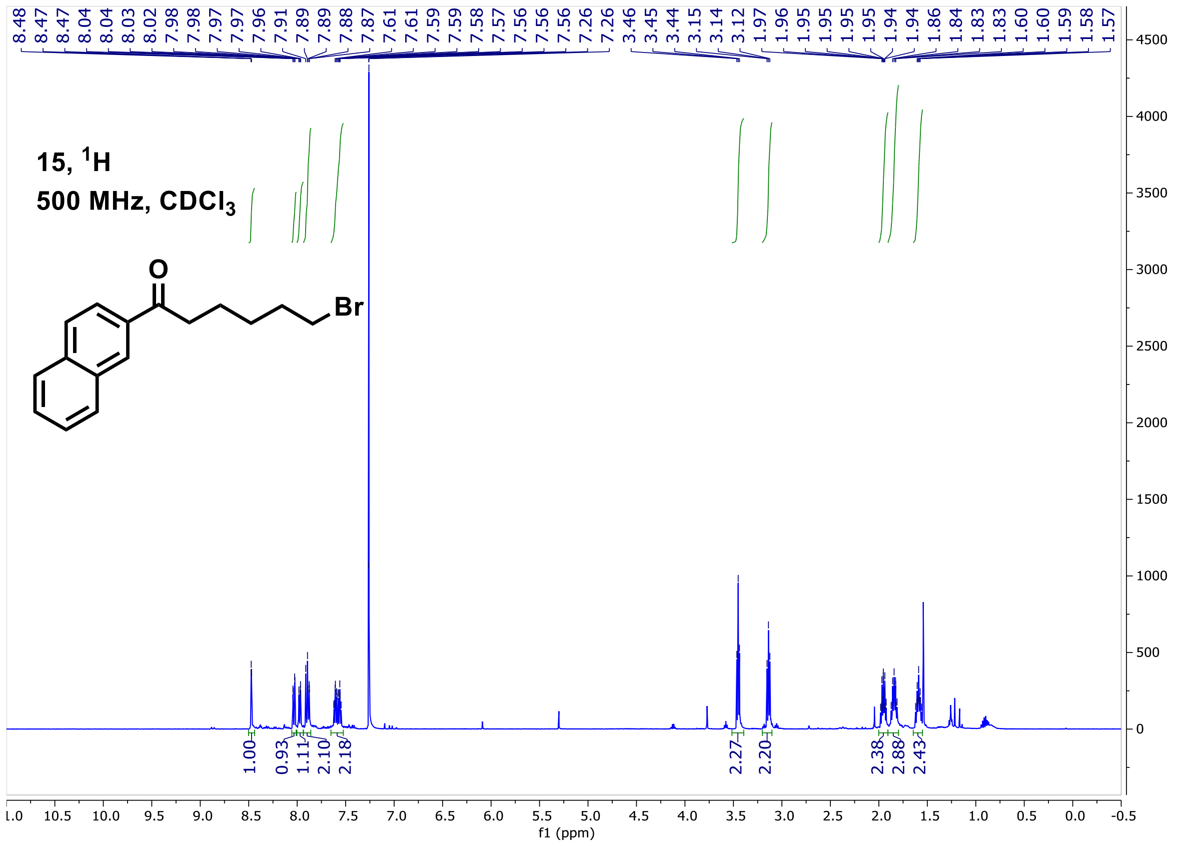
15, ¹H
500 MHz, CDCl3
O
Br
f1 (ppm)

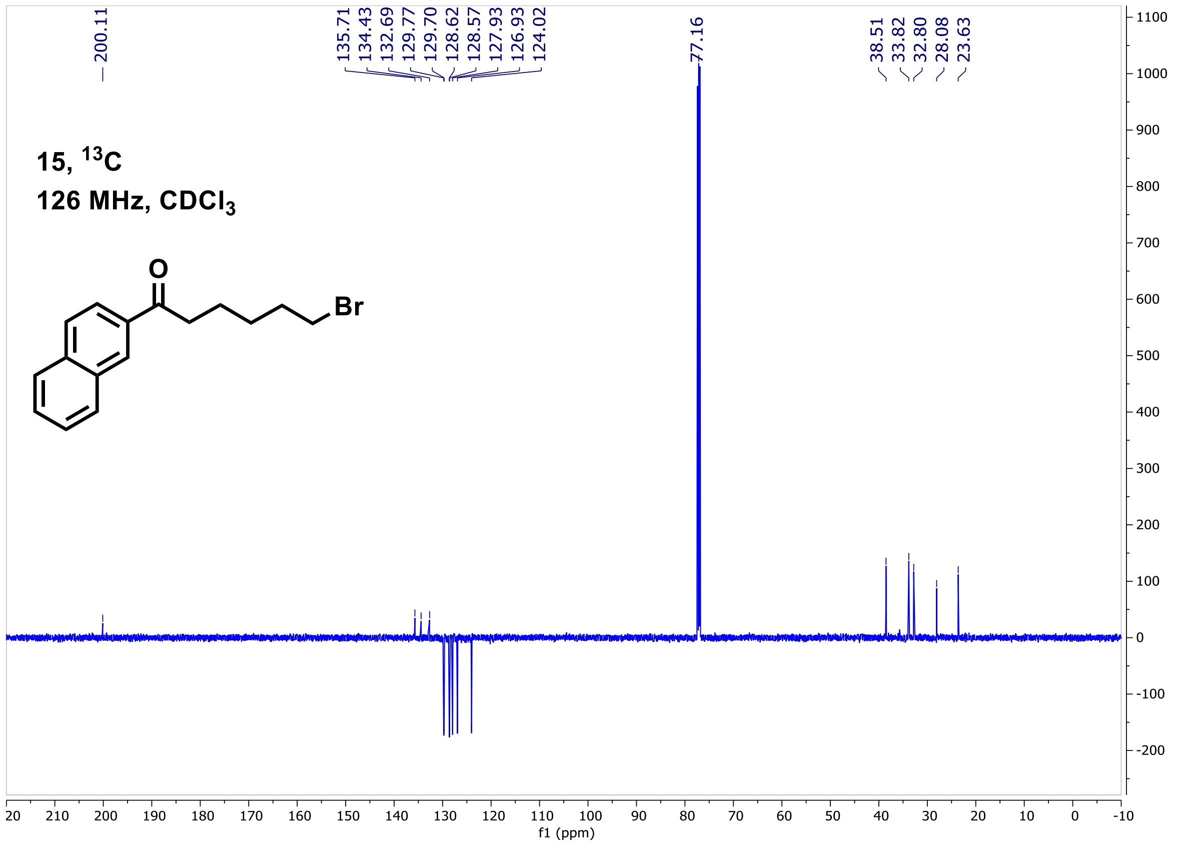
15, ¹³C
126 MHz, CDCl3
O
Br
f1 (ppm)

6-bromo-1-(phenanthren-9-yl)hexan-1-one (16)

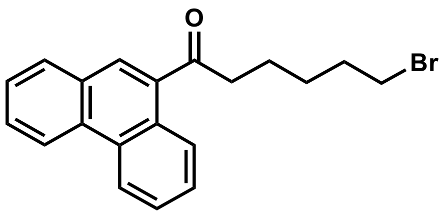

Prepared according to General Procedure (condition D) using **S16** (68 mg, 0.3 mmol). The crude residue was purified by flash column chromatography (2→5% EtOAc/Petrol, silica gel) to afford **16** (62 mg, 58%) as a brown oil.

R_f = 0.30 (10% EtOAc/Petrol); **FTIR** (ν_{max} cm^{-1}, thin film): 2976, 2935, 2866, 1676, 1595, 1571, 1504, 1456, 1244, 1205, 1153, 1124; **^{1}H NMR (500 MHz, $CDCl_3$)** δ = 8.76 – 8.71 (m, 1H), 8.69 (d, *J* = 8.0 Hz, 1H), 8.50 (dd, *J* = 8.2, 1.1 Hz, 1H), 8.09 (s, 1H), 7.99 – 7.92 (m, 1H), 7.73 – 7.77 (ddd, *J* = 8.4, 7.0, 1.4 Hz, 1H), 7.70 (ddd, *J* = 8.3, 7.0, 1.5 Hz, 1H), 7.68 – 7.63 (m, 2H), 3.45 (t, *J* = 6.7 Hz, 2H), 3.16 (t, *J* = 7.3 Hz, 2H), 2.00 – 1.91 (m, 2H), 1.86-1.89 (m, 2H), 1.67 – 1.57 (m, 2H); **^{13}C NMR (126 MHz, $CDCl_3$)** δ = 204.8, 135.7, 131.9, 131.0, 130.2, 129.8, 129.1, 128.9, 128.5, 127.7, 127.3, 127.3, 126.7, 123.0, 122.9, 42.2, 33.8, 32.8, 28.0, 23.9; **HRMS** (ES^+/Q-TOF) m/z : [M+H] Calcd for [$C_{20}H_{20}OBr$] 355.0698; Found 355.0695.

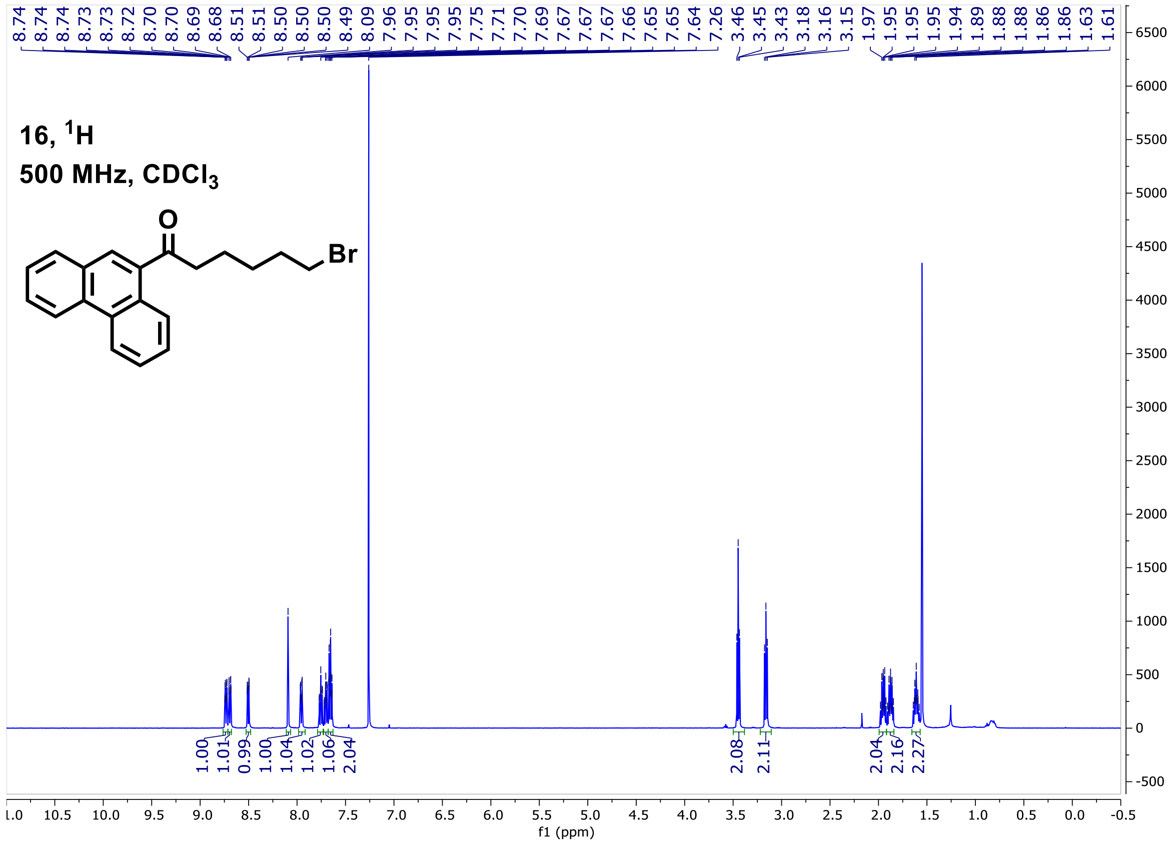

8.74
8.74
8.74
8.73
8.73
8.72
8.70
8.70
8.69
8.68
8.51
8.51
8.50
8.50
8.50
8.49
8.09
7.96
7.95
7.95
7.95
7.75
7.71
7.70
7.69
7.67
7.67
7.67
7.66
7.65
7.65
7.64
7.26
3.46
3.45
3.43
3.18
3.16
3.15
1.97
1.95
1.95
1.95
1.94
1.89
1.88
1.88
1.86
1.63
1.61
16, ^{1}H
500 MHz, $CDCl_3$
O
Br
1.00
1.01
0.99
1.00
1.04
1.02
1.06
2.04
2.08
2.11
2.04
2.16
2.27
f1 (ppm)

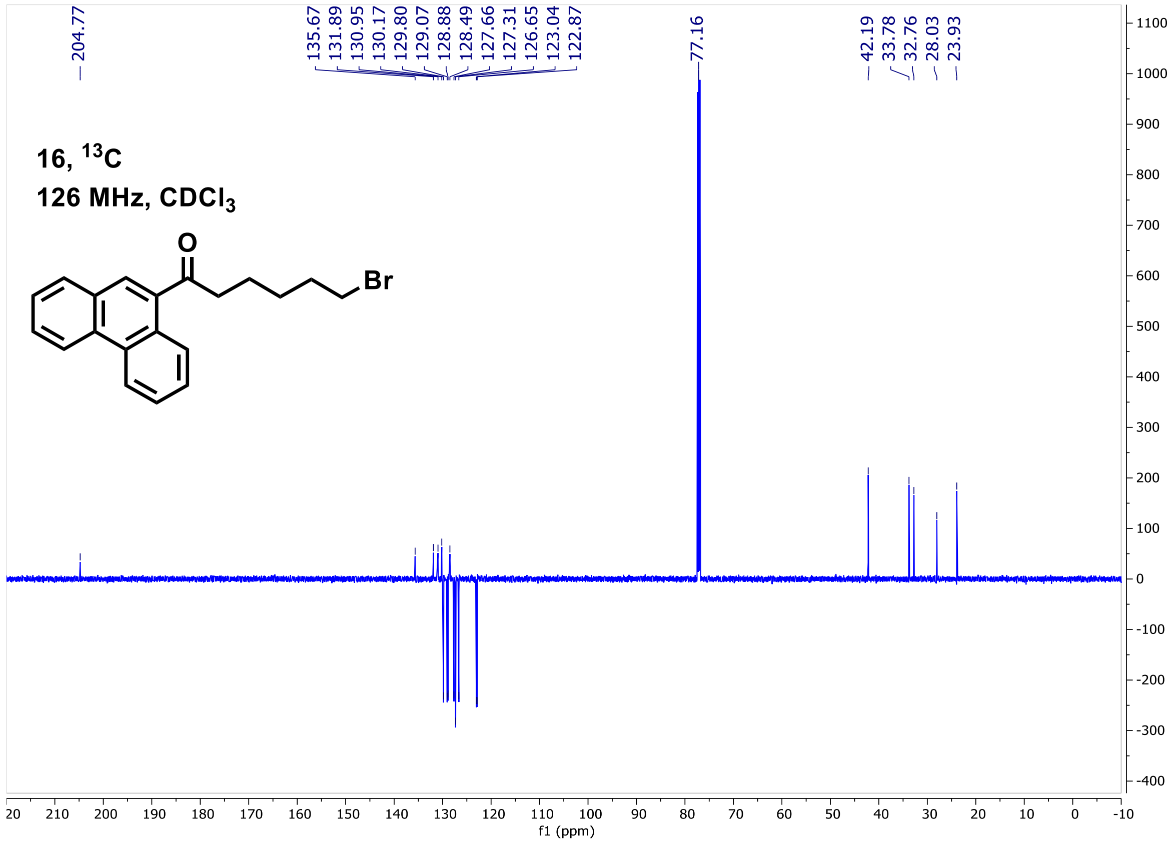

204.77
135.67
131.89
130.95
130.17
129.80
129.07
128.88
128.49
127.66
127.31
126.65
123.04
122.87
77.16
42.19
33.78
32.76
28.03
23.93
16, ^{13}C
126 MHz, $CDCl_3$
O
Br
f1 (ppm)

((1R,3s,5S,7s)-7-bromobicyclo[3.3.1]nonan-3-yl)(4-methoxyphenyl)methanone (17)

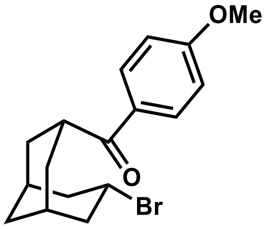

Prepared according to General Procedure (standard conditions) using **S17** (78 mg). The crude was purified by flash column chromatography (5→10 % EtOAc/Petrol, silica gel) to afford **17** (85 mg, 84%, >95:<5 d.r.) as a colourless oil.

R_f = 0.58 (15% EtOAc/Petrol); **FTIR** (ν_{max} cm^{-1}, thin film) = 2933, 1674, 1599, 1512, 1459, 1248, 1210, 1165, 1131, 1030, 839, 752; **^{1}H NMR (500 MHz, $CDCl_3$)** δ = 7.93 (d, *J* = 8.9 Hz, 2H), 6.94 (d, *J* = 8.9 Hz, 2H), 4.58 (tt, *J* = 12.2, 4.8 Hz, 1H), 3.87 (s, 3H), 3.49 (tt, *J* = 12.6, 6.1 Hz, 1H), 2.34 – 2.16 (m, 4H), 2.13 – 2.02 (m, 2H), 1.96 – 1.81 (m, 3H), 1.64 – 1.48 (m, 2H), 1.31 (dt, *J* = 13.2, 2.6 Hz, 1H); **^{13}C NMR (126 MHz, $CDCl_3$)** δ = 202.4, 163.6, 130.6, 129.6, 113.9, 55.6, 47.8, 45.3, 36.9, 29.4, 28.3, 28.1; **HRMS** (ES^+/Q-TOF) m/z : [M+H] Calcd for [$C_{17}H_{22}O_2Br$] 337.0803; Found 337.0810.

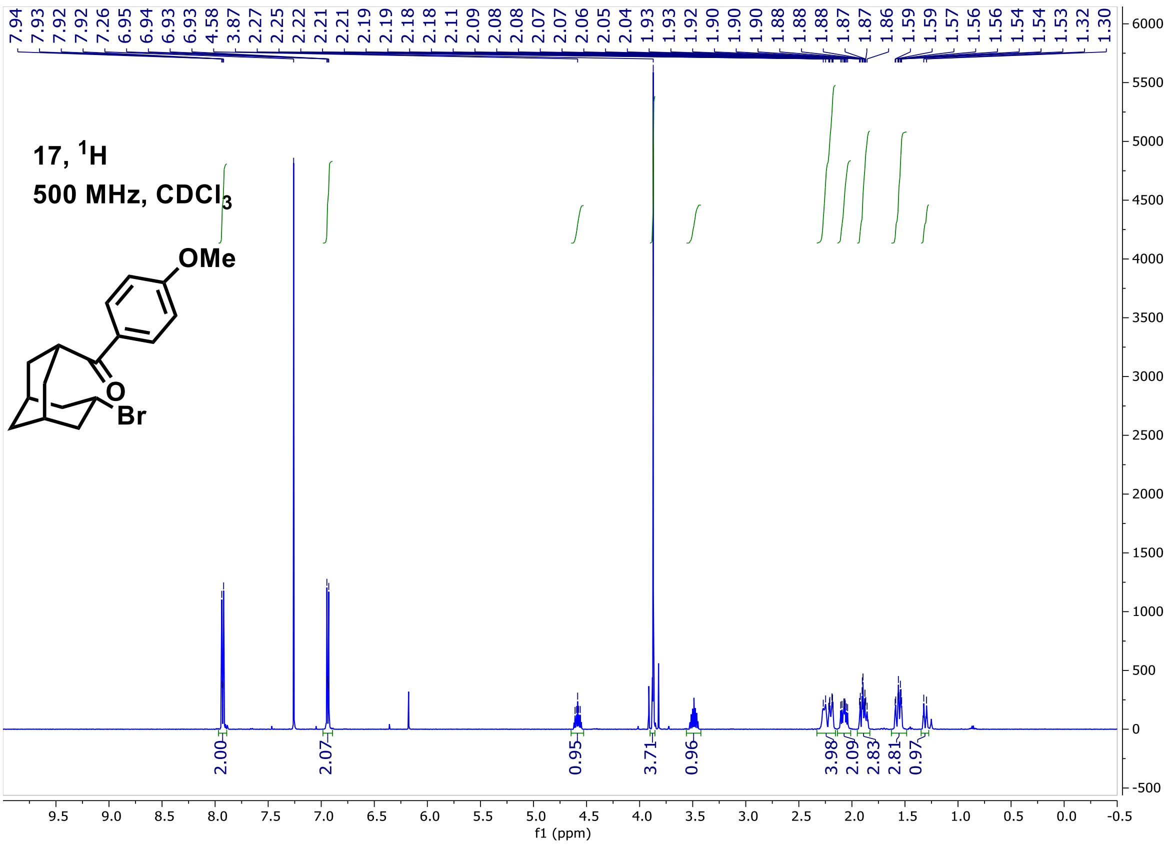
7.94 7.93 7.92 7.92 7.26 6.95 6.94 6.93 6.93 4.58 3.87 2.27 2.25 2.22 2.21 2.21 2.19 2.19 2.18 2.11 2.09 2.08 2.08 2.07 2.06 2.05 2.04 1.93 1.93 1.92 1.90 1.90 1.88 1.88 1.87 1.87 1.86 1.59 1.59 1.57 1.56 1.56 1.54 1.54 1.53 1.32 1.30
17, 1H
500 MHz, CDCl3
OMe
O
Br
2.00 2.07 0.95 3.71 0.96 3.98 2.09 2.83 2.81 0.97
f1 (ppm)

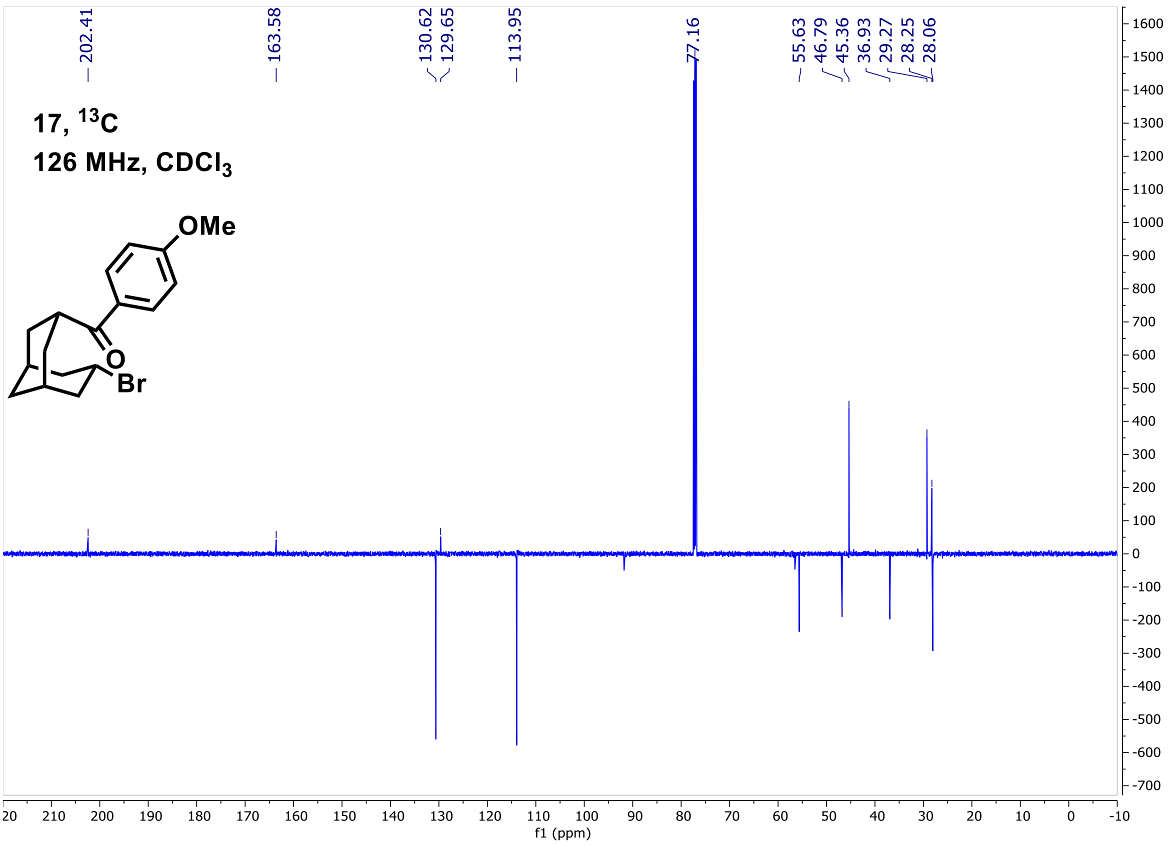
202.41 163.58 130.62 129.65 113.95 77.16 55.63 46.79 45.36 36.93 29.27 28.25 28.06
17, 13C
126 MHz, CDCl3
OMe
O
Br
f1 (ppm)

4-((1R)-2-bromocyclohexyl)-1-(4-methoxyphenyl)butan-1-one (18)

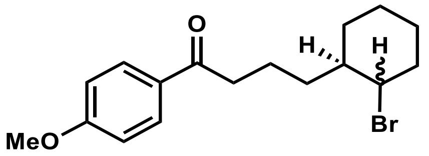

Prepared according to General Procedure (condition B) A using **S18** (78 mg, 0.30 mmol). The crude residue was purified by flash column chromatography (7→10% EtOAc/Petrol, silica gel) to afford **18** (71 mg, 70%) as a yellow oil. The compound was obtained as an inseparable mixture of diastereoisomers (1:1 d.r.).

<u>Data for both diastereoisomers:</u>

^{1}H NMR (500 MHz, $CDCl_3$) δ = 7.93 – 7.84 (m, 2H), 6.91 – 6.82 (m, 2H), 3.80 (s, 3H), 2.95 – 2.77 (m, 2H), 2.33 – 2.04 (m, 1H), 1.99 – 1.50 (m, 7H), 1.50 – 1.13 (m, 6H); **^{13}C NMR (126 MHz, $CDCl_3$)** δ = 199.0, 198.9, 163.5, 163.5, 130.4, 130.3, 130.2, 113.8, 62.1, 60.9, 55.6, 46.0, 42.6, 38.9, 38.5, 38.5, 35.3, 34.9, 31.9, 27.6, 27.5, 25.5, 25.5, 21.5, 21.3, 21.1; **HRMS** (ES^+/Q-TOF) m/z : [M+H] Calcd for [$C_{17}H_{24}O_2Br$] 339.0960; Found 339.0954.

R_f = 0.27 (10% EtOAc/Petrol); **FTIR** (ν_{max} cm^{-1}, thin film); 2976, 2935, 2866, 1676, 1595, 1571, 1504, 1456, 1244, 1205, 1153, 1124

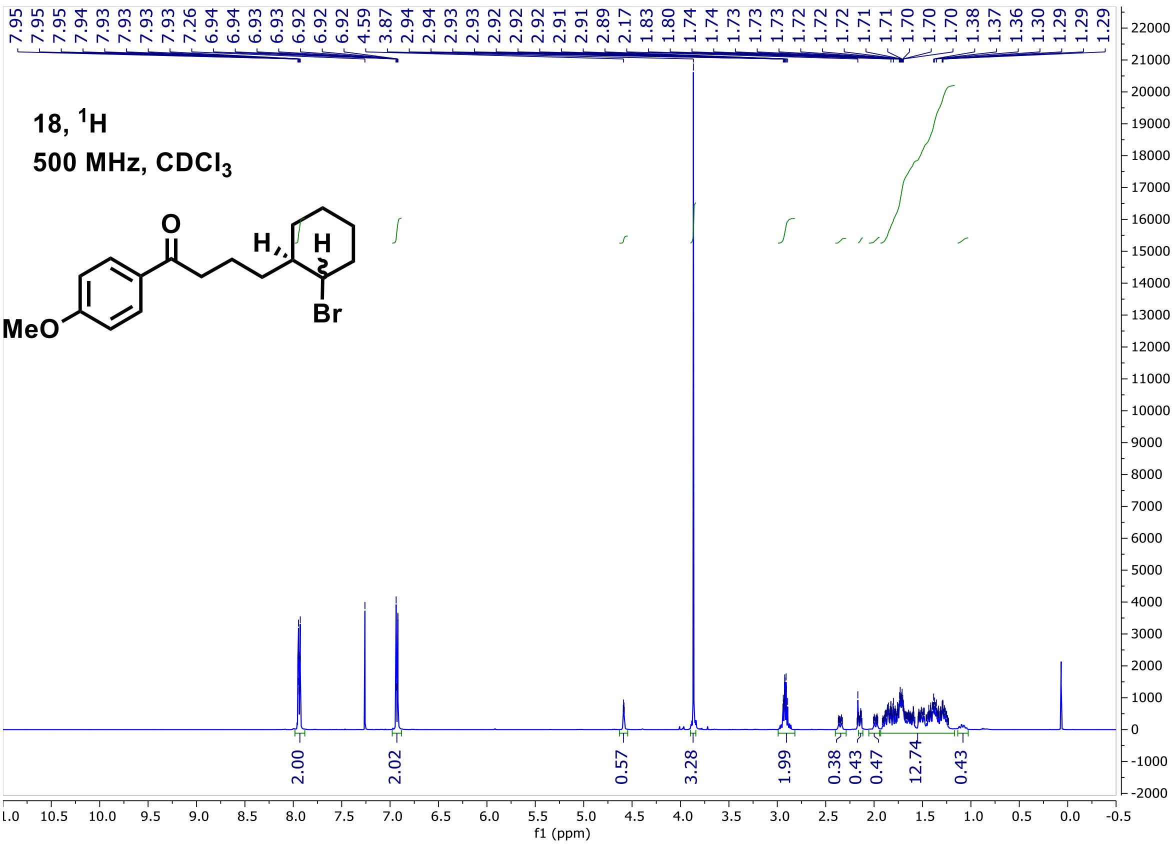
18, ¹H
500 MHz, CDCl3
MeO
O
H
H
Br
2.00
2.02
0.57
3.28
1.99
0.38
0.43
0.47
12.74
0.43
f1 (ppm)

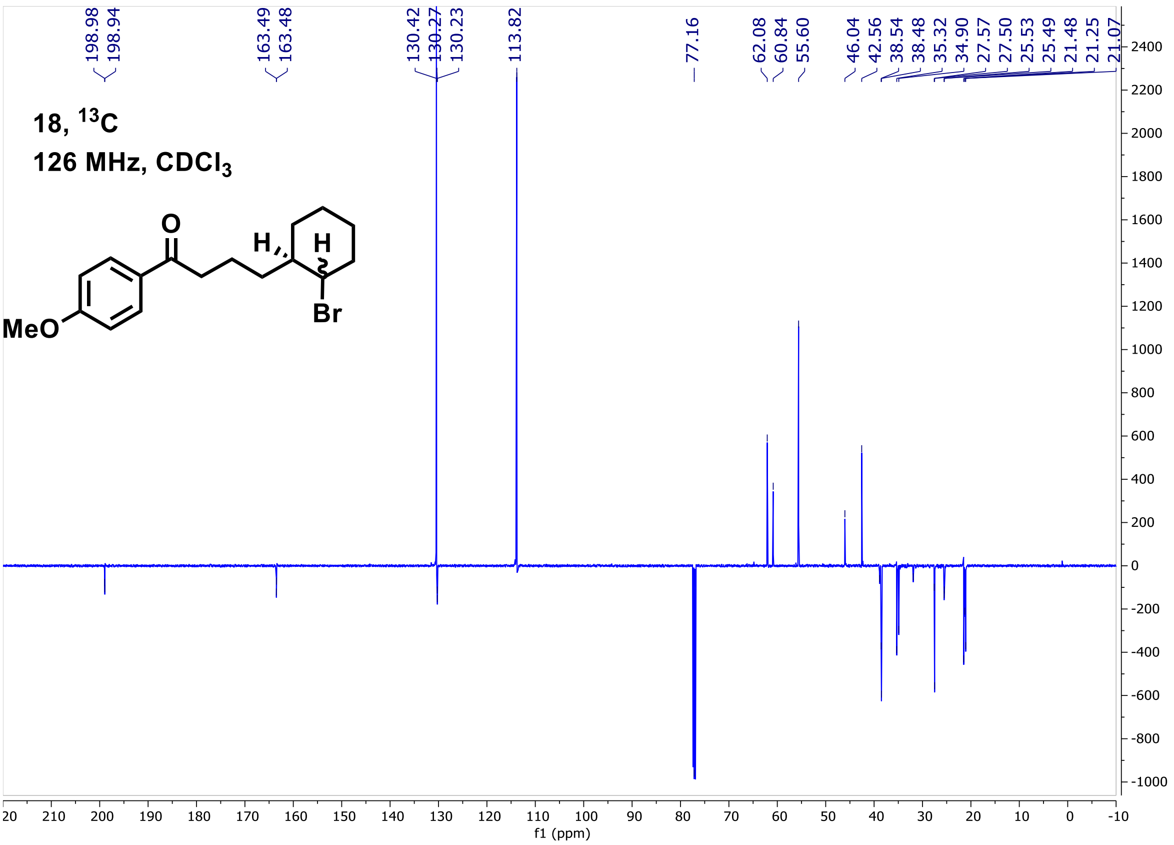
18, ¹³C
126 MHz, CDCl3
MeO
O
H
H
Br
198.98
198.94
163.49
163.48
130.42
130.27
130.23
113.82
77.16
62.08
60.84
55.60
46.04
42.56
38.54
38.48
35.32
34.90
27.57
27.50
25.53
25.49
21.48
21.25
21.07
f1 (ppm)

6-bromo-1-(4-methoxyphenyl)heptan-1-one (19)

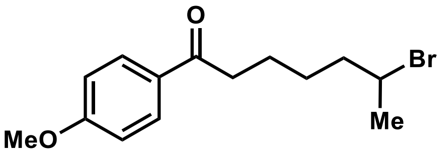

Prepared according to General Procedure (condition F) using **S19** (66 mg, 0.3 mmol). The crude residue was purified by flash column chromatography (2→6% EtOAc/Petrol, silica gel) to afford **19** (60 mg, 66%) as a brown oil.

R_f = 0.22 (10% EtOAc/Petrol); **FTIR** (ν_{max} cm^{-1}, thin film): 3450, 3392, 2983, 2941, 2929, 2918, 2904, 2862, 1690, 1610, 1581; **^{1}H NMR (500 MHz, $CDCl_3$)** δ = 7.99 – 7.88 (m, 2H), 6.98 – 6.88 (m, 2H), 4.12 – 4.15 (m, 1H), 3.87 (d, *J* = 2.2 Hz, 3H), 2.94 (t, *J* = 7.3 Hz, 2H), 1.94 – 1.67 (m, 7H), 1.66 – 1.44 (m, 2H); **^{13}C NMR (126 MHz, $CDCl_3$)** δ = 198.8, 163.5, 130.4, 130.1, 113.8, 55.6, 51.7, 41.1, 38.1, 27.7, 26.6, 23.9; **HRMS** (ES^+/Q-TOF) m/z : [M-Br] Calcd for [$C_{14}H_{19}O_2$] 219.1385; Found 219.1387.

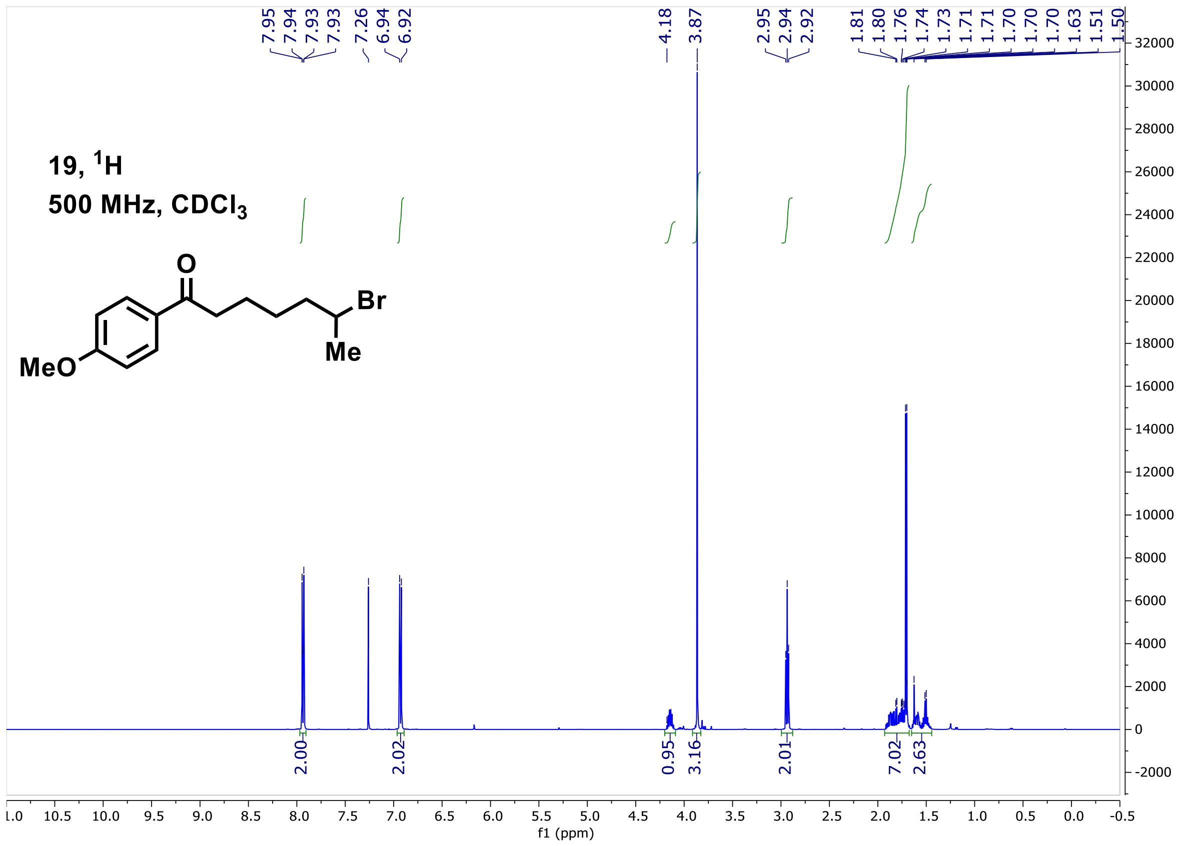
19, 1H
500 MHz, CDCl3
MeO
O
Br
Me
f1 (ppm)

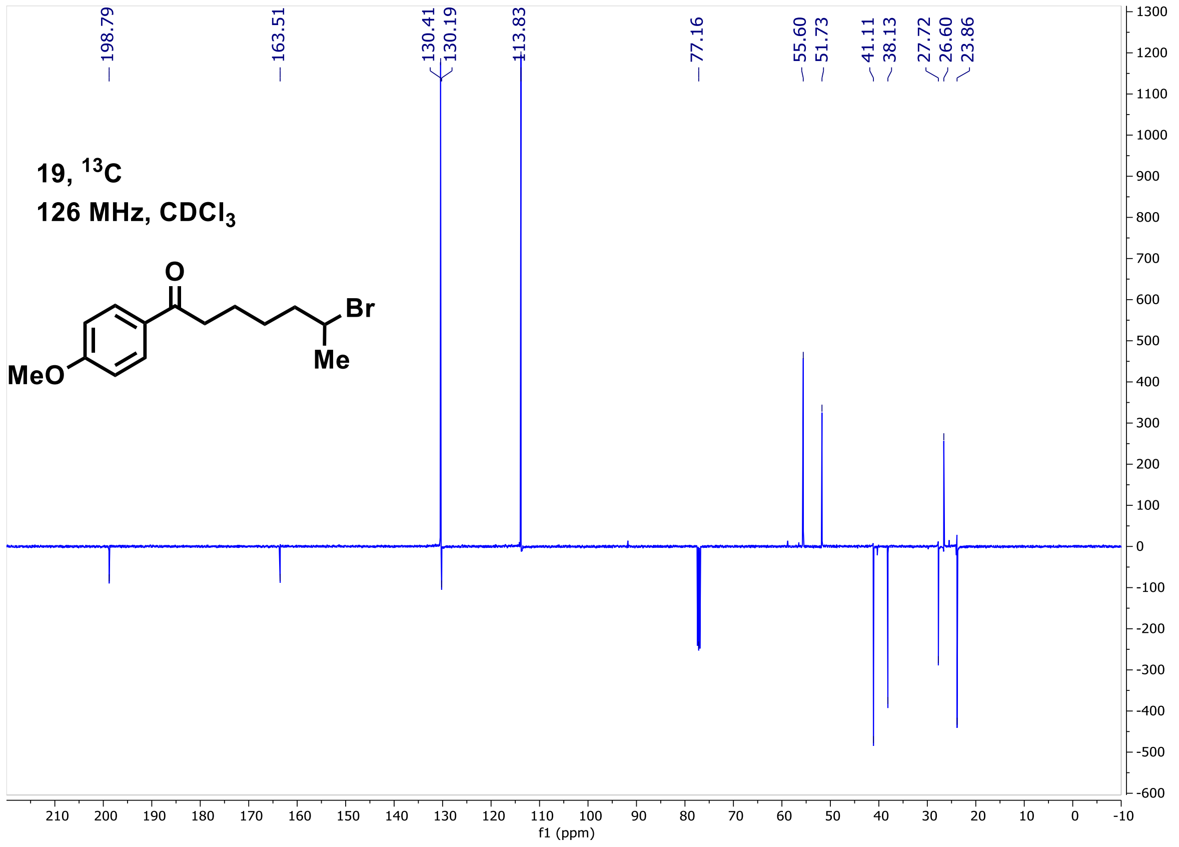
19, 13C
126 MHz, CDCl3
MeO
O
Br
Me
f1 (ppm)

6-bromo-1-(4-methoxyphenyl)-5,5-dimethylhexan-1-one (20)

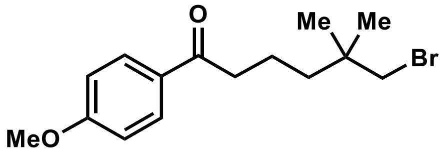

Prepared according to General Procedure (condition G) using **S20** (70 mg, 0.30 mmol). The crude residue was purified by flash column chromatography (2→6% EtOAc/Petrol, silica gel) to afford **20** (65 mg, 69%) as a brown oil.

R_f = 0.25 (10% EtOAc/Petrol); **FTIR** (ν_{max} cm^{-1}, thin film); 3450, 3392, 2983, 2941, 2929, 2918, 2904, 2862, 1678, 1581, 1473, 1217; **^{1}H NMR (500 MHz, $CDCl_3$)** δ = 7.95 (d, *J* = 9.0 Hz, 2H), 6.94 (d, *J* = 8.9 Hz, 2H), 3.87 (s, 3H), 3.31 (s, 2H), 2.92 (t, *J* = 7.4 Hz, 2H), 1.77 – 1.62 (m, 2H), 1.45 – 1.37 (m, 2H), 1.03 (s, 6H); **^{13}C NMR (126 MHz, $CDCl_3$)** δ = 198.9, 163.5, 130.4, 130.2, 113.8, 55.6, 46.7, 39.7, 38.8, 34.8, 25.8, 19.1; **HRMS** (ES^+/Q-TOF) m/z : [M-Br+H] Calcd for [$C_{15}H_{22}O_2$] 234.1614; Found 234.1617.

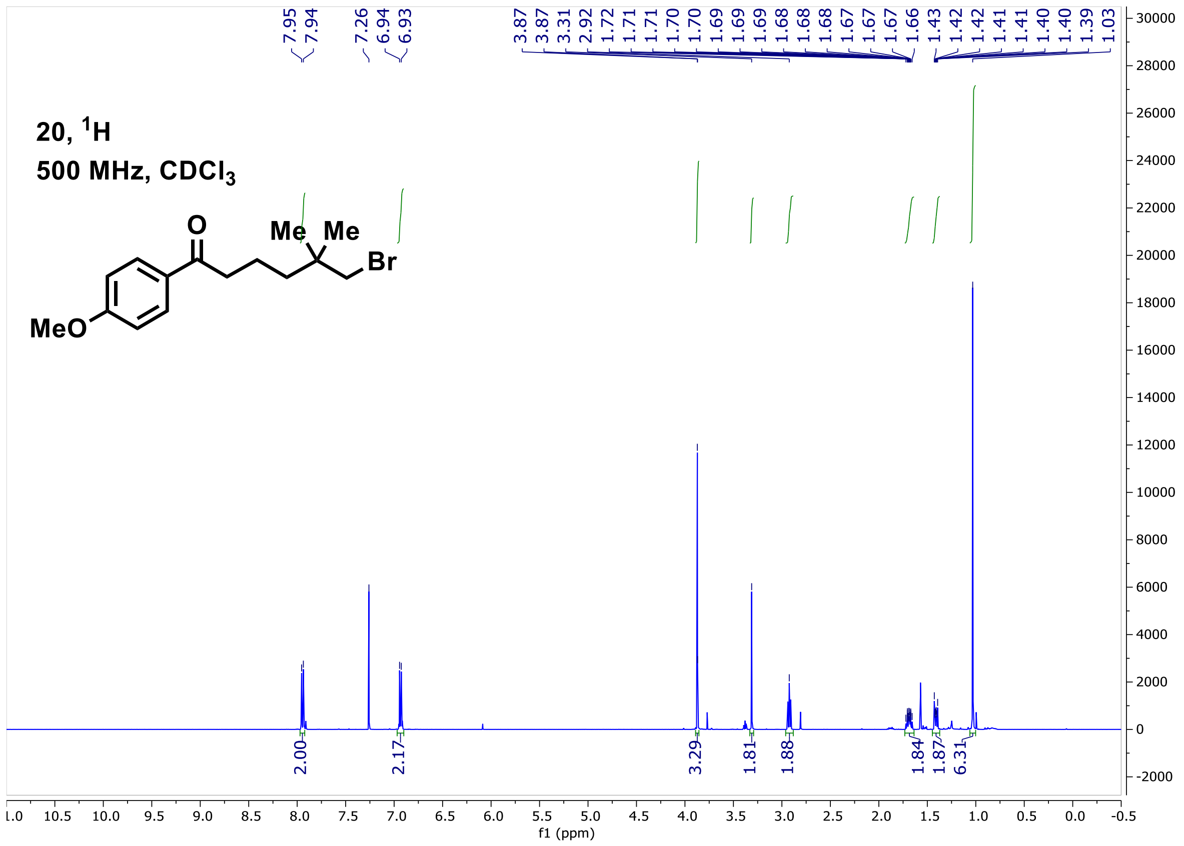

20, 1H
500 MHz, CDCl3
Me Me
O
Br
MeO
f1 (ppm)

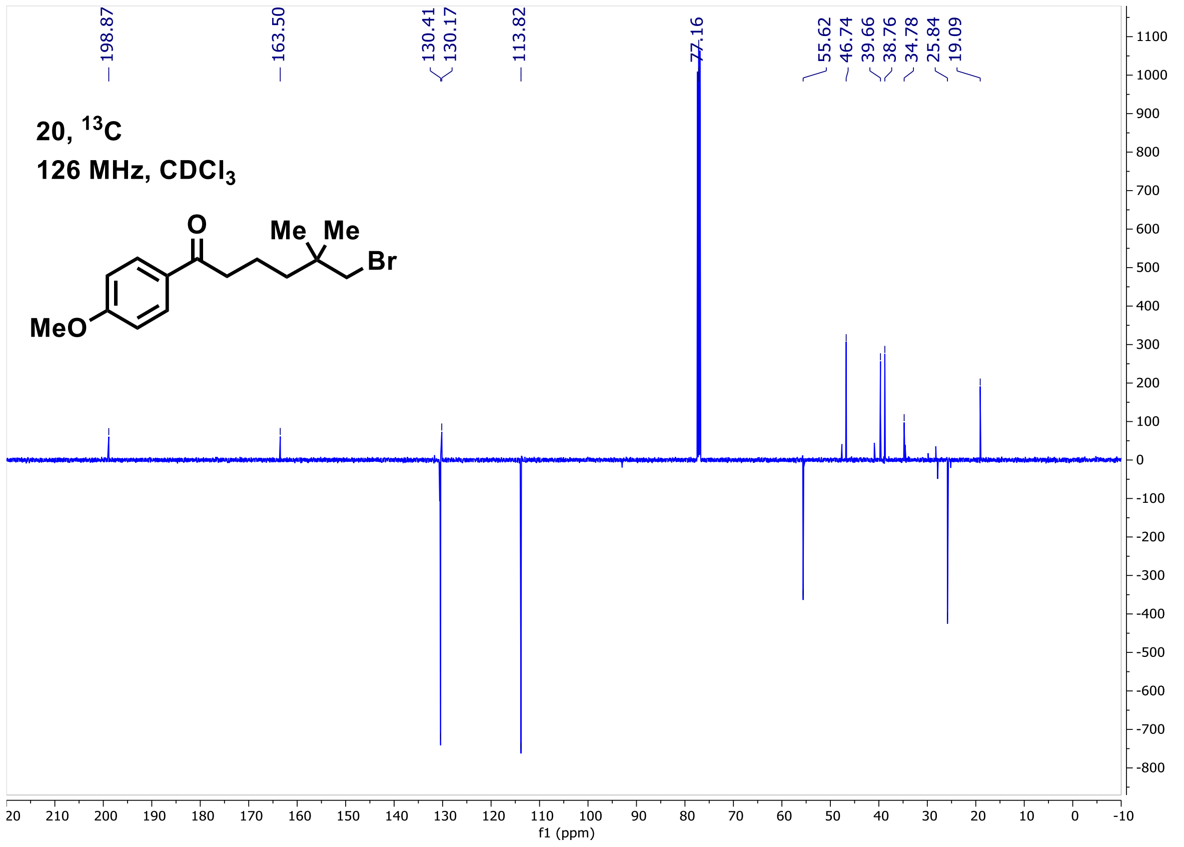

20, 13C
126 MHz, CDCl3
Me Me
O
Br
MeO
f1 (ppm)

6-bromo-4,4-difluoro-1-(4-methoxyphenyl)hexan-1-one (21)

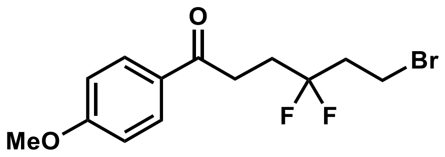

Prepared according to General Procedure (condition F) using **S21** (73 mg, 0.3 mmol). The crude residue was purified by flash column chromatography (6→8% EtOAc/Petrol, silica gel) to afford **21** (80 mg, 83%) as a yellow oil.

R_f = 0.29 (12% EtOAc/Petrol); **FTIR** (ν_{max} cm^{-1}, thin film); 2968, 2949, 2929, if1676, 1597, 1460, 1367, 1303, 1184, 1055; **^{1}H NMR (500 MHz, $CDCl_3$)** δ = 8.00 – 7.93 (m, 2H), 6.97 – 6.91 (m, 2H), 3.88 (s, 3H), 3.53 – 3.46 (m, 2H), 3.20 – 3.14 (m, 2H), 2.56 – 2.43 (m, 2H), 2.43 – 2.27 (m, 2H); **^{13}C NMR (126 MHz, $CDCl_3$)** δ = 196.4, 163.9, 130.5, 129.6, 123.6 (t, *J* = 242.1 Hz), 114.0, 55.7, 40.8 (t, *J* = 25.5 Hz), 31.1 (t, *J* = 24.6 Hz), 30.6 (t, *J* = 3.7 Hz), 23.5 (t, *J* = 5.9 Hz); **^{19}F NMR (471 MHz, CDCl3) δ =** -100.1 (m); **HRMS** (ES^+/Q-TOF) m/z : [M+H] Calcd for [$C_{13}H_{16}O_2F_2Br$] 321.0302; Found 321.0300.

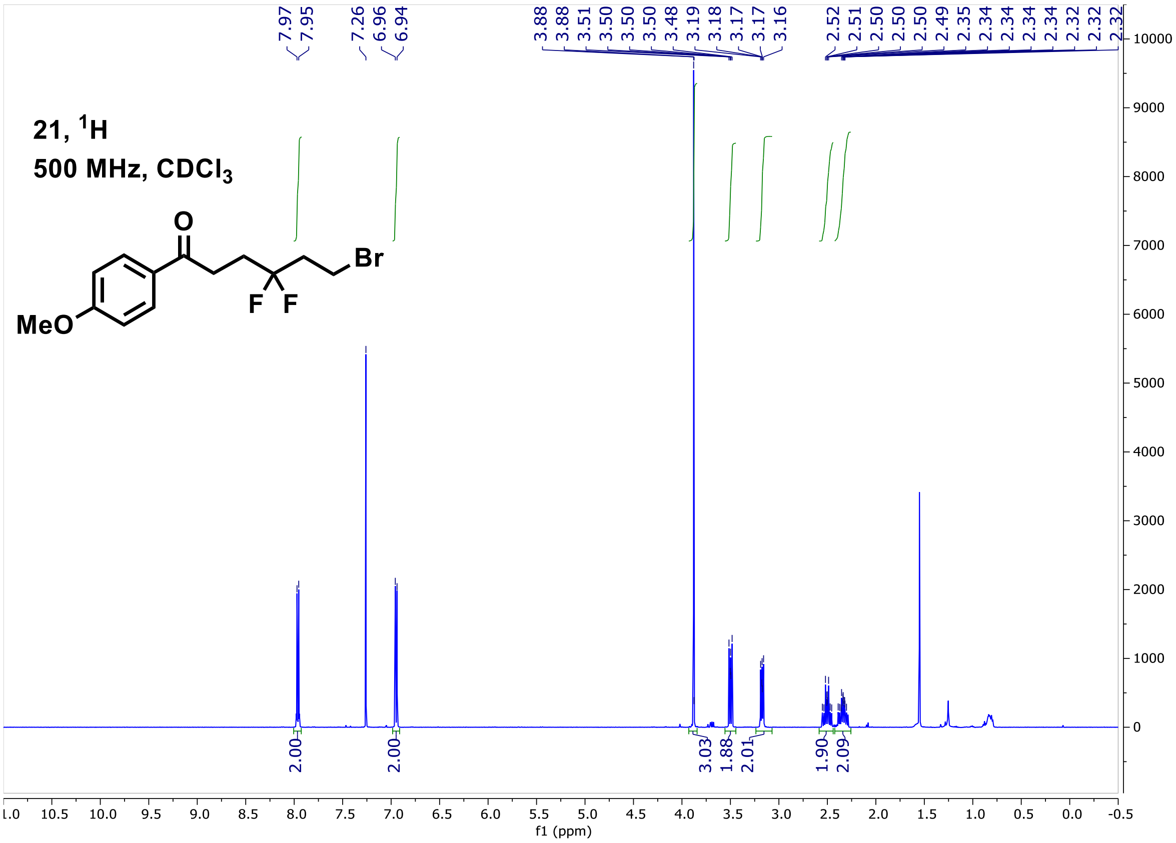

21, 1H
500 MHz, CDCl3
MeO
O
Br
F F
7.97
7.95
7.26
6.96
6.94
3.88
3.88
3.51
3.50
3.50
3.50
3.48
3.19
3.18
3.17
3.17
3.16
2.52
2.51
2.50
2.50
2.50
2.49
2.35
2.34
2.34
2.34
2.34
2.32
2.32
2.32
2.00
2.00
3.03
1.88
2.01
1.90
2.09
f1 (ppm)

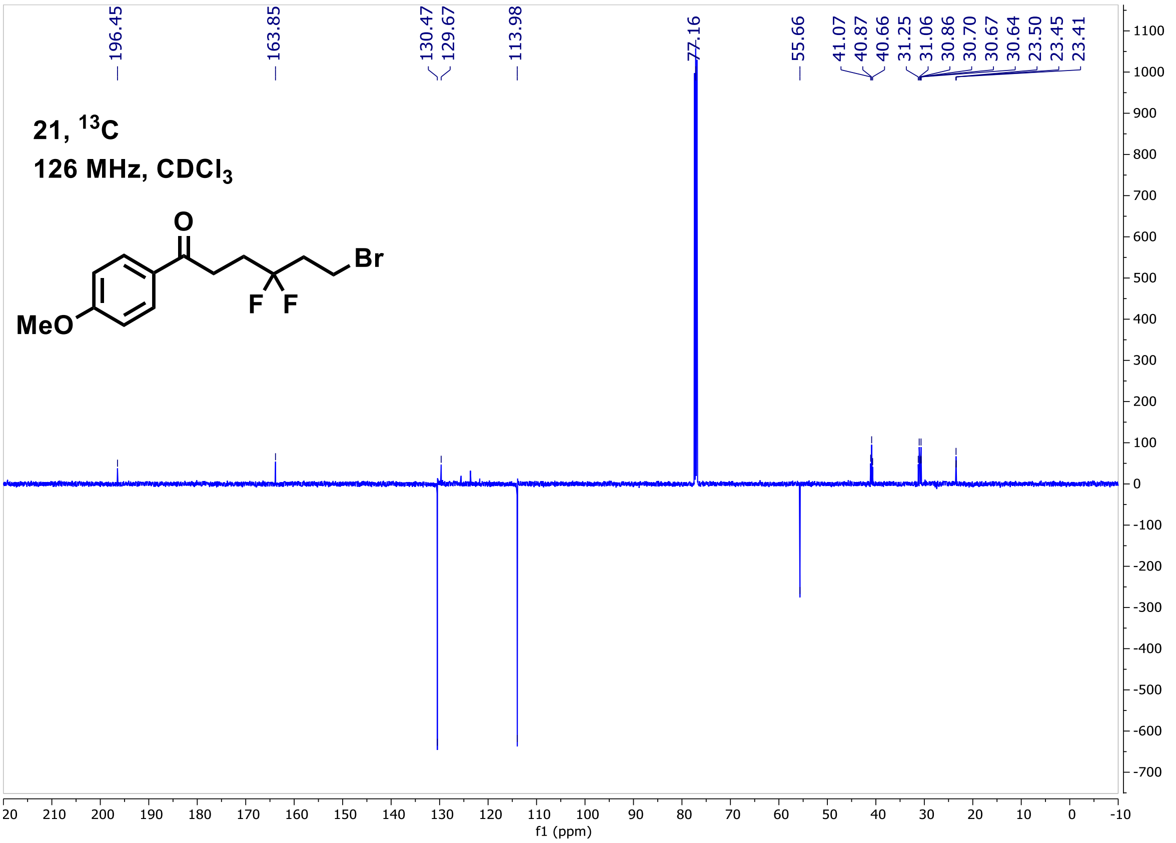

21, 13C
126 MHz, CDCl3
MeO
O
Br
F F
196.45
163.85
130.47
129.67
113.98
77.16
55.66
41.07
40.87
40.66
31.25
31.06
30.86
30.70
30.67
30.64
23.50
23.45
23.41
f1 (ppm)

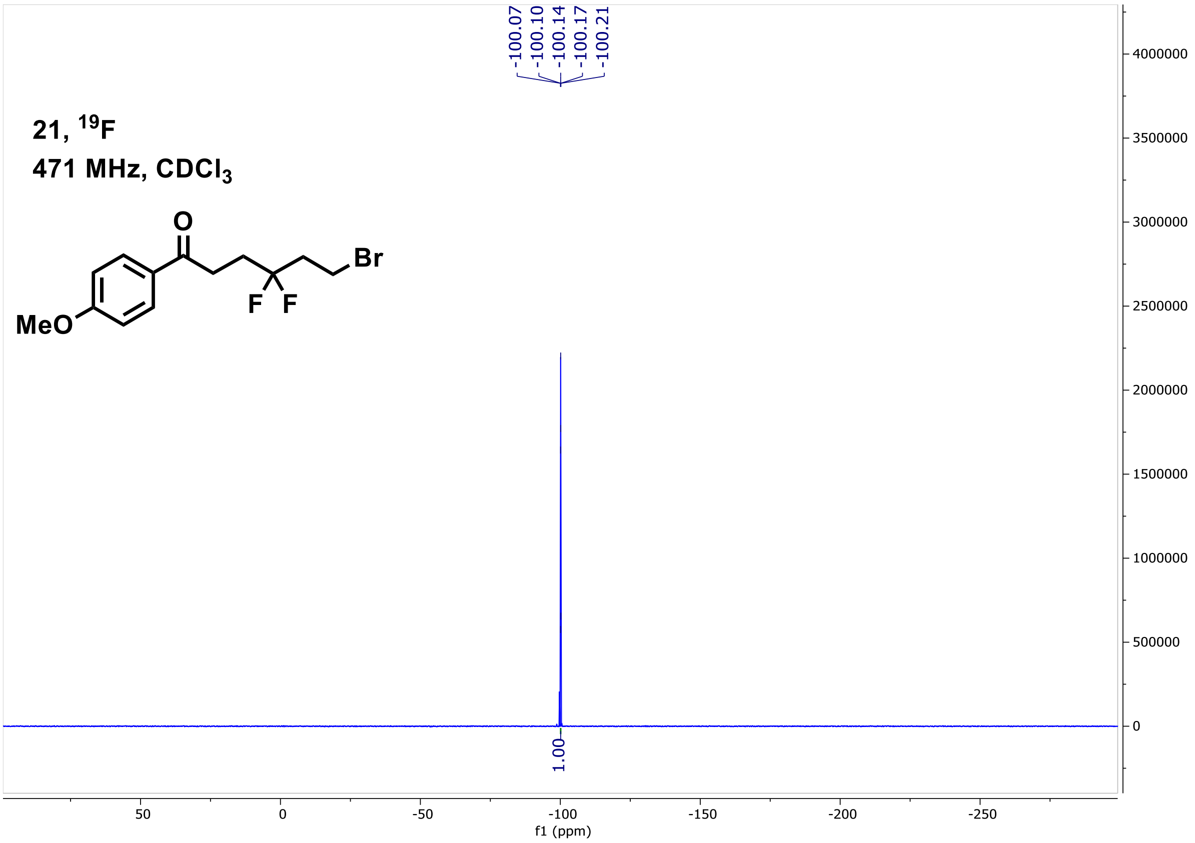
-100.07
-100.10
-100.14
-100.17
-100.21
21, 19F
471 MHz, CDCl3
O
Br
F F
MeO
1.00
4000000
3500000
3000000
2500000
2000000
1500000
1000000
500000
0
50
0
-50
-100
-150
-200
-250
f1 (ppm)

3-(2-(2-bromoethyl)-5,5-dimethyl-1,3-dioxan-2-yl)-1-(4-methoxyphenyl)propan-1-one (22)

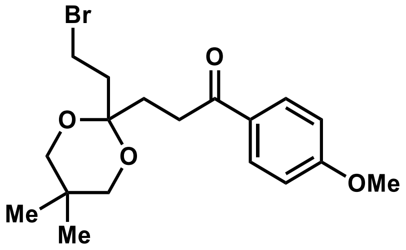

Prepared according to General Procedure (condition B) using **S22** (92 mg). The crude was purified by flash column chromatography (5→10% EtOAc/Petrol, silica gel) to afford **22** (106 mg, 93%) as a colourless oil.

R_f = 0.48 (15% EtOAc/Petrol); **FTIR** (ν_{max} cm^{-1}, thin film) = 2951, 2870, 1678, 1599, 1512, 1465, 1320, 1259, 1172, 1122, 1028, 837; **^{1}H NMR (500 MHz, $CDCl_3$)** δ = 7.96 (d, *J* = 8.9 Hz, 2H), 6.94 (d, *J* = 8.9 Hz, 2H), 3.87 (s, 3H), 3.63 – 3.39 (m, 6H), 3.13 – 2.96 (m, 2H), 2.41 – 2.28 (m, 2H), 2.21 – 2.12 (m, 2H), 0.98 (s, 3H), 0.93 (s, 3H); **^{13}C NMR (126 MHz, $CDCl_3$)** δ = 198.2, 163.6, 130.4, 130.1, 113.8, 99.5, 70.3, 55.6, 39.2, 32.3, 29.7, 27.8, 27.4, 22.9, 22.7; **HRMS HRMS** (ES$^+$/Q-TOF) m/z : [M+H] Calcd for [$C_{18}H_{26}O_4Br$] 385.1014; Found 385.1016.

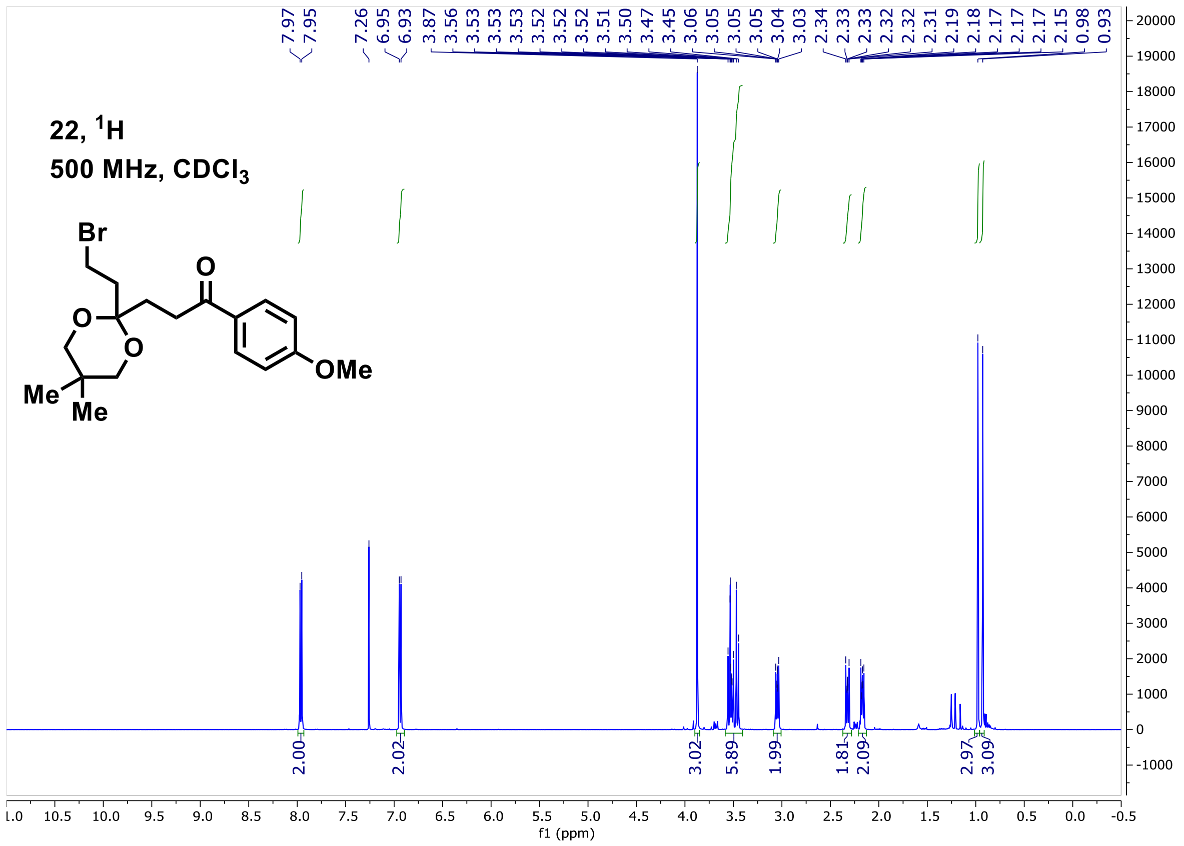
22, 1H
500 MHz, CDCl3
f1 (ppm)

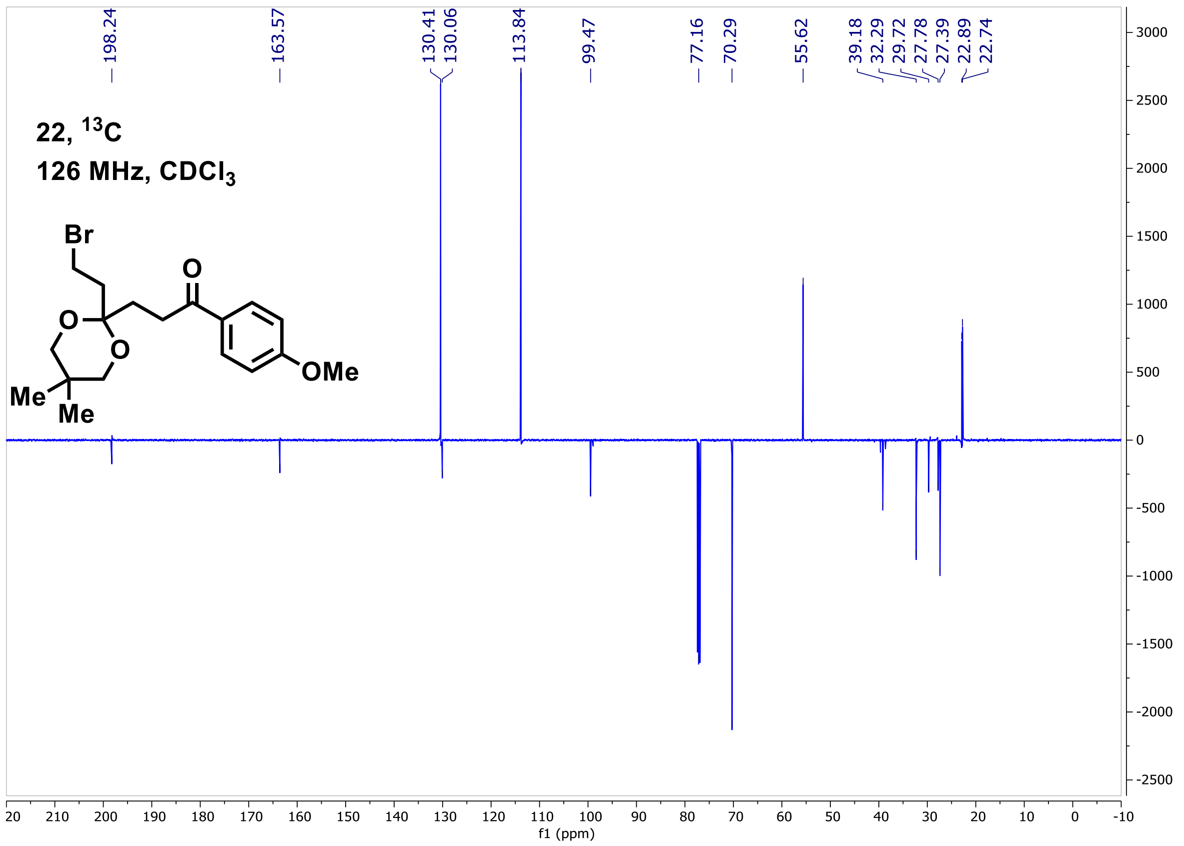
22, 13C
126 MHz, CDCl3
f1 (ppm)

(2-(3-bromopropyl)phenyl)(4-methoxyphenyl)methanone (23)

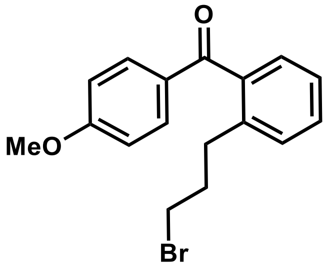

Prepared according to General Procedure (condition F) using **S23** (76 mg, 0.3 mmol). The crude residue was purified by flash column chromatography (5→10% EtOAc/Petrol, silica gel) to afford **23** (60 mg, 60%) as a yellow oil.

R_f = 0.22 (10% EtOAc/Petrol); **FTIR** (ν_{max} cm^{-1}, thin film): 3450, 3392, 2983, 2941, 2929, 2918, 2904, 2862, 1692, 1581; **^{1}H NMR (500 MHz, $CDCl_3$)** δ = 7.83 – 7.73 (m, 2H), 7.44 – 7.38 (m, 1H), 7.38 – 7.31 (m, 1H), 7.31 – 7.23 (m, 2H), 6.98 – 6.88 (m, 2H), 3.88 (s, 3H), 3.34 (t, *J* = 6.6 Hz, 2H), 2.79 (t, *J* = 8.3 Hz, 2H), 2.18 – 2.06 (m, 2H); **^{13}C NMR (126 MHz, $CDCl_3$)** δ = 197.2, 164.0, 139.4, 139.2, 132.7, 130.7, 130.4, 130.1, 128.5, 125.8, 113.9, 55.7, 34.6, 33.4, 31.8; **HRMS** (ES^+/Q-TOF) m/z : [M+H] Calcd for [$C_{17}H_{18}O_2Br$] 333.0490; Found 333.0495.

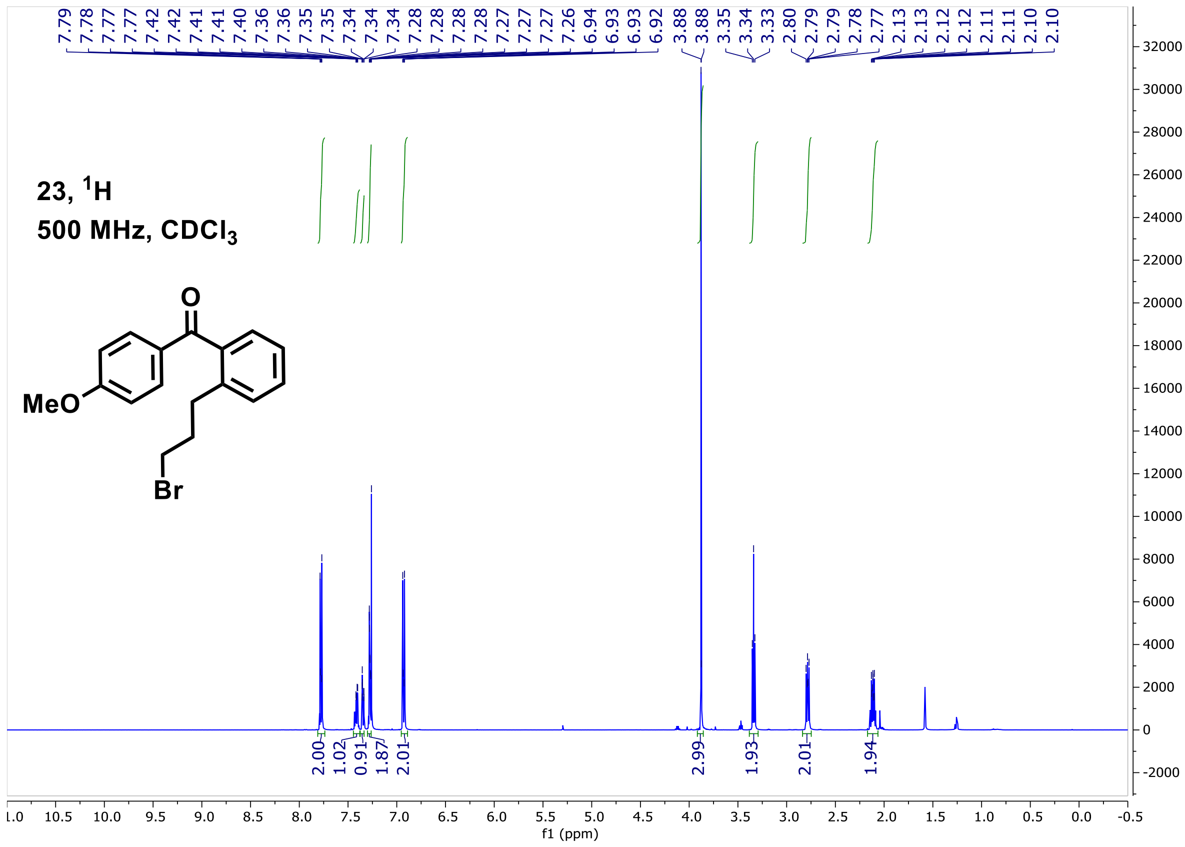
7.79 7.78 7.77 7.77 7.42 7.42 7.41 7.41 7.40 7.36 7.36 7.35 7.34 7.34 7.34 7.28 7.28 7.28 7.28 7.27 7.27 7.27 7.26 6.94 6.93 6.93 6.92 3.88 3.88 3.35 3.34 3.33 2.80 2.79 2.79 2.78 2.77 2.13 2.13 2.12 2.12 2.11 2.10 2.10
23, 1H
500 MHz, CDCl3
O
MeO
Br
2.00 1.02 0.91 1.87 2.01 2.99 1.93 2.01 1.94
f1 (ppm)

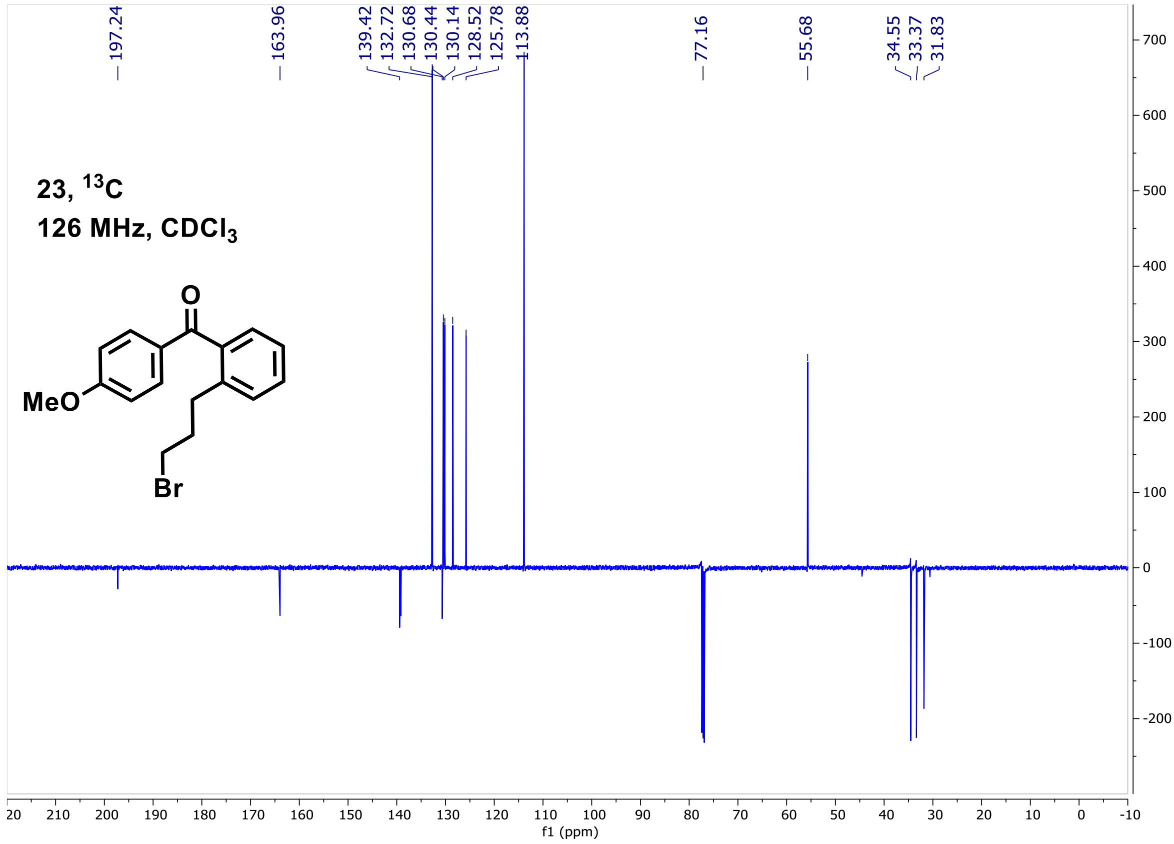
197.24 163.96 139.42 132.72 130.68 130.44 130.14 128.52 125.78 113.88 77.16 55.68 34.55 33.37 31.83
23, 13C
126 MHz, CDCl3
O
MeO
Br
f1 (ppm)

tert-butyl (2-bromoethyl)(3-(4-methoxyphenyl)-3-oxopropyl)carbamate (24)

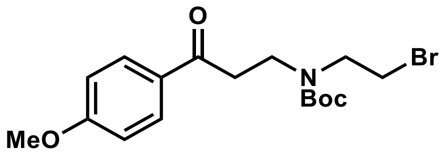

Prepared according to General Procedure (condition B) using **S24** (92 mg, 0.3 mmol). The crude residue was purified by flash column chromatography (18→20% EtOAc/Petrol, silica gel) to afford **24** (104 mg, 72%) as a brown oil.

R_f = 0.28 (25% EtOAc/Petrol); **FTIR** (ν_{max} cm^{-1}, thin film); 2972, 2328, 1687, 1600, 1510, 1367, 1257, 1168, 833; **^1H NMR (500 MHz, $CDCl_3$)** δ = 7.98 – 7.89 (m, 2H), 6.93 – 6.91 (m, 2H), 3.85 (s, 3H), 3.59 (t, *J* = 7.0 Hz, 2H), 3.40 – 3.33 (m, 4H), 3.25 – 3.11 (m, 2H), 1.44 (s, 9H); **^{13}C NMR (126 MHz, $CDCl_3$)** δ = 197.7, 163.7, 155.5, 130.5, 130.0, 113.9, 80.0, 55.6, 46.9, 43.9, 37.4, 32.1, 28.5; **HRMS** (CI^+/FTMS) m/z : [M-Br+H] Calcd for [$C_{17}H_{25}O_4N$] 307.1784; Found 307.1780.

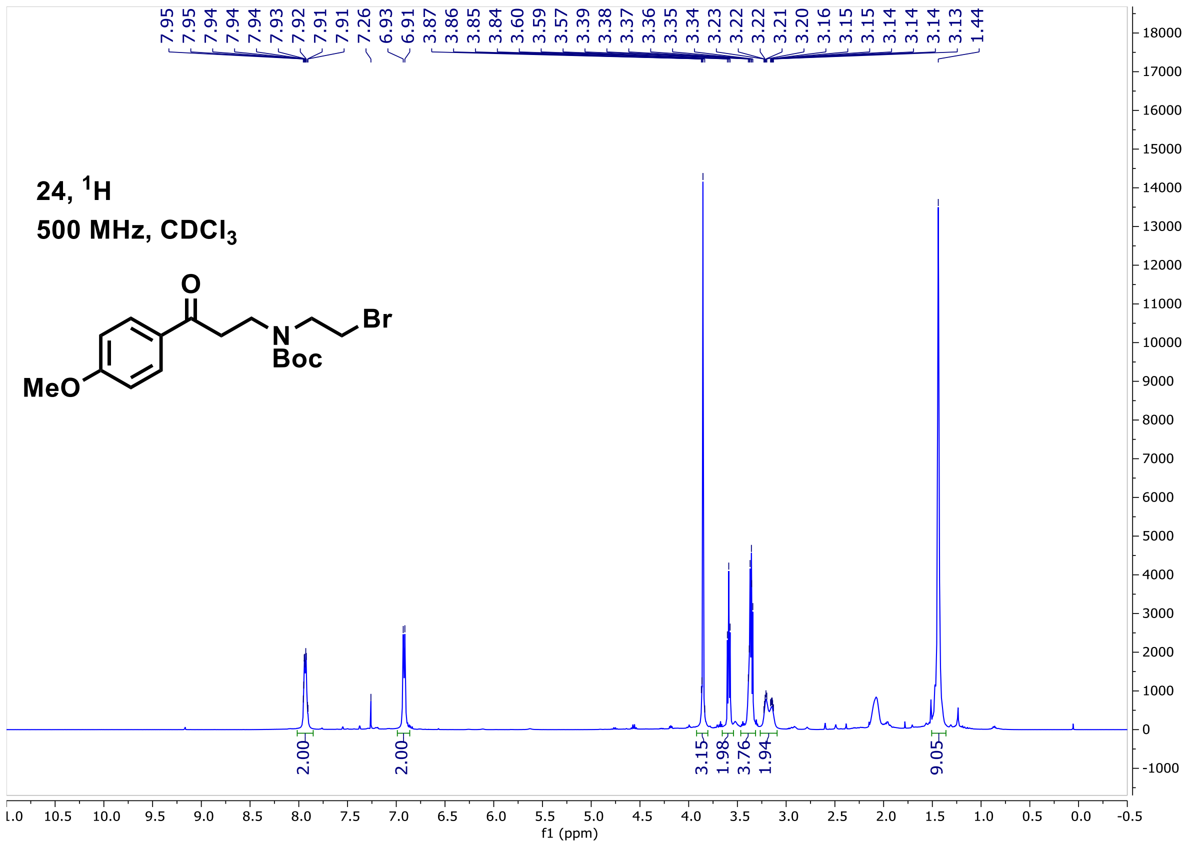
24, 1H
500 MHz, CDCl3
MeO
O
N
Boc
Br
f1 (ppm)

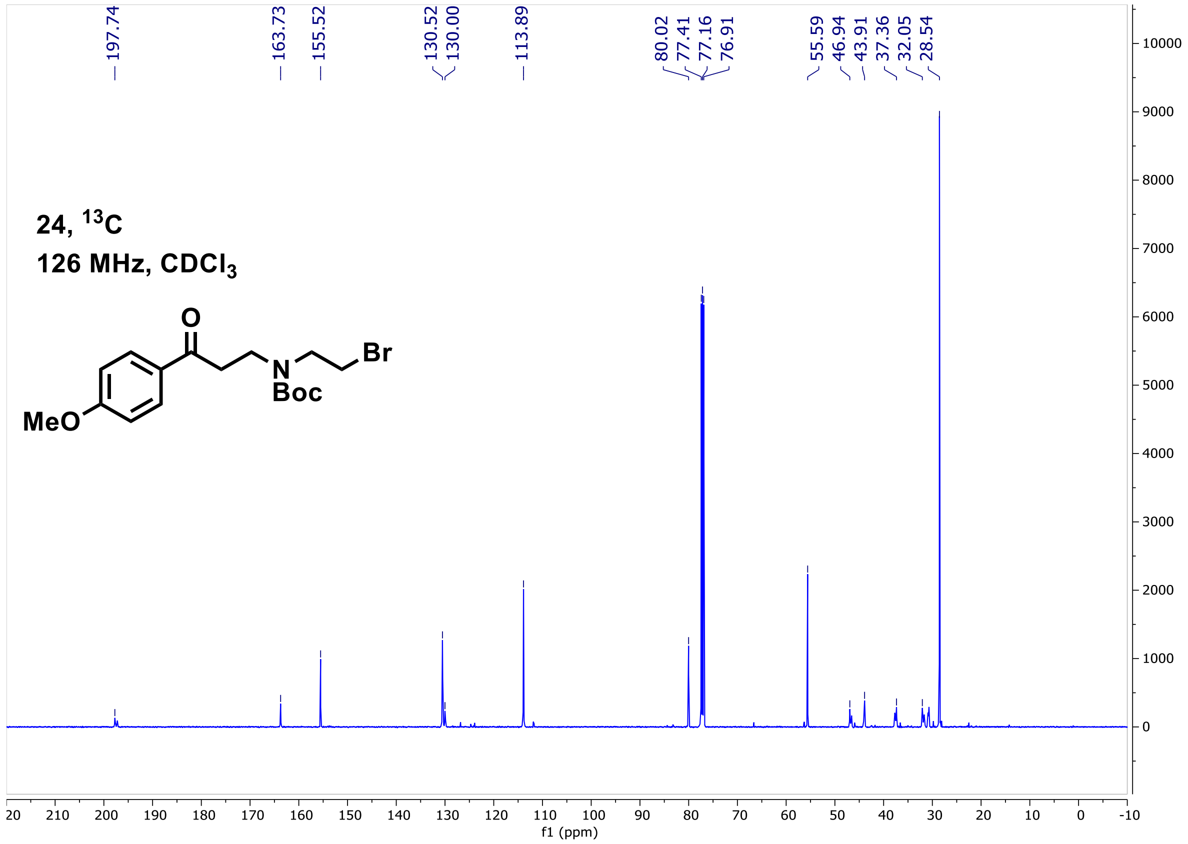
24, 13C
126 MHz, CDCl3
MeO
O
N
Boc
Br
f1 (ppm)

tert-butyl (bromomethyl)(4-(4-methoxyphenyl)-4-oxobutyl)carbamate (25)

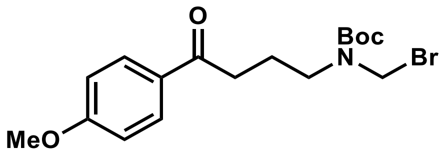

Prepared according to General Procedure (condition H) using **S25** (92 mg, 0.3 mmol). The crude residue was purified by flash column chromatography (20→23% EtOAc/Petrol, silica gel) to afford **25** (104 mg, 90%) as a yellow oil.

R_f = 0.28 (25% EtOAc/Petrol); **FTIR** (ν_{max} cm^{-1}, thin film) 2935, 1697, 1598, 1415, 1367, 1257, 1029, 829; **^{1}H NMR (400 MHz, $CDCl_3$)** δ = 7.93 (d, *J* = 8.5 Hz, 2H), 6.92 (d, *J* = 8.4 Hz, 2H), 4.87 (s, 2H), 3.86 (s, 3H), 3.38 (m, 2H), 2.93 (t, *J* = 7.1 Hz, 2H), 1.99 (m, 2H), 1.43 (s, 9H); **^{13}C NMR (126 MHz, $CDCl_3$)** δ = 206.2, 163.2, 158.8, 132.4, 115.9, 112.9, 80.6, 55.6, 39.8, 38.5, 28.5, 28.2, 22.5; **HRMS** (ES^+/Q-TOF) m/z : [M-Br] Calcd for [$C_{17}H_{24}NO_4$]306.1705; Found 306.1703.

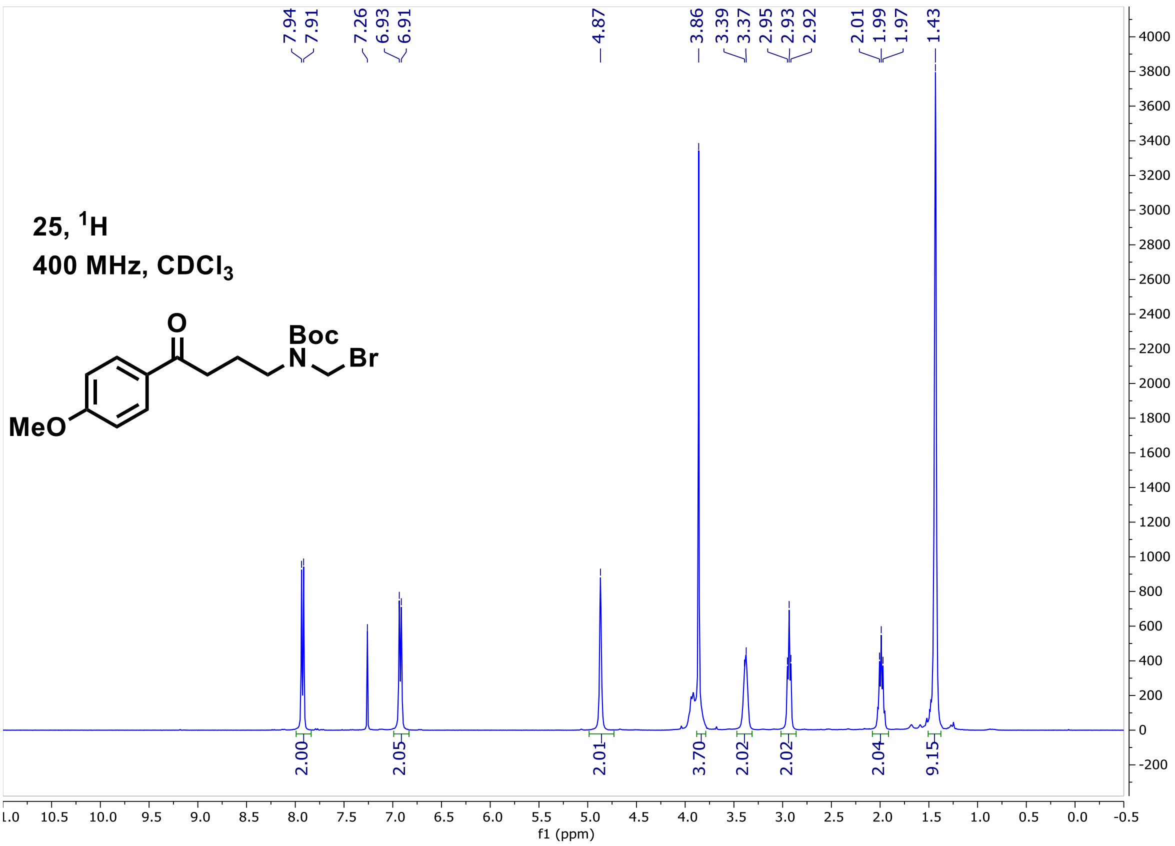

25, ^{1}H
400 MHz, $CDCl_3$
Boc
N
Br
O
MeO
7.94
7.91
7.26
6.93
6.91
4.87
3.86
3.39
3.37
2.95
2.93
2.92
2.01
1.99
1.97
1.43
2.00
2.05
2.01
3.70
2.02
2.02
2.04
9.15
f1 (ppm)

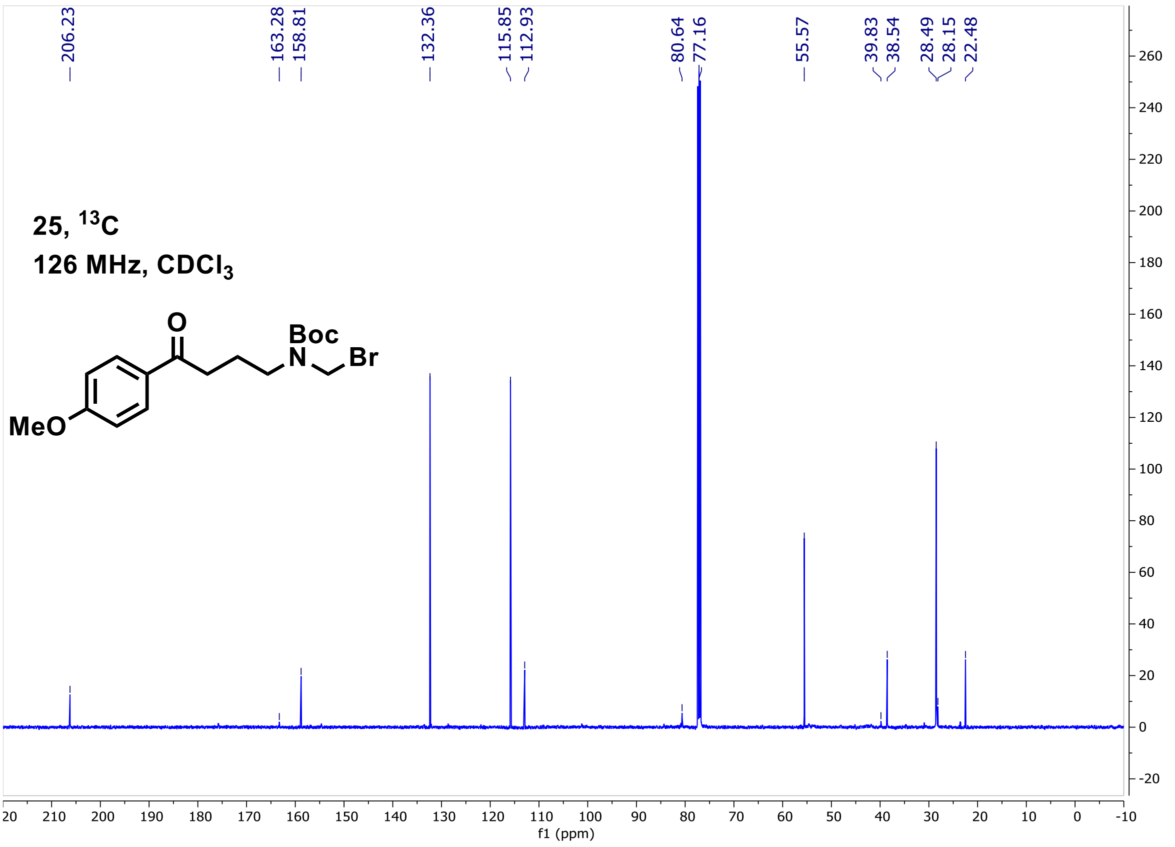

25, ^{13}C
126 MHz, $CDCl_3$
Boc
N
Br
O
MeO
206.23
163.28
158.81
132.36
115.85
112.93
80.64
77.16
55.57
39.83
38.54
28.49
28.15
22.48
f1 (ppm)

tert-butyl (2-bromoethyl)(4-(4-methoxyphenyl)-4-oxobutyl)carbamate (26)

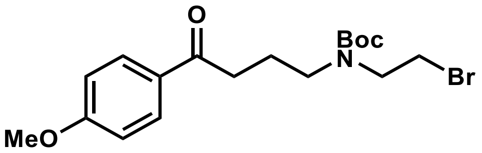

Prepared according to General Procedure (condition B) using **S26** (96 mg, 0.3 mmol). The crude residue was purified by flash column chromatography (18→22% EtOAc/Petrol, silica gel) to afford **26** (94 mg, 78%) as an orange oil.

R_f = 0.28 (25% EtOAc/Petrol); **FTIR** (ν_{max} cm^{-1}, thin film); 2972, 2848, 1681, 1598, 1575, 1510, 1467, 1415, 1365, 1255, 1166, 1029, 839; **^{1}H NMR (400 MHz, $CDCl_3$)** δ = 7.95 (d, *J* = 8.3 Hz, 2H), 6.94 (d, *J* = 8.4 Hz, 2H), 3.87 (s, 3H), 3.60 (t, *J* = 7.0 Hz, 2H), 3.42 – 3.34 (m, 4H), 3.25 – 3.13 (m, 2H), 2.10 (s, 2H), 1.45 (s, 9H) ; **^{13}C NMR (101 MHz, $CDCl_3$)** δ = 196.0, 163.8, 155.6, 130.6, 130.0, 113.9, 80.1, 55.6, 47.0, 46.6, 44.0, 37.7, 37.4, 28.6; **HRMS** (ES^+/Q-TOF) m/z : [M-H] Calcd for [$C_{18}H_{25}NO_4Br$] 398.0967; Found 398.0969.

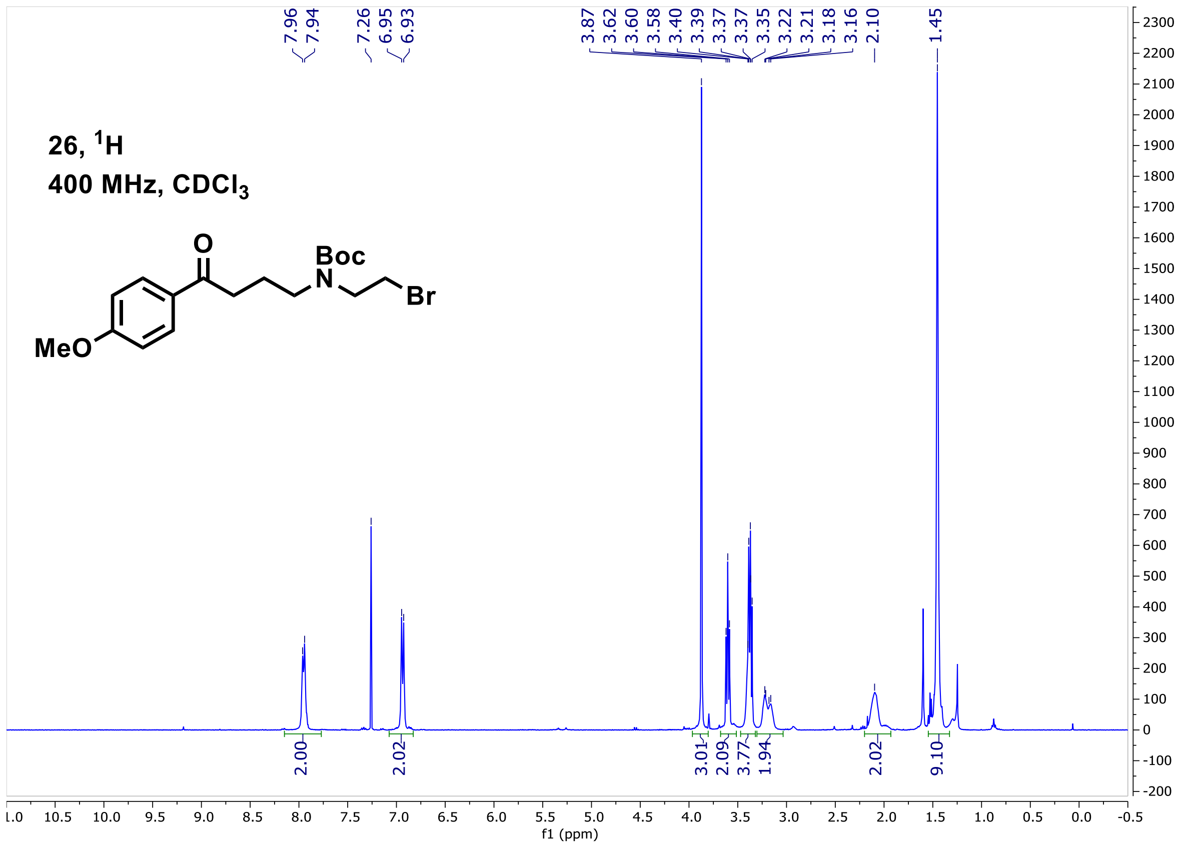
26, ^{1}H
400 MHz, $CDCl_3$
MeO
O
Boc
N
Br
f1 (ppm)

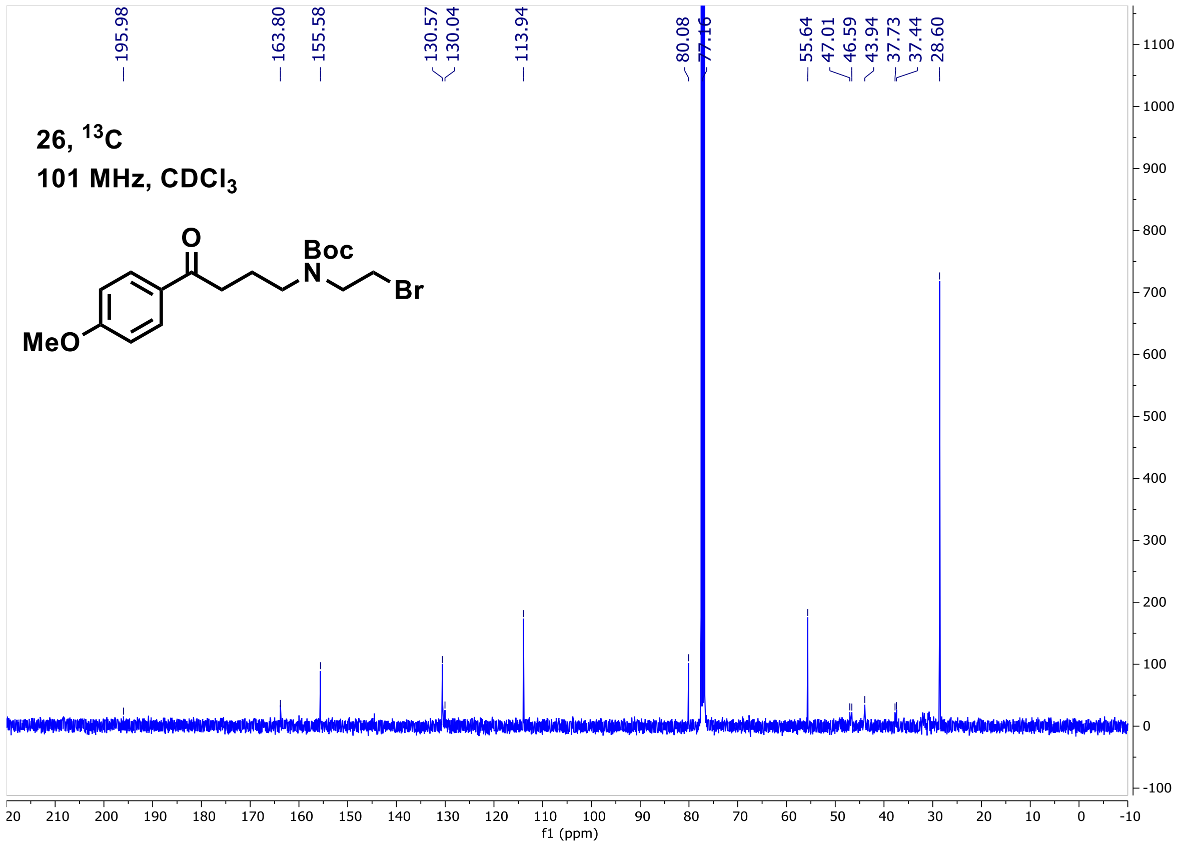
26, ^{13}C
101 MHz, $CDCl_3$
MeO
O
Boc
N
Br
f1 (ppm)

1-(4-methoxyphenyl)ethan-1-one (27)

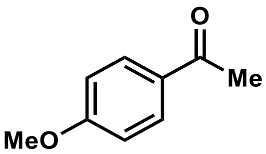

Prepared according to General Procedure (condition H) using **S27** (72.7 mg, 0.3 mmol). The crude residue was purified by flash column chromatography (7→10% EtOAc/Petrol, silica gel) to afford **27** (38 mg, 85%) as an off white solid.

R_f = 0.27 (10% EtOAc/Petrol); **M.p.:** 38-40 °C (lit.[16] 38-39); **^{1}H NMR (500 MHz, $CDCl_3$)** δ = 7.98 – 7.89 (m, 2H), 6.99 – 6.86 (m, 2H), 3.87 (s, 3H), 2.56 (s, 3H); **^{13}C NMR (126 MHz, $CDCl_3$)** δ = 196.9, 163.6, 130.7, 130.5, 113.8, 55.6, 26.5.

These data are consistent with those previously reported in the literature.[17]

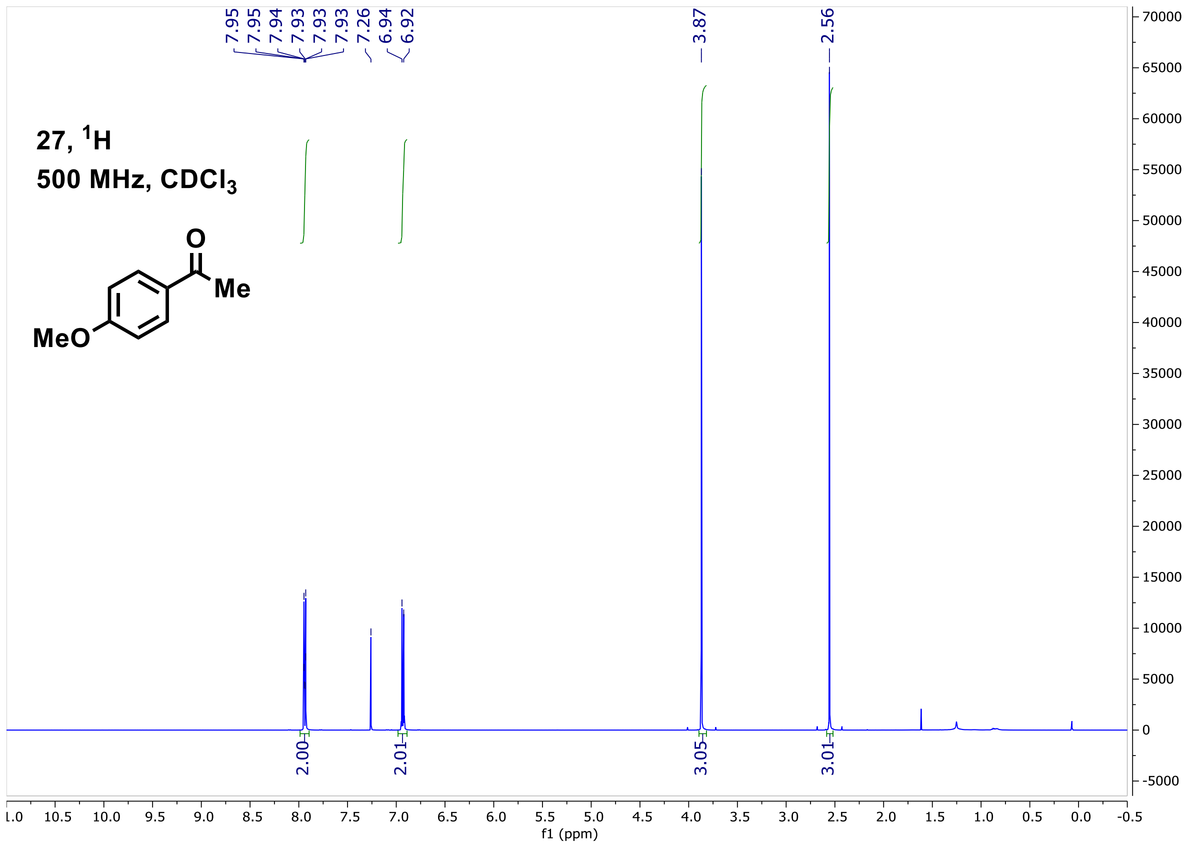
27, ^{1}H
500 MHz, $CDCl_3$
MeO
Me
O
7.95
7.95
7.94
7.93
7.93
7.93
7.26
6.94
6.92
3.87
2.56
2.00
2.01
3.05
3.01
f1 (ppm)

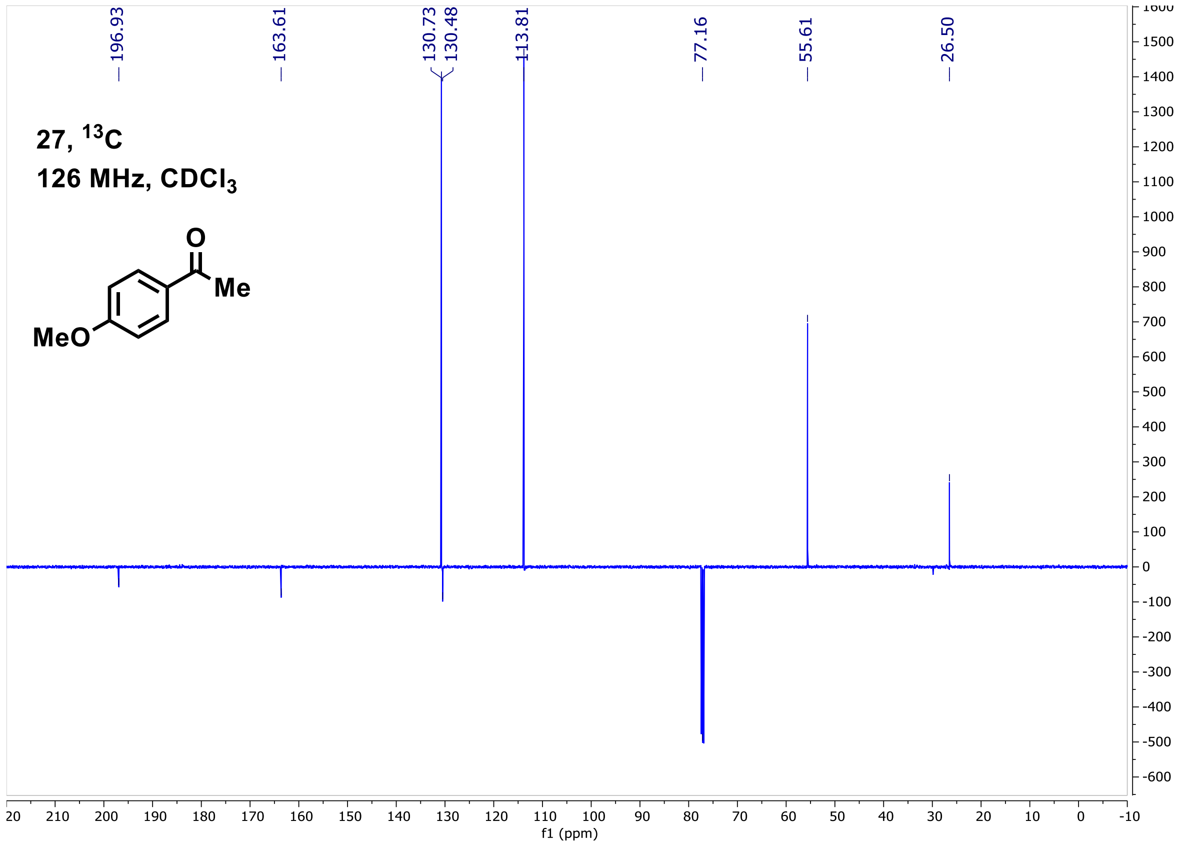
27, ^{13}C
126 MHz, $CDCl_3$
MeO
Me
O
196.93
163.61
130.73
130.48
113.81
77.16
55.61
26.50
f1 (ppm)

6-chloro-1-(4-methoxyphenyl)hexan-1-one (28)

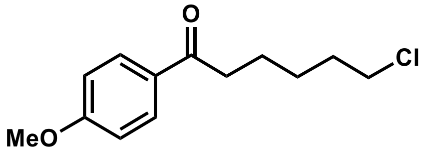

Prepared according to General Procedure (condition I) using **1** (62.0 mg, 0.3 mmol). ^{1}H NMR analysis of the crude reaction mixture showed 40% conversion. The crude residue was purified by flash column chromatography (2→5% EtOAc/Petrol, silica gel) to afford **28** (16 mg, 22%) as a yellow oil.

R_f = 0.20 (10% EtOAc/Petrol); **^{1}H NMR (500 MHz, $CDCl_3$)** δ = 7.94 (d, *J* = 8.9 Hz, 2H), 6.93 (d, *J* = 8.9 Hz, 2H), 3.87 (s, 3H), 3.55 (t, *J* = 6.7 Hz, 2H), 2.94 (t, *J* = 7.3 Hz, 2H), 2.14 – 1.61 (m, 4H), 1.61 – 1.40 (m, 2H); **^{13}C NMR (126 MHz, $CDCl_3$)** δ =198.8, 163.5, 130.4, 130.2, 113.9, 55.6, 45.1, 38.1, 32.6, 26.8, 23.8; **HRMS** (ES^+/Q-TOF) m/z : [M+H] Calcd for [$C_{13}H_{18}O_2Cl$] 241.0995; Found 241.0998

These data are consistent with those previously reported in the literature.[8]

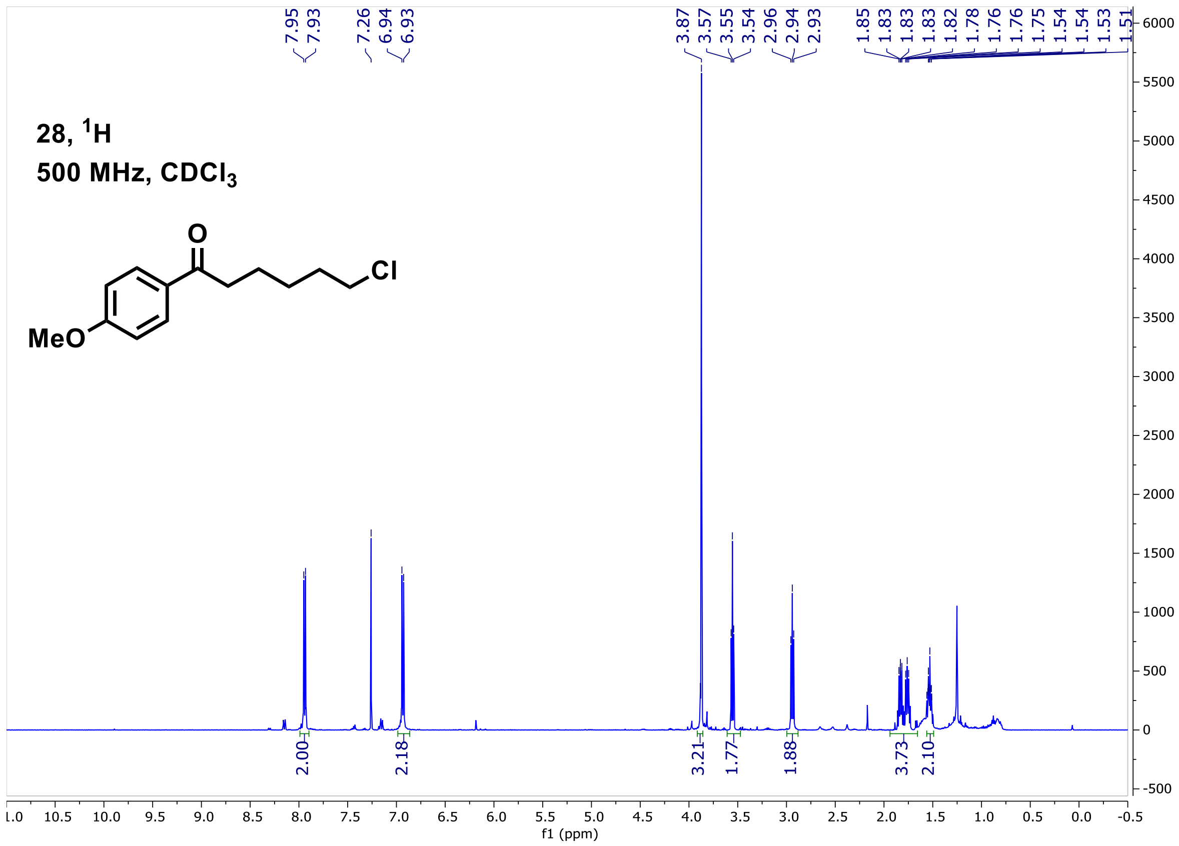
28, 1H
500 MHz, CDCl3
MeO
O
Cl
7.95
7.93
7.26
6.94
6.93
3.87
3.57
3.55
3.54
2.96
2.94
2.93
1.85
1.83
1.83
1.83
1.82
1.78
1.76
1.76
1.75
1.54
1.54
1.53
1.51
2.00
2.18
3.21
1.77
1.88
3.73
2.10
f1 (ppm)

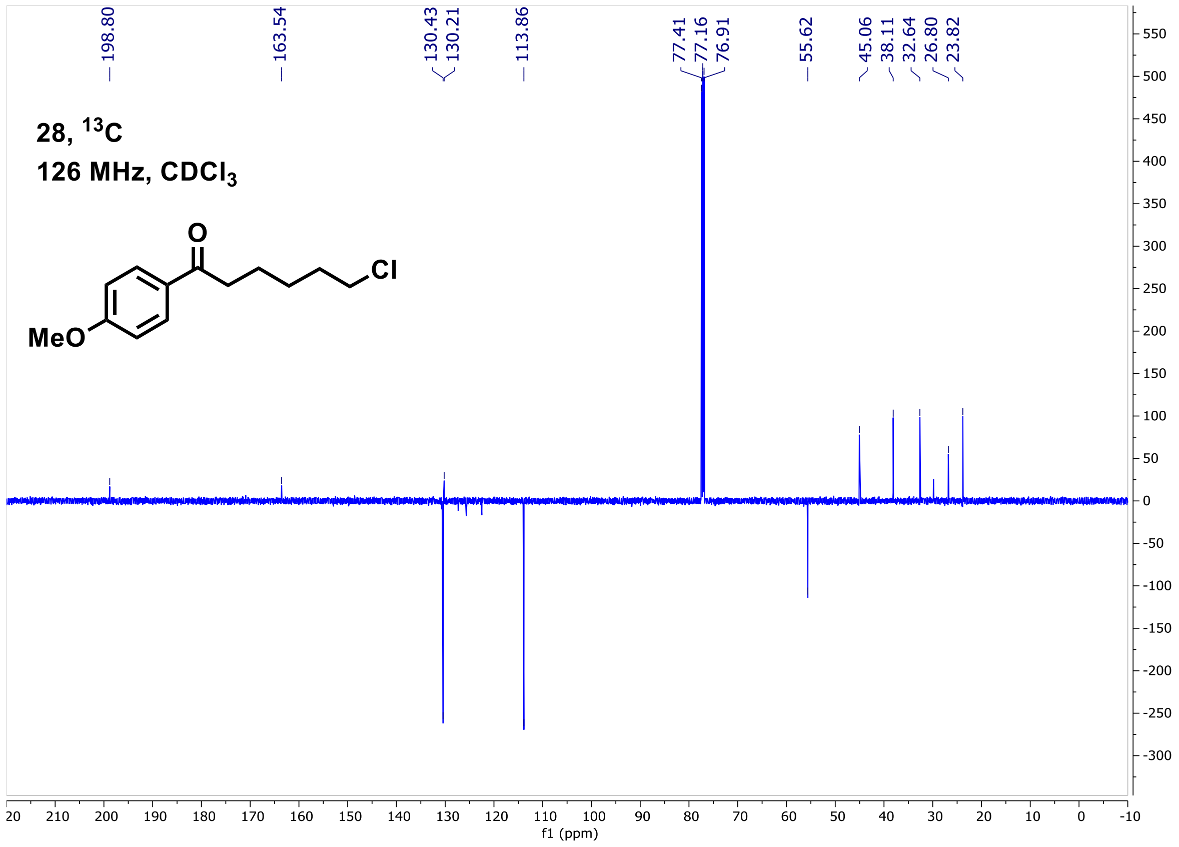
28, 13C
126 MHz, CDCl3
MeO
O
Cl
198.80
163.54
130.43
130.21
113.86
77.41
77.16
76.91
55.62
45.06
38.11
32.64
26.80
23.82
f1 (ppm)

6-iodo-1-(4-methoxyphenyl)hexan-1-one (29)

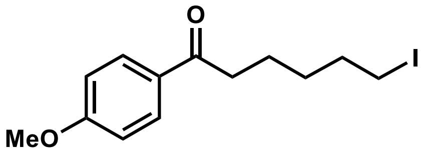

Prepared according to General Procedure (condition J) using **1** (62 mg, 0.3 mmol). ^{1}H NMR analysis of the crude reaction mixture showed 66% conversion. The crude residue was purified by flash column chromatography (2→5% EtOAc/Petrol, silica gel) to afford **29** (51 mg, 51%) as a yellow oil.

R_f = 0.17 (10% EtOAc/Petrol); **^{1}H NMR (500 MHz, $CDCl_3$)** δ = 7.99 – 7.89 (m, 2H), 6.99 – 6.88 (m, 2H), 3.87 (s, 3H), 3.21 (t, *J* = 7.0 Hz, 2H), 2.94 (t, *J* = 7.3 Hz, 2H), 1.95 – 1.81 (m, 2H), 1.81 – 1.71 (m, 2H), 1.52 – 1.45 (m, 2H); **^{13}C NMR (126 MHz, $CDCl_3$)** δ = 198.8, 163.6, 130.4, 130.2, 113.9, 55.6, 38.1, 33.5, 30.4, 23.5, 7.0.; **HRMS** (ES^+/Q-TOF) m/z : [M+H] Calcd for [$C_{13}H_{18}O_2{}^{127}I$] 333.0351; Found 333.0361.

These data are consistent with those previously reported in the literature.[18]

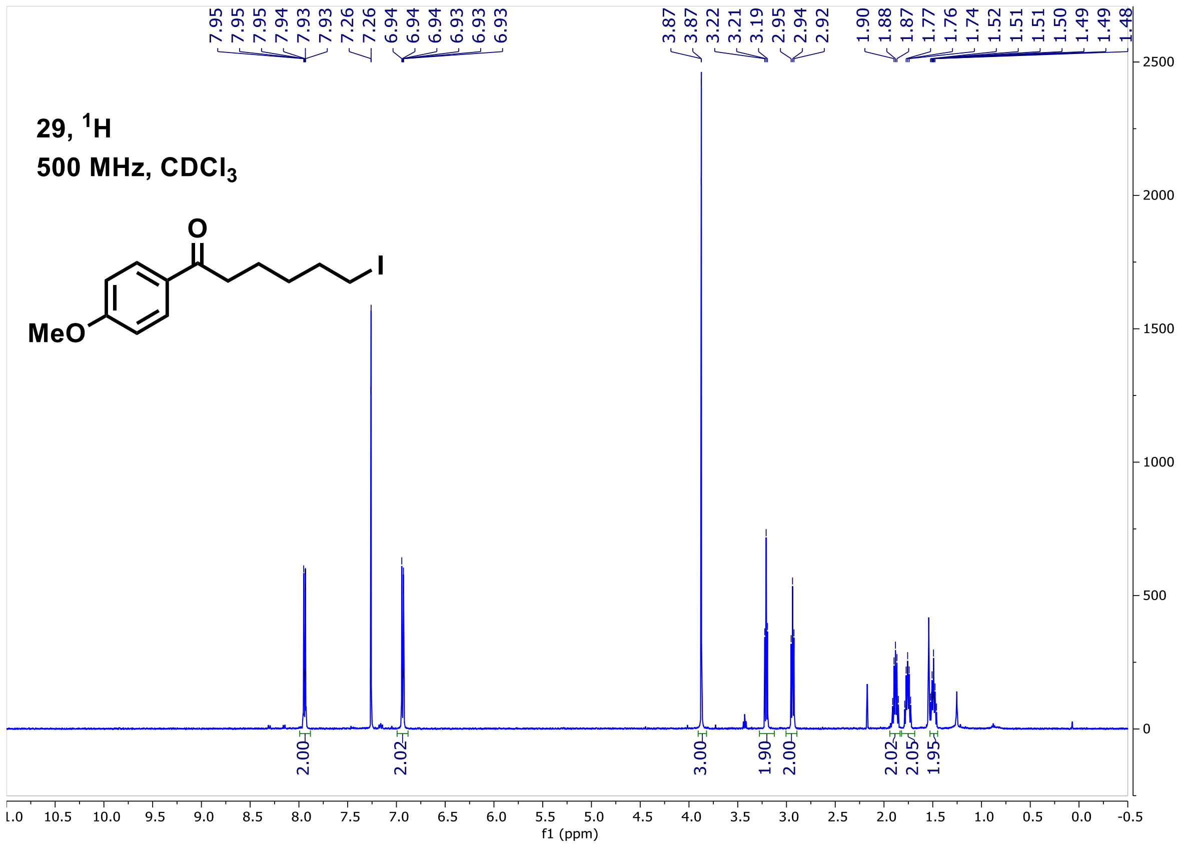

29, ^{1}H
500 MHz, $CDCl_3$
MeO
O
I
7.95 7.95 7.95 7.94 7.93 7.93 7.26 7.26 6.94 6.94 6.94 6.93 6.93 6.93
3.87 3.87 3.22 3.21 3.19 2.95 2.94 2.92
1.90 1.88 1.87 1.77 1.76 1.74 1.52 1.51 1.51 1.50 1.49 1.49 1.48
2.00 2.02 3.00 1.90 2.00 2.02 2.05 1.95
f1 (ppm)

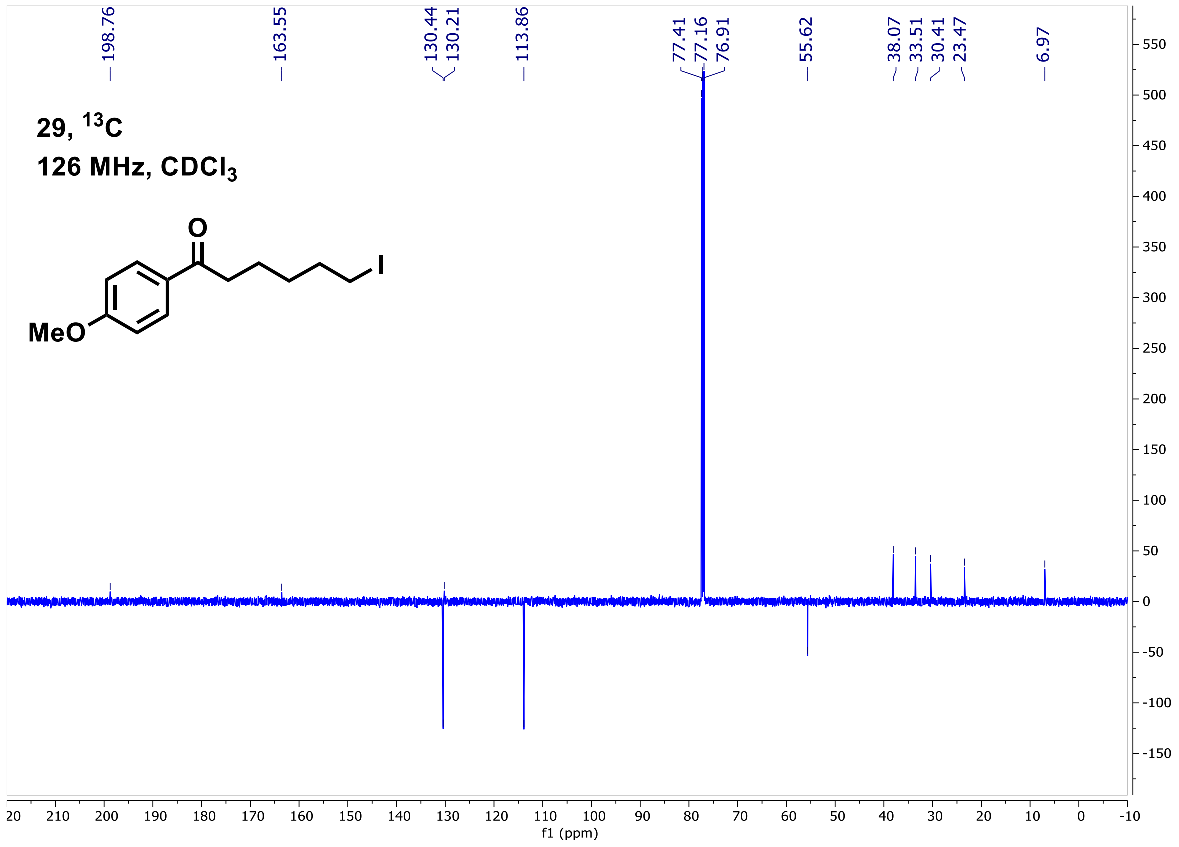

29, ^{13}C
126 MHz, $CDCl_3$
MeO
O
I
198.76
163.55
130.44 130.21
113.86
77.41 77.16 76.91
55.62
38.07 33.51 30.41 23.47
6.97
f1 (ppm)

6-(4-methoxyphenyl)-6-oxohexanal (30)

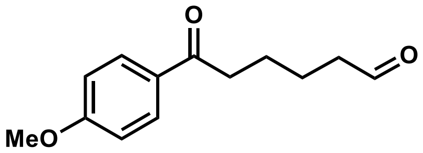

Prepared according to General Procedure (condition K) using **1** (62 mg, 0.3 mmol).The crude residue obtained was purified flash column chromatography (5→10% EtOAc/Petrol, silica gel) to **30** (46 mg, 70%) as a colourless viscous oil.

R_f = 0.12 (15% EtOAc/Petrol); **^{1}H NMR (400 MHz, $CDCl_3$)** δ = 9.78 (t, *J* = 1.6 Hz, 1H), 7.93 (d, *J* = 8.9 Hz, 2H), 6.93 (d, *J* = 8.9 Hz, 2H), 3.87 (s, 3H), 2.95 (t, *J* = 6.8 Hz, 2H), 2.53 – 2.46 (m, 2H), 1.82 – 1.67 (m, 4H) **^{13}C NMR (101 MHz, CDCl3)** δ = 202.5, 198.5, 163.6, 130.4, 130.1, 113.9, 55.6, 43.9, 37.9, 23.9, 21.9; **HRMS** (ES^+/Q-TOF) m/z : [M+H] Calcd for [$C_{13}H_{17}O_3$] 221.1178; Found 221.1176.

These data are consistent with those previously reported in the literature.[3]

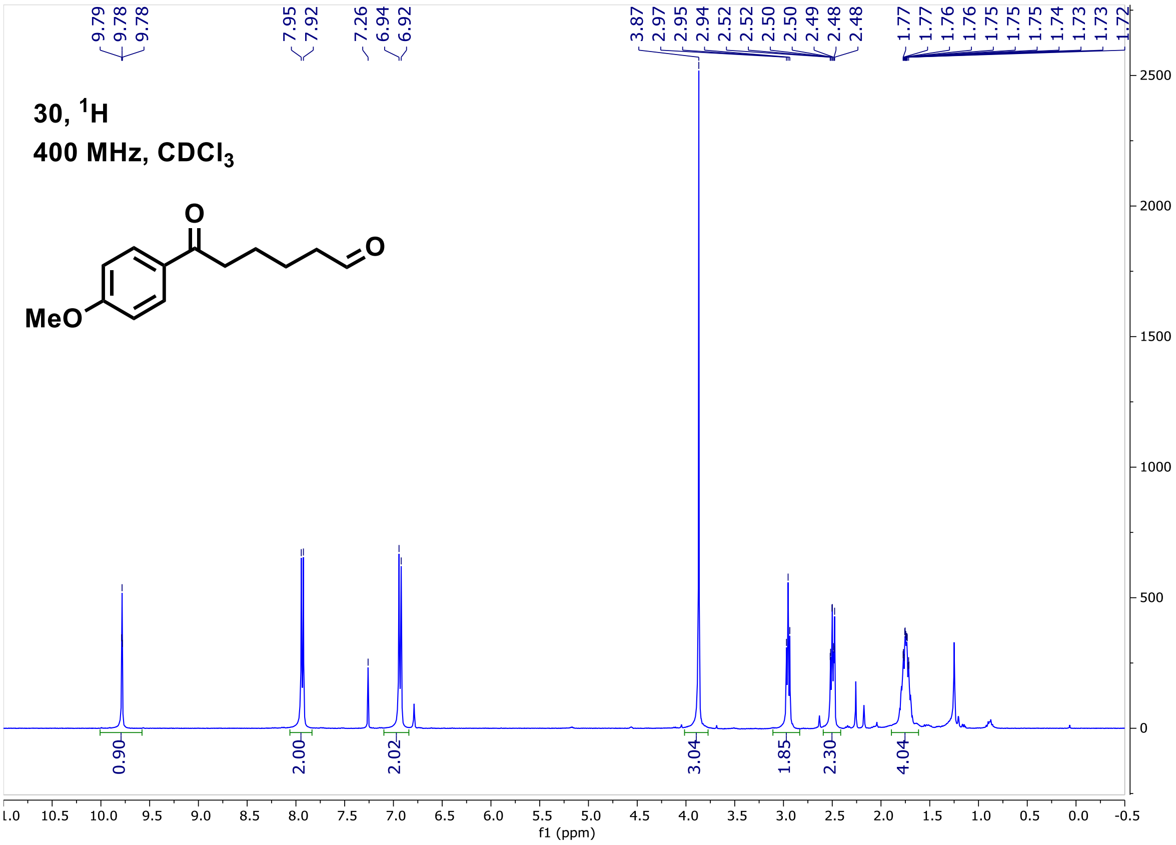

30, ^{1}H
400 MHz, $CDCl_3$
MeO
O
O

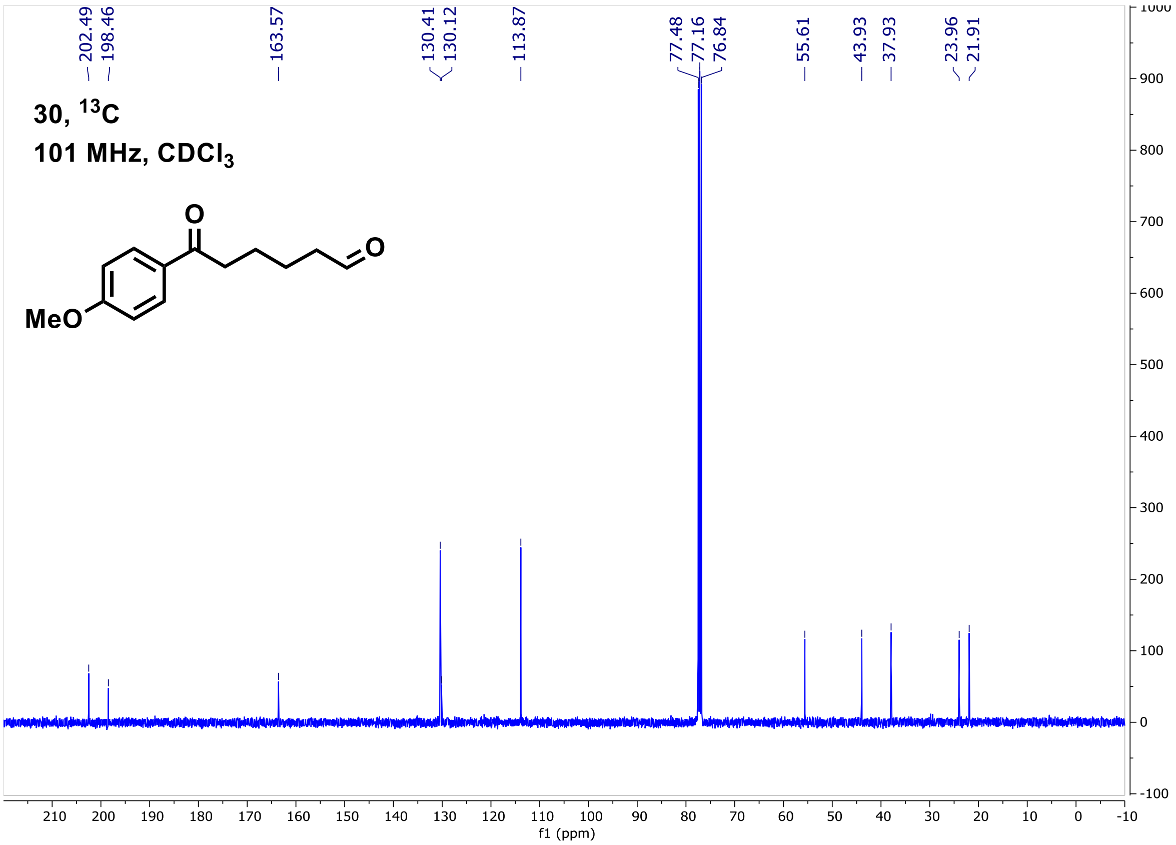

30, ^{13}C
101 MHz, $CDCl_3$
MeO
O
O

5. A. Product Derivatizations

Mechanochemical Nickel Catalyzed Cross-Electrophile Coupling

1-(4-methoxyphenyl)-6-phenylhexan-1-one (31)

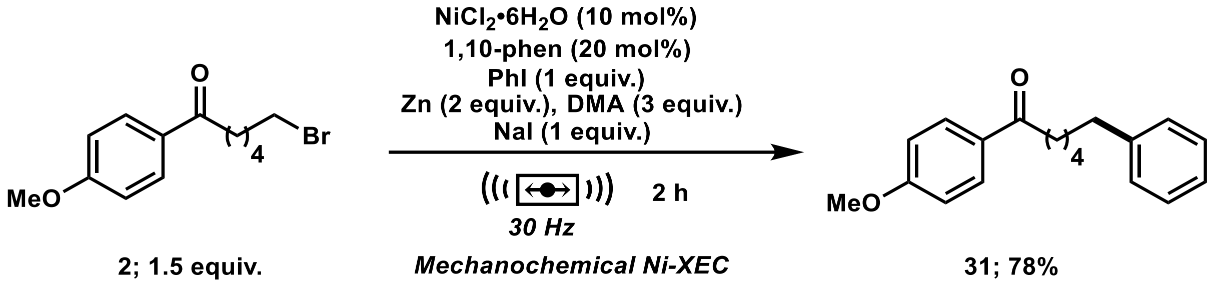

Prepared according to a literature procedure.[19] To a 15 mL stainless steel milling jar (FormTech Scientific) was added a 3 g stainless steel milling ball. Iodobenzene (0.102 g, 0.5 mmol), alkyl bromide **2** (0.224 g, 1.5 equiv.), $NiCl_2.6H_2O$ (0.012 g, 10 mol%), 1,10-phenanthroline (0.018 g, 20 mol%), zinc granular (20-30 mesh, unactivated, 0.065 g, 2 equiv.), NaI (0.075 g, 1 equiv.) and *N,N*-dimethylacetamide (0.140 mL, 3 equiv.) were all added under an air atmosphere. The milling jar was closed and placed on the mixer mill (Retsch MM400). The reaction was milled for 2 hours at 30 Hz. After the reaction period, the mixture was washed from the jar into a conical flask with CH_2Cl_2 (~15 mL). 1 M HCl was added to the flask and the mixture was stirred for 5 minutes to hydrolyse metal salts. The resulting mixture was transferred to a separating funnel and the layers separated. The aqueous layer was extracted with CH_2Cl_2 (2 x 10 mL). The combined organic layers were washed with brine (~30 mL), dried over anhydrous $MgSO_4$, filtered and concentrated under reduced pressure to give the crude product as an orange oil. The crude product was purified by flash column chromatography (0→10% EtOAc / petrol) to give **31** (0.110 g, 78%) as a white solid.

R_f = 0.39 (10% EtOAc / petrol); **M.p.** = 41-43 °C; **^{1}H NMR (500 MHz, $CDCl_3$)** δ 7.96 – 7.92 (m, 2H), 7.30 – 7.25 (m, 2H), 7.20 – 7.16 (m, 3H), 6.95 – 6.91 (m, 2H), 3.87 (s, 3H), 2.93 – 2.90 (m, 2H), 2.66 – 2.60 (m, 2H), 1.81 – 1.73 (m, 2H), 1.72 – 1.64 (m, 2H), 1.47 – 1.39 (m, 2H); **^{13}C NMR (126 MHz, $CDCl_3$)** δ = 199.1, 163.4, 142.7, 130.4, 130.3, 128.5, 128.4, 125.8, 113.8, 55.6, 38.3, 35.9, 31.5, 29.2, 24.5.

The spectroscopic data is in accordance with literature.[4]

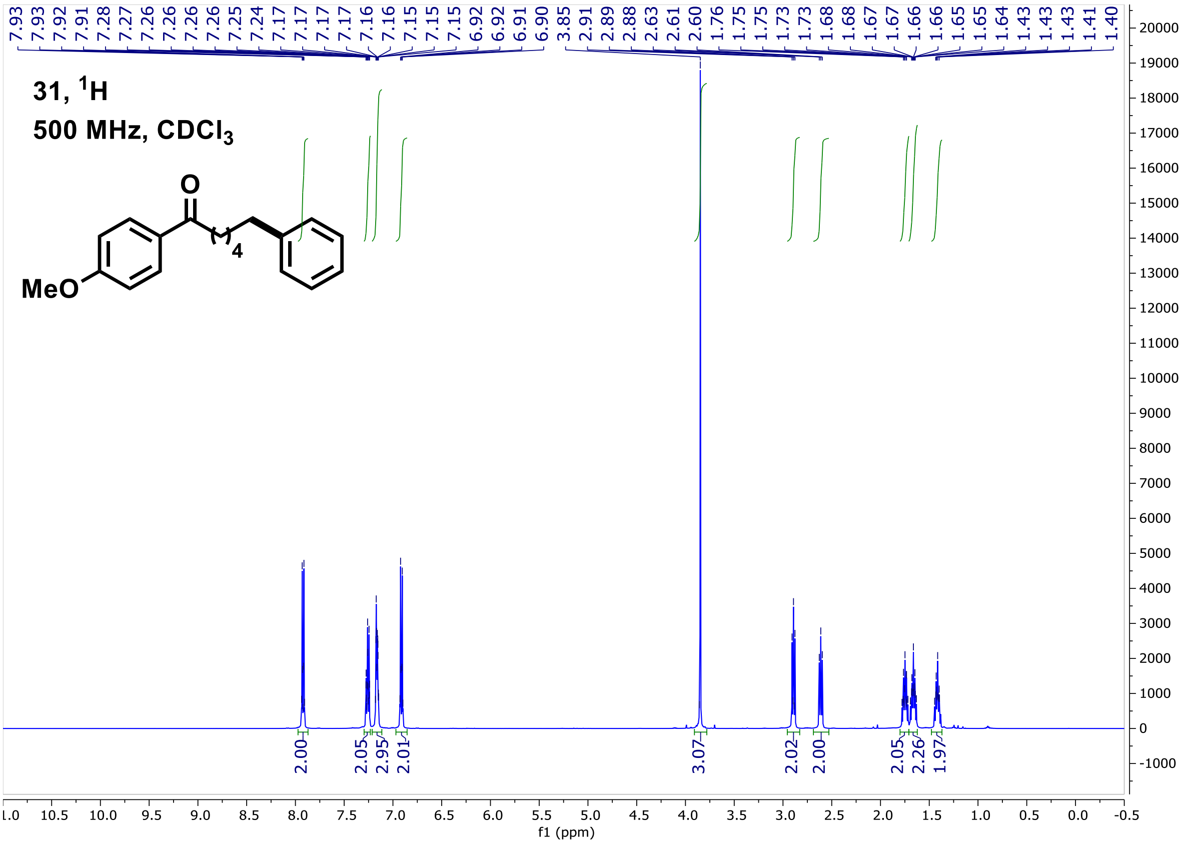
31, 1H
500 MHz, CDCl3
MeO
O
4
7.93
7.93
7.92
7.91
7.28
7.27
7.26
7.26
7.26
7.26
7.25
7.24
7.17
7.17
7.17
7.17
7.16
7.16
7.15
7.15
7.15
6.92
6.92
6.91
6.90
3.85
2.91
2.89
2.88
2.63
2.61
2.60
1.76
1.75
1.75
1.73
1.73
1.68
1.68
1.67
1.67
1.66
1.66
1.65
1.65
1.64
1.43
1.43
1.43
1.41
1.40
2.00
2.05
2.95
2.01
3.07
2.02
2.00
2.05
2.26
1.97
f1 (ppm)

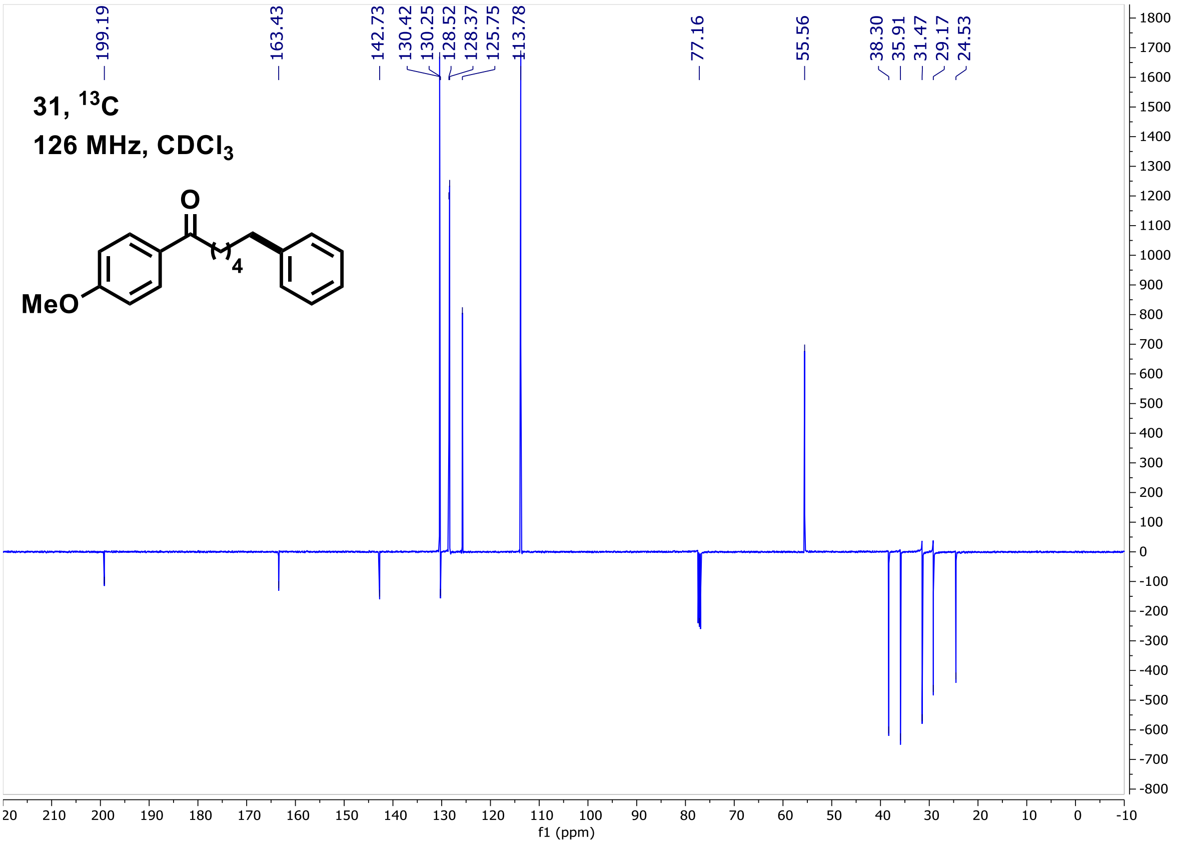
31, 13C
126 MHz, CDCl3
MeO
O
4
199.19
163.43
142.73
130.42
130.25
128.52
128.37
125.75
113.78
77.16
55.56
38.30
35.91
31.47
29.17
24.53
f1 (ppm)

Baeyer-Villiger oxidation

4-methoxyphenyl 6-bromohexanoate (32)

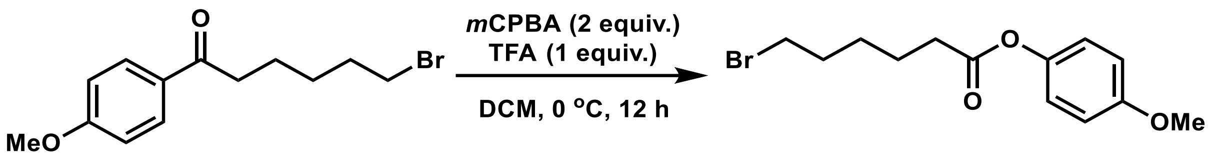

A flame dried 25 mL RBF was charged with 6-bromo-1-(4-methoxyphenyl)hexan-1-one **2** (0.5 mmol, 1.0 equiv, 145 mg) and 3-chlorobenzoperoxoic acid (~ 70% purity, 172 mg; 2.0 mmol, 2.0 equiv.). The solids were dissolved in 2 mL of dry DCM to form a suspension and stirred vigorously while cooled with an ice bath. Trifluoroacetic acid (1.0 mmol, 1.0 equiv, 37 μL) was added slowly via syringe. The vial was left in the ice bath overnight, during which time the reaction warmed up to room temperature. The crude material was washed in saturated $NaHCO_3$, extracted with DCM, concentrate to small volume, and purified by silica gel column chromatography to afford **32** as a colourless liquid (128 mg, 84%).

R_f = 0.39 (15% EtOAc/Petrol); **FTIR** (ν_{max} cm^{-1}, thin film) 2933, 2839, 1752, 1606, 1593, 1458, 1442, 1190, 1116, 1029, 833, 819, 756; **^{1}H NMR (500 MHz, $CDCl_3$)** δ = 7.04 – 6.94 (m, 2H), 6.91 – 6.83 (m, 2H), 3.80 (s, 3H), 3.44 (t, *J* = 6.7 Hz, 2H), 2.56 (t, *J* = 7.4 Hz, 2H), 2.03 – 1.86 (m, 2H), 1.81 – 1.71 (m, 2H), 1.57 (m, 2H); **^{13}C NMR (126 MHz, $CDCl_3$)** δ = 172.4, 157.3, 144.3, 122.4, 114.6, 55.7, 34.2, 33.6, 32.5, 27.8, 24.2; **HRMS** (EI$^+$/FTMS) m/z : [M] Calcd for [$C_{13}H_{17}O_3Br$] 300.0361, found 300.0355.

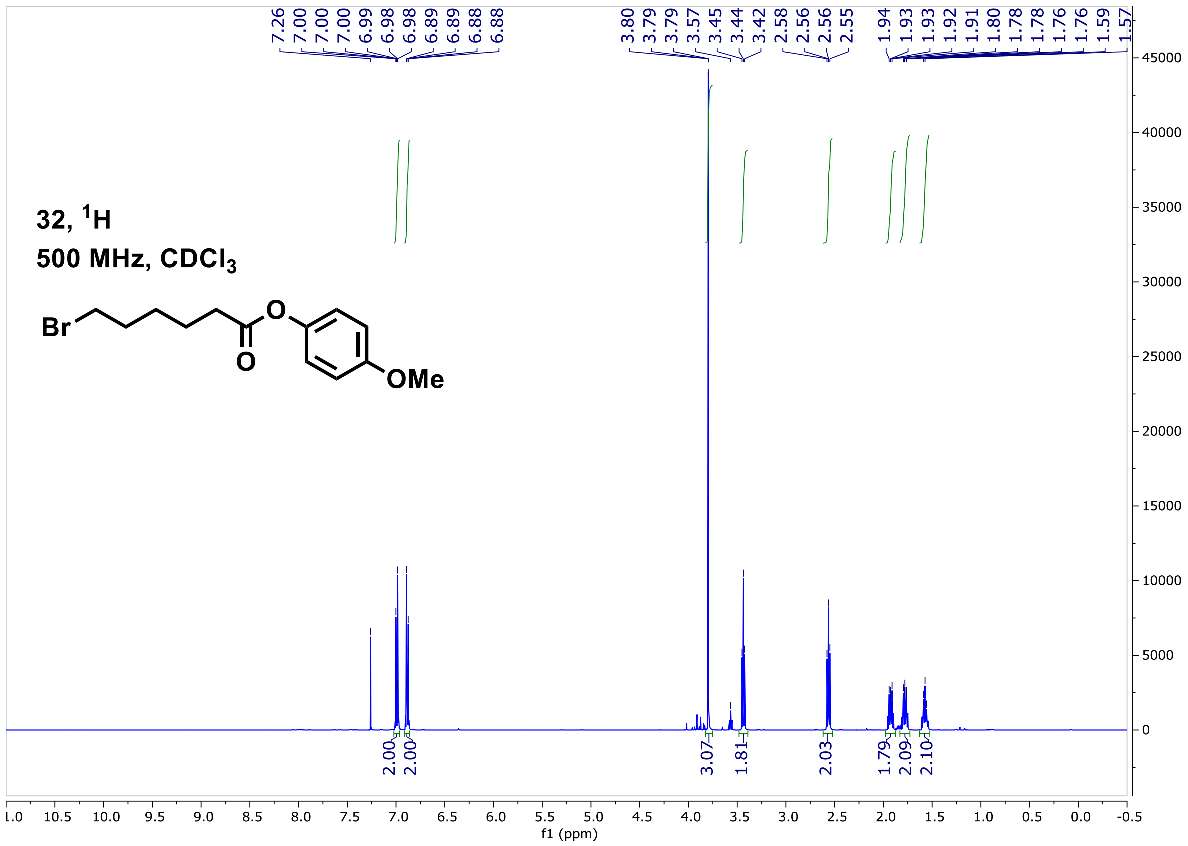
7.26
7.00
7.00
7.00
6.99
6.98
6.98
6.89
6.89
6.88
6.88
3.80
3.79
3.79
3.57
3.45
3.44
3.42
2.58
2.56
2.56
2.55
1.94
1.93
1.93
1.92
1.91
1.80
1.78
1.78
1.76
1.76
1.59
1.57
32, 1H
500 MHz, CDCl3
Br
O
O
OMe
2.00
2.00
3.07
1.81
2.03
1.79
2.09
2.10
f1 (ppm)

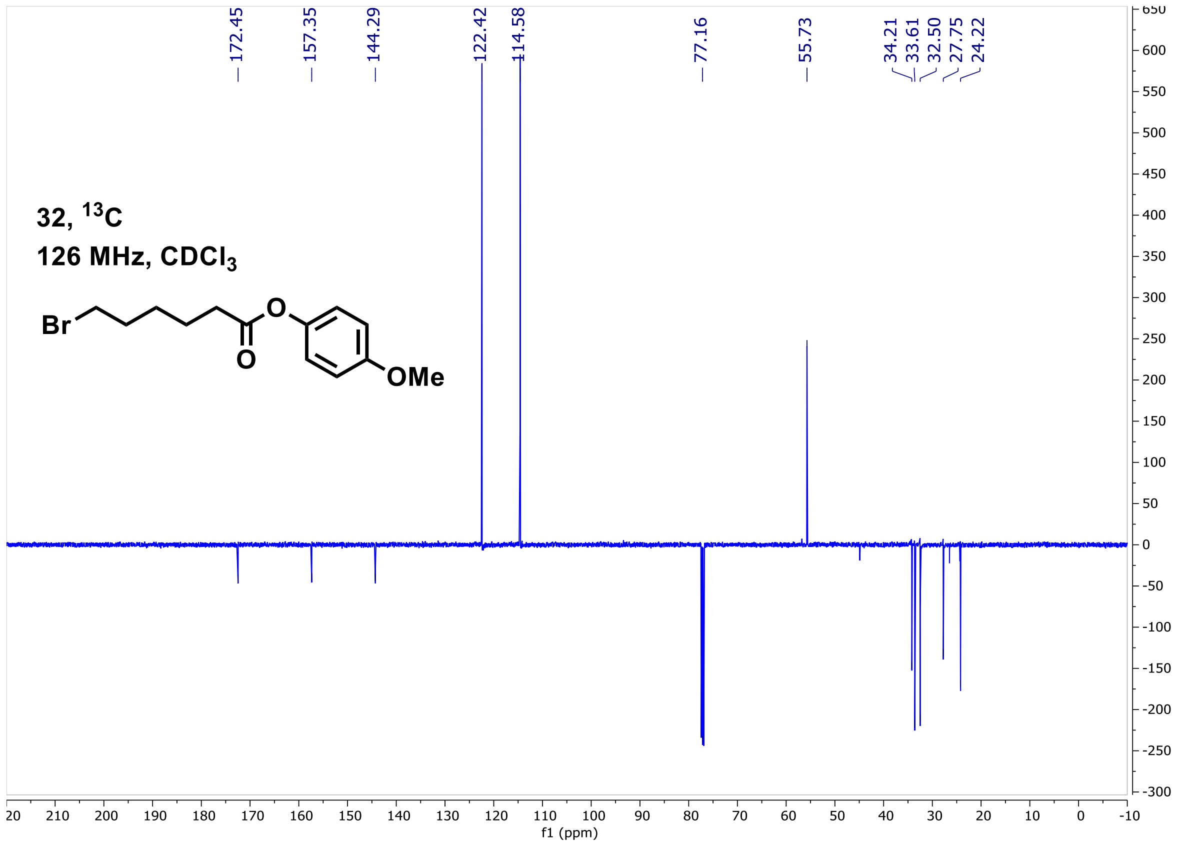
172.45
157.35
144.29
122.42
114.58
77.16
55.73
34.21
33.61
32.50
27.75
24.22
32, 13C
126 MHz, CDCl3
Br
O
O
OMe
f1 (ppm)

5. B. Experimental for Flow Electrochemical Scale Up

General Methods for Flow Processes

The flow setup used PFA tubing with a 0.79±0.1 mm internal diameter and 1.58±0.1 mm outer diameter supplied by Polyflon. All flow fittings and connections were purchased from Kinesis (Gripper fitting nuts, part number: 002103; Adapters, part number: P-618; Omnilok type-p fitting ferrule, part number: 008FT16; Y-Connector, part number: P-512; Pumps used was Knauer P4.1S and P2.1S Azura HPLC pumps. Pumps were calibrated by pumping solvent into a measuring cylinder, recording the time taken for the desired volume to be dispensed and then adjusting the pump's flow rate to the correct value, if required. The power supply used was a Voltcraft LRP-1205 that supplied DC to the electrochemical system. The electrochemical flow cell was purchased from Cambridge Reactor Design, the Ammonite 8 (part number: 74660). The electrochemical flow cell consists of carbon/platinum electrodes that are fixed either side of a FFKM gasket with a channel groove length of 1000 mm and an internal volume of 2.5 mL, of which 1 mL is exposed to the electrodes. The inlet and outlet fittings of the ammonite 8 cell were modified from 1/16" ID to accommodate 1/32" ID PFA tubing using Swagelok reducers, nuts and ferrules.

The electrochemical cell was dismantled after 3 runs, and electrodes were washed with IPA and MeCN. Graphite electrode was polished using silica 230-400 particle size with cotton. Platinum electrode was washed with IPA and MeCN and prior to use was burned on Bunsen burner to remove any organic/ inorganic impurities.

To investigate the initial parameters, recirculation flow was performed which was later translated into continuous flow scaleup.

Recirculating Flow

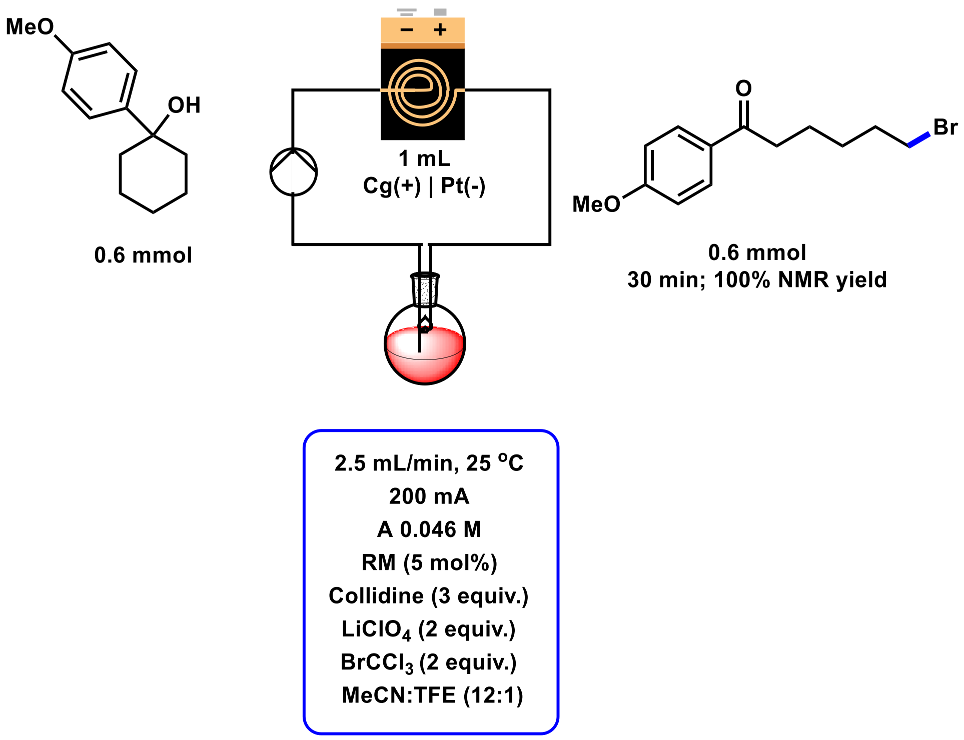

Fixed Parameters

A 25 mL round-bottom flask and stirrer bar was flame dried and charged with **1** (0.6 mmol, 0.125 g), RM-2 (0.06 mmol, 10 mg), collidine (1.8 mmol, 240 µL) and sealed with a Suba-seal and parafilm. The reagent flask was evacuated on a Schlenk line and back filled with nitrogen gas for three cycles. Both the inlet and outlet tubing of the electrochemical flow system were pushed through the Suba-seal and the reagents dissolved with dry degassed MeCN (12 mL) and trifluoroethanol (1 mL). The reaction mixture was then purged with N_2 for 10 min. $BrCCl_3$ (120 µL, 0.12 mmol) was then added under N_2. The HPLC pump was primed with the reaction mixture and set to recirculate at 2.5 mL/min. The electrolysis was then commenced and conducted at a constant current of 400 mA, the recirculated reaction was monitored by TLC until completion, which, at this scale required 30 min.

Upon completion, electrolysis was turned off and 0.2 mmol of trimethoxybenzene was added as an internal standard. The crude reaction mixture was then analyzed by ^{1}H NMR spectroscopy, which showed quantitative yield.

Single Pass Flow

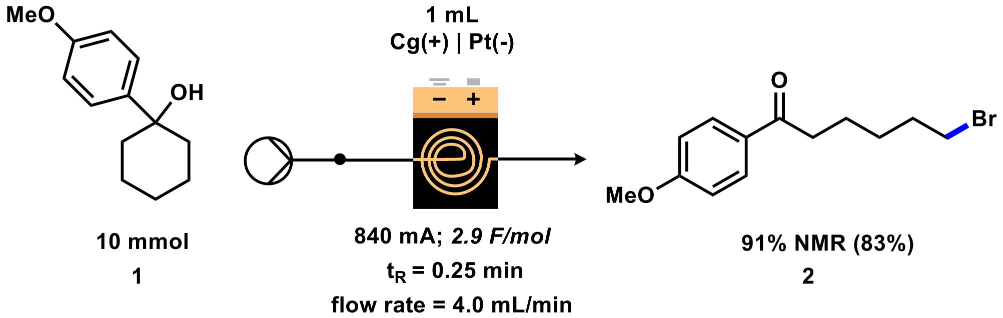

A 500 mL round-bottom flask and stirrer bar was flame dried and charged with **1** (10.0 mmol, 2.06 g), RM-2 (0.5 mmol, 168 mg), collidine (4.0 mL, 30.0 mmol) and sealed with a Suba-seal and parafilm. The reagent flask was evacuated on a Schlenk line and back filled with nitrogen gas for three cycles. Both the inlet and outlet tubing of the electrochemical flow system were pushed through the Suba-seal and the reagents dissolved with dry degassed MeCN (200 mL) and trifluoroethanol (16.6 mL). The reaction mixture was then purged with N_2 for 10 min. $BrCCl_3$ (1.9 mL, 20.0 mmol) was then added under N_2.

The HPLC pump was primed with the reaction mixture and flow was set to 4.0 mL/min at constant current of 840 mA. HPLC pumping was initiated, and the outlet of the flow system was set to waste for the first 3.46 mL (representing 1 whole flow path volume, inclusive of tubing, connectors and reactor) of reaction mixture to allow the flow system to be filled. After this initial priming, the power supply was switched on and the electrolysis commenced at a constant current with the system outlet still set to waste for a further 2.5 mL (representing a total volume from 'in' to 'out' of the electrochemical reactor; exposed electrochemical path = 1 mL). The outlet stream of the flow system was then collected for 205 mL.

After the end of electrolysis, to the collected volume was added sat. NH_4Cl (50 mL) and extracted with EtOAc (100 mL). The organic layer was then washed with aq. $CuSO_4$ (25 mL x 2). The organic layer was then dried over $MgSO_4$ and concentrated under reduced pressure. The crude residue this obtained was purified by flash column chromatography (5→10 % EtOAc/Petrol, silica gel) to afford **2** (2.3 g, 83%) as off white solid.

Graphical illustrations of the flow set-up

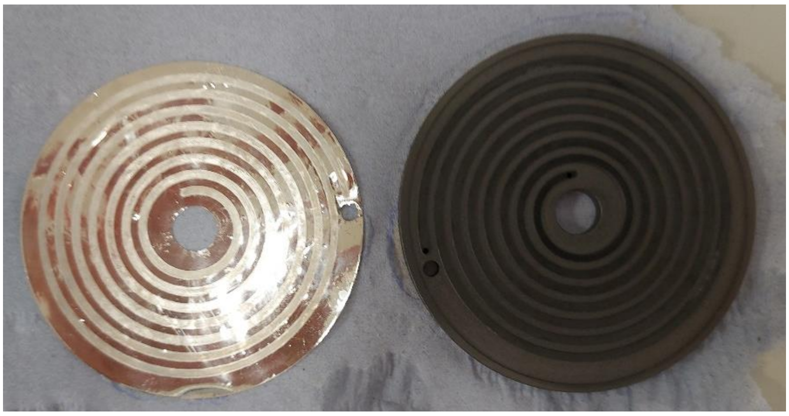

Figure S17 – platinum electrode (left) and graphite electrode with microfluid channel (right)

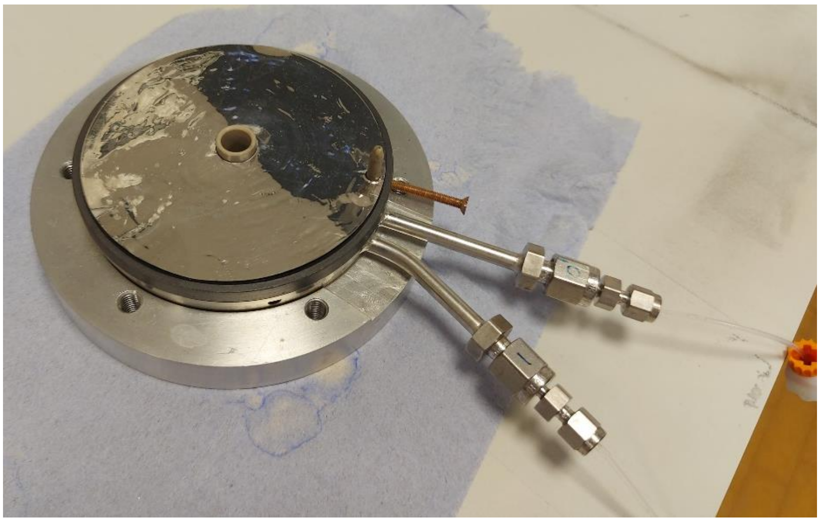

Figure S18 – graphite and platinum electrodes loaded onto the Ammonite8 flow reactor body

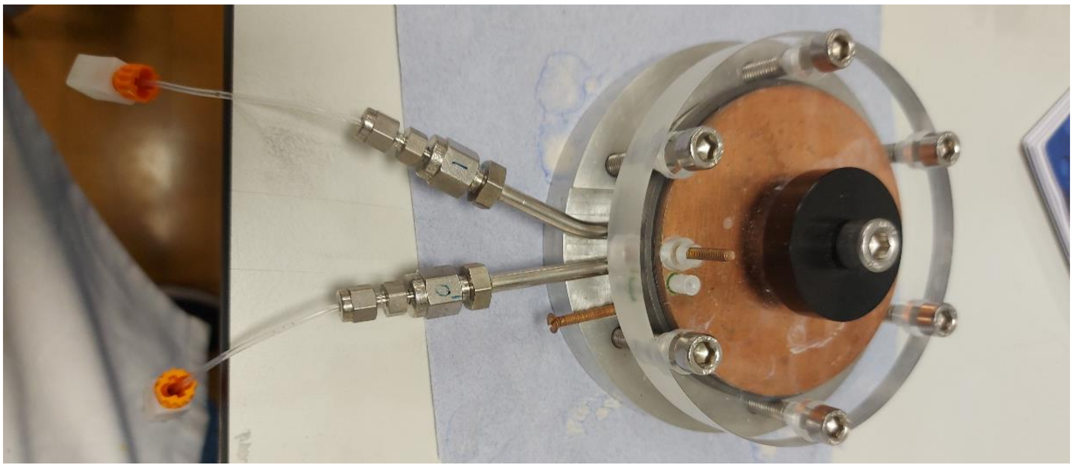

Figure S19 – assembled Ammonite8 electrochemical flow reactor

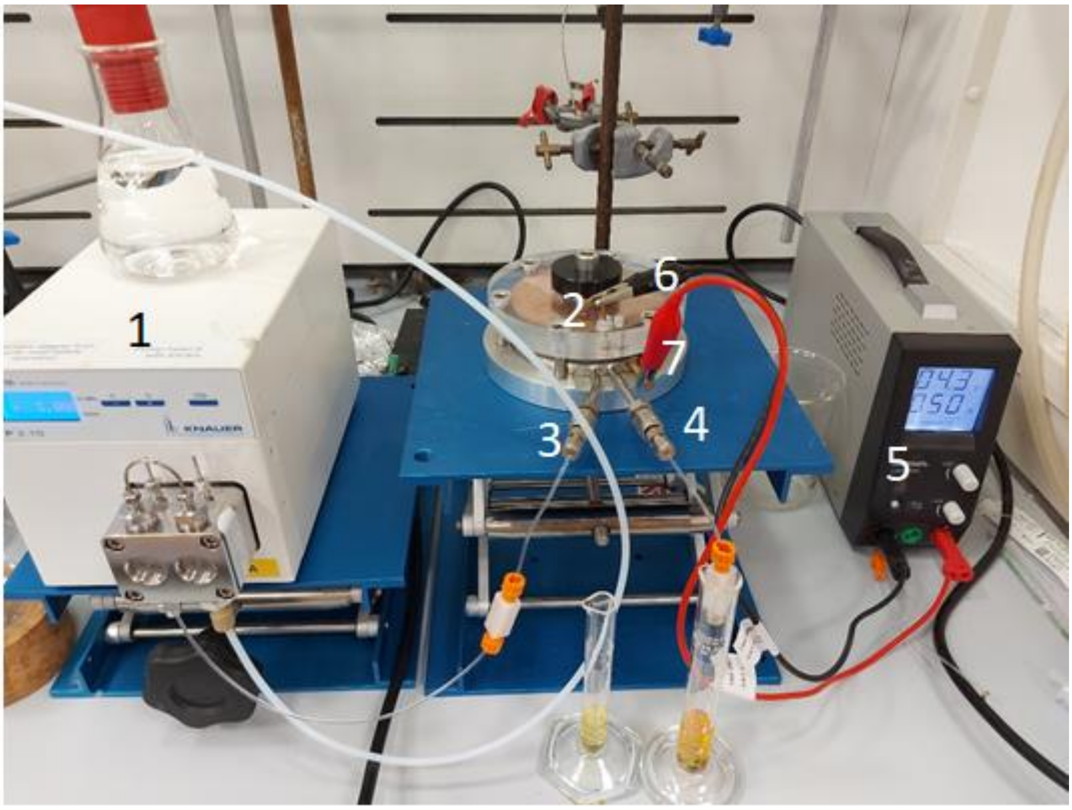

Figure S20 – A fully assembled flow electrochemical reactor

1- HPLC pump, 2- Ammonite8 electrochemical flow reactor, 3- reactor inlet, 4- reactor outlet, 5- potentiostat, 6- cathode and 7- anode

6. Mechanistic Studies

Synthesis of 1-methoxy-4-(1-methoxycyclohexyl)benzene (33)

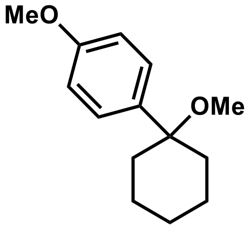

To a solution of **S1** (618.9 mg, 3.00 mmol) in THF (15 mL) was slowly added NaH (60% dispersed in mineral oil, 156 mg, 3.90 mmol, 1.30 eq.). Once gas evolution had ceased, the solution was cooled to 0 °C and methyl iodide (1.87 mL, 30.0 mmol, 10.0 eq.) was added slowly. The reaction mixture was stirred at rt for 64 h, before being cooled to 0 °C and H_2O (15 mL) was added. The layers were separated, and the aqueous layer was further extracted with Et_2O (2 × 20 mL). The combined organics were then washed with H_2O and brine, dried ($MgSO_4$), and concentrated under reduced pressure. The crude residue was purified by flash column chromatography (5% EtOAc/petrol, silica gel) to afford **33** (80 mg, 12%) as a colourless oil.

R_f = 0.48 (10% EtOAc/Petrol); **^{1}H NMR (300 MHz, $CDCl_3$)** δ = 7.37 – 7.29 (m, 2H), 6.92 – 6.84 (m, 2H), 3.81 (s, 3H), 2.94 (s, 3H), 2.06 – 1.93 (m, 2H), 1.80 – 1.46 (m, 7H), 1.37 – 1.17 (m, 1H). **^{13}C NMR (75 MHz, $CDCl_3$)** δ = 158.5, 138.1, 127.4, 113.6, 77.1, 55.3, 49.5, 35.6, 25.8, 22.2.

These data are consistent with those previously reported in the literature.[20]

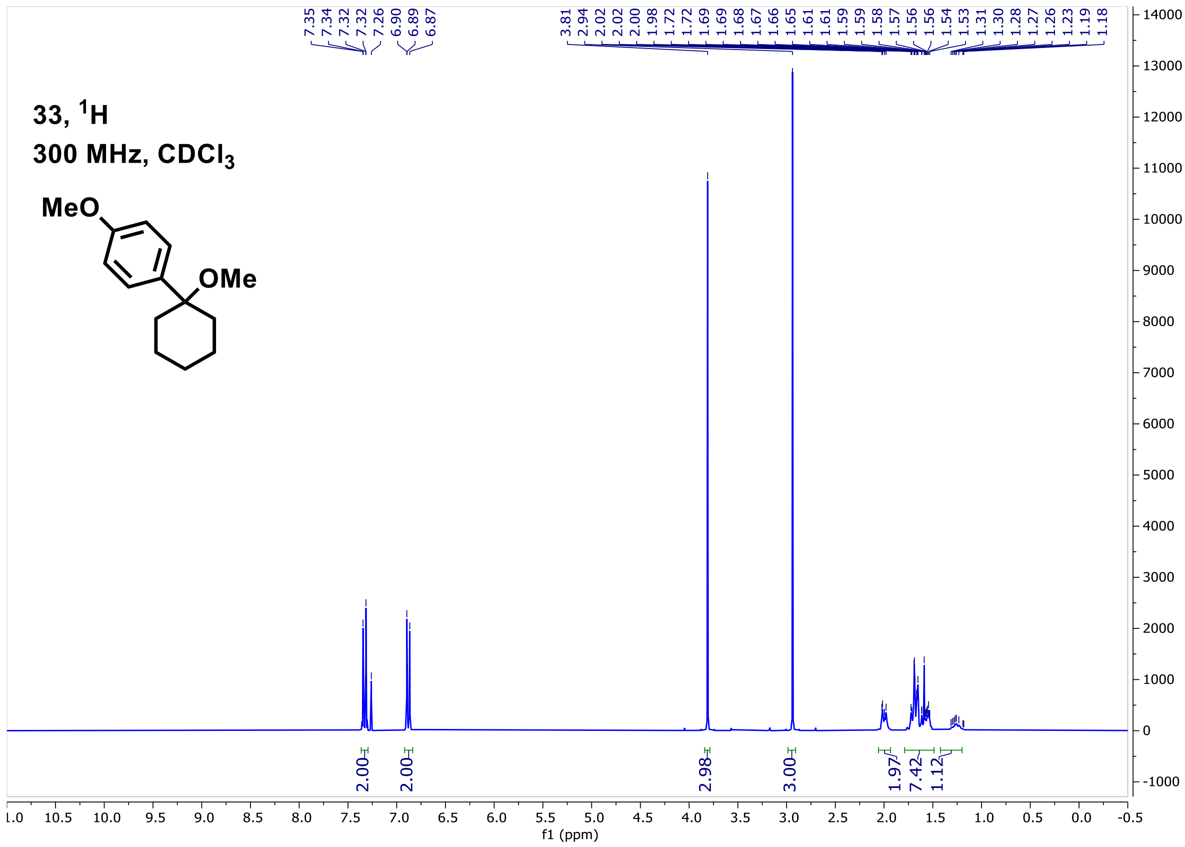
33, ¹H
300 MHz, CDCl₃
MeO
OMe
f1 (ppm)

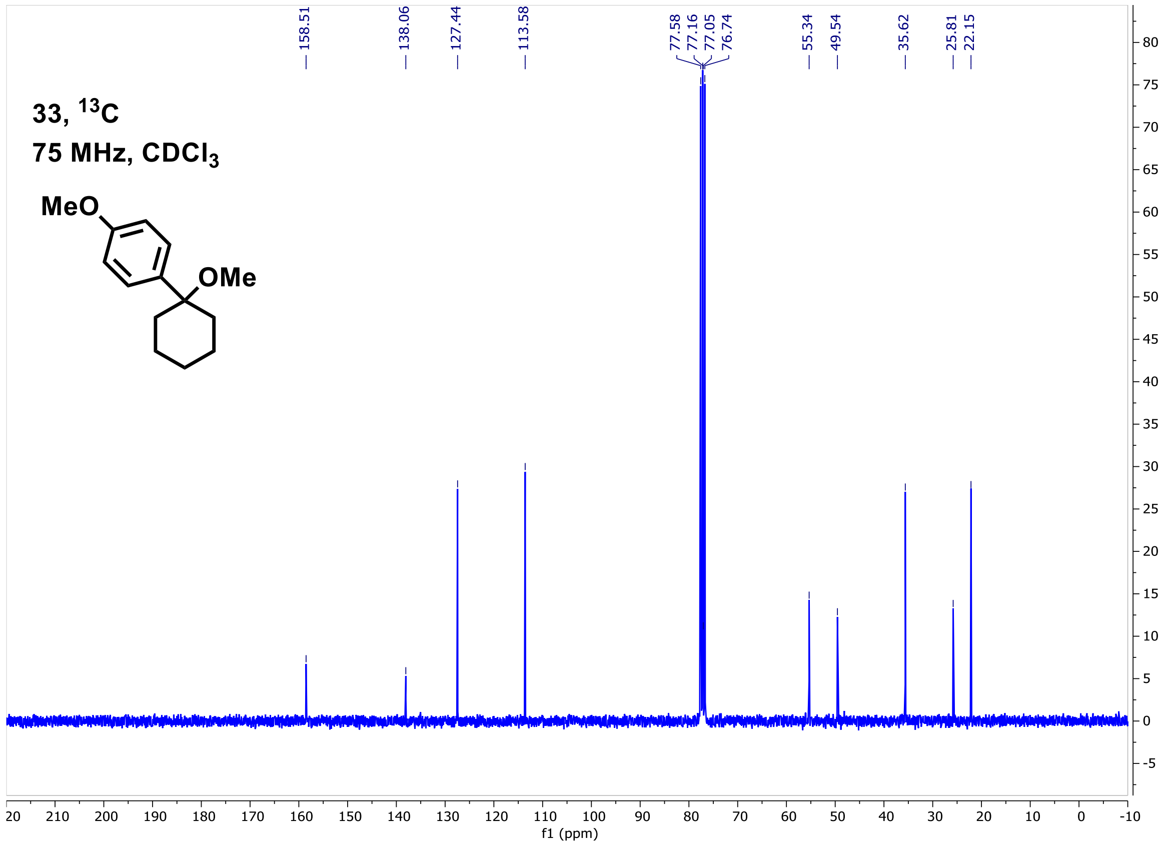
33, ¹³C
75 MHz, CDCl₃
MeO
OMe
f1 (ppm)

Reaction of **(33)** under electrochemical conditions:

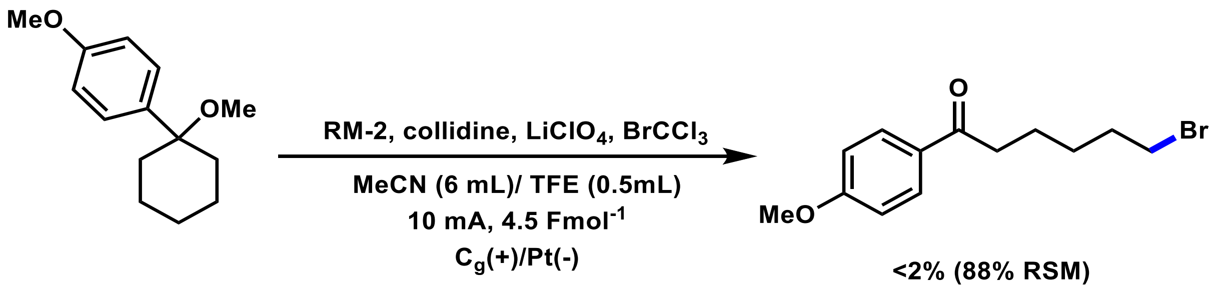

Experimental Procedure:

The reaction was performed according to General Procedure (standard conditions) using **33** (66.1 mg, 0.30 mmol). To the reaction mixture was then added 0.1 mmol of trimethoxybenzene as an internal standard to attain the crude NMR yield.

Cyclic voltammogram of **RM-2**

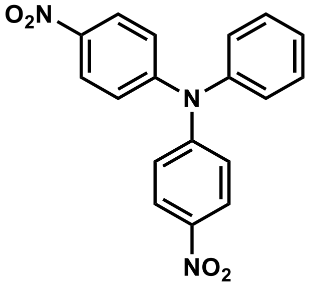

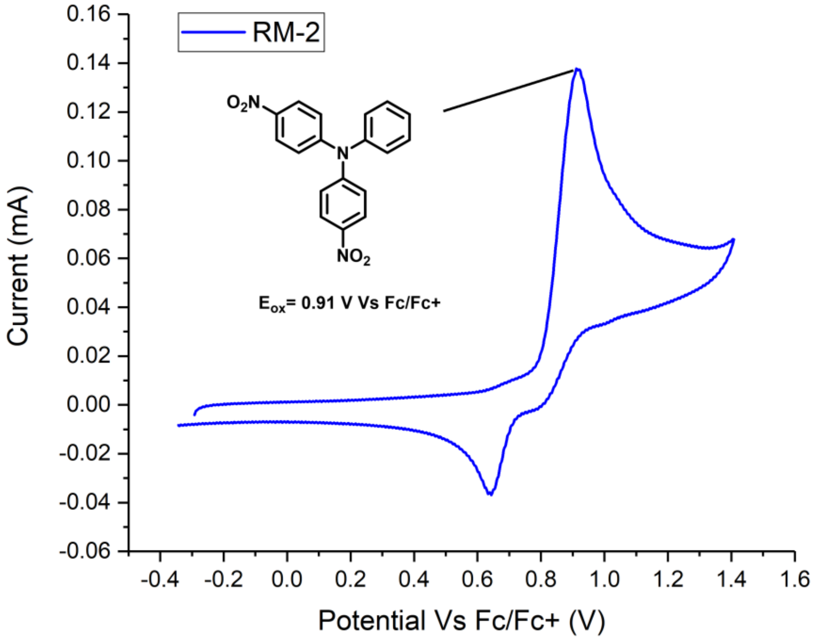

Figure S21 – Cyclic voltammogram of RM-2 (4.0 mM) MeCN (4 mM), $LiClO_4$ (0.1 M).
***Scan rate**: 100 mV/s*

Cyclic voltammogram of **1**

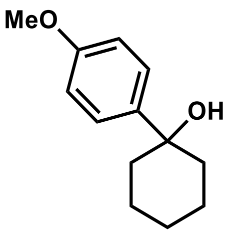

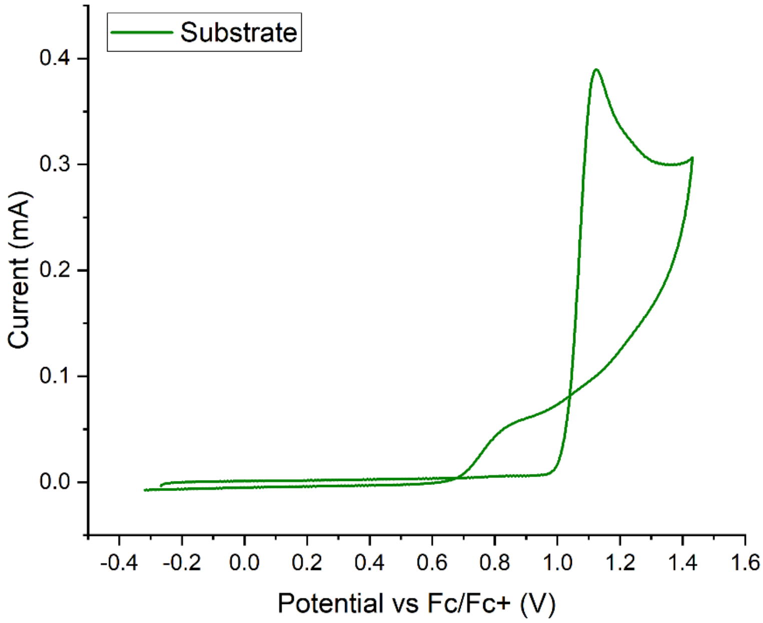

Figure S22 – Cyclic voltammogram of parent substrate (4.0 mM) MeCN (4 mM), $LiClO_4$ (0.1 M).
***Scan rate**: 100 mV/s*

Cyclic voltammogram of **RM-2 + 1 + Collidine**

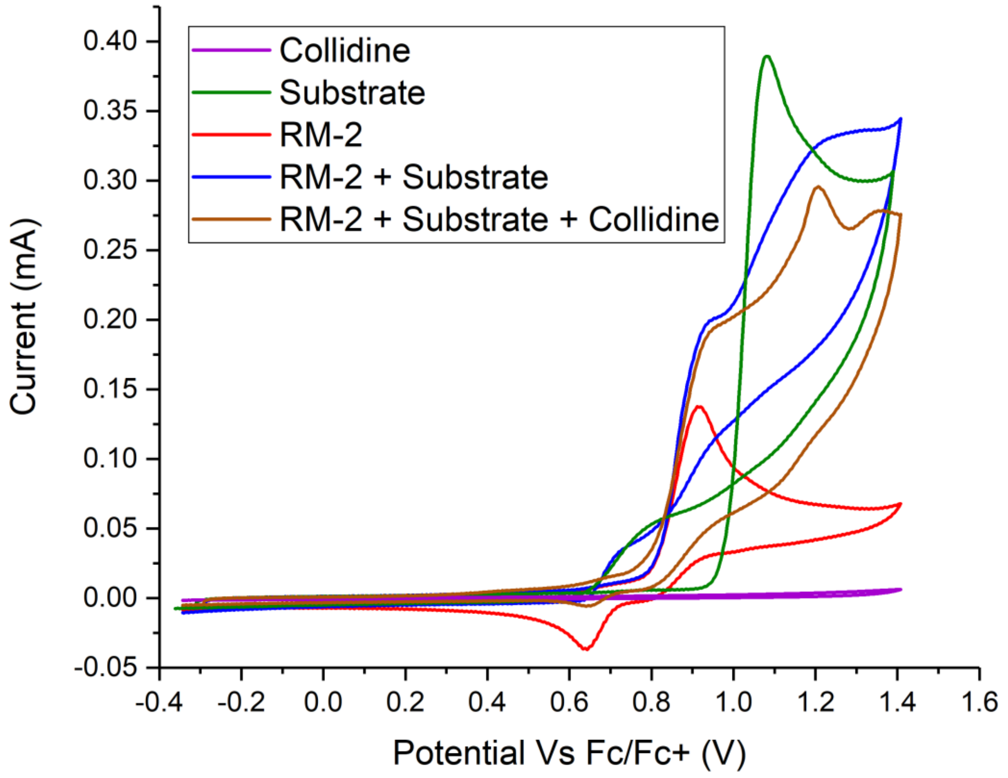

*Figure S23 – Cyclic voltammogram of (purple line) collidine (4.0 mM), (green line) parent substrate (4.0 mM), (red line) RM-2 (4.0 mM), (blue line)RM-2 + parent substrate, (brown line) RM-2 + parent substrate + collidine in MeCN/TFE (12:1), $LiClO_4$ (0.1 M). **Scan rate**: 100 mV/s*

Evidence for the H-bonding interaction between substrate and collidine

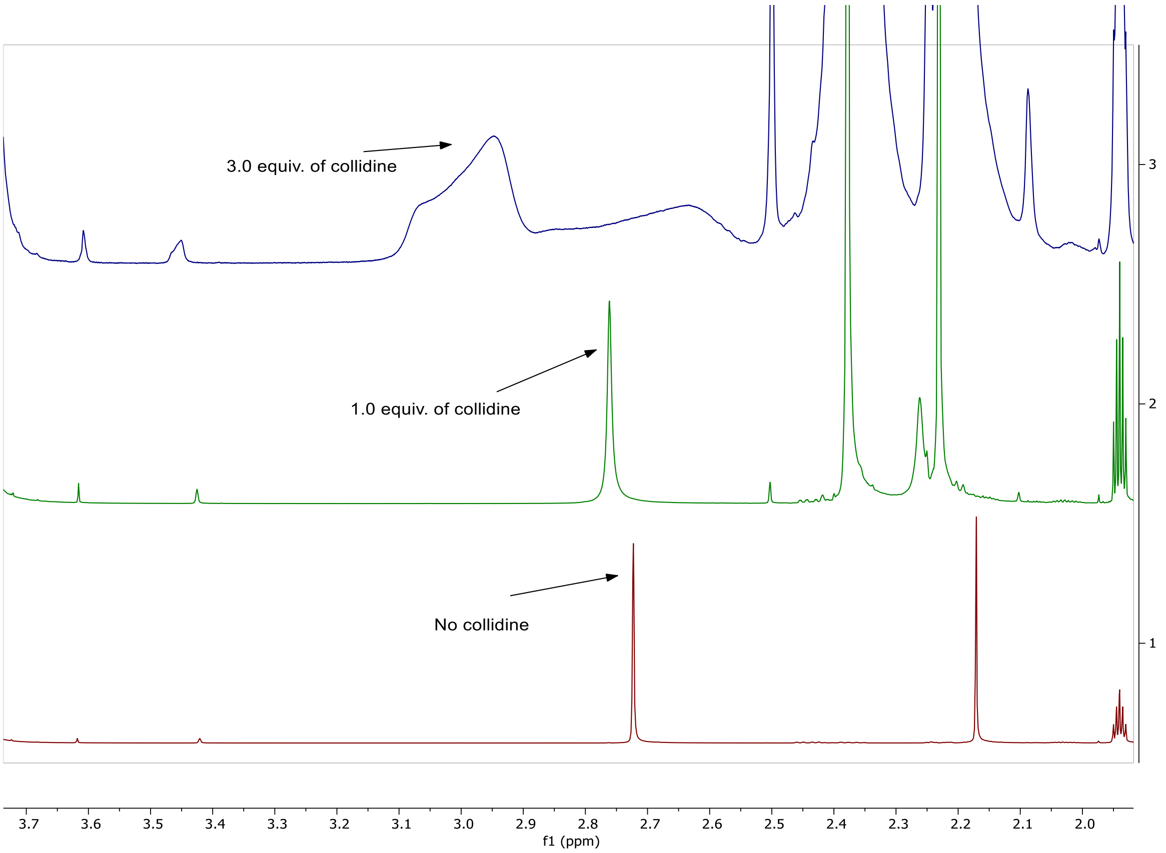

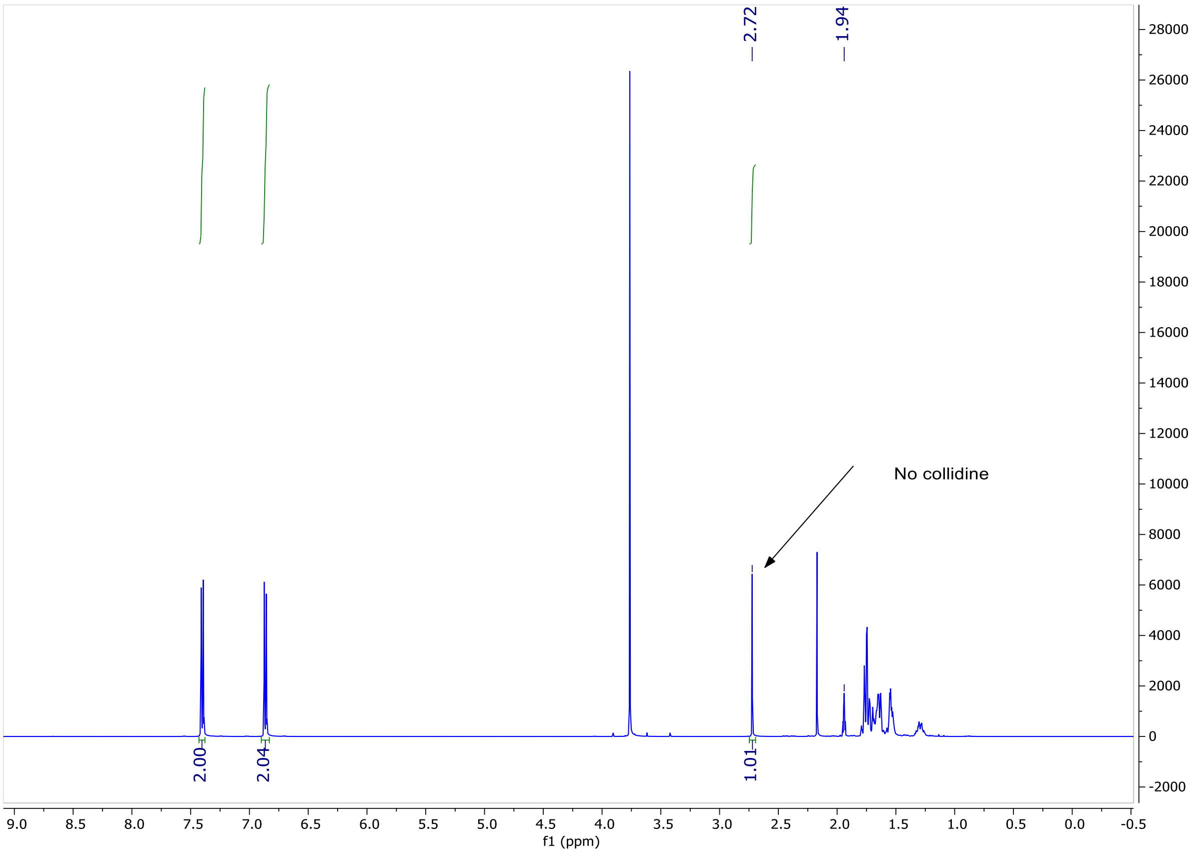

^1H NMR of **1** in CD_3CN with no collidine (2.72 ppm, O – H peak)

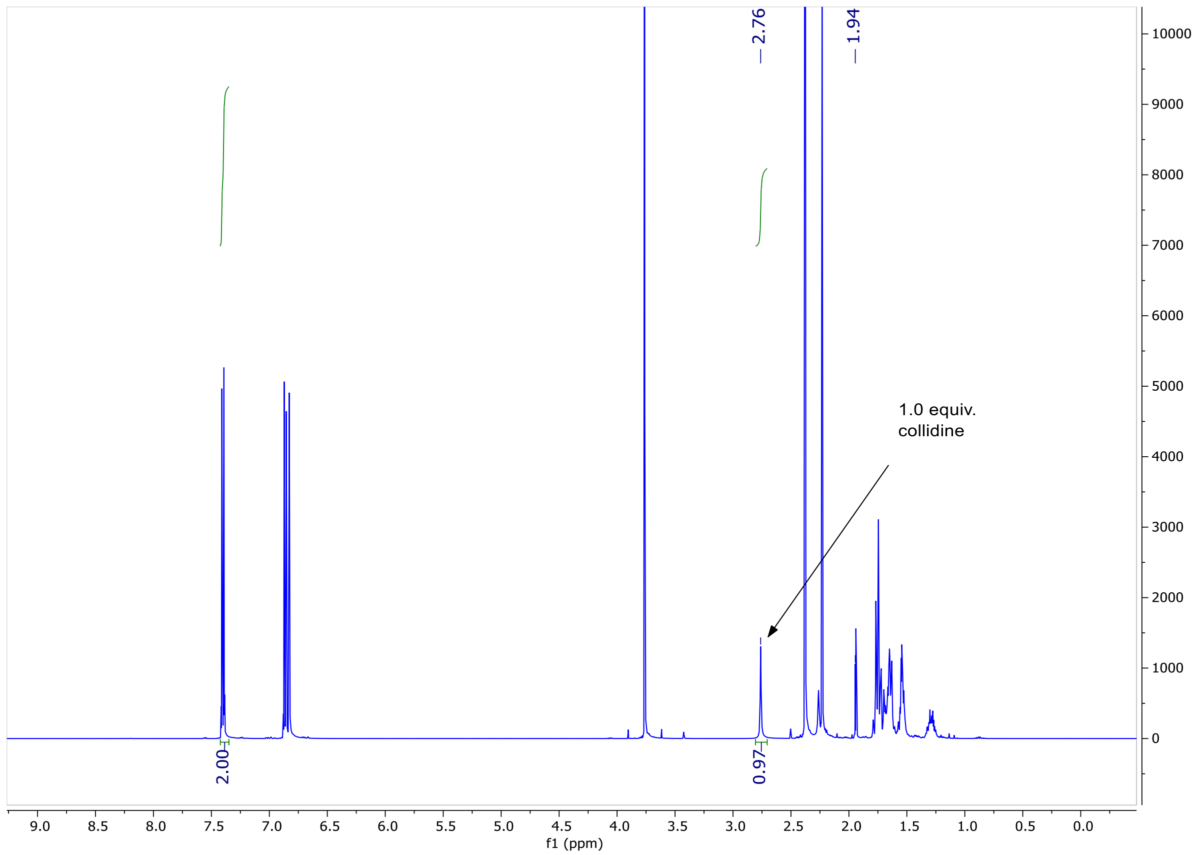

^{1}H NMR of **1** in CD_3CN with 1.0 equiv. of collidine (2.76 ppm, O – H peak)

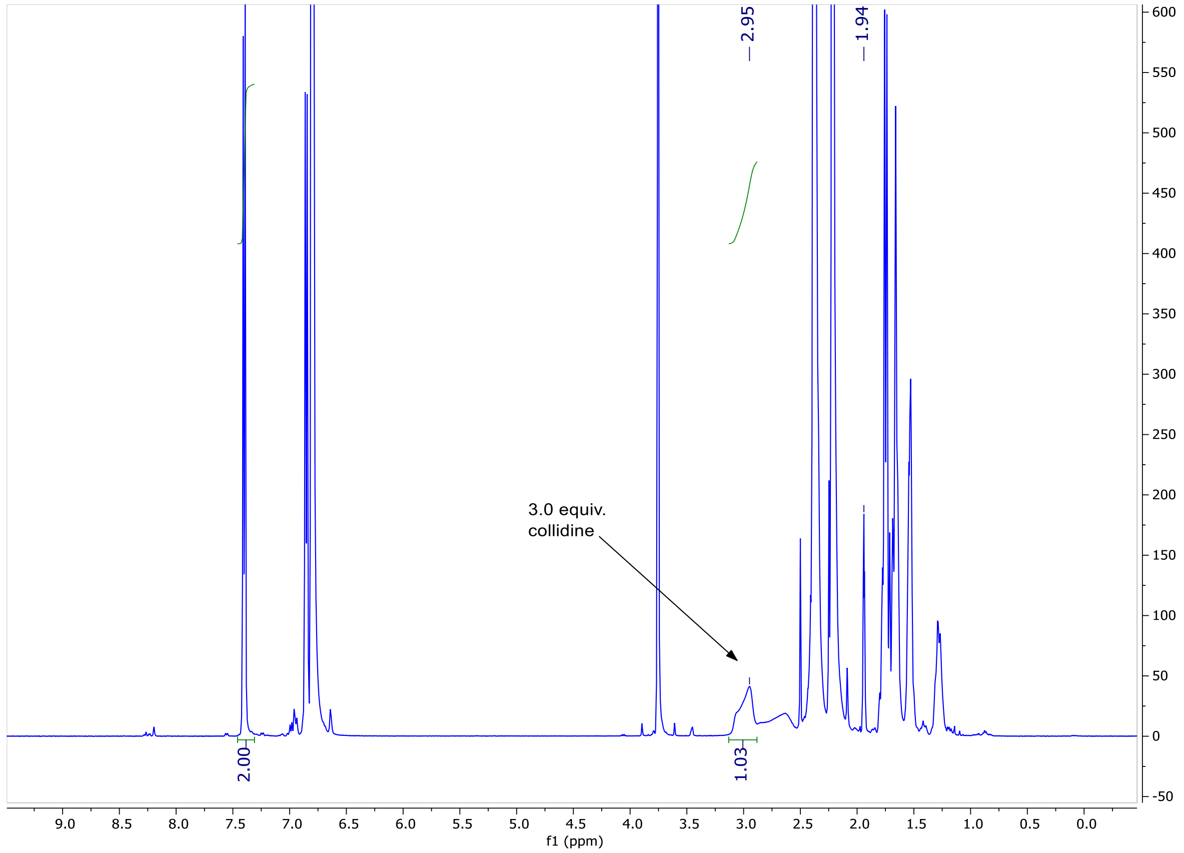

^{1}H NMR of **1** in CD_3CN with 3.0 equiv. of collidine (2.95 ppm, O – H peak)

Evidence for the fate of bromotrichloromethane ($BrCCl_3$)

To determine the fate of the brominating reagent $BrCCl_3$, the parent bromination reaction was run as per the optimized conditions using **1** and the ^{1}H NMR of the crude reaction mixture was recorded in CD_3CN. Peak at 7.57 ppm signifies the formation of chloroform.

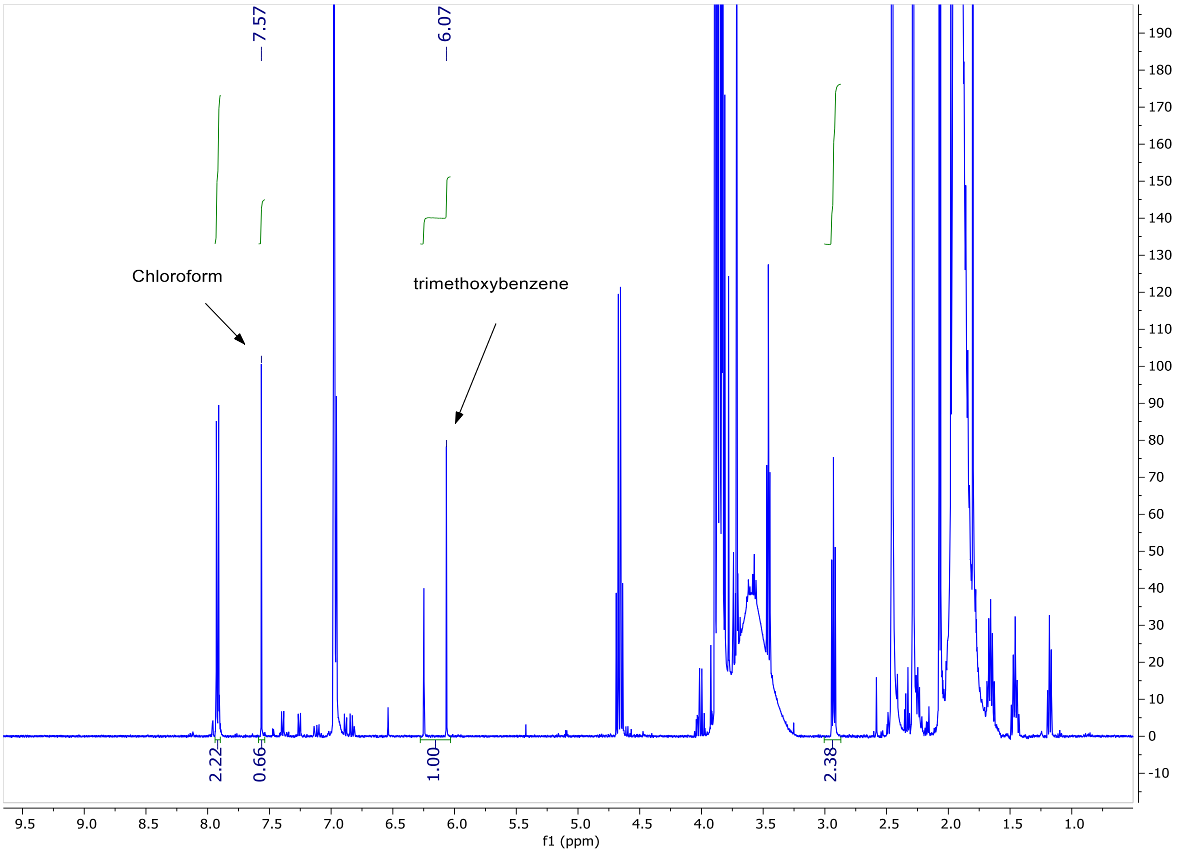

Plausible reaction pathways for the formation of chloroform.[20]

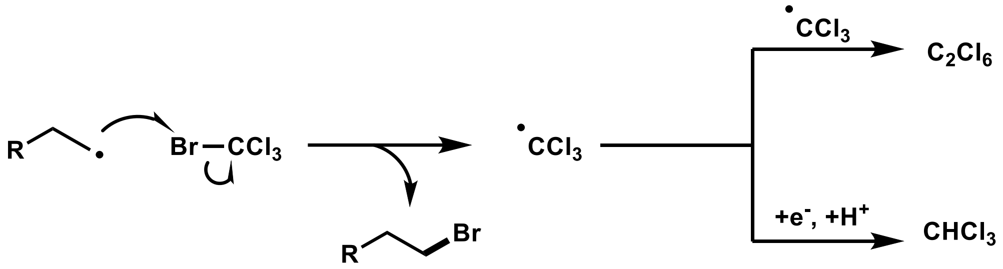

7. References

(1) Wen, Y.; Chen, G.; Huang, S.; Tang, Y.; Yang, J.; Zhang, Y. The Barbier–Grignard-Type Arylation of Ketones and Unexpected Cross-Coupling of Phenolic Ketones Using Unactivated Aryl Bromides. *Adv. Synth. Catal.* **2016**, *358*, 947–957.

(2) Jia, K.; Zhang, F.; Huang, H.; Chen, Y. Visible-Light-Induced Alkoxyl Radical Generation Enables Selective C(Sp3)–C(Sp3) Bond Cleavage and Functionalizations. *J. Am. Chem. Soc.* **2016**, *138*, 1514–1517.

(3) Wang, J.; Huang, B.; Shi, C.; Yang, C.; Xia, W. Visible-Light-Mediated Ring-Opening Strategy for the Regiospecific Allylation/Formylation of Cycloalkanols. *J. Org. Chem.* **2018**, *83*, 9696–9706.

(4) Huang, L.; Ji, T.; Rueping, M. Remote Nickel-Catalyzed Cross-Coupling Arylation via Proton-Coupled Electron Transfer-Enabled C–C Bond Cleavage. *J. Am. Chem. Soc.* **2020**, *143, 3532–3539* ,

(5) Zhang, Z.; Zhang, G.; Xiong, N.; Xue, T.; Zhang, J.; Bai, L.; Guo, Q.; Zeng, R. Oxidative α-C-C Bond Cleavage of 2° and 3° Alcohols to Aromatic Acids with O2at Room Temperature via Iron Photocatalysis. *Org. Lett.* **2021**, *23*, 2915–2920.

(6) Schnaubelt, L.; Petzold, H.; Dmitrieva, E.; Rosenkranz, M.; Lang, H. A Solvent- and Temperature-Dependent Intramolecular Equilibrium of Diamagnetic and Paramagnetic States in Co Complexes Bearing Triaryl Amines. *Dalt. Trans.* **2018**, *47*, 13180–13189.

(7) Yamamoto, K.; Toguchi, H.; Kuriyama, M.; Watanabe, S.; Iwasaki, F.; Onomura, O. Electrophotochemical Ring-Opening Bromination of *Tert* -Cycloalkanols. *J. Org. Chem.* **2021**, 86, 16177–16186.

(8) Yayla, H. G.; Wang, H.; Tarantino, K. T.; Orbe, H. S.; Knowles, R. R. Catalytic Ring-Opening of Cyclic Alcohols Enabled by PCET Activation of Strong O–H Bonds. *J. Am. Chem. Soc.* **2016**, *138*, 10794–10797.

(9) Mečiarová, M.; ˇ tefan Toma, S.; Podlesná, J.; Kiripolsky, M.; Císařová, I. Study of Ultrasound Promoted Aromatic Nucleophilic Substitution of Halobenzenes with Amines. *Monatshefte f€ u ur Chemie* **2003**, *134*, 37–43.

(10) Thamaraiselvi, P.; Varathan, E.; Subramanian, V.; Easwaramoorthi, S. Multipolar Triphenylamines: Effect of Spectator Donor-Acceptor Pair on Intramolecular Charge Transfer Interactions. *Dye. Pigment.* **2020**, *172*, 107838.

(11) Wu, X.; Davis, A. P.; Lambert, P. C.; Kraig Steffen, L.; Toy, O.; Fry, A. J. Substituent Effects on the Redox Properties and Structure of Substituted Triphenylamines. An Experimental and Computational Study. *Tetrahedron* **2009**, *65*, 2408–2414.

(12) Zeng, C. C.; Zhang, N. T.; Lam, C. M.; Little, R. D. Novel Triarylimidazole Redox Catalysts: Synthesis, Electrochemical Properties, and Applicability to Electrooxidative C-H Activation. *Org. Lett.* **2012**, *14*, 1314–1317.

(13) Das Sharma, S.; Hazarika, P.; Konwar, D. An Efficient and One-Pot Synthesis of 2,4,5-Trisubstituted and 1,2,4,5-Tetrasubstituted Imidazoles Catalyzed by InCl3·3H2O. *Tetrahedron Lett.* **2008**, *49*, 2216–2220.

(14) Lan, Y.; Yang, C.; Xu, Y. H.; Loh, T. P. Direct Coupling of Sp3 Carbon of Alkanes with α,β-Unsaturated Carbonyl Compounds Using a Copper/Hydroperoxide System. *Org. Chem. Front.* **2017**, *4*, 1411–1415.

(15) Fan, X.; Zhao, H.; Yu, J.; Bao, X.; Zhu, C. Regiospecific Synthesis of Distally Chlorinated Ketones via C–C Bond Cleavage of Cycloalkanols. *Org. Chem. Front.* **2016**, *3*, 227–232.

(16) Bogert, M. T.; Curtin, L. P. The Relation Between Molecular Structure and Odor in Tri-Substituted Benzenes.I. Derivatives of Para-Methoxy-Acetophenone *J. Am. Chem. Soc.* **2002**, *45*, 2161–2167.

(17) Yang, Y. M.; Yan, W.; Hu, H. W.; Luo, Y.; Tang, Z. Y.; Luo, Z. Photoinduced Acetylation of Anilines under Aqueous and Catalyst-Free Conditions. *J. Org. Chem.* **2021**, *86*, 12344–12353.

(18) Shi, J. L.; Wang, Y.; Wang, Z.; Dou, B.; Wang, J. Ring-Opening Iodination and Bromination of Unstrained Cycloalkanols through β-Scission of Alkoxy Radicals. *Chem. Commun.* **2020**, *56*, 5002–5005.

(19) Jones, A. C.; Nicholson, W. I.; Leitch, J. A.; Browne, D. L. A Ball-Milling-Enabled Cross-Electrophile Coupling. *Org. Lett.* **2021**, *23*, 6337–6341.

(20) Leas, D. A.; Dong, Y.; Vennerstrom, J. L.; Stack, D. E. One-Pot, Metal-Free Conversion of Anilines to Aryl Bromides and Iodides. *Org. Lett.* **2017**, *19*, 2518–2521..
